# Understanding ketone hydrogenation catalysis with anionic iridium(iii) complexes: the crucial role of counterion and solvation†

**DOI:** 10.1039/d4sc04629c

**Published:** 2024-11-11

**Authors:** Paven Kisten, Sandrine Vincendeau, Eric Manoury, Jason M. Lynam, John M. Slattery, Simon B. Duckett, Agustí Lledós, Rinaldo Poli

**Affiliations:** a CNRS, LCC (Laboratoire de Chimie de Coordination), Université de Toulouse, UPS, INPT 205 Route de Narbonne, BP 44099, F-31077 Toulouse Cedex 4 France rinaldo.poli@lcc-toulouse.fr +33-561553003 +33-561333174; b Department of Chemistry, University of York Heslington York YO10 5DD UK john.slattery@york.ac.uk; c Departament de Química, Universitat Autònoma de Barcelona 08193 Cerdanyola del Vallès, Catalonia Spain Agusti.Lledos@uab.cat; d Institut Universitaire de France 1, Rue Descartes, 75231 Paris Cedex 05 France

## Abstract

Catalytic asymmetric hydrogenation of ketones is an important approach to prepare valuable chiral alcohols. Understanding how transition metals promote these reactions is key to the rational design of more active, selective and sustainable catalysts. A highly unusual mechanism for asymmetric hydrogenation of acetophenone catalysed by an anionic Ir^III^ hydride system, including a strong counterion dependence on catalyst activity, is explored and rationalised here. The active catalyst, generated *in situ* from [IrCl(COD)]_2_ and a bidentate ligand (P,S^R^) under H_2_ in the presence of a strong base (M^+i^PrO^−^ in isopropanol, M = Li, Na, K), is the solvated M^+^[Ir(H)_4_(P,S^R^)] salt (P,S^R^ = CpFe[1,2-C_5_H_3_(PPh_2_)(CH_2_S^R^)], with R = ^i^Pr, Ph, Bz and Cy). Catalyst activity increases, for all R derivatives, significantly as the counterion is varied in the order Li < Na < K. For the most active K system, the addition of 18-crown-6 drastically reduces the activity. While the cation strongly affects catalyst activity, it does not significantly affect enantioselectivity. DFT calculations explored these effects in detail and showed that the solvation model used in the calculations is critical. Only a hybrid implicit/explicit solvent model including sufficient explicit solvent molecules to properly describe the first solvation shell of the cation is able to reproduce the experimental observations. This model revealed the fundamental importance of the alkali-metal cation coordination sphere in understanding the counterion effects. The turnover-determining states in the catalytic cycle are those involved in outer-sphere hydride transfer to the substrate. This step leads to coordination of the alkoxide product to the alkali-metal cation, with a significant rearrangement of the coordination sphere of M, whereas there is little change in the geometrical parameters around Ir or the alkoxide. The DFT calculations also pinpointed the major enantio-discriminating interactions and rationalised the insensitivity of the enantioselectivity on the alkali metal cation placement.

## Abstract 2

A highly unusual mechanism for asymmetric hydrogenation catalysed by an anionic Ir^III^ hydride system, including a strong counterion dependence on catalyst activity, is explored and rationalised.

## Graphical abstract

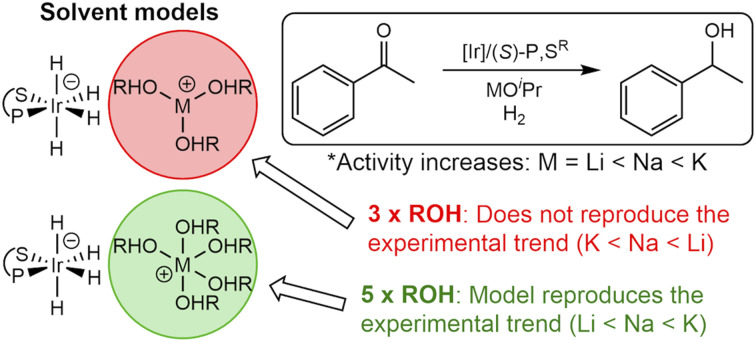

## Introduction

The asymmetric hydrogenation of polar prochiral substrates, particularly aryl ketones, is a highly important route to valuable chiral alcohols.^1–6^ Transition metal catalysis can prove effective for these reactions, but catalyst activation, speciation in solution and catalytic reaction mechanisms are not always well understood. A detailed understanding of how metals can promote these transformations is important to allow the rational design of more active, selective and sustainable catalysts. In many reactions a pre-catalyst, for example [IrCl(COD)]_2_, is used to deliver the metal to the system. For most pre-catalysts, the presence of a strong base is required to afford high activities. The role of the base is usually attributed to the stabilisation of a more active neutral hydride species, derived from the pre-catalyst, relative to cationic species that may otherwise form in the protic reaction solvents (typically alcohols) that are used.^7^ When a chloride-containing complex is used as a pre-catalyst, as is often the case, the base is also invoked in the activation step, where substitution of the chloride by an alkoxide produced by deprotonation of the alcohol solvent (*e.g.* isopropoxide when the reactions are conducted in isopropanol) is followed by β-H elimination.^8^ However, in some of our laboratories we have demonstrated that this rationale is insufficient,^9^ at least for ketone hydrogenations catalysed by [IrCl(COD)]_2_/LL’ (COD = 1,5-cyclooctadiene), where LL’ is a planar chiral 1,2-disubstituted ferrocene ligand containing diphenylphosphino and thioether donor groups, CpFe[1,2-C_5_H_3_(PPh_2_)(CH_2_SR)] (R = Et, ^i^Pr, ^*t*^Bu, Bz, Ph, *etc.*),^10^ henceforth abbreviated as (P,S^R^).

**Scheme 1:**
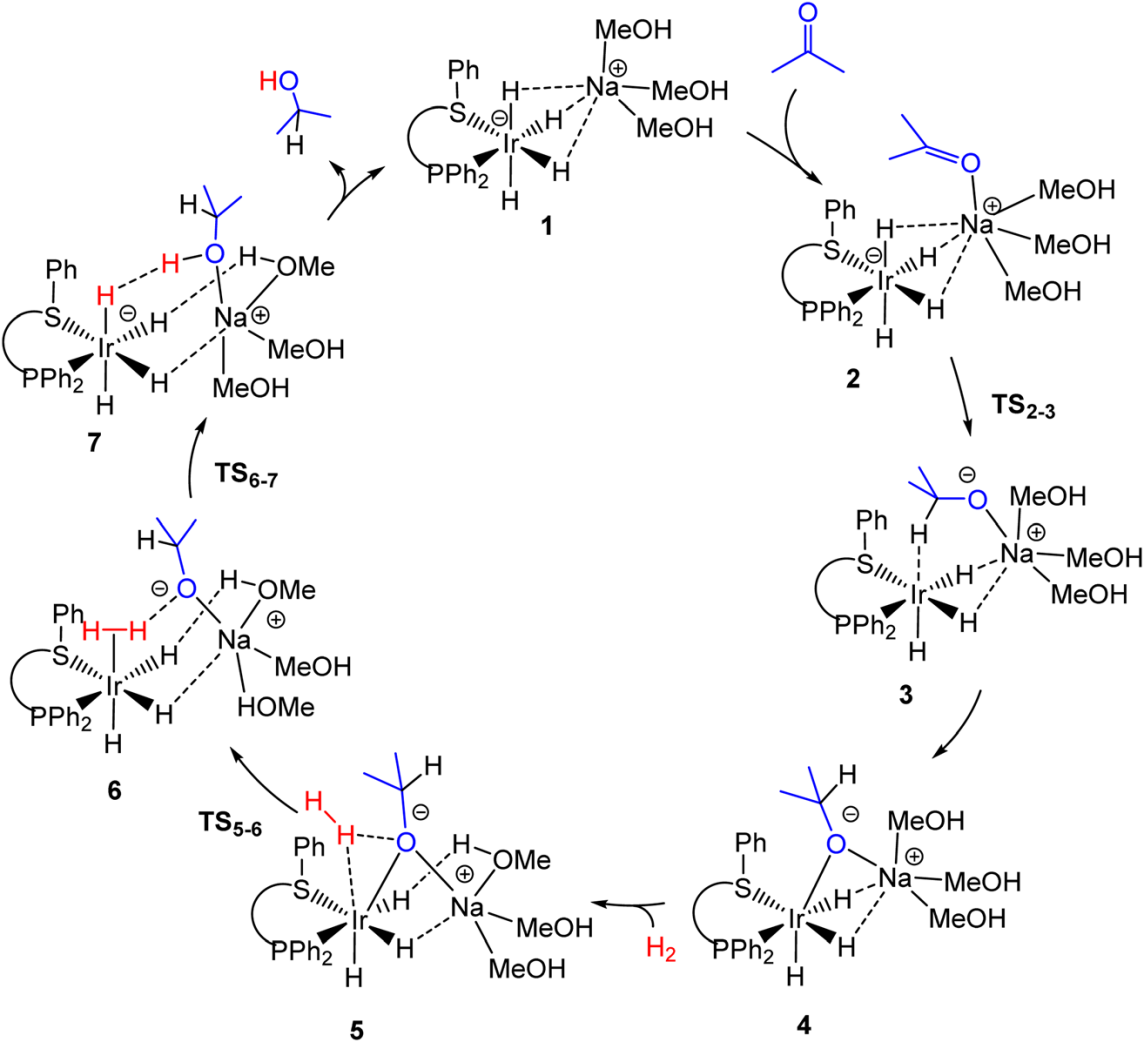
Schematic catalytic cycle for ketone hydrogenation catalysed by [IrCl(COD)((*S*)-P,S^Ph^)]/NaOMe, according to a DFT investigation.^9^ Note that the coordination geometries around the metals and solvent interactions vary with the alkali metal used, as described below.

The stoichiometric reaction between [IrCl(COD)]_2_ and ((*S*)-P,S^R^) was shown to yield well-defined [IrCl(COD)((*S*)-P,S^R^)] complexes with either a 5-coordinate square pyramidal^11^ or a 4-coordinate square planar^12^ (for R = ^*t*^Bu) geometry. These complexes (either isolated or made *in situ*) proved highly active catalysts and led to excellent enantioselectivity in aromatic ketone hydrogenation, though only in the presence of an excess of a strong base (*e.g.* 5 equivalents of NaOMe per Ir).^13^ However, the use of an alternative precursor, [Ir(OMe)(COD)]_2_, which contains an internal methoxide base, alongside the (P, S^R^) ligand still required the presence of additional strong base to yield an active catalyst, suggesting an anionic active catalyst is formed under the reaction conditions.^9^ A parallel DFT investigation revealed that the most stable species generated by a sequence of COD hydrogenation and H_2_ oxidative addition steps is the anionic tetrahydrido complex [Ir(H)_4_((*S*)-P,S^R^)]^−^. The latter is therefore a strong candidate for the catalyst resting state. This complex does not contain mobile protons for a classical outer-sphere bifunctional (Noyori-type)^7^ mechanism, nor a vacant coordination site for an inner-sphere mechanism. Instead, the DFT calculations rationalised the catalytic action through a novel mechanistic variant of the outer-sphere bifunctional mechanism, which is summarised in Scheme 1. This involves a cooperative action between the alkali-metal cation for ketone activation and the iridium centre for hydride transfer to the carbonyl carbon atom, thereby generating an alkoxide intermediate. This step is followed by transfer of a proton originating from heterolytic cleavage of H_2_ by the alkoxide and the Ir centre. The calculated Gibbs energy span of the resulting catalytic cycle, 18.2 kcal mol^−1^, was consistent with the experimental activity.^9^ However, the effect of the [Ir(H)_4_((*S*)-P,S^R^)]^−^ counterion was not explored and yet has proved to be a highly important feature of this system, significantly affecting its activity, as described below.

The extreme lability of the [Ir(H)_4_((*S*)-P,S^R^)]^−^ complex has so far not allowed us to isolate or indeed spectroscopically identify it. However, we have obtained a related complex with the chelating diphenylphosphinoethane (dppe) ligand, [Ir(H)_4_(dppe)]^−^, under identical conditions to those used for hydrogenation catalysis (from [IrCl(COD)]_2_/dppe in basic isopropanol under H_2_ at room temperature) and also under transfer hydrogenation conditions (no H_2_ gas added), even though the latter required warming to 80 °C.^14^ Analogous anionic tetrahydrido complexes of Ir^III^ were previously reported in the chemistry of monodentate phosphine ligands, [Ir(H)_4_L_2_]^−^ (L = P^i^Pr_3_, PPh_3_), obtained by reaction of [Ir(H)_5_L_2_] with KH, but not under catalytic conditions.^15,16^ This gives further credence to the implication of anionic [Ir(H)_4_((*S*)-P,S^R^)]^−^ as the active species in ketone hydrogenation with [IrCl(COD)]_2_/((*S*)-P,S^R^).

The action of anionic hydride complexes as (transfer) hydrogenation catalysts, and their generation by strong bases, has not been widely acknowledged or studied. In early work, G. P. Pez *et al.* suggested the involvement of anionic Ru hydride complexes, *e.g.* [K(L_*n*_)][RuH_2_(*κ*^2^:C,P-*o*-C_6_H_4_PPh_2_)(PPh_3_)_2_] and [K(L_*n*_)][(PPh_3_)_3_(PPh_2_)RuH_2_] (L_*n*_ = solvent or crown ether), as active species for the catalytic reduction of aromatic compounds as well as aldehydes, ketones, esters and nitriles.^17–21^ Of special note is the fact that the addition of either a crown ether or cryptand was shown to dramatically reduce the catalytic activity.^19^ However, subsequent work by J. Halpern *et al.* demonstrated that the anionic [Ru(H)_3_(PPh_3_)_3_]^−^ complex, which is generated from the Pez systems under H_2_,^22,23^ is reversibly protonated in alcohol solvents to form [Ru(H)_2_(H_2_)(PPh_3_)_3_] and that the latter “tetrahydride” complex is the real catalytically active species.^24^ After these debated contributions, the role of anionic hydride species in transition metal catalysed hydrogenation was not considered again, until recently.

Just prior to our above-mentioned contribution, Dub *et al.* suggested that the active species of Noyori's [Ru(H)_2_(diphosphine)(NH_2_CHPhCHPhNH_2_)] catalyst is, in fact, the deprotonated amine–amido complex [K(L)_*n*_][Ru(H)_2_(diphosphine)(NHCHPhCHPhNH_2_)].^25^ DFT calculations carried out for that system underlined the activating action of the N^−^K^+^ function in the rate-determining hydride transfer step to the outer-sphere substrate, though the potassium ion was introduced in the calculation as a naked ion, which does not reflect the solvation of this ion under catalytic conditions. Beller's Ru PNP pincer complex [RuH(CO)Cl{HN(C_2_H_4_P^i^Pr_2_)_2_}], which catalyses the aqueous dehydrogenation of methanol under highly basic conditions,^26^ was suggested to operate *via* an anionic deprotonated pincer hydride resting state on the basis of DFT calculations. However, these were carried out on the free anion, without consideration of a possible cation effect.^27^ The ester hydrogenation catalysed by [MnBr(CO)_3_(Ph_2_CH_2_CH_2_NH_2_)] under strongly basic conditions^28^ was also proposed by Pidko *et al.*, on the basis of a DFT investigation,^29^ to proceed *via* an anionic species with a deprotonated ligand, [MnH(CO)_3_(Ph_2_CH_2_CH_2_NH)]^−^. These calculations, like the above-mentioned ones by Dub *et al.*, included only the naked cation and a comparison of the NH and N^−^M^+^ (M = Li, Na, K) systems suggested that, while the barrier of the rate-determining hydride transfer step is reduced on going from the neutral to the anionic system, the cation effect is only minor. A cation effect, however, was not experimentally probed for this manganese catalyst. The [IrCl(COD)]_2_/HL system with HL = tridentate ferrocene-based amino-phosphine-sulphonamide (f-amphamide), used as an asymmetric catalyst for ketone transfer hydrogenation, was also shown by Dang, Zhang *et al.* to operate *via* an anionic hydride complex as a resting state, *i.e.* [Ir(H)_3_L]^−^.^30^ For the latter system, DFT calculations suggested an activating role of Li^+^, which was again introduced in the calculations as a naked ion. Finally, a most recent contribution by Bai, Lang, Zhang *et al.* has shown an extremely active and enantioselective anionic Ir^III^ hydride complex with a tetradentate PNNO ligand (f-phamidol), which hydrogenates acetophenone with a TOF of 224 s^−1^ up to a TON of over 13 million and up to 99% ee.^31^ In this contribution, the cation (Na^+^) was explicitly introduced in the calculations and the effect of the cation solvation (up to two isopropanol molecules per Na^+^) was considered, but a possible effect of the cation nature was not explored, neither experimental nor computationally.

For an anionic active catalyst, the activity may somewhat be affected by the nature of the counter-cation. In addition to the above-mentioned investigation by Pez *et al.*,^19^ the effect of an alkali-metal counterion on the activity (and/or selectivity) of a (transfer) hydrogenation catalyst has been pointed out in a few cases but has generally be attributed to cooperation with a neutral hydride system. A notable example is the [{RuCl_2_(*p*-cymene)}_2_]-pseudodipeptide-catalysed enantioselective transfer hydrogenation of ketones, where the alkali-metal cation was suggested to act in a Meerwein–Ponndorf–Verley fashion in combination with a neutral Ru^II^ hydride in the rate-limiting hydride transfer step.^32,33^ In a series of papers by Hazari *et al.*,^34–36^ alkali-metal cation salts were shown to affect the activity of Fe pincer hydride complexes in methanol dehydrogenation. In this case, the DFT studies suggested that the cation accelerates the formate product extrusion, but again in cooperation with a neutral active species and a similar conclusion was reached for the related Ru catalyst.^37^ Very recently, it was found that countercations can exert a remarkable influence on the ability of anionic cobaltate salts to catalyze alkene hydrogenations.^38^ Experimental and computational studies suggested an active co-catalytic role of the counterion (K^+^, Na^+^, Li^+^, Mg^2+^) in the hydrogenation reaction through coordination to cobalt hydride intermediates.^38^ However, despite the importance of these effects, detailed studies and understanding of countercation effects in catalysis are still very rare. In contrast, anion effects in homogeneous catalysis using cationic metal complexes, *e.g.* Au^+^ systems,^39,40^ olefin polymerisation catalysis,^41^ or in asymmetric counteranion-directed catalysis,^42,43^ have been more widely studied.

In the present contribution, we show the results of catalytic studies for the acetophenone hydrogenation carried out in the presence of strong bases with different alkali metal cations, where pronounced alkali-metal ion effects are apparent. Extensive DFT investigations pinpoint the role of the alkali-metal cation in ketone activation and its assistance in the rate- and enantio-determining hydride-transfer step. A key aspect that emerged during the DFT study was the importance of the solvent model used for the correct modelling of the catalytic cycle. Quite surprisingly, a major effect was revealed for the rearrangement of the alkali-metal coordination sphere, which must be described by an appropriate number of explicit solvent molecules. These observations are important not only for the present work, but to inform the development of computational methodologies for studying many homogeneous catalytic reactions where solvent plays an underappreciated role.

## Results and discussion

### Catalytic investigations

The effect of the alkali-metal cation on the catalytic activity of the system generated from [IrCl(COD)((*S*)--P,S^R^)]/MO^i^Pr/H_2_ (M = Li, Na, K) was probed for four different ((*S*)-P,S^R^) ligands (R = ^i^Pr, Ph, Bz, Cy), under similar conditions to those of our initial investigation, namely hydrogenation under 30 bar of H_2_ pressure at 3 °C with a 0.2% catalyst loading and with activation by 1% strong base (5 equiv. relative to catalyst) in isopropanol.^13^ It is useful to recall here that no activity is obtained unless the neutral Ir^I^ precatalyst is activated by a strong alkali metal base, which is rationalised by the conversion to the active anionic [IrH_4_((*S*)--P,S^R^)]^−^ complex.^9^ Alkoxide bases (NaOMe, KO^*t*^Bu) and even KOH were found able to activate the precatalyst, whereas no activity resulted in the presence of NEt_3_.^13^ The Ir complexes with Ph- and Bz-substituted ligands were previously reported,^12,13^ whereas those with the ^i^Pr- and Cy-substituted ligands have been used here as hydrogenation catalysts for the first time. The operating base in entries 1–5 (Table 1) is isopropoxide, because the employed KO^*t*^Bu in the experiments of entries 3 and 4 is a stronger base and thus the equilibrium position in HO^i^Pr strongly favours the formation of isopropoxide. The results of entries 1–3 indicate a significant effect of the alkali-metal counterion associated with the base on the catalytic activity, confirming the proposition that there is an explicit role for the alkali metal cation in the catalytic cycle. Notably, all four ((*S*)-P,S^R^) systems show the same trend of activity (Li^+^ < Na^+^ < K^+^). When using the same cation, the isopropyl system consistently gives the highest catalytic activity. The addition of 18-crown-6 to the potassium system (entry 4) results in a dramatic quenching of the catalytic activity. Once again, a comparable effect is shown by all four ((*S*)-P,S^R^) systems. This result is also in perfect agreement with the proposed catalytic cycle (Scheme 1), because the high affinity of this particular crown ether for K^+^ is expected to weaken the ability of the cation to ion pair with the anionic catalyst. In this respect, it is interesting to note that the previously reported [Ir(H)_4_L_2_]^−^ (L = P^i^Pr_3_, PPh_3_) complexes were crystallised and structurally investigated as crown-ether adducts of the K^+^ salts.^15,16^ Furthermore, all structures that feature *cis* L ligands also reveal K⋯H interactions with three *mer* hydride ligands, as found by the DFT calculations for the [Na(MeOH)_3_][Ir(H)_4_((*S*)-P,S^Ph^)] model (1 in Scheme 1). The deliberate addition of degassed water to the reaction mixtures has little or no effect on the catalytic activities (*cf.* entries 3 and 5). Finally, using the tetramethylammonium cation (entry 6) also resulted in reduced activity. This result is interesting when compared with the identical activities reported (for the Et-substituted ligand system) in the presence of KO^*t*^Bu and KOH.^13^ Thus, the activity drop for the Cy-substituted ligand system from entry 3 to entry 6 cannot be attributed to the weaker basicity of OH^−^ in isopropanol, but rather to the replacement of K^+^ with Me_4_N^+^.

**Table 1:** Results of the acetophenone hydrogenation using different (*S*-P,S^R^) ligands and bases^a^

Entry	Base (+additive)	Conversion/%^b^ (ee^c^/%)			
		R = ^i^Pr^d^	R = Ph^e^	R = Bz^d^	R = Cy^e^
1	LiO^i^Pr	20 (34)	12 (65)	8 (78)	2 (69)
2	NaO^i^Pr	61 (51)	26 (60)	19 (80)	60 (72)
3	KO^*t*^Bu	90 (58)	34 (66)	21 (78)	70 (75)
4	KO^*t*^Bu + 18-crown-6^f^	14 (60)	12 (60)	4 (81)	14 (67)
5	KO^*t*^Bu + H_2_O^g^	93 (29)	30 (67)	23 (50)	84 (75)
6	Me_4_NOH	—	—	—	10 (23)

a Conditions: *p*(H_2_) = 30 bar; *t* = 4 h; *T* = 3 °C; [PhCOMe] = 1.6 M in isopropanol; [Ir] = 3.2 × 10^−3^ M; [base] = 1.6 × 10^−2^ M.

b Determined from the area of the residual acetophenone in the gas-chromatogram in comparison with calibration curves (estimated error = ± 1.7%).

c Determined from the relative areas of the two phenylethanol enantiomers separated on a chiral column (estimated error = ± 1.5%); the *S* enantiomer is the major one.

d Pre-catalyst prepared *in situ* from [IrCl(COD)]_2_ and ((*S*)-P,S^R^).

e With isolated [IrCl(COD)(PS^R^)].

f 18-crown-6/K = 1.5.

g H_2_O/K = 1.5.

The observed enantioselectivity trends are also interesting. The highest ee values were obtained for the Bz system, in agreement with the previous report.^13^ For this system, as well as for the Ph and Cy systems, all three alkali-metal cations yield essentially the same ee, except for a significant drop for the Cy system when using Me_4_N^+^ instead of an alkali metal cation. For the ^i^Pr system, however, the nature of the cation significantly affects the ee, which decreases in the order K^+^ > Na^+^ > Li^+^. It is particularly interesting to note that, for all ((*S*)-P,S^R^) systems, the presence of the crown ether, although strongly quenching the activity, does not significantly affect the ee. Conversely, the presence of water, although not significantly changing the activity, results in an ee decrease of approximately a factor of 2 for the ^i^Pr and Bz systems. The Ph and Cy systems, on the other hand, yield ee similar to those of entries 1–3.

### DFT investigations of the cation effect

We have performed DFT calculations to disclose the influence of the cation on the catalyst activity, using a hybrid implicit-explicit (cluster-continuum) solvent model in which, in addition to the continuum model, five explicit solvent molecules have been introduced. The goal of the calculations is, after finding a model that reproduces the experimental trend, to identify its origin. We are aware that to obtain more quantitative agreements would require the use of very expensive DFT-based Molecular Dynamics (DFT-MD) simulations, placing the reactive system inside a box with a huge number of solvent molecules. This is out of our scope, and we will show that, with a careful description of the solvent, static DFT calculations are able to capture the main features without performing these highly demanding DFT-MD calculations. As we will show in what follows the main point is that a complete description of the first solvation sphere of the cation is mandatory to reproduce the experimental trends.

**Scheme 2:**
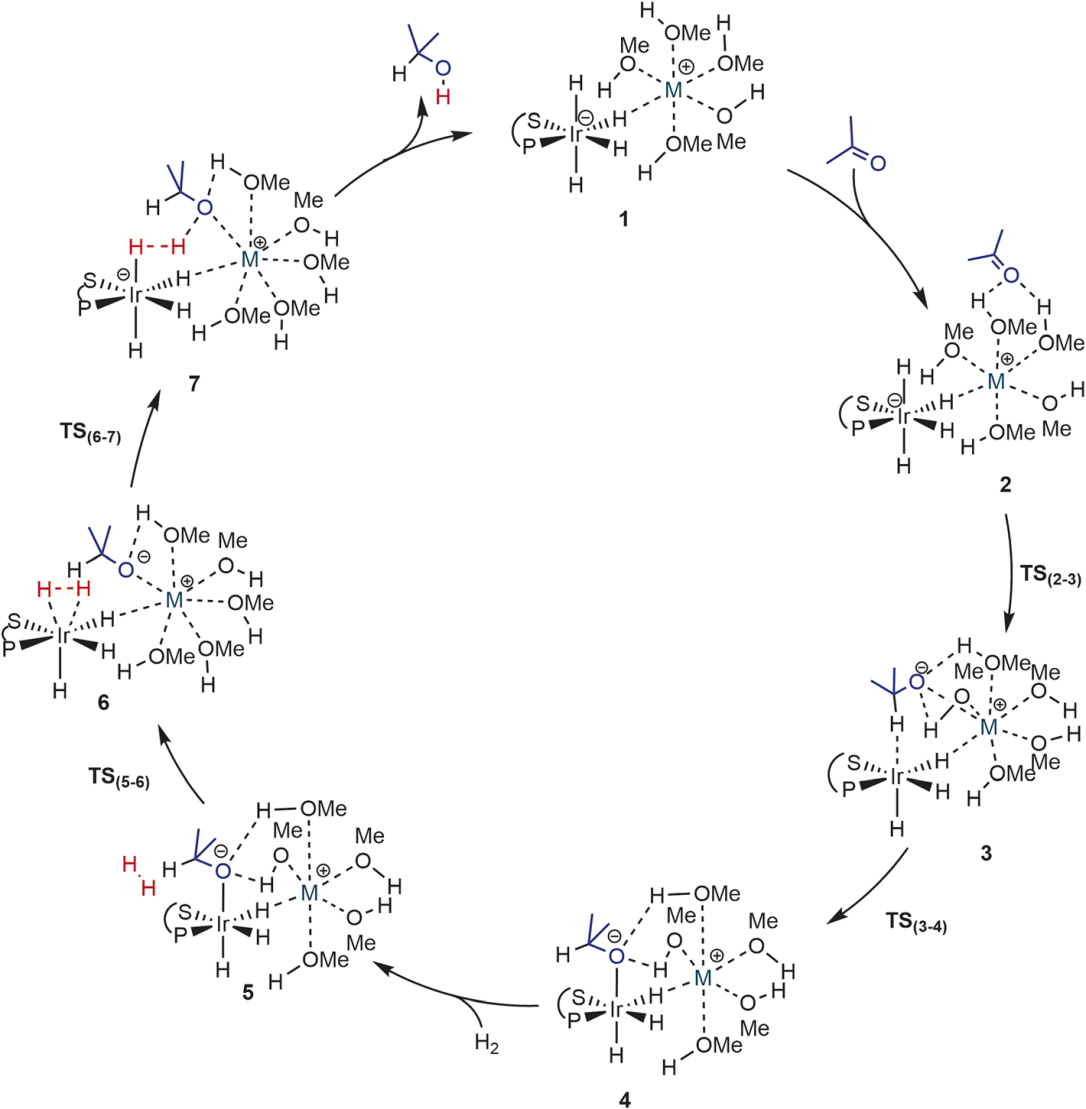
Catalytic cycle for the hydrogenation of acetone with [M(MeOH)_5_][Ir(H)_4_((*S*)-P,S^Ph^)] (M = Li^+^, Na^+^, K^+^).

**Fig. 1:**
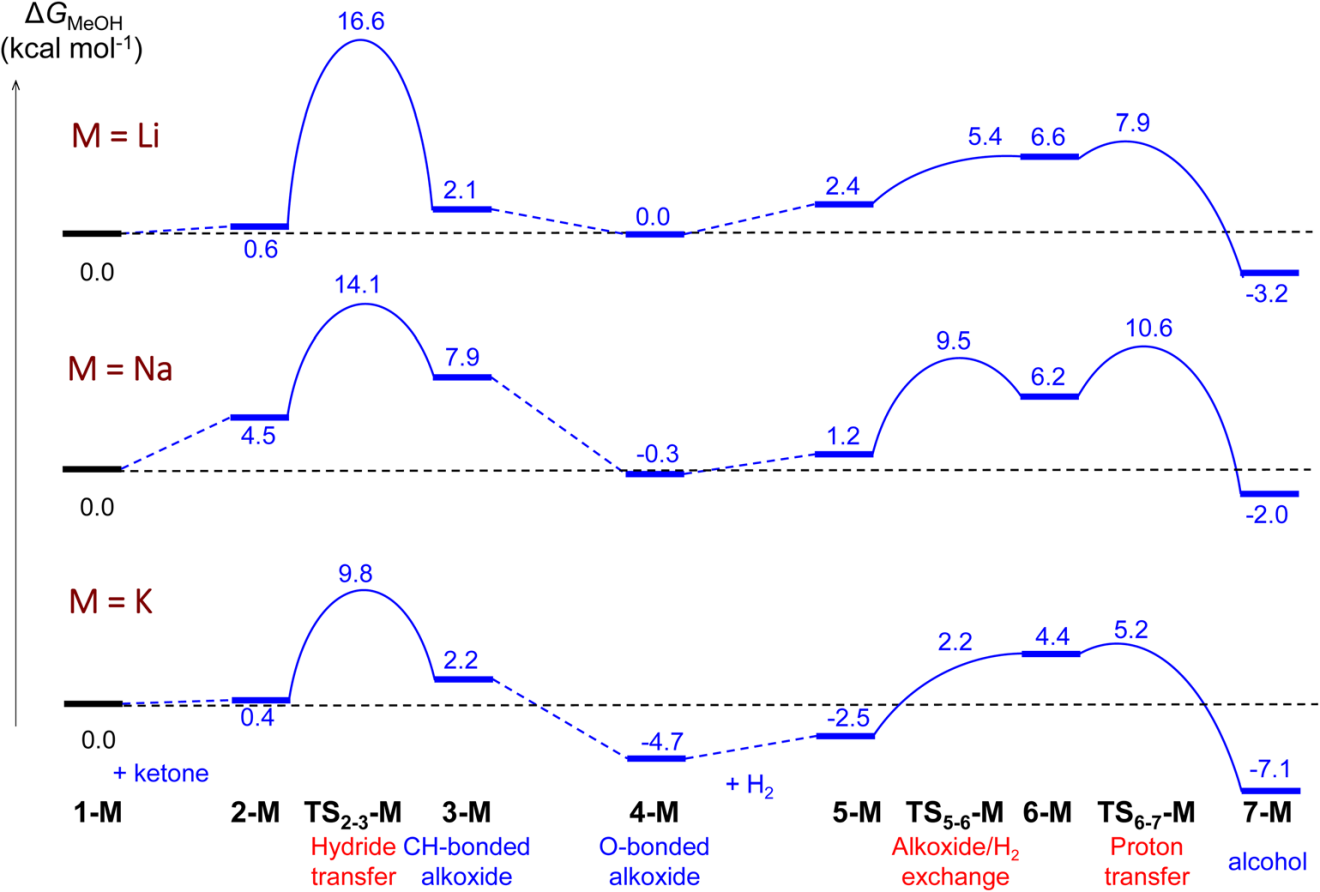
Computed Gibbs energy profiles for ketone hydrogenation catalysed by [M(MeOH)_5_]^+^[Ir(H)_4_((*S*)-P,S^Ph^)]^−^ (M = Li, Na, K). The profile follows the catalytic cycle of Scheme 2, with 5 MeOH molecules explicitly included in the calculations.

Using the cluster-continuum solvent model with five solvent molecules, the (*S*)-P,S^Ph^ ligand without any truncation, and acetone as the model substrate molecule, we have computed all the steps of the catalytic cycle of ketone hydrogenation depicted in Scheme 2 for the three alkali cations, initially modelling the isopropanol solvent used experimentally as methanol. The computed Gibbs energy profiles are shown in Fig. 1. However, once the rate determining step (hydride transfer) was identified, we reoptimised all the structures along this step using isopropanol in the hybrid continuum explicit (five solvent molecules) solvent model, in order to provide a solvent description more akin to the experimental system. This approach has allowed the reproduction of the experimental activity trend: K^+^ > Na^+^ > Li^+^. As we will discuss later, an accurate description of the first solvation sphere of the cation is crucial if DFT is to match the experimental trend. This was already achieved using [M(MeOH)_5_]^+^. Contrarily, using the previously reported model^9^ with only three methanol molecules ([M(MeOH)_3_]^+^), we found that the experimental trend was not mirrored in the computational study, *i.e.* Li^+^ would be expected to show the highest activity. We will discuss this issue later on.

As can be seen from Fig. 1, the cation has an effect on the energy span of the catalytic cycle. This energy span corresponds to the difference between the transition state of the outer-sphere hydride transfer (TS_2-3_) and the initial state, 1, which is the {[Ir(H)_4_((*S*)-P,S^R^)]^−^; [M(MeOH)_5_]^+^} ion pair plus an acetone molecule infinitely apart. The barrier of the hydride transfer step changes appreciably with the cation, following the experimental trend: K^+^ (9.8 kcal mol^−1^) < Na^+^ (14.1 kcal mol^−1^) < Li^+^ (16.6 kcal mol^−1^). After this step, which generates an O-coordinated alkoxide 4*via* a less stable isomeric σ-complex 3, the cation influence is much smaller. From 4, H_2_ enters into play, first displacing the alkoxide (TS_5-6_) to form a η^2^-H_2_ complex (6). The latter is able to protonate the displaced alkoxide *via*TS_6-7_, yielding the alcohol (7) and regenerating complex 1. The proton transfer TS (TS_6-7_) is the highest point in this pathway, and it is found 7.9, 10.9 and 9.9 kcal mol^−1^ above 4, for Li, Na and K cations, respectively, thus the barrier to proton transfer from 4 is lower than the hydride transfer barrier for Li^+^ and Na^+^, whereas the two barriers are effectively the same for K^+^.

**Fig. 2:**
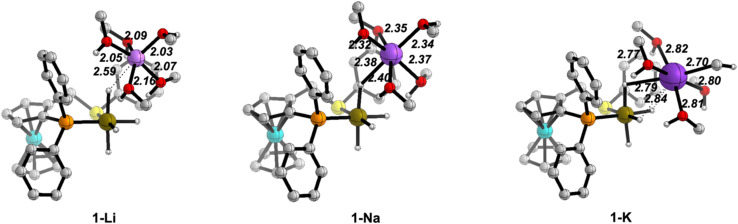
Optimised structures of the iridium complex/cation ion pairs (1) with the (MeOH)_5_ solvent model. Distances in Å. C–H hydrogen atoms have been omitted for clarity.

We will present selected structures to discuss the changes in the cation solvation sphere along the catalytic pathway for the three ions. 3D plots of all the optimised structures are collected in Fig. S6–S9 in the ESI.†Fig. 2 displays the optimised structures of the iridium tetrahydride complex/cation ion pair before the arrival of the ketone molecule.

The cations are placed in less crowded space opposite to the ((*S*)-P,S^Ph^) ligand and have a well-organised solvation sphere, involving the five MeOH molecules plus interactions with the hydride ligands of the Ir^III^ anion. The M–O distances (M = Li, Na, K) involving the five solvent molecules are 2.03–2.16 Å for Li^+^, 2.32–2.40 Å for Na^+^ and 2.70–2.82 Å for K^+^. These distances are close to the sum of the covalent radii of the metals and oxygen, particularly for Na^+^ and K^+^ (1.94, 2.32 and 2.69 Å, for Li, Na and K, respectively).^44^ The additional alkali metal-hydride interactions show a different behaviour for Li^+^*vs.* the other two cations. For Na^+^ and K^+^, one hydride ligand completes a distorted octahedral solvation sphere, thus bridging the alkali-metal cation close to the Ir^III^ centre (Na–H: 2.40 Å; K–H: 2.79 Å), while the hydride ligand is out of the first solvation sphere of Li^+^ (Li–H: 2.59 Å), presumably due to its smaller ionic radius and preference for lower coordination numbers. Usually, the promoting effect of alkali cations in hydrogenation reactions involving carbonyl groups have been discussed from DFT calculations in terms of activation of the carbonyl moiety by a direct M^+^⋯O=C interaction.^29,30^ However, those calculations did not include the solvation sphere of the cations, which leads to non-physical models. Our results highlight that, in order for a direct M^+^⋯O=C interaction to result in an alcohol solvent, at least one of the solvent molecules needs to be displaced from the cation solvation sphere.

**Fig. 3:**
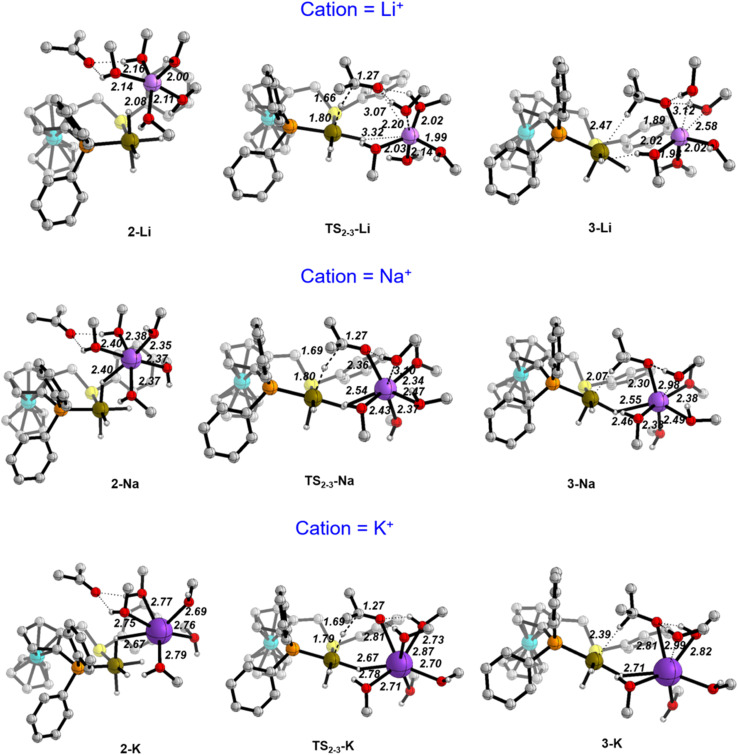
Optimised structures along the hydride transfer step for the three cations in the (MeOH)_5_ solvent model. Distances in Å. C–H hydrogen atoms have been omitted for clarity.

Fig. 3 depicts the optimised structures along the hydride transfer step for the three cations: the initial intermediate in which the acetone has been incorporated (2), the transition state for the outer-sphere hydride transfer (TS_2-3_) and the generated σ-complex where the alkoxide that is produced by hydride transfer interacts with the Ir^III^ centre through the formed C–H bond (3). In all of the structures, two solvent molecules are H-bonded to the acetone oxygen atom but, notably, the acetone molecule is itself not incorporated in the coordination sphere of the alkali metal cations. These two H-bonds strengthen as the negative charge on the oxygen atom increases. For instance, in the K^+^ system the O–H distances are 2.01/1.89 Å in 2, 1.80/1.90 Å in TS_2-3_ and 1.61/1.63 Å in 3. Similar changes were found for the Li^+^ and Na^+^ systems.

Values of the main distances involved in TS_2-3_ (Ir–H, H–C and C–O), collected in Fig. 3, show almost no difference between the three systems. Indeed, they are also very similar to those obtained without cation, just keeping the two solvating methanol molecules, for which the computed hydride transfer barrier is 14.6 kcal mol^−1^ (see Fig. S4†). The same situation is evident with atomic charges, as computed with the Charge Model 5 (CM5) (Table 2).^45^ Regarding the acetone substrate polarisation in TS_2-3_, the charge transferred to the acetone oxygen atom is about the same in the Li^+^ and cation-free cases, whereas it is slightly greater for the Na^+^ and K^+^ systems (Table 2).

**Table 2:** Main distances (Å) and CM5 atomic charges in the transition states of the hydride transfer (TS_2-3_). Values in parentheses have been obtained using isopropanol in the hybrid continuum-explicit (five solvent molecules) solvent model

	Li^+^	Na^+^	K^+^	No cation
**Distances**				
Ir–H	1.80 (1.76)	1.80 (1.78)	1.79 (1.78)	1.78 (1.78)
H–C	1.66 (1.80)	1.69 (1.73)	1.69 (1.72)	1.68 (1.68)
C–O	1.27(1.27)	1.27 (1.27)	1.27 (1.26)	1.27 (1.26)

**Charges**				
O	−0.38 (−0.39)	−0.45 (−0.41)	−0.43 (−0.43)	−0.39 (−0.37)
C	0.15 (0.18)	0.15 (0.17)	0.15 (0.16)	0.15 (0.15)
H	−0.07 (−0.08)	−0.07 (−0.07)	−0.07 (−0.07)	−0.08 (−0.07)
Ir	−0.12 (−0.14)	−0.12 (−0.13)	−0.12 (−0.12)	−0.20 (−0.18)
CH_3_CO	−0.19 (−0.10)	−0.24 (−0.16)	−0.20 (−0.29)	−0.19 (−0.19)
M	0.47 (0.46)	0.63 (0.63)	0.77 (0.77)	—

The three TS_2-3_ transition states have markedly different barriers (Fig. 1), even though they exhibit very similar geometric and charge parameters for iridium coordination of the acetone substrate. However, the cation solvation sphere changes significantly along the hydride transfer pathway, suggesting that solvation rearrangement is the main cause of the difference in barrier. Alkoxide coordination to the alkali cation stabilises the negative charge that develops during the hydride transfer step, this disrupts the solvation sphere of the cation, leading to different barriers depending on the cation present. Once the alkoxide has formed (3), it coordinates to the alkali-metal cation.

In the Li^+^ system, two methanol molecules are displaced to produce the tetrahedral solvation sphere of this cation in 3-Li, formed by the alkoxide and three solvent molecules. The lithium cation does not interact with any hydride ligand throughout this process. In contrast, for the Na^+^ and K^+^ systems, one methanol molecule is displaced by alkoxide, thereby maintaining a distorted octahedral solvation sphere for the alkali-metal cation in 3-Na and 3-K, which is made up by the alkoxide, four solvent molecules and a bridging hydride ligand. This change in solvation is more favourable for K^+^, the cation with the largest ionic radius and greater effective charge (Table 2). Overall, the calculations using the (MeOH)_5_ solvent model reproduce correctly the experimentally observed cation effect on the hydrogenation rate and allow its rationalisation, stressing the importance of the changes in the solvation sphere of the alkali cation along the reaction. However, to assess the validity of this model and to get more reliable results, we have reoptimized all the structures involved in the hydride transfer step (1-M, 2-M, TS_23_-M, 3-M and 4-M) in the experimentally employed isopropanol solvent, described with the cluster-continuum solvent model.

The obtained relative Gibbs energies are compared to those in methanol in Table 3. The main geometrical parameters and charges of the hydride-transfer transition states are compared in Table 2. 3D-structures of all the species can be found in the ESI (Fig. S10–S14).† The main conclusion from these calculations using the solvent used experimentally is that they give essentially very similar results to those in methanol. The trend in the barriers for the hydride transfer step (K < Na < Li) is preserved, with very close barriers. We have also computed the barriers for the hydride-transfer step to acetophenone leading to the lowest *S* enantiomer with the three cations and (^i^PrOH)_5_. The obtained values (K^+^: 9.9 kcal mol^−1^; Na^+^: 12.5 kcal mol^−1^; Li^+^: 14.4 kcal mol^−1^) are very similar to those obtained with acetone (Table 3). However, while with acetone there is no stabilization of the initial intermediates 2-M and the barriers are computed with respect to the separated species (1-M + ketone), with acetophenone the initial intermediates are below the separated species and the energy span is computed as a difference between 2-M and TS_2-3_-M. The absolute barriers are not consistent with the activities seen experimentally (non-quantitative yields in 4 hours). There are also concentration effects at play when considering rates, that would be difficult to address in the calculated barriers.

**Table 3:** Comparison of relative Gibbs energies (kcal mol^−1^) of the species involved in the hydride transfer step with methanol and isopropanol solvents. Cluster-continuum solvent model with (ROH)_5_ explicit solvent molecules + SMD continuum

	K^+^		Na^+^		Li^+^		No cation		TMA^+^
	Me(OH)	^i^Pr(OH)	Me(OH)	^i^Pr(OH)	Me(OH)	^i^Pr(OH)	Me(OH)	^i^Pr(OH)	^i^Pr(OH)
1-M + ketone	0.0	0.0	0.0	0.0	0.0	0.0	0.0	0.0	0.0
2-M	0.4	0.9	4.5	4.7	0.6	6.6	8.1	6.7	8.0
TS_23_-M^a^	**9.8**	**9.9**	**14.1**	**13.2**	**16.6**	**14.9**	**14.6**	**16.9**	**17.2**
3-M	2.2	2.3	7.9	4.5	2.1	1.3	6.3	9.0	6.2
4-M	−4.7	−9.3	−0.3	−5.5	0.0	−1.4	0.1	1.5	3.8

a These values give the energy span of the catalytic cycle.

The most noticeable change when moving from the methanol to the isopropanol solvent model is a higher stabilisation of the alkoxide intermediates 4-M in isopropanol solvent. Isopropanol has a significant effect on the stability of the alkoxide intermediate 4, and this may then have an impact on the barriers to the subsequent steps. To address this issue, we have first computed the barrier for the 3 to 4 rearrangement for the three cations and the (^i^PrOH)_5_ model, to discard it as rate-determining step, and then we have recomputed the full energy profile with the potassium cation and the isopropanol model.

The 3 to 4 step involves a change in the coordination mode of the alkoxide formed in the previous step, from a weak C–H⋯Ir interaction to a stronger Ir–O bond. In this rearrangement solvation stabilizes the anionic alkoxide when it has been released from the iridium coordination sphere and the barriers are low, well below those of the hydride transfer for the three cations. However, they display a cation dependence (K: 1.3, Na: 3.0, Li: 6.5 kcal mol^−1^) that follows that of the stability of the O-bound alkoxide (structures 4-M; K: −9.3, Na: −5.5, Li: −1.4 kcal mol^−1^). See TS_3-4_-M-iso in Fig. S10–S12.†

**Fig. 4:**
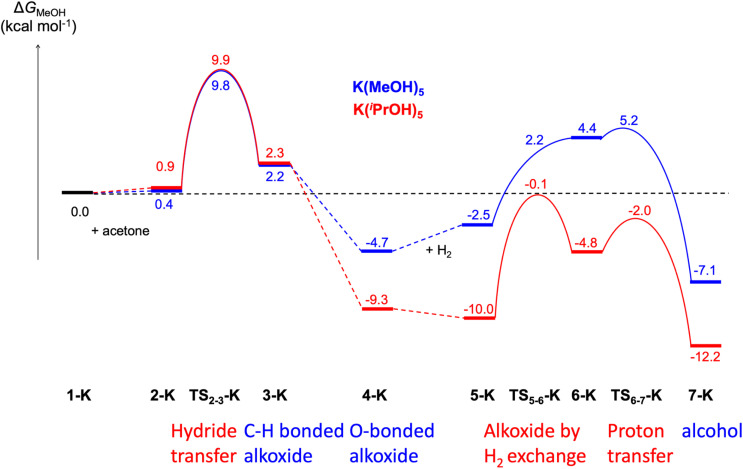
Comparison of the computed Gibbs energy profiles for ketone hydrogenation catalysed by [K(solvent)_5_]^+^[Ir(H)_4_((*S*)-P,S^Ph^)]^−^ with 5 MeOH or 5 ^i^PrOH molecules explicitly included in the calculations.

Regarding the complete profile with potassium cation in the isopropanol solvent model, comparing with the same profile with methanol, a strong stabilization of all the structures involving an alkoxide is observed (from 4-K). Isopropanol solvent stabilises the alkoxide better than methanol. However, the barriers remain unchanged: 9.9 kcal mol^−1^ for the hydride transfer step and 9.9 kcal mol^−1^ for the second part of the reaction (Fig. 4, optimized structures in Fig. S12†). Overall, these results substantiate the choice of acetone as a model to validate the catalytic mechanism.

With the isopropanol solvent model, the highest barrier is found in the cation-free system. This barrier displays consistent values of 16.9 kcal mol^−1^ when keeping two solvating isopropanol molecules, 17.1 kcal mol^−1^ with three solvent molecules and 17.6 kcal mol^−1^ when isopropanol is only modelled as a continuum (no explicit solvent).

**Fig. 5:**
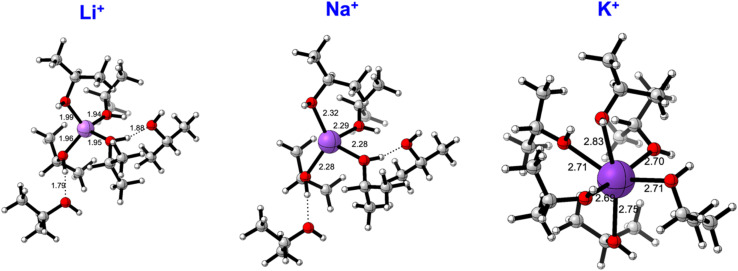
Favoured solvation spheres for [M(^i^PrOH)_6_]^+^ systems. Distances in Å.

From our analysis, the solvation sphere of the cation plays an important role. We have always found ordered and strongly oriented first solvation shells around the spherical alkali cations, with oxygen atoms pointing toward the cation. We have further analysed this issue by determining the preferred conformation of lithium, sodium and potassium solvated cations in the presence of six solvent (isopropanol) molecules, in isopropanol (continuum). Fig. 5 displays these structures. Lithium shows a clear preference to accommodate four isopropanol molecules in the first solvation sphere. In presence of 6 isopropanol molecules a 4 + 2 conformation, with four solvent molecules in the first solvation sphere in a tetrahedral-like arrangement is 5.3 kcal mol^−1^ more stable than a 5 + 1 and 7.7 kcal mol^−1^ than having the 6 isopropanol molecules interacting with the cation. The situation is reversed for potassium, for which an octahedral-like coordination of six alcohols molecules is favoured over the 5 + 1 and 4 + 2 configurations (3.1 and 2.7 kcal mol^−1^ above). An intermediate situation is found for sodium. The 4 + 2 is the preferred conformation, but six-coordination is only 1.6 kcal mol^−1^ less favourable. Both the favoured coordination numbers and the optimised M–O distances agree with experimental studies and molecular dynamics simulations studying structural characteristics of the solvation environment of alkali metal ions in various electron-donor oxygen containing solvents, with methanol among them.^46,47^

**Fig. 6:**
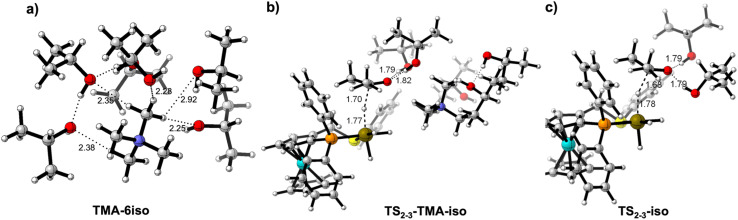
(a) Solvation sphere for [(TMA)(^i^PrOH)_6_]^+^ system. Transition states for the hydride transfer in the [(TMA)(^i^PrOH)_5_]^+^ (b) and cation free (c) systems. Distances in Å.

These results raise the question of the influence in the ketone hydrogenation of a cation like tetramethylammonium (TMA). TMA is a large cation with a relatively low charge density that has been described as one of the simplest and most spherical representatives of the so-called ‘‘hydrophobic ions’’ and for which minor polarization effects can be expected.^48^ A loose solvation can be expected for TMA, and indeed that is what we have found when optimising a [(TMA)(^i^PrOH)_6_]^+^ cluster in isopropanol (Fig. 6a).

From this solvation scenario, a behaviour similar to the cation-free system can be foreseen in the ketone hydrogenation reaction. We have computed the hydride transfer step in isopropanol with a [(TMA)(^i^PrOH)]_5_^+^ cluster. The results, collected in Table 3, agree with this similarity with the cation-free case (barriers of 16.9 and 17.2 kcal mol^−1^ for the cation-free and TMA systems, respectively). Indeed, this result was found in the experimental study using Me_4_N^+^ instead of an alkali-metal cation (Table 1, entry 6). Comparison of both transition states (TS_23_-iso and TS_23_-TMA, Fig. 6) highlight the structural likeness of the transition states, in which the incipient alkoxide is interacting with only two solvent molecules with no influence of the cation.

### The importance of a proper solvent model

As commented above, the hydride transfer goes along with a reorganisation of the cation solvation sphere. It can be expected that an appropriate description of the first solvation shell of the cation is a requirement for a reliable description of the process. Indeed, in the initial solvent model we only included three methanol molecules, as we did in a previous study of this reaction mechanism.^9^ We found, however, that this model was not able to reproduce the experimental activity trend (see Fig. S5†). We will only comment on the main features of this inaccurate model. First, the order of the Gibbs energy barriers for the hydride transfer step is reversed: Li^+^ (9.7 kcal mol^−1^) < Na^+^ (13.1 kcal mol^−1^) < K^+^ (13.6 kcal mol^−1^). Secondly, the alkoxide/H_2_ exchange (TS_5-6_) is associated with remarkably higher barriers, in such a way that the cycle energy span (Li^+^: 11.6 kcal mol^−1^; Na^+^: 18.5 kcal mol^−1^; K^+^: 20.6 kcal mol^−1^) is determined by the relative energy of this ligand exchange transition state and 4-M, rather than by the hydride transfer step.

**Fig. 7:**
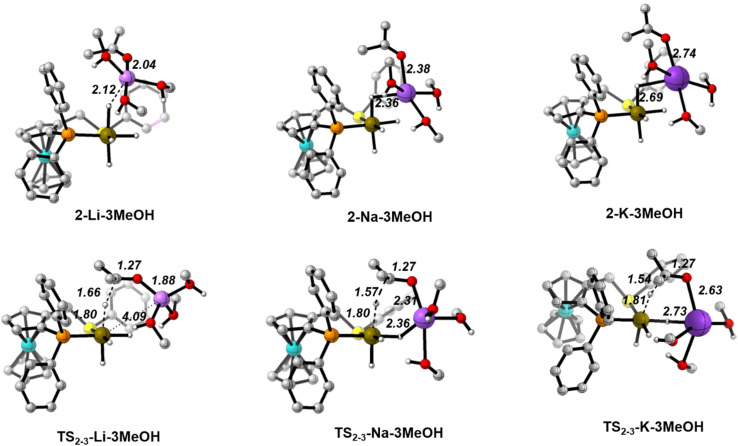
Optimised structures along the hydride transfer step for the three cations with the [M(MeOH)_3_]^+^ solvent model. Distances in Å. C–H hydrogen atoms have been omitted for clarity.

Fig. 7 collects the optimised structures of the 2-M-3MeOH intermediates and TS_2-3_-M-3MeOH transition states. With this model, the carbonyl substrate is already in contact with the cation in 2, hence there is no need to displace a solvent molecule from the cation during the hydride transfer process. Moreover, the Na^+^ and K^+^ cations cannot reach their optimal distorted octahedral solvation shell in any of the states.

**Fig. 8:**
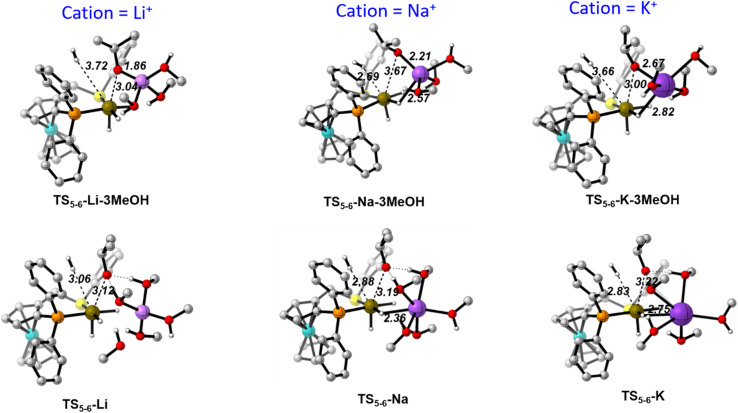
Transition states of the alkoxide by H_2_ ligand exchange with the [M(MeOH)_3_]^+^ (top) and [M(MeOH)_5_]^+^ (bottom) solvent models. Distances in Å. C–H hydrogen atoms have been omitted for clarity.

After formation of the alkoxide intermediate 4, the next step involves the exchange of a strongly bound, negatively charged alkoxide ligand by a neutral, weakly donating H_2_ molecule. It is apparent that, without the help of solvent molecules to stabilise the departing alkoxide ligand *via* H-bonding, ligand exchange will not be favoured. Fig. 8 compares the transition states of the alkoxide/H_2_ exchange (TS_5-6_), optimised with the [M(MeOH)_3_]^+^ and [M(MeOH)_5_]^+^ solvent models, for the three cations. The stabilizing H-bond interaction between two M^+^-coordinated MeOH molecules and the leaving alkoxide can be appreciated in all TS_5-6_-M structures, while it is absent in all TS_5-6_-M-3MeOH structures. Thanks to the assistance by these interactions, the barriers for this step are lowered to 5.4 (Li^+^), 9.8 (Na^+^) and 6.9 (K^+^) kcal mol^−1^. The corresponding barriers for the 3MeOH model are 11.6 (Li^+^), 13.7 (Na^+^) and 14.1 (K^+^) kcal mol^−1^.

### DFT investigations of the cation effect on enantioselectivity and limitations of the solvent model

**Fig. 9:**
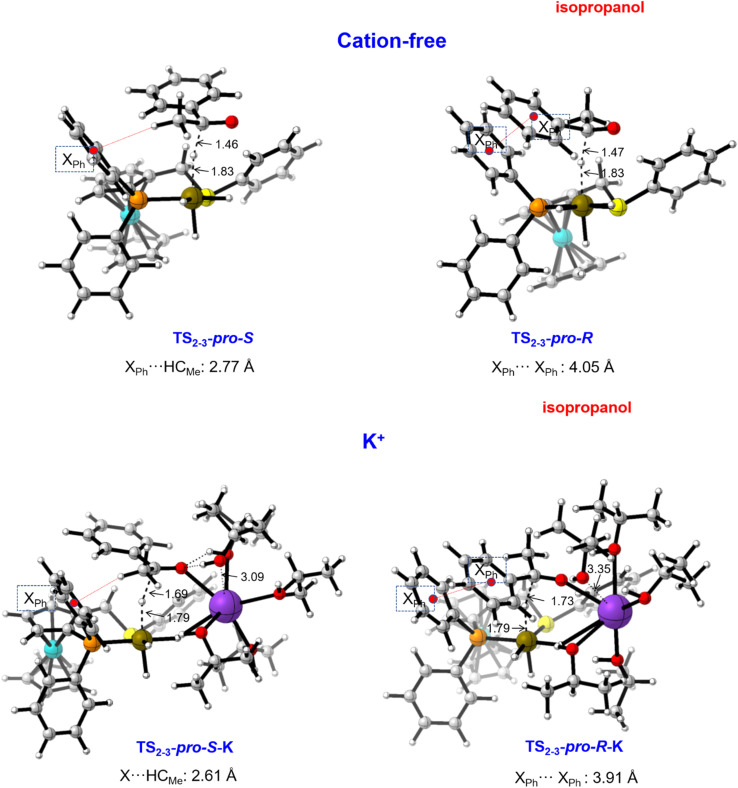
*pro-S* and *pro-R* transition states for hydride transfer to acetophenone, in absence of the cation and with [K(^i^PrOH)_5_]^+^. Distances in Å.

Contrarily to the strong alkali-metal cation effect on the catalytic activity, its influence on enantioselectivity is small (Table 1). To understand the origin of this behaviour, we have performed DFT calculations on the enantio-determining hydride-transfer step using the real prochiral acetophenone substrate while employing the ((*S*)-P,S^Ph^) ligand. Preliminary results revealed that calculations dealing with enantioselectivity required a description of the system that is as realistic as possible. For this reason, they were performed using an isopropanol solvent model, with the usual cluster-explicit approach. For this system, the *S* enantiomer of the ligand leads to a formation preference for the *S* enantiomer of the 1-phenylethanol product with all three alkali-metal cations yielding essentially the same ee. The *pro-R* and *pro-S* transition states TS_2-3_ were computed for the three [M-(^i^PrOH)_5_]^+^ cations, as well as for the cation-free catalyst without explicit solvent molecules for comparative purposes. Fig. 9 shows the resulting TS geometries, and important distances for the cation-free (top) and K (bottom) systems. Similar outputs result for the other two cations (see Fig. S15†). The main interaction that discriminates between the two diastereomeric transition states involves a phenyl group of the phosphine ligand. This group interacts with the acetophenone methyl group (C–H–π interaction) in the *pro-S* TS and with the acetophenone phenyl group (π–π interaction) in the *pro-R* TS. The cation is located in the less crowded space opposite to this key *P*-phenyl group. Consequently, the nature of the cation does not significantly alter the main interactions that govern the ee. Table 4 gathers the calculated X_Ph_⋯HC_Me_ and X_Ph_··· X_Ph_ distances for all computed systems, together with the Gibbs energy difference between both transition states 
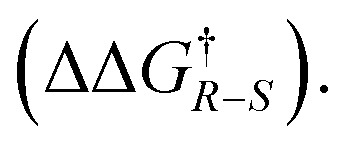
 The geometrical parameters describing the substrate–ligand interaction are very similar in all cases, in agreement with the limited influence the cation plays on enantioselectivity.

**Table 4:** Main distances (Å), Gibbs energy differences (kcal mol^−1^) and computed enantiomeric excesses between the *pro-S* and *pro-R* transition states for hydride transfer to acetophenone with the [M(^i^PrOH)_5_]^+^ model

	X_Ph_⋯HC_Me_	X_Ph_··· X_Ph_	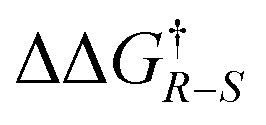 a	ee^a^
Cation free	2.767	4.049	3.3 (3.7)	99 (100)
Li^+^	2.516	3.850	3.8 (2.7)	100 (98)
Na^+^	2.534	3.867	3.1 (2.1)	99 (94)
K^+^	2.615	3.918	1.2 (1.7)	77 (89)

a B3LYP-D3/BS2 values in parentheses.

In each case, the *pro-S* transition state is lower than that of *pro-R*, in qualitative agreement with the experimental results. However, the computed 
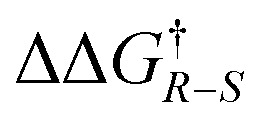
 overestimate these differences, as comparison of experimental and computed ees showcases.^49^ We have recomputed the ΔΔ*G*_*R*–*S*_ with the B3LYP-D3 functional at the fixed M06-optimised geometries, with very similar outcome (Table 4). The calculations correctly predict that the *S* isomer is preferred and rationalizes the little influence of the cation nature on the ees as a consequence of the cation placement, but also reveal the difficulties in quantitatively reproducing the experimental ees in such a complex system. Several factors can be invoked to justify this discrepancy. On one hand, the energies are very sensitive to the position of isopropanol molecules and slightly different arrangements of the five solvent molecules appreciably modifies the value of 
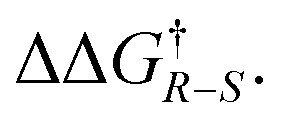
 On the other hand, the balance between the relative strength of the C–H–π and π–π interactions may be functional-dependent. Finally, our model of five solvent molecules satisfies the coordination requirements of the alkali cation but does not satisfy the van der Waals requirements of the substrate. The ΔΔ*G*^†^ reflects the different TS stabilization when docking the substrate with the two different enantiofaces, but this could be overestimated by the lack of explicit van der Waals interactions between the substrate and the solvent. The stronger interaction in the TS leading to the preferred product enantiomer may be somewhat buffered by stronger interactions with the solvent as well. To prove that, a much more expensive full explicit solvent model would be needed.

## Conclusions

The role of anionic hydride complexes as active catalysts in hydrogenation and hydrogen-transfer reactions is now becoming well-established. When such species operate in solvents such as ^i^PrOH, the cation may associate with the anion in addition to coordination of the ion by solvent molecules. The present contribution has illustrated one such case for the active catalyst produced *in situ* by activation of [IrCl(COD)((*S*)-P,S^R^)] under H_2_ in the presence of a strong base. The stark dependence of the catalytic activity on the nature of the base alkali cation (K^+^ > Na^+^ > Li^+^) and the severe activity dampening observed when an 18-crown-6 is added to the K^+^ system confirm our previous suggestion of an anionic active complex, with ion pairing between the anion and cation.^9^ The proposition, made solely on the basis of DFT calculations in the same contribution,^9^ that such a species is the Ir^III^ tetrahydrido complex M^+^[Ir(H)_4_((*S*)-P,S^R^)], has recently found strong support by the observed formation of the related (and more stable) K^+^[Ir(H)_4_(dppe)]^−^ from [IrCl(COD)(dppe)] in isopropanol and KO^*t*^Bu under hydrogenation conditions (H_2_ gas at 25 °C) and even under transfer hydrogenation conditions (without H_2_ at 80 °C).^14^ This hypothesis is further supported by the DFT calculations carried out in the present contribution, where the experimental trend of alkali metal cation-dependent activity has not only been reproduced but also rationalised, at the condition of using a fully explicit cation coordination sphere, M(ROH)_5_^+^. Most intriguingly, the activity does not appear to depend on any activating effect by the cation on either the ketone substrate or the iridium hydride species, but rather on the coordination sphere rearrangement at the alkali metal cation when, in the rate-determining transition state, the incipient alkoxide is formed and concurrently establishes a bond with the alkali metal cation, replacing one or more alcohol molecules in its coordination sphere. These results indicate that the inclusion of explicit solvent molecules (and a sufficient number of them) is of paramount importance for the appropriate interpretation of certain effects that may be observed in catalytic cycles that involve anionic active species.

The calculations reported here were also able to pinpoint the main enantio-discriminating interaction in the rate-determining transition state, rationalizing the relatively low effect of the alkali metal nature on the ee and further supporting the proposed catalyst structure. However, they are not able to quantitative reproduce the experimental ees, pointing out the need of a full explicit solvent model, involving very expensive DFT-based Molecular Dynamics simulations with a solvent box, for a more quantitative agreement. Despite this shortcoming, static DFT calculations with a careful choice of the solvent model can reproduce the experimental trends and identify its origin. On the basis of the work presented in this contribution, the DFT tool may now be applied with confidence to further explorations of the ligand space in order to achieve improved activities and enantioselectivities for any prochiral substrate of interest.

## Data availability

The experimental and computational data, including *xyz* coordinates for all states, have been included as part of the ESI† and FAIR data have been deposited at https://hal.science/hal-04777200.

## Author contributions

PK: investigation and analysis. SV: investigation and analysis. EM: supervision, review and editing. JML: supervision, review and editing. JMS: supervision, review and editing. SBD: supervision, review and editing. AL: conceptualization, investigation, analysis, writing, review and editing. RP: conceptualization, funding acquisition, writing, review and editing.

## Conflicts of interest

There are no conflicts to declare.

## Supplementary Material

SC-OLF-D4SC04629C-s001

## References

[cit1] Tang W., Zhang X. (2003). New Chiral Phosphorus Ligands for Enantioselective Hydrogenation. Chem. Rev..

[cit2] Xie J. H., Zhu S. F., Zhou Q. L. (2011). Transition Metal-Catalyzed Enantioselective Hydrogenation of Enamines and Imines. Chem. Rev..

[cit3] Blaser H.-U. , Pugin B. and Spindler F., in Organometallics as Catalysts in the Fine Chemical Industry, ed. M. Beller and H. U. Blaser, 2012, vol. 42, pp. 65–102

[cit4] Seo C. S. G., Morris R. H. (2019). Catalytic Homogeneous Asymmetric Hydrogenation: Successes and Opportunities. Organometallics.

[cit5] Wang D.-S., Chen Q.-A., Lu S.-M., Zhou Y.-G. (2012). Asymmetric Hydrogenation of Heteroarenes and Arenes. Chem. Rev..

[cit6] Wen J. L., Wang F. Y., Zhang X. M. (2021). Asymmetric hydrogenation catalyzed by first-row transition metal complexes. Chem. Soc. Rev..

[cit7] Sandoval C. A., Ohkuma T., Muniz K., Noyori R. (2003). Mechanism of asymmetric hydrogenation of ketones catalyzed by BINAP/1,2-diamine-ruthenium(II) complexes. J. Am. Chem. Soc..

[cit8] Yamakawa M., Ito H., Noyori R. (2000). The metal-ligand bifunctional catalysis: A theoretical study on the ruthenium(II)-catalyzed hydrogen transfer between alcohols and carbonyl compounds. J. Am. Chem. Soc..

[cit9] Hayes J. M., Deydier E., Ujaque G., Lledós A., Malacea-Kabbara R., Manoury E., Vincendeau S., Poli R. (2015). Ketone Hydrogenation with Iridium Complexes with “non N–H” Ligands: The Key Role of the Strong Base. ACS Catal..

[cit10] Routaboul L., Vincendeau S., Daran J.-C., Manoury E. (2005). New ferrocenyl P,S and S,S ligands for asymmetric catalysis. Tetrahedron: Asymmetry.

[cit11] Malacea R., Daran J.-C., Duckett S. B., Dunne J. P., Godard C., Manoury E., Poli R., Whitwood A. C. (2006). Parahydrogen studies of H_2_ addition to an Ir(I) complex containing chiral phosphine- thioether ligands: implications for catalysis. Dalton Trans..

[cit12] Malacea R., Manoury E., Routaboul L., Daran J.-C., Poli R., Dunne J. P., Withwood A. C., Godard C., Duckett S. B. (2006). Coordination chemistry and diphenylacetylene hydrogenation catalysis of planar chiral ferrocenylphosphine-thioether ligands with cyclooctadieneiridium(I). Eur. J. Inorg. Chem..

[cit13] Le Roux E., Malacea R., Manoury E., Poli R., Gonsalvi L., Peruzzini M. (2007). Highly efficient asymmetric hydrogenation of alkyl aryl ketones catalyzed by iridium complexes with chiral planar ferrocenyl phosphino-thioether ligands. Adv. Synth. Catal..

[cit14] Kisten P., Manoury E., Lledós A., Whitwood A. C., Lynam J., Slattery J., Duckett S. B., Poli R. (2023). Ir^I^(η^4^-diene) precatalyst activation by strong bases: formation of an anionic Ir^III^ tetrahydride. Dalton.

[cit15] Abdur-Rashid K., Gusev D. G., Landau S. E., Lough A. J., Morris R. H. (1998). Organizing chain structures by use of proton-hydride bonding. The single-crystal X-ray diffraction structures of K(Q) Os(H)(5)((PPr3)-Pr-*i*)(2) and K(Q) Ir(H)(4)((PPr3)-Pr-*i*)(2) , Q = 18-crown-6 and 1,10-diaza-18-crown-6. J. Am. Chem. Soc..

[cit16] Landau S. E., Groh K. E., Lough A. J., Morris R. H. (2002). Large effects of ion pairing and protonic-hydridic bonding on the stereochemistry and basicity of crown- ; azacrown- ; and cryptand-222-potassium salts of anionic tetrahydride complexes of iridium(III). Inorg. Chem..

[cit17] Grey R. A., Pez G. P., Wallo A. (1980). Selective Homogeneous Catalytic Hydrogenation of Polynuclear Aromatics. J. Am. Chem. Soc..

[cit18] Grey R. A., Pez G. P., Wallo A., Corsi J. (1980). Homogeneous Catalytic Hydrogenation of Carboxylic Acid Esters to Alcohols. Chem. Commun..

[cit19] Grey R. A., Pez G. P., Wallo A. (1981). Anionic Metal Hydride Catalysts. 2. Application to the Hydrogenation of Ketones, Aldehydes, Carboxylic Acid Esters, and Nitriles. J. Am. Chem. Soc..

[cit20] Pez G. P., Grey R. A., Corsi J. (1981). Anionic Metal Hydride Catalysts. 1. Synthesis of Potassium Hydrido(phosphine)ruthenate Complexes. J. Am. Chem. Soc..

[cit21] Grey R. A., Pez G. P., Wallo A., Corsi J. (1983). The hydrogention of carbonyl compounds catalyzed by anionic ruthenium hydride complexes a,d alkali-doped supported group-VIII metals. Ann. N. Y. Acad. Sci..

[cit22] Wilczynski R., Fordyce W. A., Halpern J. (1983). Coordination Chemistry and Catalytic Properties of Hydrido(phosphine)ruthenate Complexes. J. Am. Chem. Soc..

[cit23] Fordyce W. A., Wilczynski R., Halpern J. (1985). Hydrido(phosphine)ruthenate complexes and their role in the catalytic hydrogenation of arenes. J. Organomet. Chem..

[cit24] Linn D. E., Halpern J. (1987). Roles of Neutral and Anionic Ruthenium Polyhydrides in the Catalytic Hydrogenation of Ketones and Arenes. J. Am. Chem. Soc..

[cit25] Dub P. A., Henson N. J., Martin R. L., Gordon J. C. (2014). Unravelling the Mechanism of the Asymmetric Hydrogenation of Acetophenone by [RuX2(diphosphine)(1,2-diamine)] Catalysts. J. Am. Chem. Soc..

[cit26] Nielsen M., Alberico E., Baumann W., Drexler H. J., Junge H., Gladiali S., Beller M. (2013). Low-temperature aqueous-phase methanol dehydrogenation to hydrogen and carbon dioxide. Nature.

[cit27] Alberico E., Lennox A. J. J., Vogt L. K., Jiao H., Baumann W., Drexler H.-J., Nielsen M., Spannenberg A., Checinski M. P., Junge H., Beller M. (2016). Unravelling the Mechanism of Basic Aqueous Methanol Dehydrogenation Catalyzed by Ru-PNP Pincer Complexes. J. Am. Chem. Soc..

[cit28] van Putten R., Uslamin E. A., Garbe M., Liu C., Gonzalez-de-Castro A., Lutz M., Junge K., Hensen E. J. M., Beller M., Lefort L., Pidko E. A. (2017). Non-Pincer-Type Manganese Complexes as Efficient Catalysts for the Hydrogenation of Esters. Angew. Chem., Int. Ed..

[cit29] Liu C., van Putten R., Kulyaev P. O., Filonenko G. A., Pidko E. A. (2018). Computational insights into the catalytic role of the base promoters in ester hydrogenation with homogeneous non-pincer-based Mn-P,N catalyst. J. Catal..

[cit30] Liang Z. Q., Yang T. L., Gu G. X., Dang L., Zhang X. M. (2018). Scope and Mechanism on Iridium-f-Amphamide Catalyzed Asymmetric Hydrogenation of Ketones. Chin. J. Chem..

[cit31] Yin C., Jiang Y.-F., Huang F., Xu C.-Q., Pan Y., Gao S., Chen G.-Q., Ding X., Bai S.-T., Lang Q., Li J., Zhang X. (2023). A 13-million turnover-number anionic Ir-catalyst for a selective industrial route to chiral nicotine. Nat. Commun..

[cit32] Västilä P., Zaitsev A. B., Wettergren J., Privalov T., Adolfsson H. (2006). The importance of alkali cations in the {RuCl2(p-cymene)}(2)-pseudodipeptide-catalyzed enantioselective transfer hydrogenation of ketones. Chem.–Eur. J..

[cit33] Slagbrand T., Kivijarvi T., Adolfsson H. (2015). Bimetallic Catalysis: Asymmetric Transfer Hydrogenation of Sterically Hindered Ketones Catalyzed by Ruthenium and Potassium. ChemCatChem.

[cit34] Bielinski E. A., Lagaditis P. O., Zhang Y. Y., Mercado B. Q., Wurtele C., Bernskoetter W. H., Hazari N., Schneider S. (2014). Lewis Acid-Assisted Formic Acid Dehydrogenation Using a Pincer-Supported Iron Catalyst. J. Am. Chem. Soc..

[cit35] Bielinski E. A., Forster M., Zhang Y., Bernskoetter W. H., Hazari N., Holthausen M. C. (2015). Base-Free Methanol Dehydrogenation Using a Pincer-Supported Iron Compound and Lewis Acid Co-catalyst. ACS Catal..

[cit36] Zhang Y., MacIntosh A. D., Wong J. L., Bielinski E. A., Williard P. G., Mercado B. Q., Hazari N., Bernskoetter W. H. (2015). Iron catalyzed CO2 hydrogenation to formate enhanced by Lewis acid co-catalysts. Chem. Sci..

[cit37] Govindarajan N., Meijer E. J. (2019). Elucidating cation effects in homogeneously catalyzed formic acid dehydrogenation. Faraday Discuss..

[cit38] Gawron M., Gilch F., Schmidhuber D., Kelly J. A., Horsley Downie T. M., Jacobi von Wangelin A., Rehbein J., Wolf R. (2024). Counterion Effect in Cobaltate-Catalyzed Alkene Hydrogenation. Angew. Chem., Int. Ed..

[cit39] Jia M., Bandini M. (2015). Counterion Effects in Homogeneous Gold Catalysis. ACS Catal..

[cit40] Epton R. G., Unsworth W. P., Lynam J. M. (2023). DFT Studies of Au(I) Catalysed Reactions: Anion Effects and Reaction Selectivity. Isr. J. Chem..

[cit41] Zaccaria F. , Sian L., Zuccaccia C. and Macchioni A., in Advances in Organometallic Chemistry, ed. P. J. Pérez, Academic Press, 2020, vol. 73, pp. 1–78

[cit42] Phipps R. J., Hamilton G. L., Toste F. D. (2012). The progression of chiral anions from concepts to applications in asymmetric catalysis. Nat. Chem..

[cit43] Mahlau M., List B. (2013). Asymmetric Counteranion-Directed Catalysis: Concept, Definition, and Applications. Angew. Chem., Int. Ed..

[cit44] Cordero B., Gomez V., Platero-Prats A. E., Reves M., Echeverria J., Cremades E., Barragan F., Alvarez S. (2008). Covalent radii revisited. Dalton Trans..

[cit45] Marenich A. V., Jerome S. V., Cramer C. J., Truhlar D. G. (2012). Charge Model 5: An Extension of Hirshfeld Population Analysis for the Accurate Description of Molecular Interactions in Gaseous and Condensed Phases. J. Chem. Theory Comput..

[cit46] Smirnov P. R. (2019). Structure of the Li+ Ion Close Environment in Various Solvents. Russ. J. Gen. Chem..

[cit47] Smirnov P. R. (2020). Structure of the Nearest Environment of Na^+^, K^+^, Rb^+^, and Cs^+^ Ions in Oxygen-Containing Solvents. Russ. J. Gen. Chem..

[cit48] Mason P. E., Martinek T., Fábián B., Vazdar M., Jungwirth P., Tichacek O., Duboué-Dijon E., Martinez-Seara H. (2024). Hydration of biologically relevant tetramethylammonium cation by neutron scattering and molecular dynamics. Phys. Chem. Chem. Phys..

[cit49] Wahlers J., Margalef J., Hansen E., Bayesteh A., Helquist P., Diéguez M., Pàmies O., Wiest O., Norrby P.-O. (2021). Proofreading experimentally assigned stereochemistry through Q2MM predictions in Pd-catalyzed allylic aminations. Nat. Commun..

