# Supplementary material for: Understanding ketone hydrogenation catalysis with anionic iridium(iii) complexes: the crucial role of counterion and solvation
Source: Chem Sci. 2024 Nov 11;15(48):20478–92. doi: 10.1039/d4sc04629c (PMC11583429; doi:10.1039/d4sc04629c)
Supplement: SC-015-D4SC04629C-s001 [file SC-015-D4SC04629C-s001.pdf]

Supplementary Information (SI) for Chemical Science.
This journal is © The Royal Society of Chemistry 2024

Understanding ketone hydrogenation catalysis with anionic iridium(III) complexes: The crucial role of counterion and solvation

Paven Kisten,[a,b] Eric Manoury,[a] Sandrine Vincendeau,[a] Jason Lynam,[b] John Slattery,*[b] Simon B. Duckett,[b] Agustí Lledós*[c] and Rinaldo Poli*[a,d]

[a]*CNRS, LCC (Laboratoire de Chimie de Coordination), Université de Toulouse, UPS, INPT, 205 route de Narbonne, BP 44099, F-31077 Toulouse Cedex 4, France CNRS. Fax: +33-561553003; Tel: +33-561333174; E-mail: rinaldo.poli@lcc-toulouse.fr.*

[b] *Department of Chemistry, University of York, Heslington, York, YO10 5DD U.K. E-mail: john.slattery@york.ac.uk.*

[c] *Departament de Química, Universitat Autònoma de Barcelona, 08193 Cerdanyola del Vallès, Catalonia, Spain. E-mail: Agusti.Lledos@uab.cat.*

[d] *Institut Universitaire de France, 1, rue Descartes, 75231 Paris Cedex 05, France.*

SUPPORTING INFORMATION

Index

General Experimental S2

NMR characterisation of [IrCl(COD)((*S*)-P,S^{Cy})] S3

Computational Methods S7

Computed Gibbs energy profiles S9

3D-views of the optimized structures along the ketone hydrogenation S10

Optimized structures of hydride transfer step in isopropanol solvent S12

Optimized structures of hydride transfer *pro-S* and *pro-R* transition states with [M(iPrOH)$_5$]$^+$ (M = Li, Na; isopropanol solvent) S12

References S16

Cartesian coordinates (Å) and energies (hartrees) for all computed molecules S18

Experimental

General. All reactions and purifications were carried out under argon using high-vacuum or Schlenk line techniques. The (*S*)-P,S^R ligands[1] used in the experiments were synthesised according to the published procedures. The bases NaO^{i}Pr and LiO^{i}Pr were prepared from sodium and lithium in isopropanol. The commercially available compounds KO^{t}Bu (Alfa Aesar, 97%),18-crown-6 (TCI, 98%), $[Ir(COD)Cl]_2$ (Strem) and acetophenone (TCI, 98.5%) were used as received. The chromatographic analyses were obtained on an HP 5890 instrument equipped with a chiral Supelco BETA DEX 225 column. NMR spectra were recorded using the Bruker Avance 400 instrument available in the Laboratoire de chimie de coordination in Toulouse. NMR measurements were carried out using NMR tubes fitted with J. Young's valves

Synthesis of [IrCl(COD)((*S*)-P,S^{Cy})].

$[IrCl(COD)]_2$ (80 mg, 0.12 mmol) was added to (*S*)-P,S^{Cy} ligand (120 mg, 0.24 mmol), dry dichloromethane (10 mL) was then added to the flask *via* cannula and set to stir at room temperature for 1 h. At this point the solution was concentrated to dryness *in vacuo*, and the solid product washed with pentane (2 x 10 mL) to yield a solid powder. Yield = 148 mg (75 %). ESI-HRMS (m/z relative intensity %): [M^-Cl^+] calcd: 835.1563; found: 799.1796. The NMR analysis of the product is given in Table S1.

Asymmetric hydrogenations.

A. With the isolated [IrCl(COD)((*S*)-P,S^R)] complex. In a 5 mL glass ampoule were placed the [IrCl(COD)(P,S^R)] complex with the desired ((*S*)-P,S^R) ligand ($6.4\cdot10^{-3}$ mmol), the desired base ($3.2\cdot10^{-2}$ mmol, 5 equiv.) and 2 mL of ^{i}PrOH. Then, acetophenone (0.38 mL, 384 mg, 3.2 mmol, 500 equiv.) was added and the ampoule was placed into a stainless-steel autoclave. The reaction vessel was hermetically closed and pressurized to the required H_2 pressure (30 bars), then thermostatically regulated at the desired

temperature and stirred with a magnetic bar for the desired time. The reaction was stopped by release of the H_2 pressure and the solution was filtered through silica with CH_2Cl_2 elution at room temperature. The product was finally analysed by chiral GC. Yield and *ee* errors in Table 1 of the main section were calculated by running each reaction three times. The standard error was calculated according to the equation below:

$$Standard\ error = \frac{\sigma}{\sqrt{3}}$$

Where σ = standard deviation obtained experimentally.

B. With the *in situ* generated [IrCl(COD)((*S*)-P,S^{R})] complex. These catalytic experiments followed the same procedure as in A, after preparing the pre-catalyst solution by mixing $[IrCl(COD)]_2$, the desired ((*S*)-P,S^{R}) ligand, the base and ^{i}PrOH.

NMR characterisation of [IrCl(COD)((*S*)-P,S^{Cy})]

The ^{31}P{^{1}H} NMR spectrum for [IrCl(COD)((*S*)-P,S^{Cy})] shows a single peak in chloroform-*d* at *δ* 2.2. The ^{1}H NMR data for the complex is far less clear for the assignment of each individual proton, this is due to the crowded saturated CH region of the spectrum resulting in overlapping peaks (Figure S1). Nevertheless, the peaks could be assigned based on the known chemical shifts using a combination of ^{1}H-^{1}H COSY (Figure S2), and ^{13}C 135-DEPT (Figure S3) experiments for the CH_2 groups on the cyclohexyl and COD groups. The full characterisation of [IrCl(COD)((*S*)-P,S^{Cy})] is presented in Table S1.

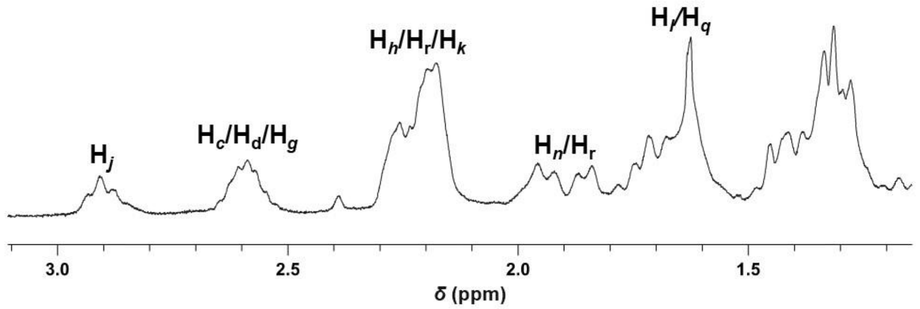

Figure S1 ^{1}H NMR spectrum for the CH_2 region for [IrCl(COD)((*S*)-P,S^{Cy})] in chloroform-*d*.

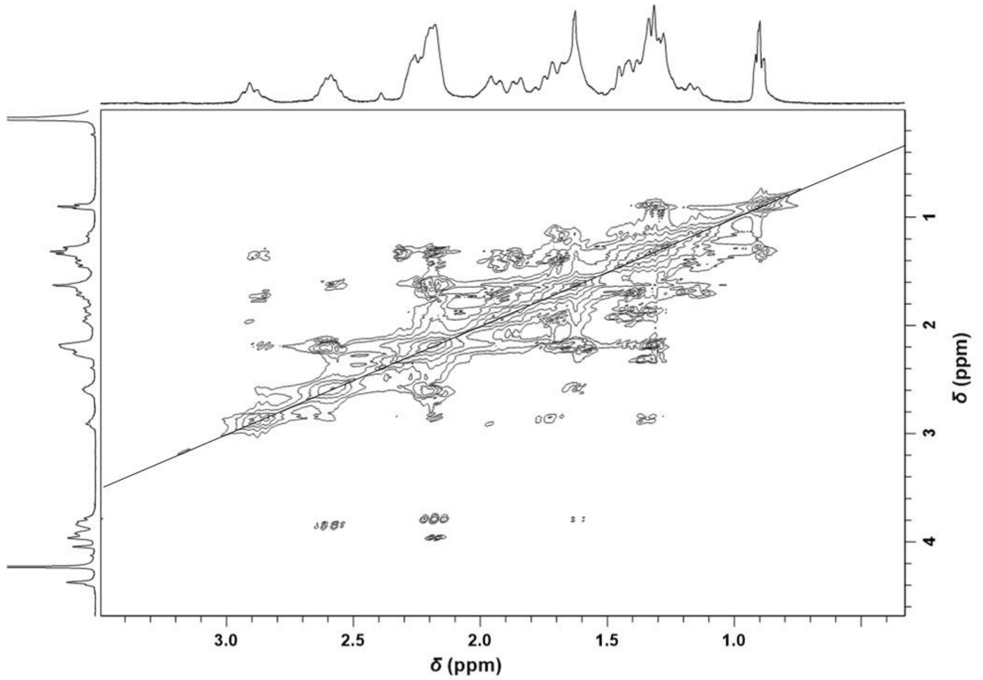

Figure S2 ^{1}H-^{1}H COSY NMR spectrum of [IrCl(COD)((*S*)-P,S^{Cy})] in chloroform-*d* at 298 K.

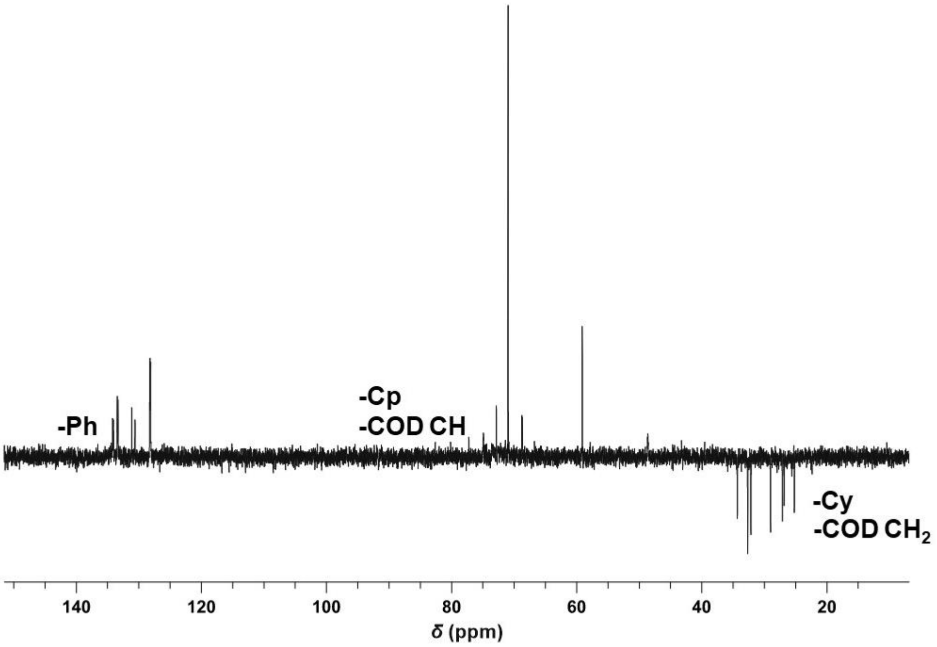

Figure S3 ^{13}C 135-DEPT NMR spectrum highlighting the different CH regions on [IrCl(COD)((*S*)-P,S^{Cy})].

Table S1. NMR data for [IrCl(COD)((*S*)-P,S^{Cy})] in chloroform-*d* at 298 K

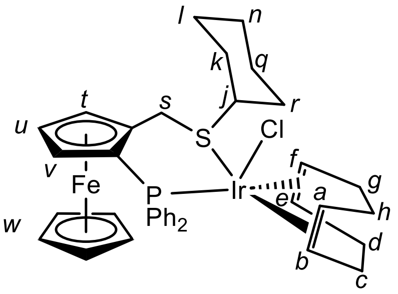

Group	Chemical Shift (*δ*)
**^{1}H**, Ph (*ortho*)<br>Ph (*meta*)<br>Ph (*para*)	7.91 (*o*); 7.32 (*o*')<br>7.56 (*m*); 7.36 (*m*')<br>7.55 (*p*); 7.39 (*p*')
C_8H_{12} CH	3.80 (*a*); 3.86 (*b*); 3.82 (e); 3.98 (*f*)
C_8H_{12} CH_2	2.61, 2.23 (*c*); 2.58, 1.65 (*d*); 2.65, 2.25 (*g*); 2.19, 1.63 (*h*)
C_6H_{11} CH	2.84 (*j*)
C_6H_{11} CH_2	2.35, 1.34 (*k*); 1.64, 1.31 (*l*); 1.95, 1.47 (*n*); 1.43, 1.18 (*q*); 2.33, 1.76 (*r*)
S – CH_2	4.30, 3.90 (*s*)
Cp	4.04 (*t*); 4.34 (*u*); 4.78 (*v*); 4.20 (*w*)
**^{13}C**, Ph (*ortho*)<br>Ph (*meta*)<br>Ph (*para*)	134.0 ($^{3}J_{CP}$ = 10.8, *o*); 133.2 (d, $^{3}J_{CP}$ = 11, *o*')<br>128.2 ($^{3}J_{CP}$ = 7.0, *m*); 128.3 (d, $^{3}J_{CP}$ = 7.3, *m*')<br>131.1 ($^{4}J_{CP}$ = 2.5, *p*); 133.2 (s, *p*')
C_8H_{12} CH	71.8 (*a*); 60.1 (*b*); 71.4 (e); 71.3 (*f*)
C_8H_{12} CH_2	34.8 (*c*); 32.1 (d, J_{CP} = 1.9, *d*); 27.1 (*d*, J_{CP} = 1.9, *g*); 31.9 (*h*)
C_6H_{11} CH	48.6 (*j*)
C_6H_{11} CH_2	34.3 (*k*); 31.8 (*l*); 24.7 (*n*); 26.8 (*q*); 31.7 (*r*)
S – CH_2	27.1 (*s*)
Cp	72.8 (d, $^{3}J_{CP}$ = 2.2, *t*); 68.7 (d, $^{3}J_{CP}$ = 6.1, *u*); 75.0 (d, $^{2}J_{PC}$ = 6.6, *v*); 70.9 (*w*)
**^{31}P**, P,S^{Cy}	2.24

The assignment of the *ipso* ^{13}C resonances for the Ph and Cp ring was prevented due to overlap by other major resonances. Specific *J*-couplings for the ^{1}H chemical shifts could not be deciphered due to the crowded spectral regions.

Computational details. The main features of the chemical and computational models employed in the previous paper[2] were used in the calculations: the actual $[Ir(H)_4((S)\text{-}P,S^{Ph})]^-$ complex, with no simplifications, was the catalyst used in the calculations and the experimental isopropanol solvent was modelled with a continuum model + explicit solvent molecules. Acetone and acetophenone were the substrates to be hydrogenated. Initially, the complete energy profiles for acetone hydrogenation were computed modelling the isopropanol used in the experiments using a methanol solvation model. This simplification substantially simplifies configurational space for the explicit solvent molecules, allowing this to be surveyed more reliably. Although this solvent description is not expected to lead to significant differences in the model used, to assess its validity and to get more reliable results we recomputed all the structures of the key hydride transfer step using isopropanol in the hybrid continuum-explicit solvent model. Preliminary results showed us that calculations regarding enantioselectivity, with acetophenone substrate, required a description of the system as realistic as possible. For this reason, they were performed using the isopropanol solvent model. DFT calculations with the M06 functional[3] were carried out using the Gaussian 16 suite of programs[4] to analyse how the alkali-metal cation influences the activity and enantioselectivity of the ketone hydrogenation in alcohol solvent. As we discuss later on, a proper description of the solvent is crucial to reproduce the experimental trends. There is increasing evidence that modelling catalytic processes in protic solvents, such as alcohols, demands the inclusion of explicit solvent molecules to account for specific solvent-solute interactions.[5] For this reason, the solvent was described by means of a cluster-continuum approach, also referred to as a hybrid implicit-explicit solvation scheme. In addition to the SMD polarizable continuum model for methanol (ε = 35.7) or isopropanol (ε = 19.3),[6] explicit solvent molecules (3 or 5) were introduced to describe the solvation sphere of the cation. The effect of the solvent was included both during geometry optimisations and single-point

calculations. Optimisations were performed using basis set BS1, which includes the 6-31G(d,p) basis set for the main-group atoms,[7, 8] and the scalar relativistic Stuttgard-Dresden (SDD) effective core potential (ECP) and its associated double-ξ basis set,[9] complemented with a set of *f* polarisation functions[10] for the Fe and Ir atoms. Frequency calculations were carried out for all the optimized geometries in order to characterise the stationary points as either minima or transition states. Connections between the transition states and the corresponding minima were checked by displacing in both directions, following the transition vector, the geometry of the transition states, and subsequent geometry optimisation until a minimum was reached.

Energies in alcohol solvent were refined by means of single-point calculations at the optimized BS1 geometries using the same functional (M06), adding Grimme's D3 correction to account for long-range dispersion effects,[11] and an extended basis set (BS2). BS2 consists in the def2-TZVP basis set for the main group atoms, and the quadruple-ζ def2-QZVP basis set for transition metals, together with the def2 ECP for Ir.[12, 13] Gibbs energies in methanol and isopropanol solvents were calculated adding to the BS2 energies in solvent the thermal and entropic corrections obtained with BS1. An additional correction of 1.9 kcal/mol was applied to all Gibbs energies to change the standard state from the gas phase (1 atm) to the condensed phase (1 M) at 298.15K ($\boldsymbol{\Delta G^{1atm\rightarrow 1M}}$).[14] In this way, all the energy values given in the article are Gibbs energies in solvent calculated using the formula:

$$\boldsymbol{G = E(BS2) + G(BS1) - E(BS1) + \Delta G^{1atm\rightarrow 1M}}$$

3D-structures were generated using CYLview.[15]

Computed Gibbs energy profiles

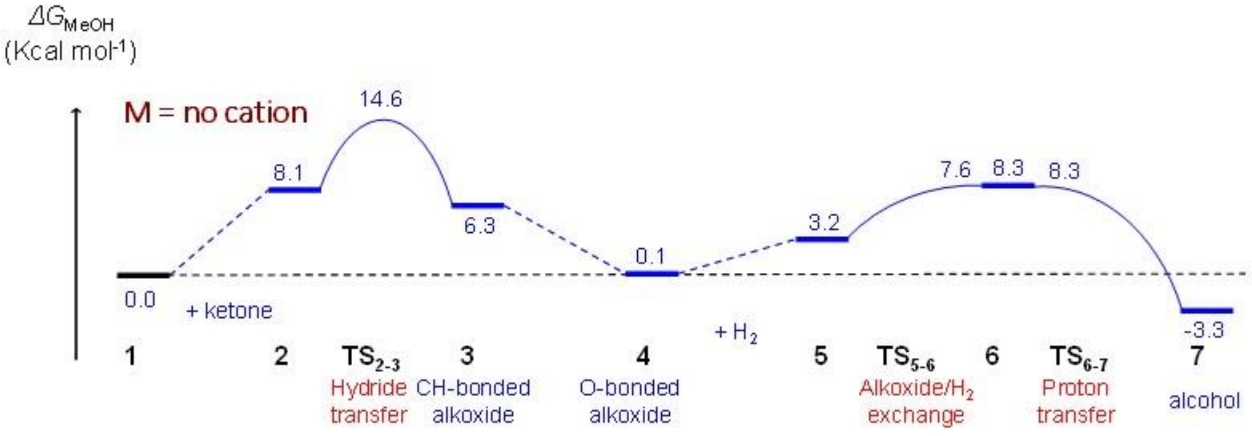

Figure S4. Computed Gibbs energy profiles for ketone hydrogenation catalysed by the cation-free $[Ir(H)_5(P,S^{Ph})]^-\cdot 2MeOH$ complex. The profile follows the catalytic cycle of Scheme 1, except that 2 MeOH molecules were explicitly included in the calculations.

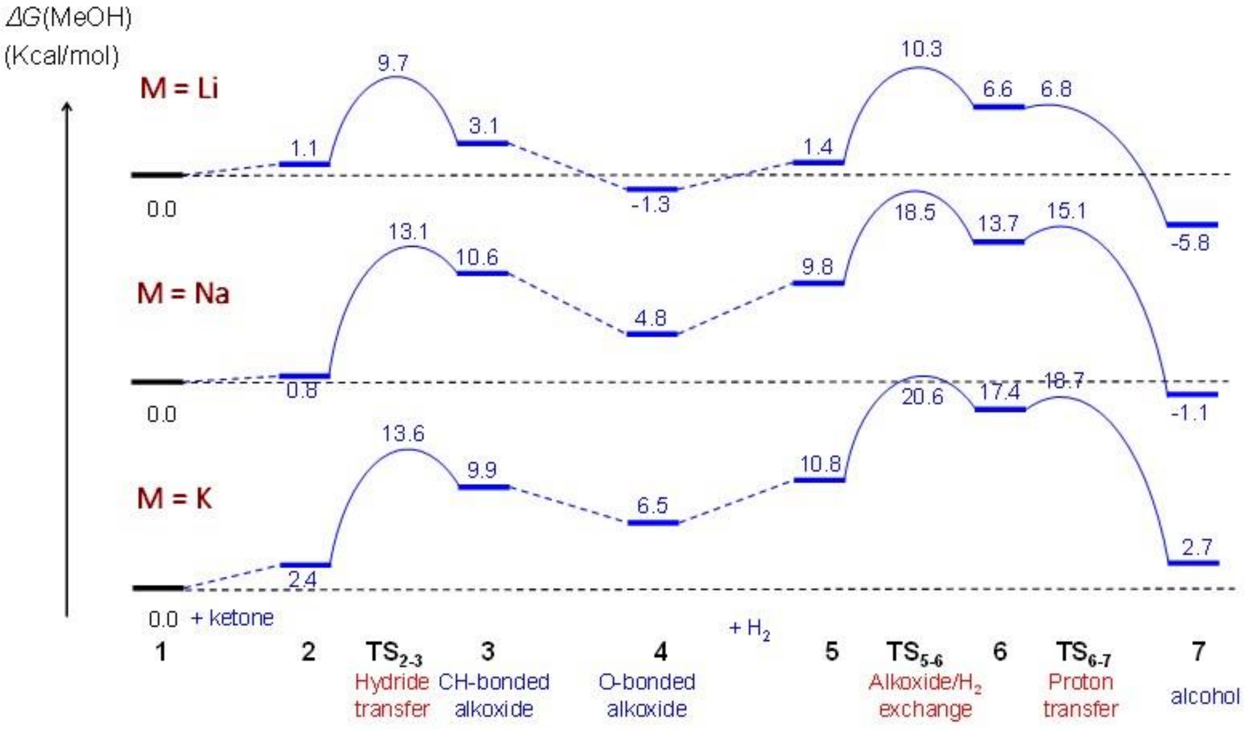

Figure S5. Computed Gibbs energy profiles for ketone hydrogenation catalysed by the $[M(MeOH)_3]^+[Ir(H)_5(P,S^{Ph})]^-$ complex. The profile follows the catalytic cycle of Scheme 1.

3D-views of the optimized structures along the ketone hydrogenation

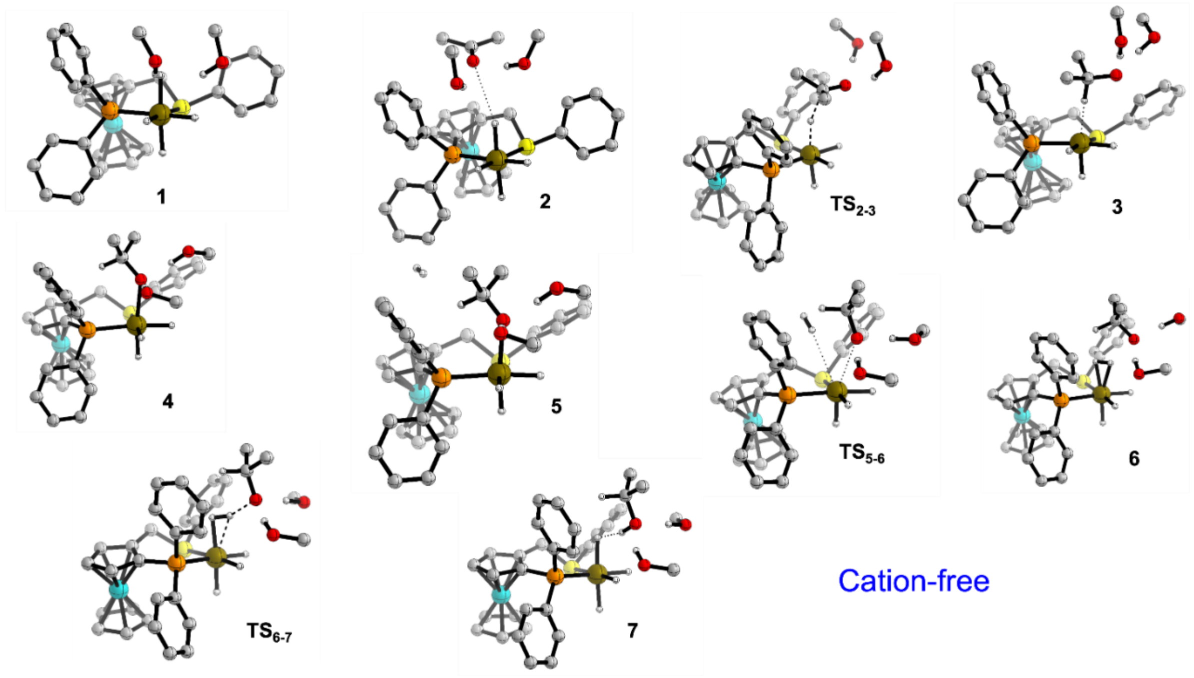

Figure S6. Optimized structures along the acetone hydrogenation following the catalytic cycle of Scheme 1, for the cation-free pathway (Gibbs energy profile of Figure S4). 2 MeOH molecules were explicitly included in the calculations. C-H hydrogen atoms have been omitted for clarity.

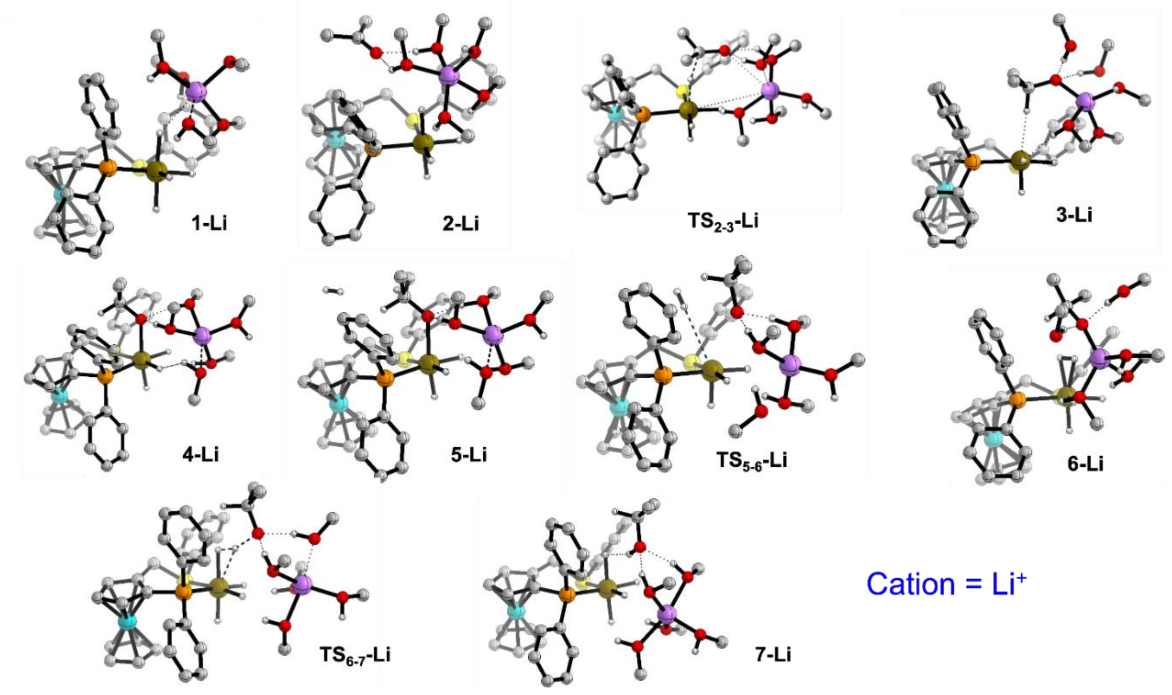

Figure S7. Optimized structures along the acetone hydrogenation following the catalytic cycle of Scheme 1, with the $[Li(MeOH)_5]^+$ solvent model (Gibbs energy profile of Figure S5, cation = Li^+). C-H hydrogen atoms have been omitted for clarity.

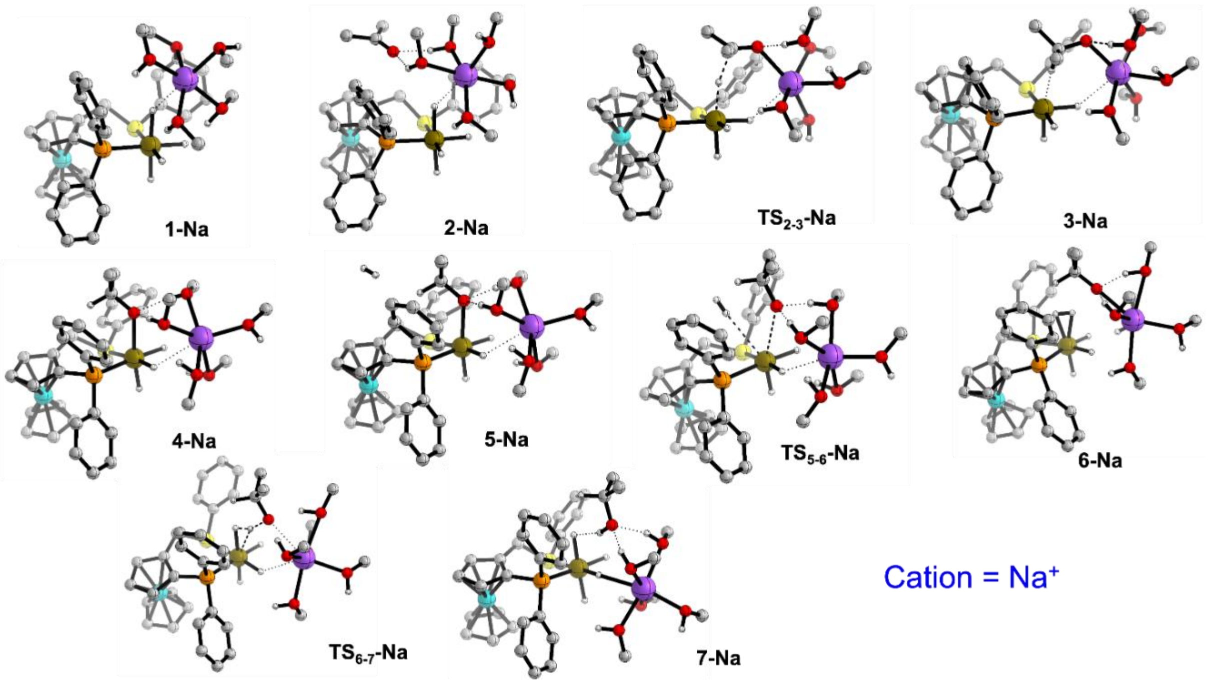

Figure S8. Optimized structures along the acetone hydrogenation following the catalytic cycle of Scheme 1, with the $[Na(MeOH)_5]^+$ solvent model (Gibbs energy profile of Figure S5, cation = Na^+). C-H hydrogen atoms have been omitted for clarity.

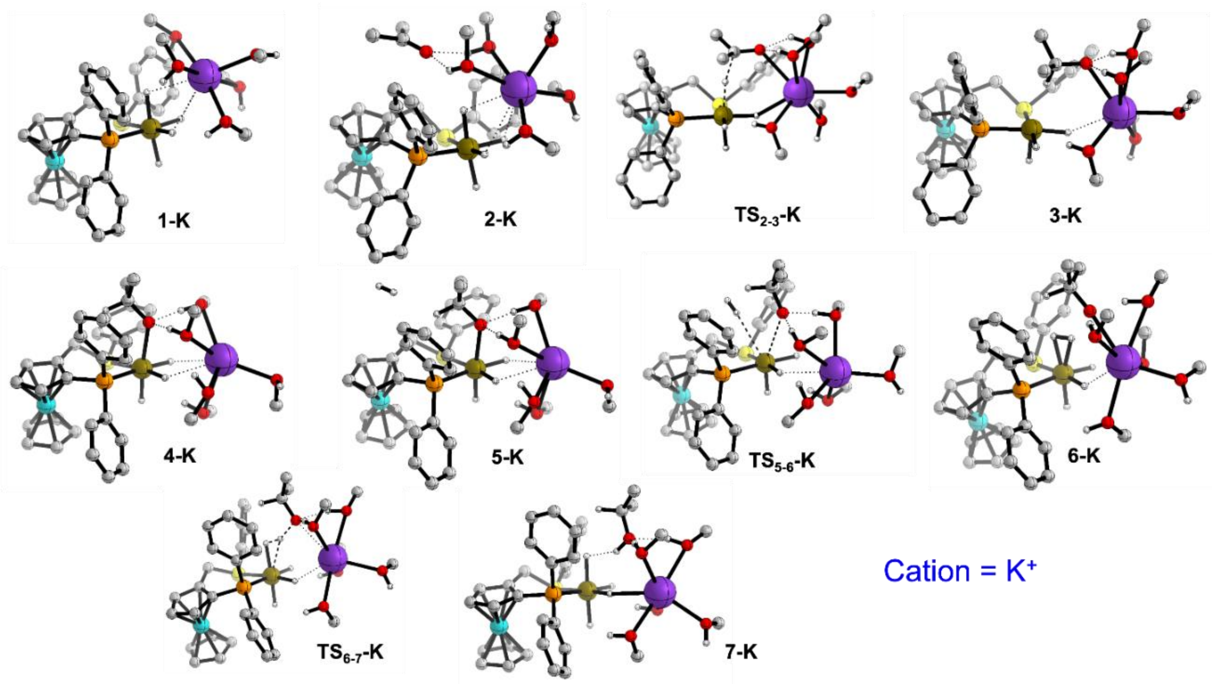

Figure S9. Optimized structures along the acetone hydrogenation following the catalytic cycle of Scheme 1, with the $[K(MeOH)_5]^+$ solvent model (Gibbs energy profile of Figure S5, cation = K^+). C-H hydrogen atoms have been omitted for clarity.

Optimized structures of hydride transfer step in isopropanol solvent

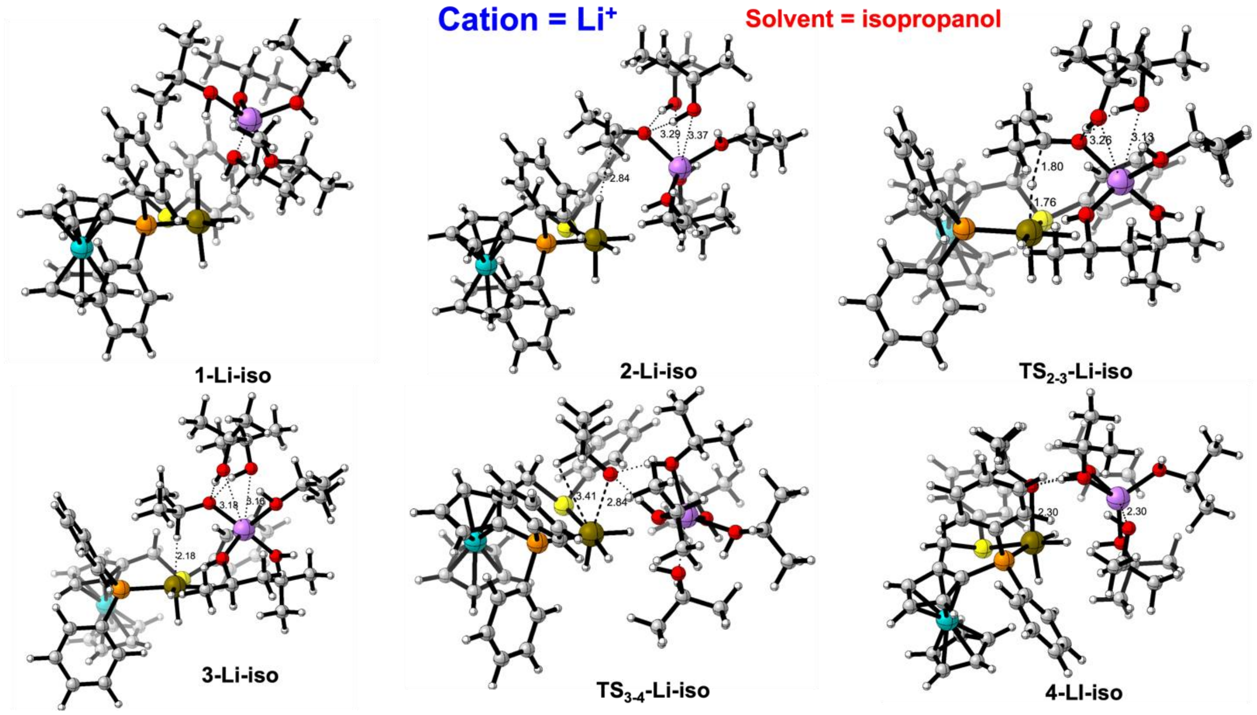

Figure S10. Optimized structures along the hydride transfer step of acetone hydrogenation in isopropanol solvent with the $[Li(iPrOH)_5]^+$ solvent model.

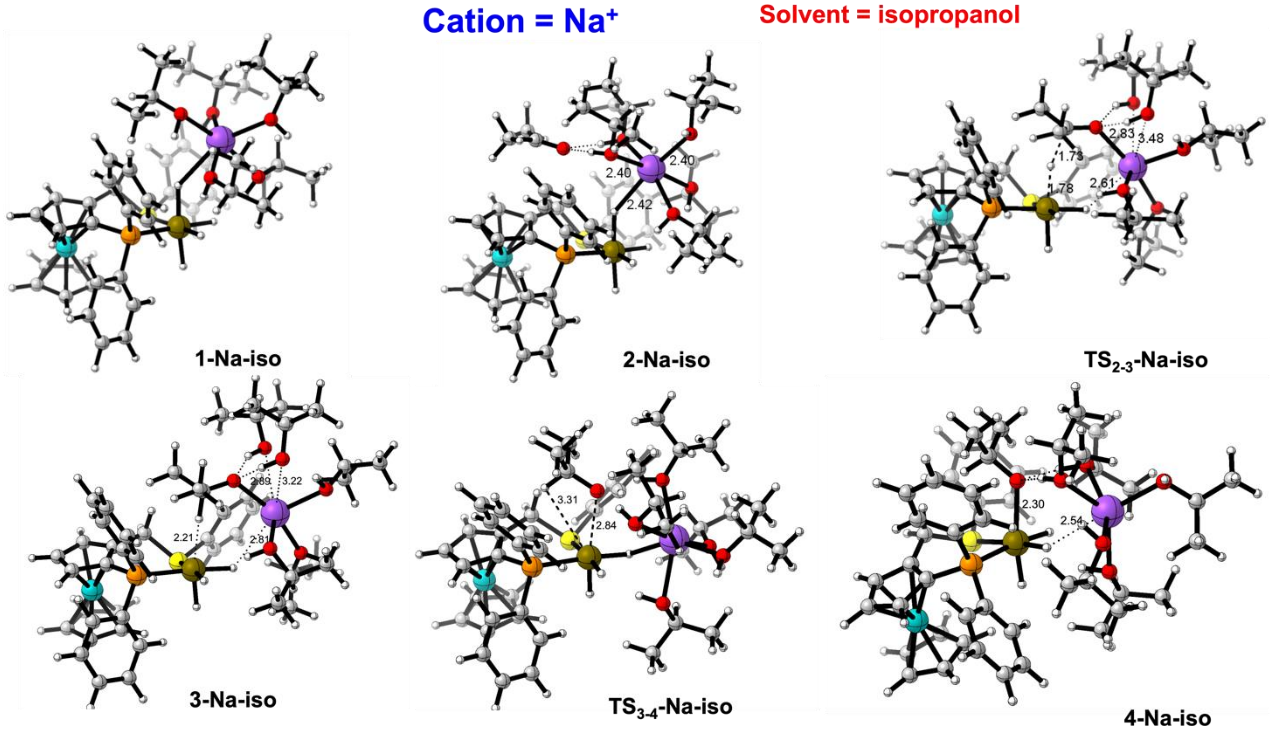

Figure S11. Optimized structures along the hydride transfer step of acetone hydrogenation in isopropanol solvent with the $[Na(iPrOH)_5]^+$ solvent model.

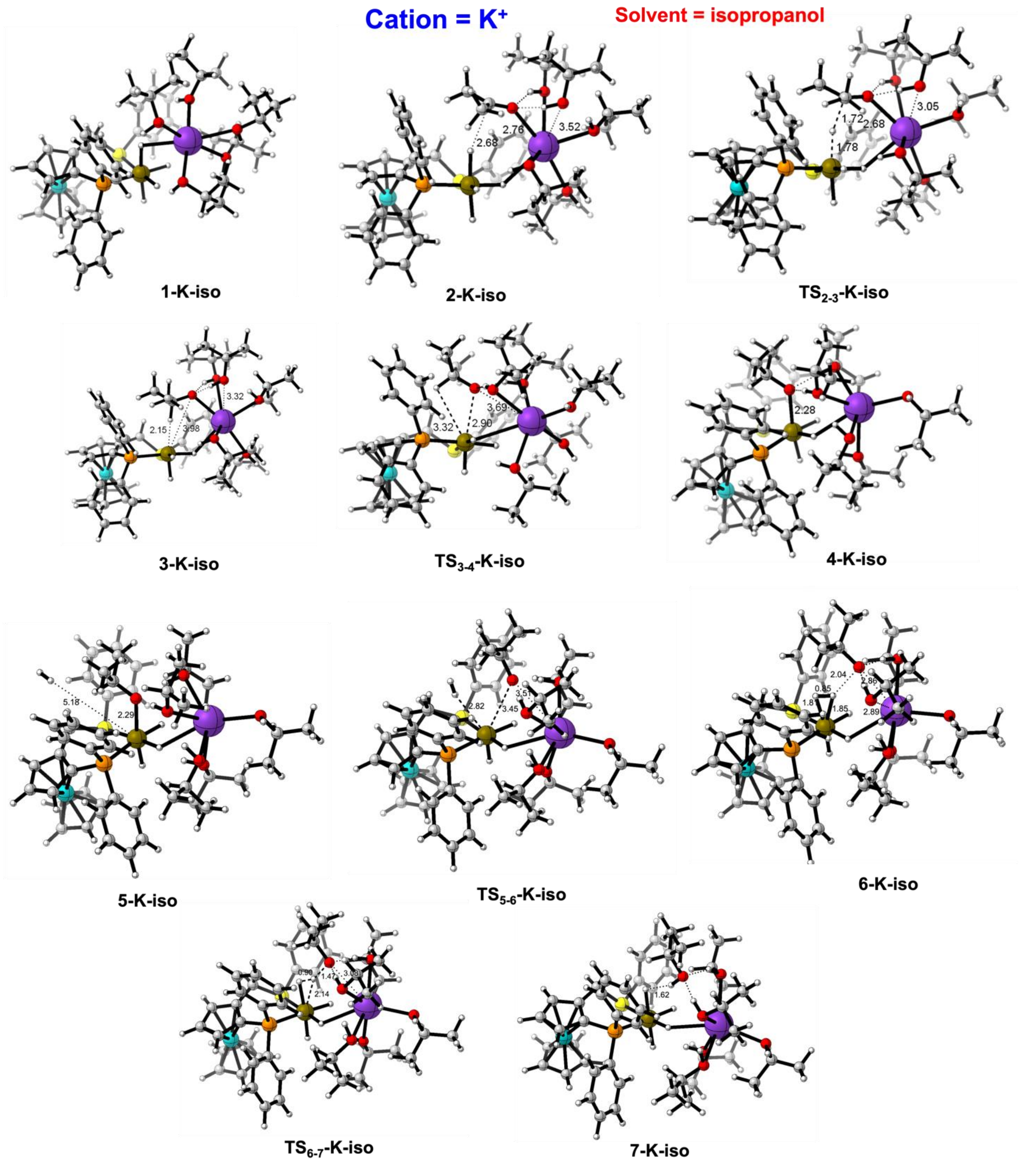

Figure S12. Optimized structures along the acetone hydrogenation following the catalytic cycle of Scheme 1, with the $[K(iPrOH)_5]^+$ solvent model (Gibbs energy profile of Figure 4).

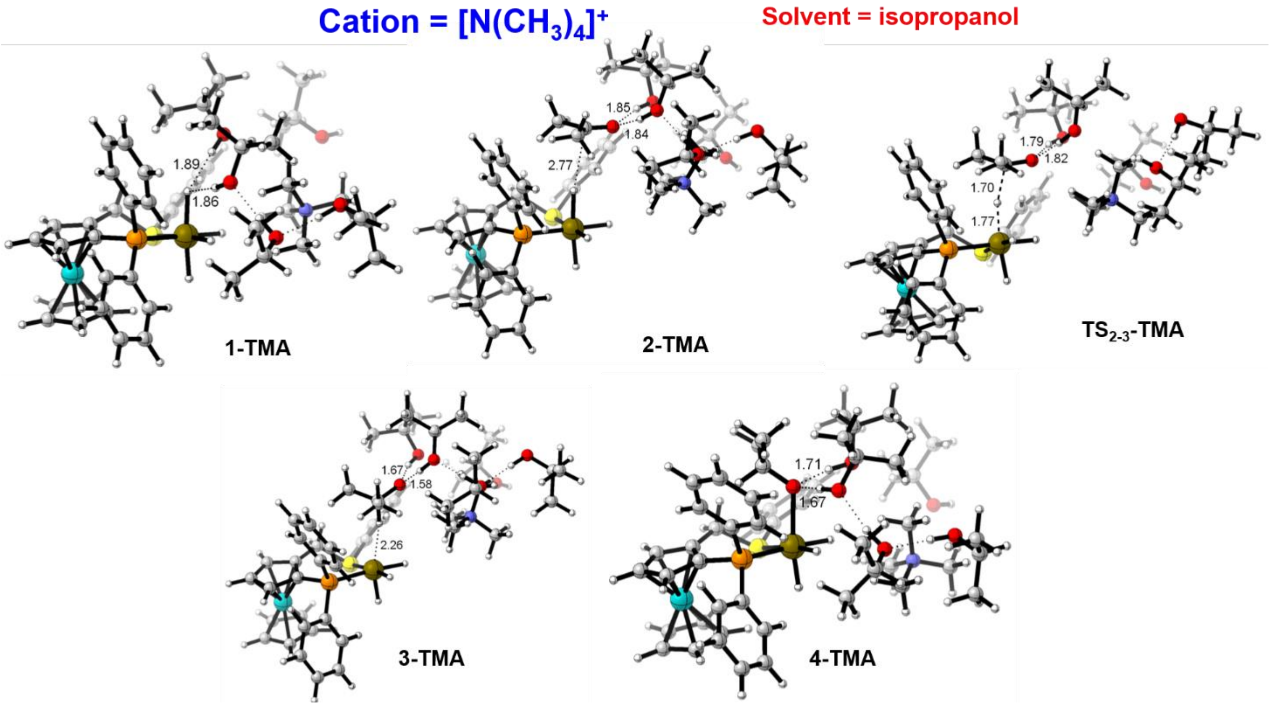

Figure S13. Optimized structures along the hydride transfer step of acetone hydrogenation in isopropanol solvent with the $[TMA(iPrOH)_5]^+$ (TMA = tetramethylammonium) solvent model.

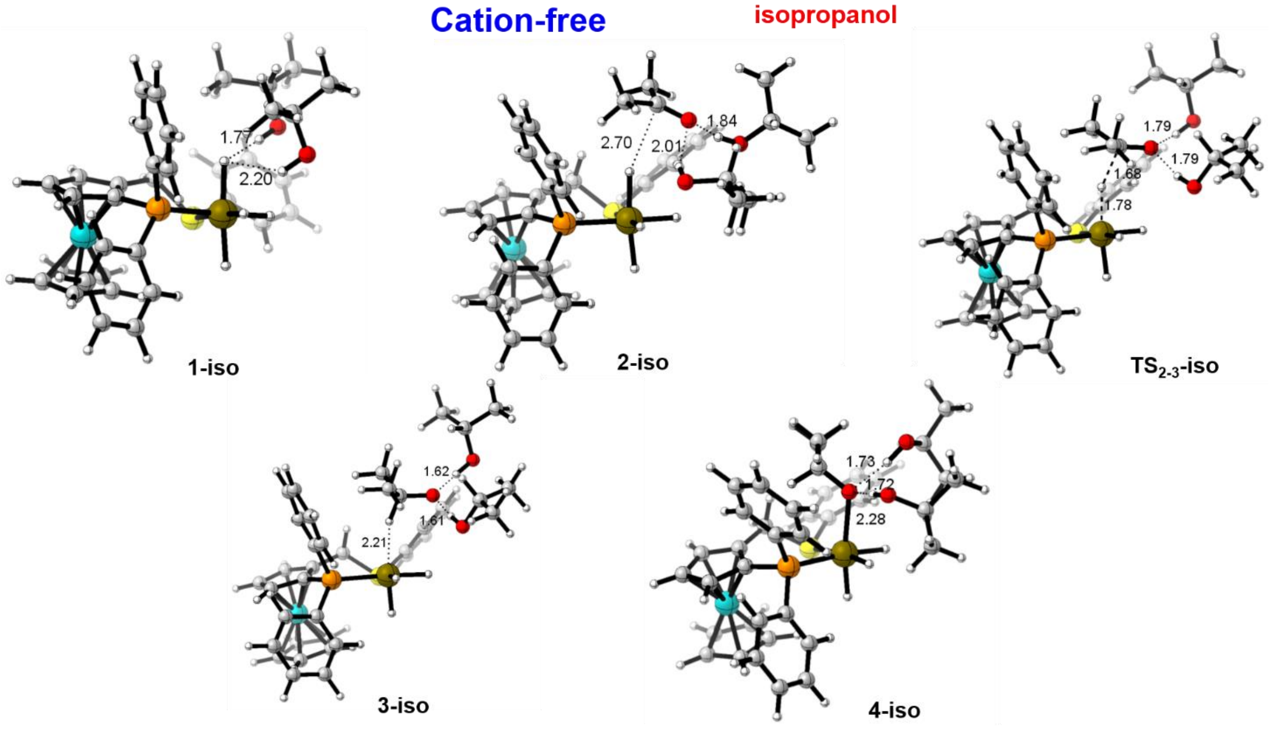

Figure S14. Optimized structures along the hydride transfer step of acetone hydrogenation in isopropanol solvent, for the cation-free pathway. 2 iPrOH solvent molecules were explicitly included in the calculations.

Optimized structures of hydride transfer *pro-S* and *pro-R* transition states with $[M(iPrOH)_5]^+$ (M = Li, Na; isopropanol solvent)

isopropanol

Li$^+$

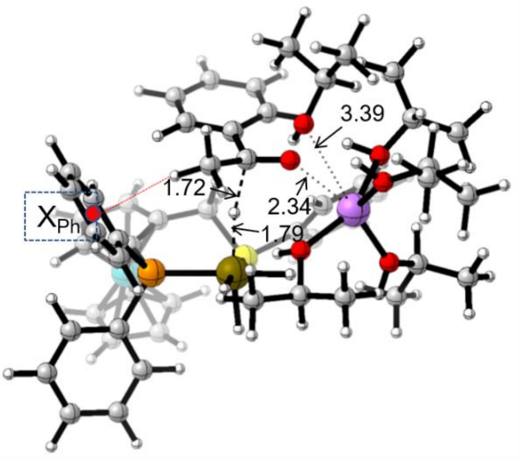

$TS_{2\text{-}3}$-*pro-S*-Li

$X_{Ph}\cdots HC_{Me}$: 2.52 Å

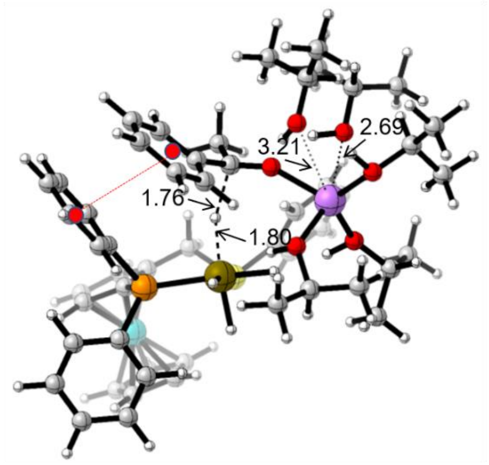

$TS_{2\text{-}3}$-*pro-R*-Li

$X_{Ph}\cdots X_{Ph}$: 3.85 Å

isopropanol

Na$^+$

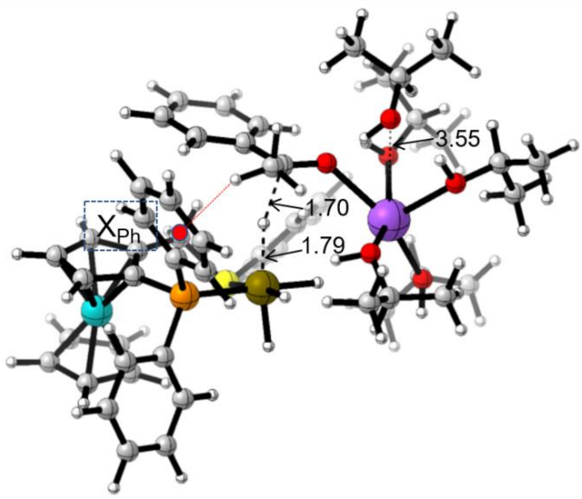

$TS_{2\text{-}3}$-*pro-S*-Na

$X_{Ph}\cdots HC_{Me}$: 2.53 Å

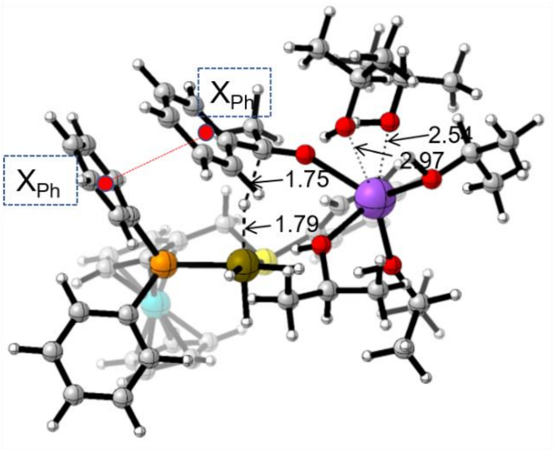

$TS_{2\text{-}3}$-*pro-S*-Na

$X_{Ph}\cdots X_{Ph}$: 3.87 Å

Figure S15. *pro-S* and *pro-R* transition states of the hydride transfer to acetophenone for the $[M(iPrOH)_5]^+$ model with M = Li and Na. Distances in Å.

References

1. L. Routaboul, S. Vincendeau, J.-C. Daran and E. Manoury, New ferrocenyl P,S and S,S ligands for asymmetric catalysis, *Tetrahedron: Asymmetry*, 2005, **16**, 2685-2690.
2. J. M. Hayes, E. Deydier, G. Ujaque, A. Lledós, R. Malacea-Kabbara, E. Manoury, S. Vincendeau and R. Poli, Ketone Hydrogenation with Iridium Complexes with "non N–H" Ligands: The Key Role of the Strong Base, *ACS Catal.*, 2015, **5**, 4368–4376.
3. Y. Zhao and D. G. Truhlar, The M06 suite of density functionals for main group thermochemistry, thermochemical kinetics, noncovalent interactions, excited states, and transition elements: two new functionals and systematic testing of four M06-class functionals and 12 other functionals, *Theor. Chem. Acc.*, 2008, **120**, 215-241.
4. M. J. Frisch*, et al.*, *Gaussian 16, Revision C.01*, Gaussian, Inc., Wallingford CT, 2016.
5. G. Norjmaa, G. Ujaque and A. Lledos, Beyond Continuum Solvent Models in Computational Homogeneous Catalysis, *Top. Catal.*, 2022, **65**, 118-140.
6. A. V. Marenich, C. J. Cramer and D. G. Truhlar, Universal Solvation Model Based on Solute Electron Density and on a Continuum Model of the Solvent Defined by the Bulk Dielectric Constant and Atomic Surface Tensions, *J. Phys. Chem. B*, 2009, **113**, 6378-6396.
7. W. J. Hehre, R. Ditchfield and J. A. Pople, Self-Consistent Molecular-Orbital Methods .12. Further Extensions of Gaussian-Type Basis Sets for Use in Molecular-Orbital Studies of Organic-Molecules, *J. Chem. Phys.*, 1972, **56**, 2257-2261.
8. M. M. Francl, W. J. Pietro, W. J. Hehre, J. S. Binkley, M. S. Gordon, D. J. Defrees and J. A. Pople, Self-Consistent Molecular-Orbital Methods .23. A Polarization-Type Basis Set for 2nd-Row Elements, *J. Chem. Phys.*, 1982, **77**, 3654-3665.
9. D. Andrae, U. Haussermann, M. Dolg, H. Stoll and H. Preuss, Energy-Adjusted Abinitio Pseudopotentials for the 2nd and 3rd Row Transition-Elements, *Theor. Chem. Acc.*, 1990, **77**, 123-141.
10. A. W. Ehlers, M. Böhme, S. Dapprich, A. Gobbi, A. Hoellwarth, V. Jonas, K. F. Koehler, R. Stegmann, A. Veldkamp and G. Frenking, A set of f-polarization functions for pseudopotential basis sets of the transition metals Sc-Cu, Y-Ag and La-Au, *Chem. Phys. Lett.*, 1993, **208**, 111-114.
11. S. Grimme, J. Antony, S. Ehrlich and H. Krieg, A consistent and accurate ab initio parametrization of density functional dispersion correction (DFT-D) for the 94 elements H-Pu, *J. Chem. Phys.*, 2010, **132**, 154104.
12. F. Weigend and R. Ahlrichs, Balanced basis sets of split valence, triple zeta valence and quadruple zeta valence quality for H to Rn: Design and assessment of accuracy, *Phys. Chem. Chem. Phys.*, 2005, **7**, 3297-3305.
13. F. Weigend, Accurate Coulomb-fitting basis sets for H to Rn, *Phys. Chem. Chem. Phys.*, 2006, **8**, 1057-1065.

14. V. S. Bryantsev, M. S. Diallo and W. A. Goddard, III, Calculation of solvation free energies of charged solutes using mixed cluster/continuum models, *J. Phys. Chem. B*, 2008, **112**, 9709-9719.
15. C. Y. Legault, *CYLview20*, 2020 Université de Sherbrooke (http://www.cylview.org).

Cartesian coordinates (Å) and energies (hartrees) for all computed molecules

Table S2. Cartesian coordinates (Å) and energies (hartrees) for all computed molecules.

Methanol solvent

A. Small molecules

H_2
E (BS1) = -1.169264336
E (BS2) = -1.170390954
$G_{298.15, 1M}$ (BS2) = -1.168766954

H	0.000000	0.000000	0.371492
H	0.000000	0.000000	-0.371492

Me_2CO
E (BS1) = -193.0357063
E (BS2) = -193.110345
$G_{298.15,1M}$ (BS2) = -193.051313

O	-0.000081	1.402090	-0.000009
C	-0.000006	0.179530	0.000036
C	-1.271807	-0.611407	-0.002764
C	1.271879	-0.611296	0.002768
H	-1.245430	-1.381953	-0.782487
H	-2.135982	0.042013	-0.146575
H	-1.370767	-1.139084	0.954704
H	1.370933	-1.138705	-0.954856
H	2.135968	0.042179	0.146823
H	1.245522	-1.382133	0.782221

Me_2CHOH
E (BS1) = -194.2367677
E (BS2) = -194.3104704
$G_{298.15,1M}$ (BS2) = -194.2272147

H	0.009907	0.088334	1.471347
O	0.099925	1.361033	-0.165085
C	-0.001692	0.042631	0.367467
C	1.154097	-0.816341	-0.102300
C	-1.335200	-0.503051	-0.088591
H	2.115048	-0.396214	0.220585
H	1.157759	-0.878241	-1.198595
H	1.080430	-1.833532	0.301143
H	-1.502251	-1.508682	0.312795
H	-2.157625	0.141582	0.242198
H	-1.363371	-0.559892	-1.184912
H	0.957477	1.718946	0.096662

PhCOMe
E (BS1) = -384.6292006
E (BS2) = -384.7683424
$G_{298.15,1M}$ (BS2) = -384.6606585

O	2.212757	-1.305625	-0.000160
C	1.689091	-0.196095	0.000164
C	2.528638	1.044691	0.000004
H	2.313796	1.659713	0.882146
H	3.585430	0.767911	-0.000205
H	2.313456	1.659795	-0.881996
C	0.208301	-0.058525	0.000065
C	-0.422546	1.190566	0.000002
C	-0.572969	-1.220223	0.000064
C	-1.810167	1.273679	-0.000048
C	-1.957606	-1.136709	0.000017
C	-2.577773	0.111994	-0.000040
H	0.165699	2.105489	-0.000001
H	-0.074761	-2.186964	0.000113
H	-2.294213	2.247540	-0.000093
H	-2.557608	-2.043883	0.000026
H	-3.663664	0.179133	-0.000073

PhCHOHMe
E (BS1) = -385.8261715
E (BS2) = -385.9660737
$G_{298.15,1M}$ (BS2) = -385.8355818

H	-1.840861	1.081913	1.037374
O	-2.208835	-0.939669	0.746304
C	-1.643614	0.283004	0.300151
C	-2.240681	0.695843	-1.035143
H	-3.326095	0.833611	-0.948583
H	-2.044979	-0.079248	-1.787491
H	-1.804822	1.638546	-1.386381
H	-3.169266	-0.847064	0.712277
C	-0.150717	0.108394	0.187186
C	0.410471	-1.120849	-0.161871
C	0.688509	1.208511	0.371028
C	1.788403	-1.247031	-0.316907
C	2.065043	1.085666	0.210536
C	2.619573	-0.145343	-0.132032
H	-0.238004	-1.982861	-0.306059
H	0.253975	2.170259	0.646106
H	2.215106	-2.212172	-0.583666
H	2.707375	1.951265	0.360469
H	3.696337	-0.246063	-0.252157

B. 5MeOH model

B1. Cation-free system

1
E (BS1) = -2320.979687
E (BS2) = -3461.326657
$G_{298.15,1M}$ (BS2) = -3460.806471

1	-2.015910000	2.959823000	-0.532382000
8	-2.415092000	3.853330000	-0.461159000
6	-2.234272000	4.286073000	0.868963000
1	-2.957828000	5.083170000	1.079318000
1	-2.406599000	3.478097000	1.597468000
1	-1.226099000	4.693901000	1.045543000
26	2.093017000	-2.312073000	0.933548000
77	-1.007046000	0.611046000	-1.176951000
15	1.054392000	0.854583000	-0.101672000
16	-1.415057000	-1.601165000	-0.154820000
6	1.464954000	-0.387604000	1.175699000

6	0.540756000	-1.305402000	1.795505000
6	1.262487000	-2.051311000	2.777595000
1	0.840255000	-2.845526000	3.385984000
6	2.614442000	-1.609978000	2.780011000
1	3.418385000	-2.017461000	3.383767000
6	2.743017000	-0.593214000	1.796204000
1	3.664023000	-0.085963000	1.529532000
6	1.361604000	-3.857879000	-0.191228000
1	0.324812000	-4.176383000	-0.199975000
6	1.936922000	-2.861396000	-1.032231000
1	1.412731000	-2.291783000	-1.793467000
6	3.305949000	-2.706593000	-0.661401000
1	4.007411000	-1.997121000	-1.090552000
6	3.575097000	-3.610609000	0.407542000
1	4.515711000	-3.707350000	0.939394000
6	2.373556000	-4.320731000	0.698842000
1	2.241882000	-5.052641000	1.488622000
6	-0.932151000	-1.457115000	1.604344000
1	-1.466315000	-0.592732000	2.025376000
1	-1.262862000	-2.369025000	2.117413000
6	1.193199000	2.419340000	0.862339000
6	1.406018000	3.624862000	0.183278000
1	1.534021000	3.623725000	-0.899825000
6	1.473695000	4.826220000	0.881024000
1	1.653399000	5.754092000	0.341684000
6	1.308455000	4.842142000	2.265255000
1	1.362697000	5.781680000	2.811081000
6	1.066930000	3.651720000	2.944304000
1	0.929557000	3.656202000	4.023845000
6	1.012450000	2.445967000	2.247392000
1	0.830177000	1.519457000	2.792955000
6	2.615622000	0.906560000	-1.090575000
6	3.799555000	1.450159000	-0.574038000
1	3.805318000	1.913059000	0.412112000
6	4.976782000	1.416472000	-1.314255000
1	5.887662000	1.842377000	-0.898345000
6	4.987237000	0.848771000	-2.587113000
1	5.907662000	0.827759000	-3.166910000
6	3.813471000	0.322756000	-3.117359000
1	3.809701000	-0.110026000	-4.115682000
6	2.635801000	0.354280000	-2.373179000
1	1.714398000	-0.051355000	-2.790804000
6	-3.179806000	-1.899956000	-0.018519000
6	-3.835581000	-2.188521000	-1.220148000
1	-3.276221000	-2.197673000	-2.155419000
6	-5.199312000	-2.446130000	-1.222223000
1	-5.701956000	-2.664470000	-2.162060000
6	-5.919978000	-2.427702000	-0.028306000
1	-6.988408000	-2.630861000	-0.031132000
6	-5.262697000	-2.154598000	1.164915000
1	-5.814256000	-2.140539000	2.102839000
6	-3.892661000	-1.889484000	1.179012000
1	-3.408679000	-1.672425000	2.127283000
1	-0.748925000	2.006561000	-1.942168000
1	-2.439065000	0.586369000	-2.004013000
1	-1.768197000	1.379545000	0.164408000
1	-0.386758000	-0.141812000	-2.547848000
8	-4.510515000	1.110494000	-0.375403000
6	-4.766188000	1.613862000	0.914363000
1	-5.805564000	1.381111000	1.175712000
1	-4.120499000	1.155594000	1.681420000
1	-4.643307000	2.707647000	0.971206000
1	-3.540460000	1.101875000	-0.514762000

2

E (BS1) = -2514.024200

E (BS2) = -3654.444149

$G_{298.15,1M}$ (BS2) = -3653.844879

1	-1.727104000	2.866438000	-0.325578000
8	-1.988188000	3.605415000	-0.896961000
6	-2.740667000	4.509939000	-0.122047000
1	-2.983284000	5.370407000	-0.755443000
1	-3.686747000	4.069517000	0.230977000
1	-2.185642000	4.882132000	0.753701000
26	1.948685000	-1.674393000	1.862411000
77	-0.504399000	-0.229815000	-1.991227000
15	1.219248000	0.627150000	-0.672392000
16	-1.059541000	-1.968385000	-0.337092000
6	1.240611000	0.073525000	1.073252000
6	0.191395000	-0.642272000	1.758964000
6	0.598439000	-0.810096000	3.119568000
1	0.032129000	-1.347870000	3.874590000
6	1.879124000	-0.213618000	3.288590000
1	2.471068000	-0.218065000	4.197728000
6	2.278330000	0.321194000	2.034187000
1	3.224511000	0.810752000	1.827671000
6	1.600674000	-3.634041000	1.386999000
1	0.615253000	-4.069282000	1.258710000
6	2.375973000	-3.006938000	0.368970000
1	2.084433000	-2.886236000	-0.669568000
6	3.577422000	-2.519810000	0.964857000
1	4.360233000	-1.962668000	0.458890000
6	3.543408000	-2.848507000	2.351320000
1	4.292439000	-2.580273000	3.088855000
6	2.321599000	-3.535620000	2.612505000
1	1.980395000	-3.881736000	3.582410000
6	-1.143340000	-1.099421000	1.270426000
1	-1.811589000	-0.237799000	1.137712000
1	-1.579549000	-1.791099000	2.003672000
6	1.171949000	2.461731000	-0.486126000
6	1.374108000	3.241832000	-1.631454000
1	1.586684000	2.757696000	-2.585369000
6	1.307782000	4.628080000	-1.566072000
1	1.469462000	5.219321000	-2.465275000
6	1.031688000	5.259000000	-0.353504000
1	0.977265000	6.344479000	-0.301946000
6	0.828717000	4.492785000	0.789054000
1	0.617601000	4.977031000	1.741785000
6	0.898505000	3.101468000	0.723888000
1	0.730254000	2.516421000	1.627122000
6	2.992964000	0.400059000	-1.160865000
6	4.006139000	1.242763000	-0.685170000
1	3.757772000	2.092234000	-0.049417000
6	5.336222000	1.014612000	-1.024653000
1	6.110853000	1.679102000	-0.647105000
6	5.673515000	-0.053421000	-1.854003000
1	6.713184000	-0.227671000	-2.123536000
6	4.673461000	-0.885748000	-2.347559000
1	4.926256000	-1.713449000	-3.007311000
6	3.342774000	-0.656318000	-2.004779000
1	2.557093000	-1.298562000	-2.403233000
6	-2.774449000	-2.447842000	-0.562951000
6	-3.005199000	-3.410201000	-1.550739000
1	-2.166784000	-3.820253000	-2.113693000
6	-4.299836000	-3.835444000	-1.818645000
1	-4.469540000	-4.583093000	-2.590444000
6	-5.372776000	-3.312731000	-1.098255000

1	-6.386473000	-3.647545000	-1.306028000
6	-5.138648000	-2.362335000	-0.111173000
1	-5.970141000	-1.949299000	0.457196000
6	-3.843303000	-1.924565000	0.162634000
1	-3.692727000	-1.167188000	0.928212000
1	-0.195849000	0.884073000	-3.116328000
1	-1.764816000	-0.677564000	-2.971840000
1	-1.581339000	0.849317000	-1.247549000
1	0.459598000	-1.324537000	-2.877700000
8	-4.208035000	1.353369000	0.086348000
6	-5.277536000	1.384259000	1.002217000
1	-5.255733000	0.541735000	1.714240000
1	-5.304209000	2.318390000	1.586903000
1	-6.214117000	1.315921000	0.437474000
1	-3.388493000	1.498089000	0.594304000
6	-2.010796000	1.974534000	2.945927000
8	-2.068032000	2.151608000	1.731686000
6	-2.845248000	0.942178000	3.630568000
6	-1.102224000	2.786624000	3.808883000
1	-3.259627000	0.224490000	2.915290000
1	-0.745560000	3.675298000	3.280250000
1	-3.674086000	1.453446000	4.139968000
1	-2.274425000	0.424983000	4.410641000
1	-0.240645000	2.163435000	4.090125000
1	-1.601831000	3.069308000	4.742603000

TS$_{2-3}$
E (BS1) = -2514.011564
E (BS2) = -3654.431727
G$_{298.15,1M}$ (BS2) = -3653.834443

1	5.187920000	-2.313238000	-0.960384000
8	5.882289000	-2.998315000	-0.881328000
6	6.650178000	-2.625005000	0.239314000
1	7.478846000	-3.334824000	0.345156000
1	7.077639000	-1.615339000	0.134725000
1	6.070461000	-2.653206000	1.178709000
26	-3.204291000	1.576531000	0.997059000
77	0.533155000	-0.230474000	-1.393306000
15	-1.230428000	-1.090798000	-0.105561000
16	0.244711000	2.023941000	-0.475526000
6	-1.930300000	-0.003408000	1.186769000
6	-1.327665000	1.200799000	1.701819000
6	-2.167936000	1.693682000	2.747707000
1	-1.994306000	2.610769000	3.302728000
6	-3.276548000	0.814176000	2.891936000
1	-4.111112000	0.944697000	3.572648000
6	-3.137246000	-0.224274000	1.932761000
1	-3.844614000	-1.029814000	1.766254000
6	-3.154896000	3.250344000	-0.176992000
1	-2.301342000	3.914783000	-0.263935000
6	-3.398885000	2.093326000	-0.972480000
1	-2.764531000	1.726125000	-1.773188000
6	-4.592795000	1.473218000	-0.496467000
1	-5.025352000	0.550255000	-0.871385000
6	-5.086382000	2.250370000	0.591744000
1	-5.957902000	2.020849000	1.195694000
6	-4.197403000	3.347410000	0.790041000
1	-4.275453000	4.098052000	1.569396000
6	-0.038077000	1.853608000	1.340894000
1	0.819483000	1.294700000	1.742272000
1	-0.018101000	2.868258000	1.758545000
6	-0.761214000	-2.565291000	0.894832000
6	-0.584260000	-3.796653000	0.252319000
1	-0.787958000	-3.886568000	-0.815326000
6	-0.155577000	-4.911183000	0.964521000
1	-0.031450000	-5.863633000	0.453150000
6	0.117987000	-4.807407000	2.327532000
1	0.454888000	-5.678865000	2.884881000
6	-0.039564000	-3.584023000	2.971486000
1	0.173420000	-3.494892000	4.034949000
6	-0.479999000	-2.468608000	2.260157000
1	-0.603214000	-1.517330000	2.778686000
6	-2.757378000	-1.703893000	-0.948671000
6	-3.629352000	-2.603320000	-0.320967000
1	-3.384143000	-3.009833000	0.659403000
6	-4.809717000	-2.997627000	-0.942182000
1	-5.475546000	-3.697224000	-0.440995000
6	-5.133331000	-2.506179000	-2.205518000
1	-6.054188000	-2.819174000	-2.693103000
6	-4.266936000	-1.625497000	-2.845571000
1	-4.505864000	-1.247010000	-3.837322000
6	-3.086230000	-1.229382000	-2.220720000
1	-2.405331000	-0.544529000	-2.725184000
6	1.758384000	2.994972000	-0.358774000
6	1.624902000	4.363732000	-0.112502000
1	0.633018000	4.802140000	-0.003262000
6	2.759257000	5.161800000	-0.011229000
1	2.653941000	6.227526000	0.180054000
6	4.023652000	4.597320000	-0.164541000
1	4.910470000	5.223111000	-0.090692000
6	4.152984000	3.234523000	-0.415504000
1	5.135242000	2.779686000	-0.533938000
6	3.020923000	2.426791000	-0.512932000
1	3.116585000	1.359211000	-0.712060000
1	0.690446000	-1.672886000	-2.113074000
1	1.775023000	0.241046000	-2.381919000
1	1.784971000	-0.734813000	-0.239054000
1	-0.466632000	0.204912000	-2.630101000
8	6.276755000	0.777066000	-0.068345000
6	6.335908000	0.995602000	1.320932000
1	5.470108000	1.565284000	1.697769000
1	6.401788000	0.054826000	1.892140000
1	7.236833000	1.580031000	1.540933000
1	5.470197000	0.243606000	-0.237301000
6	3.250759000	-1.284073000	0.371772000
8	4.151481000	-0.923303000	-0.441745000
6	3.125580000	-0.550443000	1.682526000
6	2.863629000	-2.738916000	0.401854000
1	3.245615000	0.529992000	1.542985000
1	2.746704000	-3.137900000	-0.610966000
1	3.941800000	-0.902052000	2.334173000
1	2.176967000	-0.760082000	2.189324000
1	1.949389000	-2.908323000	0.977434000
1	3.686037000	-3.285291000	0.890200000

3
E (BS1) = -2514.027120
E (BS2) = -3654.448788
G$_{298.15,1M}$ (BS2) = -3653.847775

1	5.328967000	-1.937274000	-0.843090000
8	6.053214000	-2.621845000	-0.846794000
6	6.640352000	-2.591254000	0.427500000
1	7.367421000	-3.410322000	0.502420000
1	7.178569000	-1.649929000	0.624622000
1	5.907167000	-2.735135000	1.241813000
26	-3.148289000	1.614880000	1.036578000

77	0.463527000	-0.233634000	-1.451621000
15	-1.261903000	-1.092825000	-0.063437000
16	0.288312000	1.995790000	-0.448456000
6	-1.917245000	0.008033000	1.234038000
6	-1.277903000	1.198534000	1.738062000
6	-2.099973000	1.721127000	2.783398000
1	-1.898286000	2.636004000	3.332345000
6	-3.230462000	0.872268000	2.939752000
1	-4.057683000	1.030076000	3.623549000
6	-3.124379000	-0.177712000	1.988968000
1	-3.854606000	-0.964390000	1.831632000
6	-3.073145000	3.276990000	-0.153391000
1	-2.209482000	3.926989000	-0.247732000
6	-3.336586000	2.116133000	-0.937044000
1	-2.708770000	1.730206000	-1.734179000
6	-4.539797000	1.520139000	-0.453634000
1	-4.987182000	0.599858000	-0.817703000
6	-5.019406000	2.316089000	0.627188000
1	-5.893382000	2.106077000	1.234624000
6	-4.112714000	3.400582000	0.813458000
1	-4.177857000	4.160375000	1.585014000
6	0.034202000	1.801852000	1.370577000
1	0.872646000	1.199718000	1.750828000
1	0.106907000	2.808105000	1.802194000
6	-0.745839000	-2.553633000	0.927560000
6	-0.621203000	-3.800126000	0.301901000
1	-0.894287000	-3.911623000	-0.748047000
6	-0.155217000	-4.901852000	1.010649000
1	-0.072261000	-5.866782000	0.514861000
6	0.212142000	-4.768236000	2.348601000
1	0.580816000	-5.629323000	2.901827000
6	0.106494000	-3.529695000	2.973817000
1	0.391653000	-3.418115000	4.018003000
6	-0.374291000	-2.427487000	2.268878000
1	-0.458425000	-1.464048000	2.772919000
6	-2.792705000	-1.705563000	-0.884242000
6	-3.666365000	-2.582998000	-0.228894000
1	-3.416972000	-2.966221000	0.759769000
6	-4.851616000	-2.985160000	-0.834889000
1	-5.519922000	-3.667113000	-0.313341000
6	-5.176794000	-2.524124000	-2.109184000
1	-6.101687000	-2.843513000	-2.584641000
6	-4.307888000	-1.666417000	-2.776403000
1	-4.548489000	-1.313301000	-3.776876000
6	-3.122132000	-1.261175000	-2.167510000
1	-2.443328000	-0.594118000	-2.696549000
6	1.811775000	2.949290000	-0.375317000
6	1.688923000	4.334537000	-0.241584000
1	0.700138000	4.789404000	-0.186791000
6	2.830824000	5.126127000	-0.182581000
1	2.735160000	6.205020000	-0.081351000
6	4.090350000	4.536234000	-0.261532000
1	4.983611000	5.155920000	-0.219817000
6	4.206974000	3.155819000	-0.395241000
1	5.184147000	2.677310000	-0.447173000
6	3.068601000	2.352984000	-0.453147000
1	3.177799000	1.271677000	-0.563254000
1	0.473121000	-1.633947000	-2.261713000
1	1.597797000	0.267952000	-2.547639000
1	2.090228000	-0.801867000	-0.352851000
1	-0.602527000	0.272995000	-2.481849000
8	6.148477000	0.673498000	0.273963000
6	6.055263000	0.966693000	1.642495000
1	5.222592000	1.652705000	1.879358000
1	5.933413000	0.064518000	2.265902000
1	6.982087000	1.461099000	1.960350000
1	5.338996000	0.155009000	0.003853000
6	3.072232000	-1.285269000	0.035411000
8	4.137129000	-0.790534000	-0.650504000
6	3.097359000	-0.932444000	1.519833000
6	2.888119000	-2.785255000	-0.164799000
1	3.182459000	0.154248000	1.660151000
1	2.829826000	-3.024399000	-1.234908000
1	3.972628000	-1.403175000	1.993856000
1	2.196608000	-1.284610000	2.043612000
1	1.979892000	-3.159443000	0.324751000
1	3.751694000	-3.323082000	0.252360000

4

E (BS1) = -2514.043725
E (BS2) = -3654.462914
$G_{298.15,1M}$ (BS2) = -3653.857685

1	1.352429000	-0.804077000	1.961671000
1	0.866114000	-1.693389000	-1.971303000
1	-0.042764000	0.271798000	-2.350026000
1	2.550355000	-2.749102000	-0.359255000
8	2.588531000	-3.660802000	-0.753537000
6	3.394529000	-3.615369000	-1.906321000
1	3.173047000	-4.491010000	-2.529918000
1	3.201464000	-2.717575000	-2.516996000
1	4.470523000	-3.634784000	-1.672950000
8	2.473382000	-1.165174000	0.264537000
6	2.389752000	-1.068772000	1.660110000
6	3.317047000	0.011917000	2.208299000
6	2.723403000	-2.408000000	2.308396000
1	3.118238000	0.986375000	1.743156000
1	4.364668000	-0.249606000	2.000241000
1	3.205444000	0.125299000	3.295736000
1	2.645563000	-2.359738000	3.403517000
1	2.049429000	-3.197441000	1.951852000
1	3.752090000	-2.702981000	2.053597000
8	5.093073000	-1.549524000	-0.153517000
6	5.690798000	-0.393410000	-0.681048000
1	4.121516000	-1.365756000	-0.042401000
1	6.747952000	-0.604368000	-0.885989000
1	5.229018000	-0.072350000	-1.630709000
1	5.655405000	0.458424000	0.020100000
26	-2.899535000	1.900262000	0.676391000
77	0.885093000	-0.287067000	-1.191208000
15	-1.032372000	-0.970509000	-0.008059000
16	0.723887000	2.013343000	-0.289818000
6	-1.802109000	0.240770000	1.125189000
6	-1.180600000	1.424484000	1.669046000
6	-2.120131000	2.038641000	2.554364000
1	-1.952268000	2.970466000	3.086152000
6	-3.307943000	1.256759000	2.572198000
1	-4.218133000	1.489248000	3.114818000
6	-3.119383000	0.158045000	1.692139000
1	-3.860275000	-0.599429000	1.459062000
6	-2.538611000	3.481285000	-0.570894000
1	-1.622903000	4.063493000	-0.575749000
6	-2.780672000	2.297446000	-1.326546000
1	-2.079962000	1.821890000	-2.005972000
6	-4.079760000	1.814202000	-0.987575000
1	-4.541982000	0.907161000	-1.366124000
6	-4.639754000	2.702468000	-0.023679000
1	-5.601542000	2.588937000	0.465257000

6	-3.687184000	3.731530000	0.234901000
1	-3.799626000	4.537971000	0.951720000
6	0.196096000	1.966271000	1.476728000
1	0.939900000	1.377537000	2.029778000
1	0.225071000	2.996711000	1.850395000
6	-0.825038000	-2.430868000	1.098186000
6	-0.522750000	-3.664783000	0.510200000
1	-0.447135000	-3.744091000	-0.574763000
6	-0.327189000	-4.794985000	1.296266000
1	-0.096327000	-5.747548000	0.823576000
6	-0.424678000	-4.706169000	2.683921000
1	-0.271370000	-5.590057000	3.299495000
6	-0.716840000	-3.482457000	3.278168000
1	-0.794246000	-3.405234000	4.360926000
6	-0.917592000	-2.350384000	2.490222000
1	-1.148761000	-1.399805000	2.970209000
6	-2.483020000	-1.517308000	-1.017375000
6	-3.489315000	-2.322516000	-0.467173000
1	-3.400669000	-2.686969000	0.555690000
6	-4.607939000	-2.673234000	-1.216227000
1	-5.379914000	-3.297828000	-0.771363000
6	-4.734123000	-2.235426000	-2.532952000
1	-5.606605000	-2.514653000	-3.119963000
6	-3.732356000	-1.451746000	-3.096722000
1	-3.814688000	-1.116544000	-4.128646000
6	-2.615191000	-1.097088000	-2.343720000
1	-1.832693000	-0.487574000	-2.791835000
6	2.256987000	2.938166000	-0.111974000
6	2.201847000	4.258286000	0.343538000
1	1.248402000	4.702664000	0.628468000
6	3.368385000	5.011117000	0.418351000
1	3.324380000	6.036996000	0.777567000
6	4.584457000	4.461673000	0.016050000
1	5.493509000	5.056945000	0.065735000
6	4.632051000	3.153565000	-0.455377000
1	5.578089000	2.720201000	-0.776579000
6	3.470749000	2.386464000	-0.514672000
1	3.494559000	1.357298000	-0.870990000
1	2.160439000	0.084491000	-2.196330000

5

E (BS1) = -2515.217244
E (BS2) = -3655.638546
$G_{298.15,\ 1M}$ (BS2) = -3655.02145

1	1.341394000	-0.771072000	1.928834000
1	0.883975000	-1.696356000	-2.002791000
1	-0.038502000	0.257026000	-2.391939000
1	2.584269000	-2.734000000	-0.357740000
8	2.641020000	-3.648825000	-0.742466000
6	3.448479000	-3.600640000	-1.894331000
1	3.233373000	-4.478618000	-2.516929000
1	3.250921000	-2.705000000	-2.506659000
1	4.524256000	-3.613070000	-1.659345000
8	2.476382000	-1.145024000	0.246796000
6	2.382681000	-1.035918000	1.640658000
6	3.304953000	0.050065000	2.187545000
6	2.713281000	-2.367944000	2.304784000
1	3.108736000	1.022053000	1.716075000
1	4.353428000	-0.212668000	1.986071000
1	3.189740000	0.169725000	3.274136000
1	2.619760000	-2.312250000	3.398872000
1	2.048963000	-3.163688000	1.944473000
1	3.747415000	-2.658965000	2.067686000
8	5.103254000	-1.500427000	-0.148469000
6	5.693134000	-0.341361000	-0.678497000
1	4.128824000	-1.326671000	-0.046766000
1	6.754072000	-0.542507000	-0.873791000
1	5.235742000	-0.030804000	-1.633858000
1	5.643161000	0.514361000	0.017058000
1	0.684030000	-0.405991000	4.080623000
1	0.469013000	0.106495000	4.578961000
26	-2.913357000	1.877143000	0.683998000
77	0.887202000	-0.288588000	-1.225616000
15	-1.031038000	-0.981888000	-0.045805000
16	0.701048000	2.012442000	-0.325226000
6	-1.800068000	0.219598000	1.098050000
6	-1.181155000	1.400917000	1.649842000
6	-2.114135000	1.995025000	2.555643000
1	-1.947223000	2.921048000	3.097803000
6	-3.296387000	1.204577000	2.575802000
1	-4.201882000	1.423323000	3.131810000
6	-3.110616000	0.120061000	1.677717000
1	-3.847567000	-0.640870000	1.443003000
6	-2.571149000	3.472307000	-0.549828000
1	-1.656059000	4.055444000	-0.560511000
6	-2.822471000	2.296759000	-1.315384000
1	-2.130954000	1.829984000	-2.010056000
6	-4.116337000	1.808320000	-0.963858000
1	-4.583612000	0.905467000	-1.345915000
6	-4.663864000	2.685163000	0.017535000
1	-5.618664000	2.565079000	0.518466000
6	-3.708809000	3.712324000	0.274389000
1	-3.811904000	4.510352000	1.001986000
6	0.187037000	1.959150000	1.445749000
1	0.942891000	1.380012000	1.992671000
1	0.206518000	2.990192000	1.818545000
6	-0.816352000	-2.445007000	1.055053000
6	-0.484012000	-3.671641000	0.468245000
1	-0.396272000	-3.747388000	-0.616159000
6	-0.271027000	-4.798073000	1.255392000
1	-0.017533000	-5.745498000	0.783889000
6	-0.376601000	-4.711935000	2.642785000
1	-0.205311000	-5.592408000	3.258530000
6	-0.700384000	-3.495548000	3.235797000
1	-0.784200000	-3.418451000	4.318389000
6	-0.924690000	-2.369339000	2.446014000
1	-1.186820000	-1.426323000	2.924037000
6	-2.482684000	-1.523847000	-1.055481000
6	-3.479798000	-2.345981000	-0.513983000
1	-3.382431000	-2.728068000	0.501648000
6	-4.600994000	-2.689806000	-1.262599000
1	-5.365950000	-3.328011000	-0.824983000
6	-4.739061000	-2.227087000	-2.569543000
1	-5.613841000	-2.500382000	-3.155921000
6	-3.746577000	-1.425473000	-3.124625000
1	-3.838585000	-1.070176000	-4.148988000
6	-2.626534000	-1.078590000	-2.372474000
1	-1.850902000	-0.455327000	-2.813695000
6	2.226463000	2.951589000	-0.154493000
6	2.161241000	4.271217000	0.301135000
1	1.204887000	4.707445000	0.588822000
6	3.321603000	5.033779000	0.373352000
1	3.269884000	6.059171000	0.732933000
6	4.541322000	4.494426000	-0.031438000
1	5.445502000	5.097208000	0.016451000
6	4.598866000	3.186622000	-0.502641000
1	5.547951000	2.761139000	-0.825394000

6	3.443934000	2.409768000	-0.559273000
1	3.475507000	1.380488000	-0.914869000
1	2.164538000	0.086688000	-2.225408000

TS_{5-6}

E (BS1) = -2515.206405

E (BS2) = -3655.628141

$G_{298.15, 1M}$ (BS2) = -3655.014463

1	3.071378000	-1.373444000	2.090146000
1	1.004254000	-1.347130000	-1.917860000
1	-0.404665000	0.288840000	-2.231271000
1	2.943603000	-2.435386000	-0.692133000
8	2.822222000	-3.300352000	-1.183243000
6	3.665540000	-3.255691000	-2.307347000
1	3.588941000	-4.209256000	-2.844846000
1	3.386783000	-2.455121000	-3.013379000
1	4.724768000	-3.110901000	-2.036722000
8	3.264442000	-0.974828000	0.069315000
6	3.852830000	-1.202037000	1.312390000
6	4.685428000	-0.008410000	1.772271000
6	4.735987000	-2.449169000	1.296358000
1	4.097586000	0.919943000	1.757225000
1	5.547635000	0.128283000	1.101994000
1	5.072470000	-0.147225000	2.792003000
1	5.175128000	-2.648194000	2.284349000
1	4.168165000	-3.336389000	0.989604000
1	5.560934000	-2.313713000	0.579803000
8	5.097007000	-0.386678000	-1.709772000
6	5.371644000	0.988818000	-1.692627000
1	4.397406000	-0.583566000	-1.012744000
1	6.174022000	1.201429000	-2.411420000
1	4.501005000	1.602609000	-1.983868000
1	5.720088000	1.345009000	-0.706833000
1	1.620895000	0.485699000	1.715380000
1	1.737090000	0.635973000	2.439437000
26	-3.550785000	1.212946000	0.373973000
77	0.689736000	0.030312000	-1.157874000
15	-0.885454000	-1.035231000	0.214537000
16	0.027560000	2.292537000	-0.327871000
6	-2.078083000	0.010106000	1.116597000
6	-1.887412000	1.379216000	1.529660000
6	-3.049536000	1.775081000	2.263551000
1	-3.214065000	2.766510000	2.674959000
6	-3.955366000	0.680050000	2.306472000
1	-4.945696000	0.686955000	2.749140000
6	-3.365761000	-0.402027000	1.599923000
1	-3.827100000	-1.367267000	1.418711000
6	-3.509652000	2.704434000	-1.021929000
1	-2.795773000	3.521869000	-1.024340000
6	-3.331367000	1.435971000	-1.647014000
1	-2.453283000	1.122192000	-2.203932000
6	-4.474835000	0.633992000	-1.354448000
1	-4.626237000	-0.397323000	-1.657784000
6	-5.360172000	1.409995000	-0.550720000
1	-6.301532000	1.072118000	-0.130564000
6	-4.763483000	2.688209000	-0.343996000
1	-5.171240000	3.490900000	0.261303000
6	-0.711589000	2.278653000	1.367826000
1	0.090971000	2.011673000	2.065683000
1	-1.018843000	3.310343000	1.578054000
6	-0.108576000	-2.008390000	1.567711000
6	0.710712000	-3.083018000	1.199887000
1	0.813178000	-3.351352000	0.148254000
6	1.400785000	-3.808690000	2.163326000
1	2.028278000	-4.645524000	1.859895000
6	1.293640000	-3.458705000	3.509187000
1	1.840342000	-4.019942000	4.264169000
6	0.486866000	-2.387556000	3.882458000
1	0.400611000	-2.109569000	4.931179000
6	-0.215961000	-1.666829000	2.917418000
1	-0.839811000	-0.826777000	3.223725000
6	-1.997856000	-2.279390000	-0.562403000
6	-2.581088000	-3.307044000	0.188896000
1	-2.343347000	-3.411085000	1.247389000
6	-3.466541000	-4.200772000	-0.404635000
1	-3.912386000	-4.994851000	0.190795000
6	-3.778177000	-4.080572000	-1.757278000
1	-4.469298000	-4.781583000	-2.220723000
6	-3.195913000	-3.068668000	-2.515330000
1	-3.428402000	-2.976440000	-3.574273000
6	-2.307499000	-2.174846000	-1.921295000
1	-1.848364000	-1.387598000	-2.518486000
6	1.405660000	3.386798000	0.036947000
6	1.135526000	4.730796000	0.306329000
1	0.107748000	5.093307000	0.299898000
6	2.183702000	5.602868000	0.577707000
1	1.972509000	6.648264000	0.793128000
6	3.498593000	5.140653000	0.560964000
1	4.317773000	5.826253000	0.766408000
6	3.763620000	3.803588000	0.277874000
1	4.790774000	3.441363000	0.259266000
6	2.718395000	2.918568000	0.020197000
1	2.916733000	1.866367000	-0.192418000
1	1.790852000	0.679963000	-2.227376000

6

E (BS1) = -2515.209488

E (BS2) = -3655.63333

$G_{298.15, 1M}$ (BS2) = -3655.01332

1	2.912165000	-1.225823000	1.681214000
1	0.911436000	-1.274289000	-1.969753000
1	-0.497942000	0.280570000	-2.448181000
1	3.485501000	-2.203628000	-0.758115000
8	3.318525000	-3.059384000	-1.267013000
6	4.526483000	-3.327679000	-1.932454000
1	4.422539000	-4.254256000	-2.511191000
1	4.810939000	-2.528415000	-2.637297000
1	5.372917000	-3.467763000	-1.237158000
8	3.966380000	-0.858645000	-0.054696000
6	3.957590000	-1.041717000	1.321858000
6	4.460285000	0.204246000	2.044261000
6	4.783214000	-2.259778000	1.725189000
1	3.849068000	1.080085000	1.779758000
1	5.498688000	0.415080000	1.747051000
1	4.435994000	0.090842000	3.136995000
1	4.751743000	-2.447356000	2.807984000
1	4.415951000	-3.159229000	1.212722000
1	5.835012000	-2.111725000	1.435722000
8	6.346393000	-0.230645000	-0.882231000
6	6.149444000	1.130061000	-1.159451000
1	5.457142000	-0.558724000	-0.545896000
1	7.052532000	1.538612000	-1.631863000
1	5.308730000	1.311672000	-1.852279000
1	5.960686000	1.727989000	-0.248990000
1	2.104779000	-0.262000000	-0.258365000
1	1.531927000	0.032052000	0.281262000

26	-3.610597000	1.116862000	0.590119000
77	0.538935000	0.107932000	-1.234647000
15	-0.992163000	-1.086838000	0.109545000
16	-0.168241000	2.319068000	-0.393533000
6	-2.093401000	-0.104306000	1.169935000
6	-1.840054000	1.247034000	1.605042000
6	-2.927901000	1.639542000	2.444192000
1	-3.037359000	2.618753000	2.900279000
6	-3.841760000	0.553990000	2.541366000
1	-4.787551000	0.561637000	3.072542000
6	-3.334508000	-0.518653000	1.758992000
1	-3.825717000	-1.472327000	1.594855000
6	-3.714505000	2.585011000	-0.834722000
1	-3.007409000	3.401194000	-0.935999000
6	-3.604346000	1.302783000	-1.447003000
1	-2.793800000	0.972846000	-2.090181000
6	-4.710233000	0.510806000	-1.015923000
1	-4.889467000	-0.529257000	-1.273156000
6	-5.503972000	1.307296000	-0.139208000
1	-6.392826000	0.980115000	0.389717000
6	-4.887418000	2.587842000	-0.025680000
1	-5.228542000	3.405857000	0.599742000
6	-0.636721000	2.097927000	1.382877000
1	0.247020000	1.677550000	1.886905000
1	-0.818430000	3.102593000	1.783855000
6	-0.112081000	-2.148282000	1.325195000
6	0.727561000	-3.156285000	0.834788000
1	0.791867000	-3.338734000	-0.238526000
6	1.493885000	-3.920847000	1.706369000
1	2.143670000	-4.698721000	1.309317000
6	1.438614000	-3.682131000	3.079397000
1	2.044860000	-4.275654000	3.760702000
6	0.610965000	-2.679451000	3.573401000
1	0.563054000	-2.488351000	4.643535000
6	-0.164165000	-1.915105000	2.701198000
1	-0.808453000	-1.133239000	3.102655000
6	-2.145042000	-2.261233000	-0.702617000
6	-2.745313000	-3.299256000	0.020387000
1	-2.487132000	-3.455372000	1.067784000
6	-3.670845000	-4.138395000	-0.591032000
1	-4.133231000	-4.940384000	-0.019606000
6	-3.998585000	-3.956512000	-1.933236000
1	-4.720207000	-4.615940000	-2.410747000
6	-3.391447000	-2.939940000	-2.664628000
1	-3.633508000	-2.802847000	-3.716361000
6	-2.466916000	-2.097158000	-2.052267000
1	-1.988307000	-1.303814000	-2.625232000
6	1.225237000	3.427568000	-0.113617000
6	0.932014000	4.774147000	0.114546000
1	-0.103300000	5.113301000	0.106808000
6	1.963658000	5.676610000	0.348637000
1	1.733313000	6.724772000	0.525909000
6	3.285900000	5.238705000	0.347428000
1	4.093156000	5.945565000	0.525876000
6	3.574105000	3.897755000	0.112784000
1	4.606871000	3.553159000	0.107779000
6	2.547403000	2.984740000	-0.119043000
1	2.780684000	1.937856000	-0.309865000
1	1.609528000	0.797449000	-2.294371000

TS$_{6-7}$

E (BS1) = -2515.207435

E (BS2) = -3655.630388

G$_{298.15, 1M}$ (BS2) = -3655.01334

1	3.318812000	-1.347395000	2.071960000
1	1.207732000	-1.180101000	-1.785765000
1	-0.322140000	0.265920000	-2.371200000
1	3.280371000	-2.538814000	-0.582329000
8	3.176742000	-3.386231000	-1.101437000
6	4.065844000	-3.269025000	-2.184935000
1	4.013227000	-4.184594000	-2.787268000
1	3.818629000	-2.421750000	-2.846342000
1	5.113059000	-3.146562000	-1.859455000
8	3.661507000	-1.060583000	0.058464000
6	4.155701000	-1.147925000	1.359803000
6	4.812357000	0.160028000	1.784412000
6	5.141589000	-2.301968000	1.476184000
1	4.101999000	0.995901000	1.709290000
1	5.666501000	0.378332000	1.125790000
1	5.180804000	0.120947000	2.818638000
1	5.522391000	-2.423124000	2.499597000
1	4.673533000	-3.246285000	1.169295000
1	6.000197000	-2.119714000	0.811319000
8	5.302532000	-0.028563000	-1.727239000
6	5.146050000	1.364014000	-1.631517000
1	4.714256000	-0.438802000	-1.031133000
1	5.772292000	1.845180000	-2.393723000
1	4.105787000	1.689752000	-1.809573000
1	5.460698000	1.763157000	-0.651108000
1	2.332274000	-0.279565000	0.076140000
1	1.690724000	0.220962000	0.402296000
26	-3.641538000	0.952280000	0.496459000
77	0.671328000	0.180923000	-1.104403000
15	-0.854288000	-1.077271000	0.173209000
16	-0.217287000	2.353721000	-0.368780000
6	-2.066320000	-0.145338000	1.160777000
6	-1.928230000	1.229952000	1.573089000
6	-3.073923000	1.560103000	2.361406000
1	-3.271127000	2.538334000	2.789444000
6	-3.911047000	0.413723000	2.449490000
1	-4.875293000	0.363387000	2.943908000
6	-3.296992000	-0.634941000	1.712716000
1	-3.712569000	-1.624192000	1.552202000
6	-3.778326000	2.378660000	-0.965133000
1	-3.120601000	3.236400000	-1.055051000
6	-3.559396000	1.093426000	-1.541495000
1	-2.700605000	0.803538000	-2.139826000
6	-4.630269000	0.240397000	-1.138740000
1	-4.732224000	-0.814017000	-1.378769000
6	-5.510983000	1.001707000	-0.315673000
1	-6.399796000	0.628579000	0.181974000
6	-4.983856000	2.321897000	-0.207027000
1	-5.405051000	3.129078000	0.382629000
6	-0.782757000	2.164265000	1.381702000
1	0.095831000	1.833771000	1.955479000
1	-1.065430000	3.166734000	1.726768000
6	0.010451000	-2.059867000	1.464607000
6	0.838558000	-3.111438000	1.055871000
1	0.891597000	-3.383630000	0.001455000
6	1.613921000	-3.800445000	1.981475000
1	2.252580000	-4.616231000	1.647184000
6	1.576654000	-3.442438000	3.328867000
1	2.187517000	-3.977645000	4.052943000
6	0.755853000	-2.397934000	3.743090000
1	0.719001000	-2.116084000	4.793510000
6	-0.025262000	-1.708488000	2.816200000
1	-0.661433000	-0.890859000	3.154930000

6	-1.897938000	-2.333763000	-0.668620000
6	-2.482685000	-3.389758000	0.041399000
1	-2.277614000	-3.512651000	1.104723000
6	-3.323570000	-4.291190000	-0.602733000
1	-3.773622000	-5.106761000	-0.040665000
6	-3.582165000	-4.154221000	-1.965261000
1	-4.237057000	-4.862489000	-2.468380000
6	-2.990460000	-3.119055000	-2.682947000
1	-3.178490000	-3.015714000	-3.749530000
6	-2.150469000	-2.214416000	-2.037642000
1	-1.683106000	-1.405988000	-2.599112000
6	1.073903000	3.565576000	-0.037765000
6	0.696392000	4.908304000	0.023715000
1	-0.343820000	5.188375000	-0.139106000
6	1.653155000	5.882096000	0.291650000
1	1.359499000	6.928445000	0.337296000
6	2.982149000	5.517286000	0.493184000
1	3.729814000	6.280451000	0.697645000
6	3.354653000	4.177465000	0.428128000
1	4.393366000	3.890309000	0.583432000
6	2.402589000	3.196237000	0.162484000
1	2.689450000	2.147740000	0.100044000
1	1.789408000	0.932293000	-2.070979000

7

E (BS1) = -2515.233233

E (BS2) = -3655.652538

$G_{298.15, 1M}$ (BS2) = -3655.031749

1	3.288938000	-1.546835000	2.026214000
1	1.545930000	-0.939730000	-1.603375000
1	-0.106508000	0.347836000	-2.490639000
1	3.362975000	-2.576767000	-0.790208000
8	3.412587000	-3.270960000	-1.476505000
6	4.319027000	-2.766271000	-2.433191000
1	4.451687000	-3.520047000	-3.217463000
1	3.953546000	-1.843600000	-2.912254000
1	5.308935000	-2.555024000	-1.996922000
8	3.797106000	-1.030299000	0.092207000
6	4.180153000	-1.229030000	1.456728000
6	4.723761000	0.057335000	2.041881000
6	5.204750000	-2.339590000	1.463589000
1	3.971420000	0.856170000	2.004174000
1	5.606951000	0.384036000	1.475059000
1	5.018917000	-0.082147000	3.089092000
1	5.500349000	-2.592831000	2.488427000
1	4.809135000	-3.242940000	0.982938000
1	6.103814000	-2.024834000	0.914368000
8	5.477330000	0.618348000	-1.395950000
6	5.098082000	1.931513000	-1.050218000
1	4.925551000	0.006275000	-0.868182000
1	5.702552000	2.629310000	-1.641532000
1	4.038362000	2.136921000	-1.272816000
1	5.277962000	2.156130000	0.014364000
1	2.966171000	-0.491447000	0.052488000
1	1.712073000	0.448548000	0.376102000
26	-3.674914000	0.590041000	0.613715000
77	0.746143000	0.349059000	-1.037415000
15	-0.681973000	-1.107033000	0.099361000
16	-0.484223000	2.355284000	-0.341701000
6	-1.955752000	-0.350233000	1.169837000
6	-1.963381000	1.014114000	1.638321000
6	-3.106044000	1.176398000	2.481753000
1	-3.398643000	2.108232000	2.956447000
6	-3.802662000	-0.061829000	2.546503000
1	-4.734483000	-0.244040000	3.071408000
6	-3.102361000	-0.999180000	1.740234000
1	-3.407987000	-2.023020000	1.551854000
6	-4.033806000	2.081359000	-0.740168000
1	-3.488133000	3.018227000	-0.782031000
6	-3.676750000	0.875345000	-1.410857000
1	-2.809633000	0.735381000	-2.049423000
6	-4.621529000	-0.129838000	-1.044893000
1	-4.601353000	-1.169477000	-1.359200000
6	-5.562330000	0.458140000	-0.149446000
1	-6.382862000	-0.054999000	0.340833000
6	-5.198203000	1.823588000	0.040061000
1	-5.694860000	2.530325000	0.696321000
6	-0.968718000	2.102490000	1.421392000
1	-0.038119000	1.906866000	1.974069000
1	-1.389332000	3.054481000	1.770298000
6	0.217856000	-2.176221000	1.300919000
6	1.029372000	-3.204559000	0.808152000
1	1.057327000	-3.409253000	-0.262749000
6	1.811246000	-3.965821000	1.669850000
1	2.435720000	-4.761582000	1.266929000
6	1.798354000	-3.705014000	3.039784000
1	2.411982000	-4.298013000	3.714969000
6	0.997808000	-2.681484000	3.538109000
1	0.980591000	-2.472424000	4.605952000
6	0.211098000	-1.920119000	2.674018000
1	-0.407528000	-1.118907000	3.079256000
6	-1.666649000	-2.359105000	-0.835125000
6	-2.187030000	-3.501480000	-0.213145000
1	-1.964498000	-3.703140000	0.834298000
6	-2.983803000	-4.393104000	-0.924008000
1	-3.382813000	-5.275160000	-0.427319000
6	-3.261917000	-4.162503000	-2.270165000
1	-3.881386000	-4.863186000	-2.826167000
6	-2.732418000	-3.042269000	-2.903184000
1	-2.933691000	-2.863084000	-3.957414000
6	-1.938132000	-2.147885000	-2.188650000
1	-1.517003000	-1.272285000	-2.682817000
6	0.585685000	3.778433000	-0.058224000
6	0.023628000	5.047880000	-0.198593000
1	-1.025725000	5.148889000	-0.473834000
6	0.809651000	6.177732000	0.010662000
1	0.373063000	7.167492000	-0.103620000
6	2.150158000	6.039349000	0.360780000
1	2.764162000	6.923258000	0.519298000
6	2.705573000	4.770228000	0.506595000
1	3.753309000	4.659794000	0.780048000
6	1.925771000	3.636339000	0.297992000
1	2.349926000	2.638136000	0.398595000
1	1.885976000	1.245864000	-1.846470000

B2. Lithium system

1-Li

E (BS1) = -2675.520372

E (BS2) = -3815.993868

G.15,1M (BS2) = -3815.32453

3	3.978696000	-0.757671000	0.055363000
1	2.920390000	-2.805246000	1.292530000
8	3.617123000	-2.164562000	1.498058000
6	4.336187000	-2.602276000	2.633917000
1	5.222408000	-1.966039000	2.731747000

1	3.744029000	-2.518578000	3.557110000
1	4.673150000	-3.642282000	2.527475000
1	2.932519000	0.577635000	-1.660258000
1	2.103477000	-1.584381000	-1.202101000
8	2.904681000	-2.156504000	-1.192341000
8	3.910624000	0.517456000	-1.574016000
6	3.151088000	-2.558939000	-2.525682000
1	3.994310000	-3.258110000	-2.521349000
1	2.284575000	-3.080415000	-2.957639000
1	3.405220000	-1.709374000	-3.177232000
6	4.435684000	1.829974000	-1.639337000
1	5.526196000	1.762897000	-1.556027000
1	4.195544000	2.312846000	-2.597394000
1	4.068118000	2.473569000	-0.825212000
26	-3.727184000	1.056229000	0.902219000
77	0.297986000	0.280529000	-1.289050000
15	-1.162414000	-1.071401000	-0.065901000
16	-0.371109000	2.351933000	-0.134268000
6	-2.182569000	-0.235412000	1.197212000
6	-1.890038000	1.043944000	1.794674000
6	-2.910517000	1.318384000	2.756096000
1	-2.977901000	2.226727000	3.347161000
6	-3.825436000	0.229895000	2.768359000
1	-4.732793000	0.164358000	3.359311000
6	-3.383088000	-0.724944000	1.813111000
1	-3.897525000	-1.644738000	1.556329000
6	-3.935236000	2.730294000	-0.257434000
1	-3.248134000	3.569499000	-0.261020000
6	-3.839138000	1.566258000	-1.074698000
1	-3.057908000	1.363532000	-1.801973000
6	-4.907128000	0.688761000	-0.719854000
1	-5.085875000	-0.299975000	-1.132374000
6	-5.662858000	1.313831000	0.314881000
1	-6.515516000	0.883770000	0.829597000
6	-5.061246000	2.573882000	0.602023000
1	-5.379847000	3.271753000	1.368939000
6	-0.688045000	1.909444000	1.615486000
1	0.204412000	1.390528000	1.992732000
1	-0.820759000	2.844417000	2.173914000
6	-0.294746000	-2.342056000	0.950450000
6	0.250549000	-3.467507000	0.323359000
1	0.092928000	-3.626638000	-0.744505000
6	0.991846000	-4.394126000	1.051892000
1	1.403016000	-5.269439000	0.553125000
6	1.207590000	-4.199939000	2.416650000
1	1.788933000	-4.922560000	2.986127000
6	0.677876000	-3.075488000	3.045704000
1	0.842954000	-2.916443000	4.109626000
6	-0.070774000	-2.153744000	2.317410000
1	-0.481704000	-1.279624000	2.823217000
6	-2.408454000	-2.099260000	-0.956169000
6	-3.039658000	-3.185664000	-0.336065000
1	-2.758200000	-3.477313000	0.675447000
6	-4.025637000	-3.905017000	-1.002857000
1	-4.509843000	-4.744192000	-0.507716000
6	-4.385852000	-3.557151000	-2.303595000
1	-5.154945000	-4.122914000	-2.825328000
6	-3.750109000	-2.493558000	-2.936175000
1	-4.017123000	-2.224923000	-3.956241000
6	-2.766040000	-1.769901000	-2.265569000
1	-2.265592000	-0.936745000	-2.758684000
6	1.084008000	3.391512000	0.032978000
6	1.447043000	4.120597000	-1.103936000
1	0.853347000	4.039354000	-2.014084000
6	2.565480000	4.943542000	-1.073889000
1	2.838913000	5.506939000	-1.963512000
6	3.330562000	5.050375000	0.087142000
1	4.205419000	5.695888000	0.108853000
6	2.962526000	4.331808000	1.218631000
1	3.547025000	4.411662000	2.133349000
6	1.838552000	3.503976000	1.200706000
1	1.569129000	2.963403000	2.105357000
1	0.688716000	-1.017445000	-2.175624000
1	1.389145000	1.143050000	-2.210410000
1	1.511605000	0.012577000	-0.110452000
1	-0.809263000	0.627312000	-2.513895000
8	3.854344000	0.854232000	1.380228000
6	3.434503000	0.687095000	2.726135000
1	3.296859000	1.656472000	3.224351000
1	2.500339000	0.109849000	2.799043000
1	4.223515000	0.144708000	3.255117000
1	3.203345000	1.420355000	0.936206000
8	5.991168000	-1.025904000	0.036496000
6	6.322293000	-2.185719000	-0.718592000
1	7.404513000	-2.363907000	-0.724481000
1	5.833782000	-3.039055000	-0.236829000
1	5.968479000	-2.106995000	-1.755803000
1	6.397559000	-0.260331000	-0.391833000

2-Li

E (BS1) = -2868.575683

E (BS2) = -4009.124187

$G_{298.15,1M}$ (BS2) = -4008.374913

3	-3.631664000	1.232405000	-0.326961000
1	-2.041906000	2.103449000	1.488494000
8	-2.857795000	2.536036000	1.183457000
6	-3.634694000	2.794129000	2.338503000
1	-4.499437000	3.395906000	2.039226000
1	-3.997067000	1.868978000	2.811575000
1	-3.066617000	3.366099000	3.086670000
1	-2.925759000	-0.249011000	-2.056847000
1	-1.825678000	1.858101000	-1.729820000
8	-2.618850000	2.438152000	-1.689512000
8	-3.890209000	-0.130456000	-1.913984000
6	-2.994904000	2.759723000	-3.014037000
1	-3.967125000	3.262887000	-2.976118000
1	-2.271546000	3.443732000	-3.482667000
1	-3.091731000	1.866640000	-3.647515000
6	-4.461875000	-1.421310000	-1.804801000
1	-5.533284000	-1.302735000	-1.606998000
1	-4.352451000	-1.990045000	-2.739417000
1	-4.014950000	-1.999949000	-0.983139000
26	3.371768000	-1.325033000	1.201179000
77	-0.191781000	-0.160760000	-1.755705000
15	1.390640000	0.998691000	-0.496363000
16	0.169714000	-2.262118000	-0.524003000
6	1.984195000	0.159584000	1.014949000
6	1.404284000	-1.008501000	1.632716000
6	2.143066000	-1.277747000	2.827253000
1	1.967345000	-2.122480000	3.487176000
6	3.169163000	-0.301186000	2.958527000
1	3.924499000	-0.268704000	3.736582000
6	3.079503000	0.576580000	1.845040000
1	3.746619000	1.406551000	1.636407000
6	3.572669000	-3.136352000	0.269525000
1	2.778689000	-3.868358000	0.166540000
6	3.856642000	-2.076201000	-0.639351000

1	3.318380000	-1.863616000	-1.557629000
6	4.940323000	-1.315712000	-0.107135000
1	5.369384000	-0.420699000	-0.547675000
6	5.325311000	-1.908768000	1.130550000
1	6.093557000	-1.540741000	1.802327000
6	4.479382000	-3.032362000	1.364043000
1	4.493988000	-3.668777000	2.242457000
6	0.208951000	-1.815658000	1.248646000
1	-0.713324000	-1.258457000	1.464473000
1	0.213842000	-2.749926000	1.825761000
6	0.741685000	2.588959000	0.179321000
6	0.349896000	3.578654000	-0.730936000
1	0.467108000	3.406639000	-1.802275000
6	-0.193942000	4.777731000	-0.285576000
1	-0.493932000	5.535183000	-1.007261000
6	-0.360716000	5.005402000	1.080103000
1	-0.789921000	5.942067000	1.429636000
6	0.028973000	4.030847000	1.992534000
1	-0.088287000	4.203811000	3.061608000
6	0.581064000	2.830480000	1.545432000
1	0.882448000	2.079186000	2.273857000
6	2.968608000	1.579981000	-1.263872000
6	3.703716000	2.638883000	-0.715549000
1	3.324410000	3.175355000	0.153785000
6	4.917690000	3.022538000	-1.276407000
1	5.477851000	3.846623000	-0.839381000
6	5.408893000	2.360866000	-2.400223000
1	6.355401000	2.665721000	-2.841720000
6	4.677579000	1.319431000	-2.963770000
1	5.048260000	0.806951000	-3.849211000
6	3.463450000	0.934309000	-2.399221000
1	2.884363000	0.125377000	-2.845034000
6	-1.313535000	-3.274024000	-0.586705000
6	-1.549562000	-3.928697000	-1.800383000
1	-0.867368000	-3.783062000	-2.637666000
6	-2.652072000	-4.761458000	-1.939302000
1	-2.825444000	-5.266474000	-2.887159000
6	-3.526526000	-4.954652000	-0.870409000
1	-4.387540000	-5.610066000	-0.978795000
6	-3.286530000	-4.307352000	0.335687000
1	-3.961973000	-4.450668000	1.177208000
6	-2.183134000	-3.466245000	0.486092000
1	-2.027799000	-2.970362000	1.441292000
1	-0.407470000	1.172848000	-2.650894000
1	-1.393902000	-0.855943000	-2.681706000
1	-1.334082000	0.327298000	-0.584402000
1	0.861059000	-0.679269000	-2.977132000
8	-3.558871000	-0.359561000	1.123315000
6	-4.407786000	-1.070562000	2.000546000
1	-3.928221000	-1.986724000	2.378353000
1	-4.714465000	-0.463651000	2.866994000
1	-5.307860000	-1.366524000	1.449607000
1	-2.758457000	-0.114837000	1.629582000
8	-5.489555000	1.991442000	-0.339911000
6	-6.495037000	1.274181000	0.362671000
1	-7.493280000	1.679313000	0.156702000
1	-6.463901000	0.238445000	0.008867000
1	-6.324998000	1.284672000	1.447925000
1	-5.506078000	2.913094000	-0.049864000
6	-1.164209000	0.249473000	3.838536000
8	-1.492856000	0.623191000	2.714028000
6	-1.507771000	-1.114603000	4.333399000
6	-0.400038000	1.143839000	4.754397000
1	-1.832825000	-1.763495000	3.513555000
1	-0.448438000	2.182891000	4.415536000
1	-2.326090000	-1.022566000	5.061058000
1	-0.662413000	-1.562840000	4.868618000
1	0.649986000	0.816208000	4.758603000
1	-0.761608000	1.055997000	5.784922000

$TS_{2\text{-}3}$-Li

E (BS1) = -2868.554123

E (BS2) = -4009.099031

$G_{298.15,1M}$ (BS2) = -4008.34938

3	4.847123000	-0.727368000	-0.864669000
1	4.453911000	-1.735287000	1.208656000
8	5.284308000	-2.089477000	0.812152000
6	6.318875000	-1.854306000	1.745516000
1	7.282615000	-1.970658000	1.236725000
1	6.272026000	-0.839076000	2.167309000
1	6.287241000	-2.571624000	2.578603000
1	3.132773000	0.288112000	-2.270456000
1	2.432838000	-1.578054000	-0.951219000
8	3.292273000	-1.992464000	-1.183618000
8	4.070922000	0.444819000	-2.480710000
6	3.147709000	-2.599637000	-2.456042000
1	4.146366000	-2.878369000	-2.811568000
1	2.534828000	-3.509917000	-2.401541000
1	2.695481000	-1.917960000	-3.190282000
6	4.247163000	1.848849000	-2.577184000
1	5.317883000	2.049360000	-2.691544000
1	3.727083000	2.258844000	-3.453455000
1	3.886219000	2.369042000	-1.677733000
26	-4.113150000	1.516188000	0.059996000
77	0.261880000	-0.202166000	-0.605556000
15	-1.792505000	-1.089695000	0.086146000
16	-0.393938000	2.090912000	-0.046668000
6	-2.959915000	0.059766000	0.894610000
6	-2.638307000	1.347432000	1.458880000
6	-3.830196000	1.872606000	2.047250000
1	-3.911411000	2.847193000	2.519096000
6	-4.880713000	0.932101000	1.862374000
1	-5.916067000	1.064386000	2.158026000
6	-4.352689000	-0.178358000	1.151215000
1	-4.917833000	-1.043689000	0.821918000
6	-3.675497000	3.067968000	-1.198855000
1	-2.875397000	3.782855000	-1.036891000
6	-3.559710000	1.823841000	-1.884478000
1	-2.656096000	1.428329000	-2.337980000
6	-4.823850000	1.164671000	-1.820738000
1	-5.050110000	0.178678000	-2.215874000
6	-5.719936000	2.004790000	-1.097449000
1	-6.747540000	1.769718000	-0.841156000
6	-5.010262000	3.179694000	-0.711976000
1	-5.404641000	3.994465000	-0.114145000
6	-1.328348000	2.052428000	1.543154000
1	-0.669799000	1.586695000	2.289469000
1	-1.499274000	3.096911000	1.832707000
6	-1.657747000	-2.416772000	1.354995000
6	-1.206634000	-3.685498000	0.971226000
1	-0.996001000	-3.893930000	-0.078412000
6	-1.027831000	-4.687833000	1.918052000
1	-0.686081000	-5.672117000	1.604435000
6	-1.283061000	-4.431889000	3.264551000
1	-1.142538000	-5.215991000	4.005451000
6	-1.716548000	-3.169343000	3.657052000
1	-1.916733000	-2.961527000	4.706265000

6	-1.906531000	-2.166739000	2.707142000
1	-2.251896000	-1.183576000	3.028056000
6	-2.867477000	-1.907554000	-1.174733000
6	-3.876614000	-2.804533000	-0.799445000
1	-4.000329000	-3.084700000	0.245938000
6	-4.724919000	-3.356631000	-1.753569000
1	-5.504261000	-4.050300000	-1.444863000
6	-4.571158000	-3.030209000	-3.099608000
1	-5.232168000	-3.466460000	-3.845549000
6	-3.560820000	-2.155258000	-3.485781000
1	-3.425156000	-1.904922000	-4.535973000
6	-2.714429000	-1.599389000	-2.528655000
1	-1.921189000	-0.917757000	-2.832982000
6	0.949811000	3.154227000	0.507444000
6	0.685912000	4.522395000	0.609801000
1	-0.303741000	4.905830000	0.361747000
6	1.689872000	5.390629000	1.023739000
1	1.483021000	6.455566000	1.103368000
6	2.957925000	4.897197000	1.323377000
1	3.744683000	5.577638000	1.641605000
6	3.219752000	3.535452000	1.207773000
1	4.210228000	3.146528000	1.436176000
6	2.217295000	2.656431000	0.800226000
1	2.418192000	1.589481000	0.705664000
1	0.620901000	-1.694129000	-1.138531000
1	1.722002000	0.352152000	-1.189340000
1	1.100303000	-0.558525000	0.945297000
1	-0.303739000	0.096110000	-2.119398000
8	5.033616000	0.904613000	0.307687000
6	6.296522000	1.508081000	0.522497000
1	6.387173000	1.912339000	1.537750000
1	7.119585000	0.796207000	0.360253000
1	6.415578000	2.338724000	-0.184660000
1	4.615343000	0.619270000	1.139830000
8	6.592618000	-1.221796000	-1.693406000
6	7.068252000	-0.573746000	-2.865519000
1	6.435460000	-0.788508000	-3.736470000
1	7.051176000	0.503305000	-2.670450000
1	8.100382000	-0.867212000	-3.091462000
1	6.648724000	-2.178353000	-1.820143000
6	2.161211000	-0.898413000	2.174713000
8	3.317938000	-0.545793000	1.795477000
6	1.465271000	-0.049833000	3.209098000
6	1.866481000	-2.374211000	2.235869000
1	1.587789000	1.016799000	2.990955000
1	2.184015000	-2.877004000	1.315663000
1	1.950013000	-0.253302000	4.176598000
1	0.401691000	-0.296472000	3.303765000
1	0.810207000	-2.579569000	2.427848000
1	2.456053000	-2.786401000	3.069525000

3-Li

E (BS1) = -2868.577748

E (BS2) = -4009.125035

$G_{298.15,1M}$ (BS2) = -4008.372533

3	-4.373109000	-0.325013000	0.726007000
1	-4.836655000	-1.911420000	-1.043565000
8	-5.535581000	-2.622599000	-1.036263000
6	-5.808520000	-2.931460000	-2.381069000
1	-6.830212000	-3.323216000	-2.468058000
1	-5.734191000	-2.043613000	-3.030427000
1	-5.126384000	-3.696961000	-2.788757000
1	-2.949109000	1.257246000	1.978424000
1	-2.289154000	-1.308593000	1.589856000
8	-3.206673000	-1.585609000	1.795713000
8	-3.879692000	1.328112000	1.702670000
6	-3.330824000	-1.617435000	3.208072000
1	-4.346156000	-1.950781000	3.447410000
1	-2.621706000	-2.325107000	3.658139000
1	-3.173905000	-0.626769000	3.659007000
6	-4.354358000	2.640845000	1.947054000
1	-5.246542000	2.796730000	1.331136000
1	-4.623792000	2.782728000	3.002590000
1	-3.608396000	3.397326000	1.668392000
26	4.129824000	1.323604000	-0.809885000
77	-0.080826000	-0.059900000	1.044341000
15	1.793456000	-1.116974000	0.078664000
16	0.573733000	2.134405000	0.130059000
6	2.762002000	-0.153907000	-1.125261000
6	2.352629000	1.069091000	-1.771097000
6	3.376832000	1.431058000	-2.699584000
1	3.370915000	2.328952000	-3.309978000
6	4.409479000	0.454595000	-2.639980000
1	5.342271000	0.477847000	-3.193158000
6	4.040777000	-0.514646000	-1.669671000
1	4.638438000	-1.368388000	-1.366996000
6	4.060385000	3.060020000	0.269436000
1	3.262507000	3.793008000	0.205782000
6	4.096062000	1.934416000	1.142525000
1	3.332933000	1.663811000	1.865257000
6	5.287684000	1.198607000	0.868462000
1	5.586137000	0.268442000	1.342905000
6	5.988382000	1.873286000	-0.172966000
1	6.911172000	1.543555000	-0.638261000
6	5.229820000	3.021933000	-0.544164000
1	5.476059000	3.717786000	-1.339066000
6	1.093767000	1.850712000	-1.623690000
1	0.250357000	1.354792000	-2.125470000
1	1.225093000	2.845121000	-2.069128000
6	1.367054000	-2.623952000	-0.881990000
6	0.918448000	-3.755141000	-0.188134000
1	0.880212000	-3.745580000	0.901884000
6	0.529407000	-4.896799000	-0.878381000
1	0.191921000	-5.772283000	-0.327590000
6	0.570975000	-4.918701000	-2.271984000
1	0.265656000	-5.811997000	-2.812493000
6	1.002118000	-3.794613000	-2.968889000
1	1.034925000	-3.805312000	-4.056345000
6	1.400974000	-2.650829000	-2.278372000
1	1.740982000	-1.778891000	-2.836790000
6	3.090102000	-1.743835000	1.226585000
6	3.971660000	-2.758640000	0.833440000
1	3.855795000	-3.239942000	-0.137066000
6	4.998851000	-3.168510000	1.677405000
1	5.676038000	-3.957795000	1.358136000
6	5.155184000	-2.576445000	2.928941000
1	5.957021000	-2.900054000	3.589085000
6	4.275551000	-1.577801000	3.335340000
1	4.384023000	-1.116627000	4.314725000
6	3.248398000	-1.166495000	2.489591000
1	2.564901000	-0.384716000	2.816287000
6	-0.844677000	3.174377000	-0.250260000
6	-0.702782000	4.555137000	-0.114632000
1	0.233266000	4.972850000	0.253585000
6	-1.761196000	5.391130000	-0.463011000
1	-1.655054000	6.468188000	-0.354828000
6	-2.944357000	4.849823000	-0.958106000

1	-3.766204000	5.505443000	-1.237296000
6	-3.080877000	3.469246000	-1.090919000
1	-4.011600000	3.039695000	-1.458601000
6	-2.035158000	2.626935000	-0.726377000
1	-2.144241000	1.546524000	-0.807593000
1	-0.416579000	-1.459342000	1.791989000
1	-1.335333000	0.617572000	1.909843000
1	-1.751302000	-0.538185000	-0.710782000
1	0.777761000	0.306617000	2.281023000
8	-5.630685000	1.216029000	-0.918244000
6	-6.774423000	0.625734000	-1.500444000
1	-6.795304000	0.755059000	-2.593845000
1	-6.840152000	-0.449928000	-1.279416000
1	-7.666758000	1.113096000	-1.090573000
1	-4.851238000	0.669009000	-1.190446000
8	-6.162574000	-0.915161000	1.454174000
6	-6.769747000	-0.054553000	2.409205000
1	-6.129468000	0.100031000	3.289161000
1	-6.936234000	0.909916000	1.918981000
1	-7.739722000	-0.446350000	2.737668000
1	-6.091226000	-1.800654000	1.834905000
6	-2.509480000	-0.981459000	-1.432328000
8	-3.791427000	-0.637710000	-1.047208000
6	-2.169788000	-0.428096000	-2.811848000
6	-2.290220000	-2.489373000	-1.412730000
1	-2.322753000	0.658821000	-2.855703000
1	-2.484444000	-2.908573000	-0.416768000
1	-2.831233000	-0.886169000	-3.562626000
1	-1.129411000	-0.645723000	-3.096477000
1	-1.264120000	-2.744769000	-1.704771000
1	-2.971579000	-2.974719000	-2.127206000

4-Li

E (BS1) = -2868.588704
E (BS2) = -4009.133252
$G_{298.15,1M}$ (BS2) = -4008.375846

1	0.357622000	0.544067000	2.635749000
1	1.424021000	-1.017883000	-1.030076000
1	-0.121002000	0.196341000	-2.086369000
3	4.198294000	-0.874101000	-0.802214000
1	2.735257000	-1.055743000	1.152807000
8	3.355742000	-1.784069000	0.841028000
6	4.281024000	-2.150640000	1.843146000
1	4.934391000	-2.930976000	1.434824000
1	4.911437000	-1.303465000	2.153774000
1	3.782586000	-2.559139000	2.734342000
1	2.709283000	0.095778000	-2.279411000
8	3.547903000	-0.291503000	-2.616227000
6	4.297985000	0.727039000	-3.260465000
1	5.068110000	0.246732000	-3.872851000
1	3.663219000	1.333598000	-3.918895000
1	4.792773000	1.395244000	-2.540666000
8	1.880280000	0.350625000	1.259604000
6	1.450871000	0.724300000	2.540499000
6	1.709209000	2.199042000	2.811220000
6	2.145926000	-0.121774000	3.597574000
1	1.201861000	2.837259000	2.077904000
1	2.789320000	2.406700000	2.748055000
1	1.365703000	2.493417000	3.812433000
1	1.801504000	0.133152000	4.609087000
1	1.955513000	-1.191317000	3.437510000
1	3.233203000	0.047272000	3.558191000
8	4.265907000	0.866434000	0.325034000
6	4.549786000	2.215001000	0.028026000
1	3.351644000	0.806432000	0.738570000
1	5.618174000	2.312257000	-0.201813000
1	3.979515000	2.581742000	-0.841598000
1	4.333007000	2.872618000	0.883668000
26	-4.148134000	0.436075000	0.033601000
77	0.570371000	0.297191000	-0.663135000
15	-1.045677000	-1.138210000	0.258432000
16	-0.894188000	2.278200000	-0.393663000
6	-2.532289000	-0.363343000	0.983257000
6	-2.700406000	1.025769000	1.340969000
6	-3.995889000	1.165124000	1.929779000
1	-4.424556000	2.104389000	2.266065000
6	-4.630112000	-0.107461000	1.945113000
1	-5.638140000	-0.315218000	2.287904000
6	-3.739250000	-1.045256000	1.359157000
1	-3.947265000	-2.096788000	1.191547000
6	-4.303703000	1.829583000	-1.455862000
1	-3.806043000	2.793953000	-1.450399000
6	-3.764676000	0.613159000	-1.966752000
1	-2.784086000	0.490427000	-2.416435000
6	-4.713665000	-0.425520000	-1.728510000
1	-4.583759000	-1.476430000	-1.969089000
6	-5.839246000	0.151839000	-1.071496000
1	-6.714660000	-0.382642000	-0.718088000
6	-5.585344000	1.544523000	-0.901897000
1	-6.234389000	2.253623000	-0.399179000
6	-1.759410000	2.179140000	1.232976000
1	-0.989086000	2.146294000	2.015362000
1	-2.329336000	3.108468000	1.351658000
6	-0.451912000	-2.193630000	1.646395000
6	0.496622000	-3.182840000	1.360890000
1	0.822014000	-3.340746000	0.331356000
6	1.025689000	-3.971686000	2.376072000
1	1.761411000	-4.737713000	2.137060000
6	0.615690000	-3.778516000	3.694739000
1	1.028950000	-4.394945000	4.490281000
6	-0.325553000	-2.796598000	3.988551000
1	-0.652044000	-2.642851000	5.015158000
6	-0.858519000	-2.007942000	2.969961000
1	-1.597310000	-1.245377000	3.214794000
6	-1.767322000	-2.416435000	-0.864193000
6	-2.383835000	-3.566905000	-0.354435000
1	-2.399448000	-3.750163000	0.719363000
6	-2.972876000	-4.493765000	-1.208511000
1	-3.447556000	-5.381380000	-0.795457000
6	-2.946213000	-4.291547000	-2.586924000
1	-3.402258000	-5.019668000	-3.254350000
6	-2.321868000	-3.161506000	-3.105571000
1	-2.286099000	-3.000364000	-4.181060000
6	-1.735633000	-2.232119000	-2.249277000
1	-1.244233000	-1.352104000	-2.660846000
6	-0.074388000	3.867326000	-0.178701000
6	-0.857168000	5.017215000	-0.048099000
1	-1.944619000	4.945888000	-0.047872000
6	-0.242232000	6.258589000	0.073224000
1	-0.852661000	7.152414000	0.181895000
6	1.147395000	6.359858000	0.040594000
1	1.623836000	7.333621000	0.129655000
6	1.922912000	5.214658000	-0.111126000
1	3.008761000	5.287612000	-0.141094000
6	1.314326000	3.966572000	-0.217359000
1	1.906955000	3.060000000	-0.326271000
1	1.716655000	1.224654000	-1.449927000

8	6.119216000	-1.306134000	-0.976176000
6	7.091166000	-0.285681000	-0.779066000
1	8.102855000	-0.705511000	-0.729642000
1	7.057656000	0.468758000	-1.576697000
1	6.868287000	0.196676000	0.176976000
1	6.321022000	-1.773696000	-1.797848000
8	3.574360000	-3.135374000	-1.467995000
6	2.544061000	-3.479375000	-2.374563000
1	2.083252000	-4.445785000	-2.124034000
1	1.752794000	-2.714736000	-2.414254000
1	2.986788000	-3.563766000	-3.372587000
1	3.185146000	-2.965455000	-0.590787000

5-Li

E (BS1) = -2869.762117

E (BS2) = -4010.308917

$G_{298.15,\ 1M}$ (BS2) = -4009.540769

1	0.340204000	0.562124000	2.580846000
1	1.431508000	-1.035131000	-1.037299000
1	-0.111177000	0.169894000	-2.108260000
3	4.197506000	-0.901796000	-0.804221000
1	2.736586000	-1.035696000	1.153118000
8	3.358531000	-1.769921000	0.856097000
6	4.280964000	-2.115929000	1.867940000
1	4.941566000	-2.898100000	1.474937000
1	4.904539000	-1.260105000	2.168493000
1	3.780332000	-2.513324000	2.762997000
1	2.709731000	0.054502000	-2.289638000
8	3.549604000	-0.333408000	-2.622968000
6	4.296415000	0.683082000	-3.273874000
1	5.069839000	0.201609000	-3.881146000
1	3.660252000	1.281508000	-3.938431000
1	4.786542000	1.359269000	-2.558375000
8	1.885523000	0.365053000	1.237567000
6	1.433078000	0.751271000	2.506355000
6	1.673932000	2.231111000	2.764670000
6	2.113102000	-0.075780000	3.587861000
1	1.162262000	2.858206000	2.025098000
1	2.752025000	2.449618000	2.702446000
1	1.326511000	2.531854000	3.763046000
1	1.738406000	0.182160000	4.588499000
1	1.942294000	-1.149169000	3.432280000
1	3.198592000	0.108703000	3.576113000
8	4.276188000	0.869430000	0.300763000
6	4.566202000	2.215039000	-0.003598000
1	3.362643000	0.816604000	0.714708000
1	5.635319000	2.306021000	-0.232582000
1	3.999078000	2.579784000	-0.876109000
1	4.351147000	2.878467000	0.847958000
1	-0.949673000	0.961690000	4.290320000
1	-1.533648000	1.257811000	4.648944000
26	-4.141575000	0.427569000	-0.022651000
77	0.579302000	0.285347000	-0.685439000
15	-1.039379000	-1.142504000	0.246957000
16	-0.883813000	2.269973000	-0.434415000
6	-2.534364000	-0.361838000	0.946783000
6	-2.705673000	1.029947000	1.291406000
6	-4.005099000	1.173483000	1.870135000
1	-4.436434000	2.115255000	2.195917000
6	-4.639296000	-0.099110000	1.890491000
1	-5.650039000	-0.304285000	2.226803000
6	-3.743785000	-1.041402000	1.318871000
1	-3.950117000	-2.094292000	1.157522000
6	-4.280340000	1.802352000	-1.530989000
1	-3.781197000	2.765970000	-1.532727000
6	-3.737848000	0.578677000	-2.020630000
1	-2.752587000	0.448653000	-2.457864000
6	-4.690668000	-0.455629000	-1.778717000
1	-4.560047000	-1.509809000	-2.004066000
6	-5.822096000	0.131665000	-1.140651000
1	-6.701804000	-0.397167000	-0.789420000
6	-5.567965000	1.526103000	-0.986418000
1	-6.221336000	2.242533000	-0.500006000
6	-1.761354000	2.180597000	1.186441000
1	-0.997574000	2.147195000	1.975659000
1	-2.329494000	3.111920000	1.297427000
6	-0.457866000	-2.168528000	1.661472000
6	0.500925000	-3.157248000	1.411416000
1	0.837873000	-3.339128000	0.389507000
6	1.024472000	-3.915208000	2.452895000
1	1.768202000	-4.681814000	2.242242000
6	0.600251000	-3.689422000	3.762009000
1	1.011669000	-4.279842000	4.578004000
6	-0.352507000	-2.708441000	4.020202000
1	-0.689948000	-2.528014000	5.039130000
6	-0.882021000	-1.953342000	2.975013000
1	-1.632086000	-1.192907000	3.190451000
6	-1.745562000	-2.442083000	-0.860056000
6	-2.358172000	-3.588364000	-0.336544000
1	-2.381139000	-3.754018000	0.740020000
6	-2.934162000	-4.532908000	-1.180136000
1	-3.405968000	-5.417093000	-0.756544000
6	-2.898094000	-4.352525000	-2.561308000
1	-3.343738000	-5.094481000	-3.220480000
6	-2.277844000	-3.226379000	-3.093306000
1	-2.234848000	-3.082466000	-4.170959000
6	-1.704801000	-2.279246000	-2.247657000
1	-1.216202000	-1.402496000	-2.669485000
6	-0.065032000	3.860688000	-0.227952000
6	-0.847911000	5.010853000	-0.100797000
1	-1.935336000	4.939383000	-0.098433000
6	-0.233152000	6.252942000	0.014291000
1	-0.843756000	7.146929000	0.120622000
6	1.156321000	6.354632000	-0.021501000
1	1.632588000	7.328868000	0.063080000
6	1.931905000	5.209090000	-0.170633000
1	3.017669000	5.282242000	-0.202872000
6	1.323636000	3.960413000	-0.270449000
1	1.916620000	3.053560000	-0.375488000
1	1.725135000	1.206588000	-1.478684000
8	6.118061000	-1.331733000	-0.976902000
6	7.090635000	-0.309854000	-0.790695000
1	8.102803000	-0.728943000	-0.745645000
1	7.051796000	0.440772000	-1.591696000
1	6.873675000	0.177326000	0.164217000
1	6.317363000	-1.805385000	-1.795721000
8	3.572479000	-3.162928000	-1.430451000
6	2.539058000	-3.523414000	-2.327369000
1	2.074534000	-4.481682000	-2.053596000
1	1.751024000	-2.756470000	-2.383248000
1	2.980230000	-3.632221000	-3.323674000
1	3.186695000	-2.981870000	-0.553966000

$TS_{5\text{-}6}$-Li

E (BS1) = -2869.752225

E (BS2) = -4010.300751

$G_{298.15,\ 1M}$ (BS2) = -4009.535942

1	2.219706000	0.612498000	3.405429000
1	1.241574000	-1.041977000	-0.727453000
1	-0.377220000	-0.087553000	-1.888401000
3	4.063635000	-0.957860000	-1.295903000
1	3.259333000	-1.079654000	0.962970000
8	3.601644000	-1.872488000	0.416817000
6	4.724398000	-2.482995000	1.026215000
1	5.025030000	-3.341530000	0.414156000
1	5.580264000	-1.794478000	1.098699000
1	4.486554000	-2.854597000	2.032295000
1	2.104685000	-0.327186000	-2.413526000
8	2.865830000	-0.739743000	-2.878057000
6	3.353447000	0.175273000	-3.847497000
1	4.166811000	-0.314084000	-4.393078000
1	2.572621000	0.448024000	-4.568825000
1	3.741513000	1.096377000	-3.388165000
8	2.755355000	0.344870000	1.429711000
6	3.081890000	0.775633000	2.718236000
6	3.401070000	2.265308000	2.738578000
6	4.254396000	-0.012058000	3.288066000
1	2.556563000	2.857511000	2.363005000
1	4.272084000	2.470768000	2.096267000
1	3.638790000	2.620015000	3.750716000
1	4.513540000	0.318241000	4.303104000
1	4.020086000	-1.083887000	3.334105000
1	5.142946000	0.117344000	2.650995000
8	4.510088000	0.763050000	-0.413026000
6	4.533048000	2.078335000	-0.921379000
1	3.842179000	0.714524000	0.354417000
1	5.311738000	2.151538000	-1.690469000
1	3.569666000	2.366537000	-1.374214000
1	4.774494000	2.811663000	-0.136527000
1	0.332815000	1.530943000	2.219597000
1	0.268608000	1.935406000	2.846581000
26	-4.278937000	0.294664000	-0.382753000
77	0.368826000	0.302340000	-0.581641000
15	-1.182019000	-0.944538000	0.639060000
16	-1.067169000	2.312265000	-0.653381000
6	-2.802557000	-0.180230000	0.943540000
6	-3.098442000	1.231462000	0.982497000
6	-4.478618000	1.369610000	1.332160000
1	-5.006865000	2.315360000	1.407079000
6	-5.038352000	0.072853000	1.502736000
1	-6.077126000	-0.149762000	1.722359000
6	-4.014105000	-0.880993000	1.259119000
1	-4.128209000	-1.960599000	1.273655000
6	-4.218139000	1.371833000	-2.118557000
1	-3.732601000	2.336469000	-2.225276000
6	-3.601871000	0.100035000	-2.303513000
1	-2.563211000	-0.068320000	-2.572381000
6	-4.569086000	-0.907254000	-2.010296000
1	-4.399860000	-1.979297000	-2.025488000
6	-5.783708000	-0.255844000	-1.646795000
1	-6.698930000	-0.744326000	-1.329420000
6	-5.566681000	1.151800000	-1.712677000
1	-6.287474000	1.920219000	-1.454282000
6	-2.200057000	2.408524000	0.808808000
1	-1.566084000	2.558398000	1.691594000
1	-2.809577000	3.309639000	0.667692000
6	-0.578865000	-1.316270000	2.334098000
6	0.625441000	-2.022892000	2.454836000
1	1.130369000	-2.392736000	1.561063000
6	1.181455000	-2.259641000	3.706830000
1	2.113381000	-2.817475000	3.788094000
6	0.549414000	-1.778376000	4.853657000
1	0.988681000	-1.955337000	5.833278000
6	-0.644355000	-1.072383000	4.741251000
1	-1.143614000	-0.698353000	5.632856000
6	-1.210374000	-0.845641000	3.487258000
1	-2.145442000	-0.290148000	3.413113000
6	-1.639249000	-2.605603000	-0.003586000
6	-1.965263000	-3.660911000	0.855940000
1	-1.948121000	-3.511254000	1.935073000
6	-2.312000000	-4.907750000	0.342848000
1	-2.558989000	-5.720916000	1.022274000
6	-2.339421000	-5.113857000	-1.034364000
1	-2.605371000	-6.090396000	-1.433559000
6	-2.021302000	-4.068712000	-1.898248000
1	-2.033597000	-4.224985000	-2.975150000
6	-1.670803000	-2.822912000	-1.384563000
1	-1.406091000	-2.013410000	-2.064420000
6	-0.140144000	3.820604000	-0.332905000
6	-0.763880000	5.050382000	-0.551652000
1	-1.795471000	5.085981000	-0.899986000
6	-0.058425000	6.228549000	-0.329882000
1	-0.544885000	7.186881000	-0.498093000
6	1.267510000	6.180780000	0.095504000
1	1.818129000	7.103912000	0.262121000
6	1.887741000	4.952335000	0.304841000
1	2.923093000	4.910049000	0.639772000
6	1.185453000	3.768028000	0.095191000
1	1.663707000	2.801325000	0.257843000
1	1.500549000	1.122626000	-1.507317000
8	5.669624000	-1.720349000	-2.112369000
6	6.754462000	-0.827879000	-2.349133000
1	7.630272000	-1.363858000	-2.732420000
1	6.481810000	-0.034349000	-3.057932000
1	7.017124000	-0.372386000	-1.389919000
1	5.450220000	-2.168007000	-2.940828000
8	2.865305000	-3.629803000	-1.557404000
6	1.499586000	-3.873025000	-1.821133000
1	0.965756000	-4.237709000	-0.928541000
1	0.976728000	-2.974878000	-2.188575000
1	1.430350000	-4.646529000	-2.595731000
1	2.919325000	-3.059264000	-0.767935000

6-Li

E (BS1) = -2869.756880

E (BS2) = -4010.305782

$G_{298.15,\ 1M}$ (BS2) = -4009.534047

1	2.428139000	0.279153000	2.139872000
1	0.891706000	-0.539204000	-1.468852000
1	-1.041441000	0.154948000	-2.158846000
3	3.827661000	-0.663408000	-1.042077000
1	4.174427000	-1.643918000	0.947055000
8	4.403137000	-2.609323000	0.854668000
6	5.707893000	-2.788898000	1.357988000
1	6.201688000	-3.602599000	0.811159000
1	6.322053000	-1.883534000	1.241992000
1	5.708131000	-3.060508000	2.426875000
1	2.231172000	0.758645000	-2.112229000
8	3.189345000	0.764463000	-2.323048000
6	3.659519000	2.089473000	-2.128696000
1	4.665387000	2.158954000	-2.556651000
1	3.020132000	2.820282000	-2.642259000
1	3.711525000	2.352441000	-1.061230000

8	3.895259000	-0.007462000	0.717607000
6	3.480208000	0.571545000	1.916231000
6	3.507772000	2.094134000	1.847405000
6	4.332264000	0.089474000	3.086215000
1	2.879136000	2.467835000	1.026754000
1	4.533074000	2.453158000	1.674354000
1	3.146122000	2.547547000	2.780528000
1	3.993867000	0.516480000	4.040270000
1	4.290917000	-1.004576000	3.176077000
1	5.383328000	0.377201000	2.934535000
8	6.521869000	0.465455000	0.395896000
6	6.807936000	1.833603000	0.254303000
1	5.546692000	0.349806000	0.559276000
1	7.808625000	1.942735000	-0.182851000
1	6.098466000	2.347920000	-0.415219000
1	6.811948000	2.372705000	1.217192000
1	1.355128000	1.071481000	0.069732000
1	0.722792000	1.129130000	0.613657000
26	-4.473292000	-0.327515000	0.152302000
77	-0.106442000	0.599647000	-0.940650000
15	-1.095278000	-1.054928000	0.443530000
16	-1.841667000	2.315003000	-0.473684000
6	-2.722260000	-0.635461000	1.135164000
6	-3.205281000	0.700647000	1.384441000
6	-4.498788000	0.585870000	1.980314000
1	-5.131373000	1.424574000	2.254456000
6	-4.817798000	-0.793493000	2.111784000
1	-5.750915000	-1.199834000	2.486962000
6	-3.730637000	-1.547563000	1.593516000
1	-3.692248000	-2.628536000	1.511200000
6	-4.953409000	0.802867000	-1.488764000
1	-4.693223000	1.847058000	-1.622454000
6	-4.155308000	-0.314315000	-1.870338000
1	-3.177436000	-0.264900000	-2.339459000
6	-4.838425000	-1.502218000	-1.473534000
1	-4.471388000	-2.518408000	-1.585003000
6	-6.061546000	-1.116151000	-0.850657000
1	-6.787796000	-1.786142000	-0.402953000
6	-6.131146000	0.307736000	-0.857992000
1	-6.921767000	0.908878000	-0.421991000
6	-2.495936000	2.001615000	1.226750000
1	-1.635903000	2.072154000	1.908242000
1	-3.185356000	2.823413000	1.452964000
6	-0.133420000	-1.462125000	1.955926000
6	1.041693000	-2.214552000	1.845841000
1	1.347978000	-2.617746000	0.879852000
6	1.821624000	-2.472779000	2.967766000
1	2.731038000	-3.061895000	2.862009000
6	1.448354000	-1.965479000	4.211042000
1	2.063098000	-2.162777000	5.086932000
6	0.290131000	-1.203725000	4.326481000
1	-0.009734000	-0.807432000	5.294368000
6	-0.500733000	-0.955552000	3.205785000
1	-1.412786000	-0.368872000	3.314186000
6	-1.363690000	-2.699227000	-0.328318000
6	-1.615488000	-3.831631000	0.456952000
1	-1.604914000	-3.756420000	1.543890000
6	-1.873539000	-5.060147000	-0.141382000
1	-2.070299000	-5.931944000	0.478774000
6	-1.872330000	-5.175359000	-1.530242000
1	-2.070358000	-6.137997000	-1.996708000
6	-1.605347000	-4.060390000	-2.318255000
1	-1.591385000	-4.146521000	-3.402719000
6	-1.350350000	-2.828253000	-1.720333000
1	-1.142919000	-1.956469000	-2.338755000
6	-1.125044000	3.936524000	-0.150494000
6	-2.000445000	5.013419000	0.009696000
1	-3.076143000	4.859617000	-0.066164000
6	-1.492191000	6.282160000	0.264552000
1	-2.174913000	7.118976000	0.393066000
6	-0.115429000	6.481600000	0.345298000
1	0.279132000	7.476277000	0.540039000
6	0.753215000	5.409304000	0.169464000
1	1.829676000	5.559276000	0.225054000
6	0.252138000	4.133213000	-0.077369000
1	0.929095000	3.295381000	-0.225994000
1	0.515091000	1.625266000	-2.090393000
8	5.541168000	-1.333325000	-1.871345000
6	6.214052000	-0.510760000	-2.814780000
1	7.194008000	-0.926366000	-3.078836000
1	5.625682000	-0.374966000	-3.732380000
1	6.369978000	0.465434000	-2.344196000
1	5.407874000	-2.207902000	-2.260587000
8	2.722328000	-2.233138000	-1.732070000
6	2.496034000	-2.335516000	-3.132258000
1	2.145133000	-3.338359000	-3.406110000
1	1.765736000	-1.593410000	-3.484482000
1	3.451063000	-2.153188000	-3.635644000
1	1.867108000	-2.316501000	-1.286620000

$TS_{6\text{-}7}$-Li
E (BS1) = -2869.751569
E (BS2) = -4010.298199
$G_{298.15,\ 1M}$ (BS2) = -4009.531955

1	2.070445000	1.058007000	2.965468000
1	1.269773000	-0.689809000	-0.921502000
1	-0.488953000	-0.041376000	-2.088775000
3	4.170452000	-1.185644000	-1.218159000
1	3.189630000	-1.049256000	0.954161000
8	3.361298000	-1.913737000	0.480679000
6	4.205723000	-2.741334000	1.251911000
1	4.475252000	-3.616832000	0.649085000
1	5.133913000	-2.228840000	1.544976000
1	3.706319000	-3.103619000	2.162986000
1	2.541533000	0.424230000	-1.978981000
8	3.491606000	0.404507000	-2.228929000
6	3.988941000	1.720526000	-2.043365000
1	5.050444000	1.723276000	-2.311707000
1	3.469170000	2.436819000	-2.694609000
1	3.884417000	2.052271000	-0.998939000
8	3.036355000	0.591646000	1.205759000
6	3.083398000	1.119944000	2.500434000
6	3.476388000	2.589437000	2.452134000
6	4.039772000	0.323212000	3.373445000
1	2.771326000	3.155829000	1.827054000
1	4.478436000	2.695262000	2.007987000
1	3.494966000	3.050402000	3.448781000
1	4.044148000	0.684792000	4.410635000
1	3.759687000	-0.739209000	3.386974000
1	5.065994000	0.400152000	2.982739000
8	5.429884000	0.053872000	0.138397000
6	6.471709000	0.990493000	0.269857000
1	4.610328000	0.404272000	0.579096000
1	7.373915000	0.578730000	-0.197847000
1	6.245738000	1.950619000	-0.220125000
1	6.713209000	1.195275000	1.324668000
1	1.687632000	0.857933000	0.745144000

1	0.845800000	1.191734000	0.819166000
26	-4.352102000	-0.236501000	-0.201121000
77	0.182446000	0.487358000	-0.717863000
15	-1.066991000	-0.992558000	0.616719000
16	-1.549999000	2.237985000	-0.731262000
6	-2.760124000	-0.468154000	1.039275000
6	-3.228984000	0.895169000	1.077132000
6	-4.594212000	0.870919000	1.498757000
1	-5.229725000	1.746198000	1.592802000
6	-4.973426000	-0.479689000	1.730793000
1	-5.961211000	-0.825567000	2.016259000
6	-3.851215000	-1.305110000	1.449809000
1	-3.838848000	-2.389208000	1.486598000
6	-4.586157000	0.736528000	-1.987733000
1	-4.307062000	1.767959000	-2.173602000
6	-3.741802000	-0.400696000	-2.145983000
1	-2.702066000	-0.380743000	-2.459252000
6	-4.478621000	-1.556557000	-1.749416000
1	-4.100020000	-2.574123000	-1.713544000
6	-5.779375000	-1.130903000	-1.349267000
1	-6.564635000	-1.766794000	-0.954506000
6	-5.844870000	0.285795000	-1.494736000
1	-6.690287000	0.914137000	-1.235605000
6	-2.466837000	2.158403000	0.869717000
1	-1.721144000	2.309081000	1.663933000
1	-3.158639000	3.009385000	0.877593000
6	-0.348331000	-1.248391000	2.291507000
6	0.745287000	-2.104283000	2.462899000
1	1.122641000	-2.684041000	1.619857000
6	1.359883000	-2.219413000	3.706250000
1	2.198291000	-2.902724000	3.833644000
6	0.905200000	-1.466918000	4.787451000
1	1.392323000	-1.554715000	5.756340000
6	-0.178035000	-0.608871000	4.622833000
1	-0.545139000	-0.023533000	5.463376000
6	-0.804681000	-0.503248000	3.383083000
1	-1.657520000	0.166181000	3.270784000
6	-1.310110000	-2.706844000	0.000185000
6	-1.708967000	-3.743228000	0.853807000
1	-1.828802000	-3.556884000	1.920578000
6	-1.948956000	-5.017731000	0.351533000
1	-2.260259000	-5.813549000	1.024821000
6	-1.784488000	-5.276032000	-1.008165000
1	-1.969316000	-6.274548000	-1.398626000
6	-1.372052000	-4.257729000	-1.861984000
1	-1.231625000	-4.455779000	-2.922771000
6	-1.134940000	-2.979926000	-1.359857000
1	-0.815817000	-2.181370000	-2.029227000
6	-0.871571000	3.893478000	-0.513188000
6	-1.730340000	4.973831000	-0.729115000
1	-2.765351000	4.796801000	-1.019797000
6	-1.258203000	6.272693000	-0.574919000
1	-1.928120000	7.113088000	-0.742861000
6	0.069835000	6.494986000	-0.217139000
1	0.439207000	7.511805000	-0.103667000
6	0.924603000	5.416294000	-0.011155000
1	1.963784000	5.586580000	0.263418000
6	0.457877000	4.111573000	-0.156539000
1	1.123251000	3.263475000	-0.004447000
1	1.101519000	1.479938000	-1.689448000
8	5.760542000	-2.012957000	-2.127160000
6	6.527736000	-1.147393000	-2.955727000
1	7.427446000	-1.650482000	-3.329953000
1	5.944613000	-0.778396000	-3.809716000
1	6.838034000	-0.295288000	-2.343242000
1	5.458937000	-2.758482000	-2.663528000
8	2.820516000	-2.529212000	-2.143842000
6	2.261762000	-2.361067000	-3.435360000
1	1.700154000	-3.249845000	-3.752850000
1	1.598021000	-1.486298000	-3.486348000
1	3.091907000	-2.208016000	-4.133862000
1	2.109641000	-2.516417000	-1.485323000

7-Li

E (BS1) = -2869.779538

E (BS2) = -4010.322894

$G_{298.15, 1M}$ (BS2) = -4009.549761

1	2.279043000	1.129499000	3.587201000
1	1.647322000	-0.135324000	-0.352783000
1	0.060404000	0.241060000	-1.993207000
3	3.857811000	-0.790475000	-1.338836000
1	3.633386000	-1.056921000	1.183499000
8	4.052176000	-1.715049000	0.592480000
6	5.370156000	-1.936202000	1.063566000
1	5.841628000	-2.680592000	0.413755000
1	5.979303000	-1.020597000	1.037027000
1	5.374011000	-2.330693000	2.089116000
1	1.963644000	0.347702000	-2.323141000
8	2.793893000	0.285074000	-2.846429000
6	2.858780000	1.432869000	-3.670047000
1	3.813436000	1.418810000	-4.207204000
1	2.049235000	1.439872000	-4.414138000
1	2.800718000	2.364848000	-3.088905000
8	3.109509000	0.591984000	1.776720000
6	3.218215000	1.281164000	3.026319000
6	3.433652000	2.759188000	2.780466000
6	4.365218000	0.648930000	3.777527000
1	2.616171000	3.183082000	2.183500000
1	4.375639000	2.916098000	2.235847000
1	3.488940000	3.312030000	3.726039000
1	4.496007000	1.119019000	4.758728000
1	4.190008000	-0.423344000	3.930322000
1	5.300113000	0.772895000	3.212642000
8	4.704961000	0.928175000	-0.441913000
6	4.620019000	2.277826000	-0.862944000
1	4.279583000	0.879891000	0.440682000
1	4.988621000	2.343592000	-1.892320000
1	3.584056000	2.649680000	-0.836169000
1	5.245266000	2.933259000	-0.239835000
1	2.252899000	0.819648000	1.340039000
1	0.608382000	1.384185000	1.171825000
26	-4.185838000	-0.758904000	-0.065347000
77	0.298920000	0.765673000	-0.394770000
15	-0.792403000	-1.024630000	0.639861000
16	-1.756540000	2.076520000	-0.617722000
6	-2.533429000	-0.764584000	1.123681000
6	-3.212079000	0.506253000	1.201892000
6	-4.541455000	0.258427000	1.665034000
1	-5.305400000	1.019690000	1.791304000
6	-4.695915000	-1.139355000	1.877559000
1	-5.606716000	-1.642310000	2.184713000
6	-3.467714000	-1.770427000	1.543203000
1	-3.281036000	-2.839045000	1.558704000
6	-4.607463000	0.232605000	-1.803945000
1	-4.473372000	1.299445000	-1.951173000
6	-3.621909000	-0.771875000	-2.030494000
1	-2.607928000	-0.600185000	-2.378971000

6	-4.181345000	-2.031648000	-1.660501000
1	-3.668109000	-2.988651000	-1.681043000
6	-5.514028000	-1.803215000	-1.208327000
1	-6.193263000	-2.554424000	-0.819396000
6	-5.776591000	-0.404516000	-1.295724000
1	-6.690119000	0.092569000	-0.987186000
6	-2.695411000	1.882907000	0.958981000
1	-2.020514000	2.203663000	1.765654000
1	-3.538111000	2.584545000	0.908073000
6	-0.013604000	-1.434172000	2.257595000
6	1.196623000	-2.134741000	2.275120000
1	1.613540000	-2.526623000	1.346722000
6	1.889000000	-2.323776000	3.466411000
1	2.831573000	-2.869762000	3.459897000
6	1.380046000	-1.812239000	4.659045000
1	1.922677000	-1.956229000	5.591167000
6	0.173717000	-1.116628000	4.651510000
1	-0.232661000	-0.718510000	5.579154000
6	-0.519752000	-0.927705000	3.457510000
1	-1.458037000	-0.371976000	3.464250000
6	-0.852810000	-2.678508000	-0.177703000
6	-1.086984000	-3.854233000	0.547309000
1	-1.186908000	-3.817900000	1.631605000
6	-1.186386000	-5.079233000	-0.104279000
1	-1.369954000	-5.983551000	0.472215000
6	-1.043406000	-5.149999000	-1.488992000
1	-1.118327000	-6.109474000	-1.996267000
6	-0.794266000	-3.991299000	-2.217890000
1	-0.673765000	-4.038570000	-3.298616000
6	-0.698346000	-2.763312000	-1.564477000
1	-0.499754000	-1.853954000	-2.133804000
6	-1.497013000	3.854489000	-0.488814000
6	-2.371276000	4.688265000	-1.186191000
1	-3.168944000	4.255824000	-1.789159000
6	-2.215677000	6.069708000	-1.107654000
1	-2.892481000	6.721065000	-1.656217000
6	-1.196379000	6.613010000	-0.330630000
1	-1.074430000	7.692388000	-0.272240000
6	-0.331083000	5.776210000	0.371006000
1	0.464842000	6.200120000	0.979792000
6	-0.476235000	4.394568000	0.291541000
1	0.199251000	3.727085000	0.824016000
1	1.229053000	2.013589000	-0.989873000
8	5.508895000	-1.407310000	-2.304855000
6	5.956526000	-0.599229000	-3.385780000
1	6.971206000	-0.876722000	-3.696812000
1	5.286920000	-0.670719000	-4.253285000
1	5.975670000	0.436465000	-3.031521000
1	5.450839000	-2.322907000	-2.608157000
8	2.681880000	-2.485008000	-1.861646000
6	2.439369000	-2.853498000	-3.210178000
1	2.162785000	-3.912698000	-3.294045000
1	1.655351000	-2.239043000	-3.673630000
1	3.371328000	-2.697627000	-3.764253000
1	1.854076000	-2.571134000	-1.364764000

B3. Sodium system

1-Na

E (BS1) = -2830.250256
E (BS2) = -3970.737494
$G_{298.15,1M}$ (BS2) = -3970.074463

11	3.783819000	-0.595479000	0.011393000
1	2.766689000	-2.816720000	1.524161000
8	3.518491000	-2.218602000	1.645404000
6	4.214535000	-2.606567000	2.813091000
1	5.123610000	-1.998937000	2.877021000
1	3.620994000	-2.442135000	3.724629000
1	4.516013000	-3.662836000	2.778453000
1	2.966119000	0.601582000	-2.152196000
1	1.957302000	-1.697544000	-1.296748000
8	2.737416000	-2.297263000	-1.311237000
8	3.938001000	0.614610000	-2.024545000
6	2.945737000	-2.681502000	-2.655827000
1	3.771801000	-3.400431000	-2.680620000
1	2.059021000	-3.174512000	-3.081582000
1	3.210265000	-1.827487000	-3.298121000
6	4.313038000	1.970109000	-1.851183000
1	5.384442000	1.997930000	-1.623564000
1	4.140455000	2.561729000	-2.761972000
1	3.768255000	2.448951000	-1.022810000
26	-3.789690000	1.053035000	0.906392000
77	0.248256000	0.269654000	-1.283892000
15	-1.218249000	-1.076740000	-0.061047000
16	-0.435262000	2.341634000	-0.135708000
6	-2.242650000	-0.236053000	1.195335000
6	-1.951988000	1.041670000	1.796584000
6	-2.971992000	1.310582000	2.760268000
1	-3.041358000	2.217248000	3.353697000
6	-3.884495000	0.220097000	2.770047000
1	-4.790929000	0.150425000	3.361900000
6	-3.441249000	-0.730303000	1.811029000
1	-3.953101000	-1.650930000	1.552105000
6	-3.995915000	2.728034000	-0.251728000
1	-3.304774000	3.563941000	-0.258049000
6	-3.908637000	1.563912000	-1.069923000
1	-3.131614000	1.358758000	-1.800968000
6	-4.978385000	0.690510000	-0.710302000
1	-5.163004000	-0.297396000	-1.122243000
6	-5.726655000	1.318478000	0.328251000
1	-6.578888000	0.892066000	0.846709000
6	-5.118660000	2.576126000	0.612768000
1	-5.430744000	3.274628000	1.381800000
6	-0.756193000	1.915714000	1.616690000
1	0.140786000	1.413452000	2.006748000
1	-0.905866000	2.855234000	2.163222000
6	-0.374579000	-2.352689000	0.969309000
6	0.152210000	-3.495597000	0.357997000
1	0.000403000	-3.662158000	-0.709544000
6	0.865446000	-4.431053000	1.102874000
1	1.263635000	-5.319175000	0.616528000
6	1.071127000	-4.229267000	2.468426000
1	1.630803000	-4.958664000	3.050804000
6	0.559689000	-3.087172000	3.081720000
1	0.716442000	-2.922139000	4.145949000
6	-0.159738000	-2.155771000	2.336730000
1	-0.556929000	-1.268719000	2.830541000
6	-2.465273000	-2.093576000	-0.963012000
6	-3.106735000	-3.180713000	-0.354651000
1	-2.832334000	-3.482790000	0.655661000
6	-4.094442000	-3.887961000	-1.031961000
1	-4.586204000	-4.728029000	-0.545862000
6	-4.447069000	-3.526924000	-2.331168000
1	-5.217729000	-4.083163000	-2.860870000
6	-3.801891000	-2.461986000	-2.951812000
1	-4.062933000	-2.182433000	-3.970490000
6	-2.815877000	-1.750857000	-2.270993000

1	-2.308343000	-0.916883000	-2.754942000
6	0.988269000	3.423611000	0.029127000
6	1.369824000	4.112903000	-1.126073000
1	0.814756000	3.969986000	-2.052819000
6	2.460288000	4.972918000	-1.094730000
1	2.750282000	5.501557000	-2.000374000
6	3.178930000	5.157722000	0.085667000
1	4.031676000	5.832144000	0.108296000
6	2.790053000	4.481697000	1.236557000
1	3.336129000	4.623500000	2.167531000
6	1.694285000	3.615945000	1.217288000
1	1.407634000	3.109884000	2.136255000
1	0.612838000	-1.017595000	-2.197135000
1	1.339847000	1.130862000	-2.204881000
1	1.481076000	-0.013249000	-0.113397000
1	-0.864625000	0.625929000	-2.490043000
8	3.925969000	1.217353000	1.497097000
6	3.464439000	0.922646000	2.807861000
1	3.311748000	1.837671000	3.396065000
1	2.525573000	0.346344000	2.796887000
1	4.234226000	0.326074000	3.306675000
1	3.330924000	1.884797000	1.120741000
8	6.071008000	-1.093103000	0.087117000
6	6.259561000	-2.326186000	-0.593431000
1	7.302281000	-2.664586000	-0.538674000
1	5.630060000	-3.074893000	-0.100036000
1	5.967737000	-2.259617000	-1.651353000
1	6.610535000	-0.423612000	-0.353516000

2-Na

E (BS1) = -3023.305635

E (BS2) = -4163.867847

$G_{298.15,1M}$ (BS2) = -4163.118567

11	3.585155000	-1.092570000	-0.407797000
1	1.808734000	-2.109764000	1.436939000
8	2.591746000	-2.611657000	1.156145000
6	3.328964000	-2.900122000	2.328630000
1	4.126723000	-3.602952000	2.065617000
1	3.786741000	-1.997779000	2.763759000
1	2.701048000	-3.375843000	3.096536000
1	4.287809000	0.235284000	-2.782739000
1	1.871298000	-1.877006000	-2.081435000
8	2.702694000	-2.391778000	-2.175404000
8	4.707531000	0.343686000	-1.919050000
6	3.074914000	-2.343086000	-3.539934000
1	4.103131000	-2.710053000	-3.627599000
1	2.429478000	-2.979411000	-4.162753000
1	3.033073000	-1.320296000	-3.943379000
6	4.594450000	1.713002000	-1.553643000
1	5.094461000	1.837800000	-0.587062000
1	5.088462000	2.366775000	-2.284961000
1	3.543656000	2.019237000	-1.451656000
26	-3.474842000	1.366243000	1.119101000
77	0.170263000	0.113783000	-1.720908000
15	-1.466453000	-1.004924000	-0.488419000
16	-0.221060000	2.231493000	-0.535077000
6	-2.099455000	-0.136275000	0.990561000
6	-1.524239000	1.032915000	1.610204000
6	-2.293619000	1.326135000	2.779594000
1	-2.127027000	2.177187000	3.433764000
6	-3.334299000	0.362767000	2.893609000
1	-4.111466000	0.348276000	3.650424000
6	-3.223183000	-0.529880000	1.793832000
1	-3.893367000	-1.355270000	1.576826000
6	-3.623771000	3.163492000	0.152394000
1	-2.815142000	3.880412000	0.057692000
6	-3.901253000	2.092175000	-0.745261000
1	-3.342764000	1.854358000	-1.645047000
6	-5.009474000	1.357176000	-0.227898000
1	-5.440393000	0.460762000	-0.663803000
6	-5.416660000	1.977663000	0.989153000
1	-6.207431000	1.633285000	1.647197000
6	-4.559826000	3.092590000	1.224744000
1	-4.587166000	3.744885000	2.091114000
6	-0.311181000	1.821671000	1.244862000
1	0.598307000	1.258838000	1.496283000
1	-0.320728000	2.767410000	1.802953000
6	-0.856705000	-2.588729000	0.237527000
6	-0.421559000	-3.588427000	-0.641836000
1	-0.472552000	-3.421765000	-1.718818000
6	0.076483000	-4.792070000	-0.157272000
1	0.407707000	-5.559078000	-0.854529000
6	0.152247000	-5.015579000	1.217197000
1	0.543847000	-5.956966000	1.596944000
6	-0.278578000	-4.030177000	2.098637000
1	-0.230691000	-4.198393000	3.173757000
6	-0.783198000	-2.824045000	1.611963000
1	-1.122598000	-2.066417000	2.316582000
6	-3.028888000	-1.587553000	-1.288103000
6	-3.784693000	-2.634373000	-0.744852000
1	-3.430825000	-3.162226000	0.140342000
6	-4.987419000	-3.016922000	-1.330019000
1	-5.564058000	-3.831232000	-0.896077000
6	-5.446435000	-2.366500000	-2.473806000
1	-6.384207000	-2.670253000	-2.934285000
6	-4.693869000	-1.337805000	-3.032884000
1	-5.039237000	-0.834424000	-3.933640000
6	-3.491096000	-0.953466000	-2.443759000
1	-2.895817000	-0.154413000	-2.886098000
6	1.242152000	3.272755000	-0.566627000
6	1.467428000	3.960776000	-1.763684000
1	0.784804000	3.827692000	-2.602880000
6	2.560832000	4.808624000	-1.883839000
1	2.727455000	5.339219000	-2.818839000
6	3.433503000	4.985807000	-0.810610000
1	4.286255000	5.654459000	-0.903264000
6	3.203245000	4.305643000	0.379720000
1	3.878940000	4.436731000	1.223084000
6	2.111457000	3.447090000	0.509696000
1	1.964102000	2.920144000	1.449416000
1	0.352336000	-1.221377000	-2.620697000
1	1.452726000	0.758027000	-2.572237000
1	1.291444000	-0.392288000	-0.536103000
1	-0.836465000	0.643567000	-2.976337000
8	3.500694000	0.503959000	1.353343000
6	4.302644000	1.097658000	2.352557000
1	3.839094000	2.005098000	2.770009000
1	4.506293000	0.406422000	3.185767000
1	5.260197000	1.386819000	1.904887000
1	2.677464000	0.201537000	1.787873000
8	5.656864000	-2.130316000	-0.028006000
6	6.483308000	-1.274619000	0.745183000
1	7.523325000	-1.625628000	0.768363000
1	6.468076000	-0.287423000	0.270382000
1	6.122139000	-1.175358000	1.779383000
1	5.640641000	-2.999827000	0.390829000
6	0.959773000	-0.220245000	3.868036000

8	1.362304000	-0.615418000	2.775081000
6	1.293161000	1.143913000	4.372939000
6	0.113089000	-1.082121000	4.742064000
1	1.643731000	1.792753000	3.563618000
1	0.146951000	-2.127058000	4.420965000
1	2.093368000	1.048451000	5.120380000
1	0.437066000	1.595824000	4.886970000
1	-0.923796000	-0.719503000	4.678825000
1	0.413388000	-0.991593000	5.792019000

$TS_{2\text{-}3}$-Na

E (BS1) = -3023.289166

E (BS2) = -4163.846497

$G_{298.15,1M}$ (BS2) = -4163.103376

11	-4.017181000	-0.122031000	0.592996000
1	-4.339949000	-2.168161000	-0.959774000
8	-5.137356000	-2.695145000	-0.727075000
6	-6.123999000	-2.362561000	-1.689174000
1	-7.037845000	-2.916599000	-1.450077000
1	-6.359628000	-1.286869000	-1.681589000
1	-5.808970000	-2.643261000	-2.704259000
1	-3.834882000	1.099753000	3.150193000
1	-2.174360000	-1.785777000	1.295058000
8	-3.055263000	-2.137178000	1.548773000
8	-4.194390000	1.476426000	2.336223000
6	-3.088499000	-2.154372000	2.964881000
1	-3.965156000	-2.727007000	3.282867000
1	-2.194596000	-2.635714000	3.385670000
1	-3.166371000	-1.141037000	3.391220000
6	-3.674992000	2.791686000	2.201002000
1	-4.096293000	3.222149000	1.285895000
1	-3.965036000	3.427305000	3.047599000
1	-2.578463000	2.792916000	2.116995000
26	4.084594000	1.611736000	-0.521121000
77	-0.153459000	-0.249312000	0.758703000
15	1.917090000	-1.080800000	0.043835000
16	0.375983000	2.013719000	0.002668000
6	2.913381000	0.018957000	-1.022812000
6	2.461984000	1.216127000	-1.689546000
6	3.545725000	1.704913000	-2.483234000
1	3.520996000	2.617097000	-3.071837000
6	4.657001000	0.832003000	-2.322016000
1	5.640399000	0.962206000	-2.761099000
6	4.275857000	-0.198089000	-1.421775000
1	4.917218000	-0.998487000	-1.067518000
6	3.705800000	3.285624000	0.591347000
1	2.842839000	3.929942000	0.457948000
6	3.770787000	2.135998000	1.431102000
1	2.967635000	1.755336000	2.054135000
6	5.059576000	1.544495000	1.272809000
1	5.406906000	0.633991000	1.751948000
6	5.790386000	2.331321000	0.335490000
1	6.790036000	2.122202000	-0.030425000
6	4.953741000	3.406117000	-0.086325000
1	5.206400000	4.156850000	-0.827476000
6	1.126472000	1.877109000	-1.677132000
1	0.403761000	1.336980000	-2.304354000
1	1.223201000	2.901461000	-2.059966000
6	1.794996000	-2.589646000	-1.004083000
6	1.450345000	-3.806405000	-0.402367000
1	1.315963000	-3.859433000	0.678693000
6	1.283638000	-4.952808000	-1.171033000
1	1.026696000	-5.893613000	-0.688521000
6	1.442217000	-4.896145000	-2.555137000
1	1.309537000	-5.793005000	-3.156429000
6	1.769829000	-3.688574000	-3.163261000
1	1.895641000	-3.636545000	-4.242860000
6	1.949828000	-2.541030000	-2.391753000
1	2.214084000	-1.603387000	-2.881072000
6	3.156659000	-1.616322000	1.305090000
6	4.193973000	-2.502508000	0.986272000
1	4.248517000	-2.948165000	-0.006271000
6	5.159442000	-2.832081000	1.932187000
1	5.959000000	-3.521411000	1.668741000
6	5.098422000	-2.287985000	3.213723000
1	5.852086000	-2.549608000	3.953409000
6	4.063056000	-1.419779000	3.546068000
1	4.000961000	-1.000144000	4.548053000
6	3.097693000	-1.089694000	2.597587000
1	2.283131000	-0.415625000	2.860479000
6	-1.063701000	2.978367000	-0.484458000
6	-1.009807000	4.358665000	-0.289664000
1	-0.128190000	4.809019000	0.164920000
6	-2.085155000	5.154095000	-0.678698000
1	-2.046442000	6.229191000	-0.518064000
6	-3.198852000	4.574564000	-1.280104000
1	-4.034555000	5.196714000	-1.594144000
6	-3.245331000	3.195636000	-1.476078000
1	-4.116652000	2.735063000	-1.937026000
6	-2.187381000	2.390431000	-1.063784000
1	-2.236597000	1.309223000	-1.191489000
1	-0.439022000	-1.713631000	1.401669000
1	-1.621055000	0.283826000	1.330965000
1	-0.986722000	-0.749502000	-0.752563000
1	0.408550000	0.179955000	2.241628000
8	-5.594757000	1.054956000	-0.678798000
6	-6.420312000	2.107284000	-0.209866000
1	-6.112589000	3.082572000	-0.614812000
1	-7.477147000	1.942282000	-0.459087000
1	-6.321706000	2.141008000	0.880399000
1	-5.658829000	1.010721000	-1.640884000
8	-5.939165000	-1.343902000	1.548695000
6	-7.294224000	-0.984414000	1.363160000
1	-7.967662000	-1.844065000	1.491381000
1	-7.556009000	-0.236611000	2.119522000
1	-7.475392000	-0.546602000	0.370195000
1	-5.706459000	-1.997184000	0.855473000
6	-2.115628000	-1.331296000	-1.862664000
8	-3.244934000	-0.877135000	-1.503621000
6	-1.453777000	-0.723288000	-3.073164000
6	-1.826727000	-2.793617000	-1.647115000
1	-1.584152000	0.363347000	-3.101569000
1	-2.124146000	-3.118674000	-0.644822000
1	-1.957149000	-1.142579000	-3.958324000
1	-0.389840000	-0.976486000	-3.138343000
1	-0.774255000	-3.035347000	-1.820363000
1	-2.431222000	-3.350658000	-2.380691000

3-Na

E (BS1) = -3023.303452

E (BS2) = -4163.862027

$G_{298.15,1M}$ (BS2) = -4163.113153

11	-4.000792000	-0.150087000	0.586551000
1	-4.329154000	-1.964086000	-1.071861000
8	-5.116755000	-2.544632000	-0.802687000
6	-6.143689000	-2.262638000	-1.729158000

1	-7.029616000	-2.856552000	-1.474316000
1	-6.432797000	-1.198731000	-1.717521000
1	-5.848291000	-2.523801000	-2.757017000
1	-3.791168000	1.086553000	3.153855000
1	-2.162456000	-1.803788000	1.395045000
8	-3.042062000	-2.145216000	1.660154000
8	-4.159852000	1.456893000	2.341414000
6	-3.069427000	-2.140369000	3.075559000
1	-3.961932000	-2.682010000	3.403633000
1	-2.189491000	-2.642785000	3.501676000
1	-3.117343000	-1.120073000	3.488692000
6	-3.654745000	2.776673000	2.199337000
1	-4.092371000	3.204476000	1.290686000
1	-3.938092000	3.409756000	3.050143000
1	-2.559403000	2.788713000	2.099509000
26	4.096072000	1.622839000	-0.485420000
77	-0.163672000	-0.270809000	0.715771000
15	1.945545000	-1.090003000	0.017971000
16	0.386649000	2.005144000	-0.027679000
6	2.952515000	0.023348000	-1.017507000
6	2.502581000	1.219096000	-1.688495000
6	3.597485000	1.719936000	-2.458603000
1	3.576638000	2.633850000	-3.044554000
6	4.712938000	0.856028000	-2.278422000
1	5.703490000	0.996874000	-2.697545000
6	4.324119000	-0.181409000	-1.390165000
1	4.964378000	-0.978643000	-1.026532000
6	3.683541000	3.292234000	0.621769000
1	2.820842000	3.932864000	0.470080000
6	3.735309000	2.140827000	1.459971000
1	2.920069000	1.754733000	2.063676000
6	5.030330000	1.555981000	1.329250000
1	5.371839000	0.646288000	1.813995000
6	5.778128000	2.349198000	0.411044000
1	6.786675000	2.145967000	0.066997000
6	4.945888000	3.420821000	-0.026832000
1	5.211237000	4.174642000	-0.760335000
6	1.159399000	1.864464000	-1.699921000
1	0.449646000	1.311919000	-2.331969000
1	1.247622000	2.887609000	-2.087315000
6	1.822930000	-2.582827000	-1.047192000
6	1.460436000	-3.803979000	-0.464997000
1	1.317594000	-3.871713000	0.614096000
6	1.288634000	-4.936504000	-1.252431000
1	1.018688000	-5.881992000	-0.786702000
6	1.456779000	-4.859672000	-2.634380000
1	1.318764000	-5.745746000	-3.250175000
6	1.799711000	-3.646494000	-3.222631000
1	1.932125000	-3.579526000	-4.300523000
6	1.986779000	-2.512850000	-2.433121000
1	2.263577000	-1.570276000	-2.905665000
6	3.138608000	-1.625125000	1.313005000
6	4.182064000	-2.511630000	1.017864000
1	4.260268000	-2.954308000	0.025557000
6	5.122809000	-2.842863000	1.987575000
1	5.928511000	-3.532026000	1.743699000
6	5.029793000	-2.299284000	3.267235000
1	5.764470000	-2.561823000	4.025316000
6	3.988068000	-1.429475000	3.575011000
1	3.902655000	-1.009714000	4.575024000
6	3.046940000	-1.096971000	2.603645000
1	2.232893000	-0.417413000	2.851571000
6	-1.052106000	2.965840000	-0.520206000
6	-0.994309000	4.348371000	-0.341655000
1	-0.107492000	4.803478000	0.097768000
6	-2.073821000	5.139436000	-0.726620000
1	-2.032936000	6.216338000	-0.579531000
6	-3.195771000	4.552160000	-1.304843000
1	-4.036111000	5.170487000	-1.614009000
6	-3.245264000	3.171211000	-1.483687000
1	-4.124768000	2.706765000	-1.924726000
6	-2.181713000	2.368791000	-1.079135000
1	-2.243573000	1.285554000	-1.196822000
1	-0.423648000	-1.726137000	1.386437000
1	-1.598626000	0.275475000	1.339551000
1	-1.301403000	-0.894231000	-0.902063000
1	0.433639000	0.159925000	2.094682000
8	-5.635695000	1.099899000	-0.610125000
6	-6.441409000	2.166177000	-0.144087000
1	-6.093278000	3.140836000	-0.517595000
1	-7.495380000	2.042343000	-0.428223000
1	-6.377708000	2.172631000	0.949433000
1	-5.683866000	1.067267000	-1.573866000
8	-5.932264000	-1.386652000	1.548734000
6	-7.288118000	-1.022144000	1.387503000
1	-7.961353000	-1.886635000	1.484525000
1	-7.546961000	-0.306137000	2.175442000
1	-7.477575000	-0.545339000	0.414243000
1	-5.685916000	-1.965730000	0.793288000
6	-2.029658000	-1.303273000	-1.713975000
8	-3.291928000	-0.852605000	-1.487481000
6	-1.460024000	-0.809897000	-3.041968000
6	-1.908324000	-2.821267000	-1.618711000
1	-1.525591000	0.283565000	-3.117914000
1	-2.219386000	-3.180530000	-0.628992000
1	-2.047384000	-1.236819000	-3.868465000
1	-0.410229000	-1.108917000	-3.179970000
1	-0.883778000	-3.167968000	-1.807083000
1	-2.568522000	-3.281315000	-2.369744000

4-Na

E (BS1) = -3023.319125

E (BS2) = -4163.877527

$G_{298.15,1M}$ (BS2) = -4163.126264

1	0.208500000	0.507890000	2.755706000
1	1.435218000	-1.052178000	-0.890060000
1	-0.035235000	0.201342000	-2.019006000
11	4.055092000	-0.886385000	-0.830117000
1	2.613088000	-1.182183000	1.387061000
8	3.208785000	-1.946806000	1.126464000
6	4.124771000	-2.239877000	2.156853000
1	4.837241000	-2.986352000	1.785340000
1	4.696668000	-1.352331000	2.467638000
1	3.631137000	-2.661021000	3.046735000
1	2.455493000	0.104945000	-2.467433000
8	3.247919000	-0.183176000	-2.969955000
6	4.029171000	0.967512000	-3.248247000
1	4.747717000	0.708859000	-4.032509000
1	3.414366000	1.803390000	-3.607400000
1	4.596732000	1.313671000	-2.366871000
8	1.796768000	0.263181000	1.460870000
6	1.307733000	0.673916000	2.706849000
6	1.569298000	2.153494000	2.948717000
6	1.940763000	-0.153193000	3.816742000
1	1.130647000	2.771558000	2.154990000
1	2.654098000	2.343295000	2.962458000
1	1.155206000	2.488810000	3.909666000

1	1.553318000	0.129017000	4.805336000
1	1.744629000	-1.223919000	3.669851000
1	3.030856000	0.001121000	3.823113000
8	4.257278000	0.921221000	0.755492000
6	4.437774000	2.272681000	0.398467000
1	3.313671000	0.785609000	1.069900000
1	5.507840000	2.459714000	0.243946000
1	3.914287000	2.534048000	-0.537037000
1	4.092779000	2.958963000	1.187900000
26	-4.202668000	0.483048000	-0.087825000
77	0.580217000	0.271484000	-0.558718000
15	-1.126757000	-1.142256000	0.243418000
16	-0.901183000	2.261035000	-0.344635000
6	-2.636573000	-0.358340000	0.905974000
6	-2.799468000	1.023063000	1.290542000
6	-4.115380000	1.168221000	1.829913000
1	-4.544650000	2.105495000	2.170967000
6	-4.766640000	-0.095500000	1.790421000
1	-5.790165000	-0.296534000	2.088405000
6	-3.865923000	-1.032321000	1.218139000
1	-4.080575000	-2.076792000	1.017599000
6	-4.268839000	1.902414000	-1.558296000
1	-3.747150000	2.853083000	-1.519598000
6	-3.743816000	0.680434000	-2.070450000
1	-2.752519000	0.539392000	-2.489615000
6	-4.726644000	-0.336684000	-1.882682000
1	-4.614782000	-1.386939000	-2.135218000
6	-5.859694000	0.259584000	-1.255895000
1	-6.760051000	-0.257649000	-0.941544000
6	-5.576385000	1.642418000	-1.054530000
1	-6.224712000	2.360278000	-0.563479000
6	-1.835165000	2.160614000	1.242575000
1	-1.100492000	2.103416000	2.057134000
1	-2.393246000	3.098442000	1.348816000
6	-0.616839000	-2.204450000	1.658347000
6	0.318736000	-3.218584000	1.424566000
1	0.683972000	-3.396207000	0.412003000
6	0.785552000	-4.007928000	2.469900000
1	1.513157000	-4.792849000	2.270774000
6	0.325553000	-3.790094000	3.767672000
1	0.690549000	-4.406341000	4.586684000
6	-0.603204000	-2.782577000	4.010956000
1	-0.968037000	-2.608539000	5.021300000
6	-1.073896000	-1.994030000	2.962391000
1	-1.803130000	-1.211002000	3.168446000
6	-1.813079000	-2.398227000	-0.922105000
6	-2.453735000	-3.553069000	-0.454472000
1	-2.508249000	-3.754387000	0.614846000
6	-3.019036000	-4.459741000	-1.345496000
1	-3.512890000	-5.351705000	-0.965650000
6	-2.945134000	-4.231175000	-2.718010000
1	-3.383053000	-4.943532000	-3.413960000
6	-2.297521000	-3.095403000	-3.194353000
1	-2.225480000	-2.914325000	-4.264814000
6	-1.733987000	-2.186682000	-2.301457000
1	-1.222477000	-1.302664000	-2.679314000
6	-0.115113000	3.864634000	-0.116408000
6	-0.917476000	5.003544000	-0.008770000
1	-2.003524000	4.917208000	-0.026236000
6	-0.323161000	6.254925000	0.111397000
1	-0.949087000	7.139983000	0.202015000
6	1.065161000	6.377610000	0.099218000
1	1.525014000	7.359444000	0.186289000
6	1.859890000	5.243274000	-0.031007000
1	2.944735000	5.332438000	-0.046822000
6	1.271688000	3.985066000	-0.134836000
1	1.878821000	3.086756000	-0.230157000
1	1.838204000	1.166884000	-1.197428000
8	6.308481000	-1.079985000	-1.282359000
6	7.198516000	0.007689000	-1.480406000
1	8.235322000	-0.270984000	-1.252380000
1	7.153633000	0.392414000	-2.507994000
1	6.901860000	0.807517000	-0.794070000
1	6.548904000	-1.786413000	-1.895095000
8	3.459095000	-3.182032000	-1.333340000
6	2.444734000	-3.542656000	-2.253862000
1	2.007474000	-4.522226000	-2.016152000
1	1.635570000	-2.797047000	-2.294104000
1	2.901168000	-3.606021000	-3.246668000
1	3.072125000	-3.093478000	-0.442923000

5-Na

E (BS1) = -3024.492533

E (BS2) = -4165.053139

$G_{298.15, 1M}$ (BS2) = -4164.292675

1	0.188860000	0.516537000	2.701557000
1	1.445276000	-1.068232000	-0.893566000
1	-0.021280000	0.179772000	-2.038190000
11	4.071244000	-0.910594000	-0.821111000
1	2.612762000	-1.171565000	1.392882000
8	3.211391000	-1.940572000	1.151397000
6	4.118399000	-2.210482000	2.196599000
1	4.836537000	-2.962010000	1.847021000
1	4.684464000	-1.314514000	2.494841000
1	3.617310000	-2.615029000	3.090330000
1	2.461628000	0.046637000	-2.474291000
8	3.255979000	-0.242482000	-2.973251000
6	4.031013000	0.909455000	-3.263091000
1	4.759656000	0.642923000	-4.035266000
1	3.413264000	1.733961000	-3.643046000
1	4.586320000	1.275353000	-2.381894000
8	1.800927000	0.274219000	1.442389000
6	1.286840000	0.693305000	2.675786000
6	1.529357000	2.177150000	2.909833000
6	1.903531000	-0.116086000	3.807658000
1	1.103491000	2.784943000	2.101598000
1	2.611745000	2.377067000	2.946893000
1	1.090781000	2.516835000	3.858707000
1	1.492064000	0.172619000	4.785310000
1	1.720494000	-1.189901000	3.668563000
1	2.991738000	0.048866000	3.837319000
8	4.264209000	0.920430000	0.739764000
6	4.446516000	2.269801000	0.375284000
1	3.320147000	0.789126000	1.054137000
1	5.517602000	2.457283000	0.228784000
1	3.930569000	2.524407000	-0.566262000
1	4.094135000	2.960746000	1.157262000
1	-1.199836000	0.727391000	4.433646000
1	-1.592922000	1.226759000	4.825959000
26	-4.195737000	0.474014000	-0.148393000
77	0.592168000	0.261309000	-0.577149000
15	-1.118593000	-1.146380000	0.228073000
16	-0.889187000	2.253515000	-0.386071000
6	-2.637086000	-0.357005000	0.863311000
6	-2.804645000	1.026784000	1.236644000
6	-4.124320000	1.173613000	1.766089000
1	-4.557374000	2.112661000	2.097426000

6	-4.773731000	-0.091222000	1.730333000
1	-5.799290000	-0.291351000	2.021890000
6	-3.867519000	-1.030789000	1.171326000
1	-4.078697000	-2.077173000	0.976871000
6	-4.246987000	1.878777000	-1.633316000
1	-3.723922000	2.828889000	-1.599637000
6	-3.719722000	0.650811000	-2.128652000
1	-2.724886000	0.503914000	-2.537309000
6	-4.705906000	-0.362801000	-1.939106000
1	-4.593626000	-1.415733000	-2.180185000
6	-5.843289000	0.241700000	-1.328181000
1	-6.747334000	-0.270814000	-1.016718000
6	-5.559326000	1.626046000	-1.138347000
1	-6.210777000	2.350005000	-0.660583000
6	-1.840474000	2.164509000	1.191353000
1	-1.115231000	2.113359000	2.015124000
1	-2.400029000	3.102778000	1.284904000
6	-0.627355000	-2.182230000	1.668740000
6	0.311352000	-3.201490000	1.473796000
1	0.689065000	-3.407128000	0.470978000
6	0.766651000	-3.960515000	2.546830000
1	1.496116000	-4.750835000	2.378144000
6	0.295033000	-3.704536000	3.833524000
1	0.653785000	-4.295378000	4.673764000
6	-0.636386000	-2.690671000	4.038211000
1	-1.009256000	-2.482474000	5.039582000
6	-1.098681000	-1.936340000	2.961538000
1	-1.831441000	-1.149201000	3.138385000
6	-1.788783000	-2.419289000	-0.927735000
6	-2.428513000	-3.571343000	-0.452221000
1	-2.491615000	-3.760179000	0.618880000
6	-2.982468000	-4.490679000	-1.337475000
1	-3.475702000	-5.380362000	-0.951534000
6	-2.898328000	-4.277426000	-2.711817000
1	-3.327454000	-4.999545000	-3.403170000
6	-2.252056000	-3.144081000	-3.195829000
1	-2.172097000	-2.974814000	-4.267666000
6	-1.699747000	-2.222860000	-2.308843000
1	-1.188817000	-1.341056000	-2.692600000
6	-0.105460000	3.859224000	-0.164543000
6	-0.908355000	4.998267000	-0.062894000
1	-1.994346000	4.911637000	-0.080710000
6	-0.314631000	6.250491000	0.051619000
1	-0.940982000	7.135697000	0.137780000
6	1.073600000	6.373869000	0.039471000
1	1.533046000	7.356265000	0.122279000
6	1.868893000	5.239284000	-0.085244000
1	2.953720000	5.328798000	-0.100863000
6	1.281386000	3.980344000	-0.183161000
1	1.889119000	3.081787000	-0.272857000
1	1.851648000	1.151290000	-1.219677000
8	6.325050000	-1.072862000	-1.303045000
6	7.208031000	0.021436000	-1.496300000
1	8.246651000	-0.251659000	-1.269582000
1	7.160834000	0.410412000	-2.522183000
1	6.906345000	0.816328000	-0.806496000
1	6.569360000	-1.774101000	-1.920219000
8	3.465976000	-3.213087000	-1.288636000
6	2.451652000	-3.589569000	-2.202889000
1	2.016766000	-4.566415000	-1.950082000
1	1.640710000	-2.846436000	-2.253155000
1	2.907360000	-3.666110000	-3.195116000
1	3.078815000	-3.110053000	-0.399810000

$TS_{5\text{-}6}$-Na
E (BS1) = -3024.484832
E (BS2) = -4165.046278
$G_{298.15,\ 1M}$ (BS2) = -4164.279441

1	2.064371000	0.576815000	3.553086000
1	1.232193000	-1.012485000	-0.671170000
1	-0.354370000	-0.011444000	-1.852393000
11	3.494237000	-1.066506000	-1.346611000
1	3.253285000	-1.205013000	1.285102000
8	3.539857000	-2.087633000	0.870029000
6	4.909977000	-2.348292000	1.087650000
1	5.188513000	-3.241292000	0.513292000
1	5.552278000	-1.517573000	0.753631000
1	5.132883000	-2.550752000	2.145913000
1	1.671383000	-0.092750000	-2.734142000
8	2.300694000	-0.494348000	-3.370221000
6	3.073137000	0.554165000	-3.931471000
1	3.669866000	0.136309000	-4.748401000
1	2.440190000	1.352122000	-4.342243000
1	3.761881000	1.005260000	-3.197550000
8	2.728902000	0.263682000	1.621040000
6	2.980932000	0.685131000	2.927673000
6	3.378378000	2.155477000	2.970860000
6	4.061911000	-0.162473000	3.588371000
1	2.601370000	2.787339000	2.520155000
1	4.311004000	2.307892000	2.405208000
1	3.546087000	2.506306000	3.998334000
1	4.253009000	0.153185000	4.623037000
1	3.769701000	-1.221297000	3.608447000
1	5.005664000	-0.075419000	3.027883000
8	4.520232000	0.765933000	-0.192481000
6	4.504662000	2.099282000	-0.645264000
1	3.858568000	0.670975000	0.568169000
1	5.275364000	2.223174000	-1.417358000
1	3.533171000	2.384415000	-1.084746000
1	4.733503000	2.813725000	0.161941000
1	0.215776000	1.307326000	2.189216000
1	0.193813000	1.646295000	2.857488000
26	-4.317561000	0.374748000	-0.297243000
77	0.360968000	0.334224000	-0.516835000
15	-1.239943000	-0.961256000	0.607688000
16	-1.062590000	2.340523000	-0.573224000
6	-2.828776000	-0.161932000	0.988472000
6	-3.093607000	1.255774000	1.066548000
6	-4.463826000	1.414336000	1.445326000
1	-4.969683000	2.369271000	1.551777000
6	-5.048548000	0.126983000	1.596978000
1	-6.087660000	-0.078196000	1.831052000
6	-4.050926000	-0.842383000	1.310487000
1	-4.191577000	-1.918662000	1.301668000
6	-4.301019000	1.502071000	-2.002881000
1	-3.834909000	2.477896000	-2.092141000
6	-3.666379000	0.246771000	-2.231422000
1	-2.629534000	0.104310000	-2.521402000
6	-4.612678000	-0.784689000	-1.951571000
1	-4.422756000	-1.852900000	-1.991868000
6	-5.832224000	-0.163675000	-1.553560000
1	-6.734149000	-0.675441000	-1.235075000
6	-5.639256000	1.248607000	-1.583549000
1	-6.368300000	1.997504000	-1.293145000
6	-2.187534000	2.427111000	0.895049000
1	-1.548258000	2.570707000	1.774825000
1	-2.792874000	3.332404000	0.762232000

6	-0.642432000	-1.526049000	2.250009000
6	0.546851000	-2.265616000	2.279789000
1	1.039090000	-2.539422000	1.345671000
6	1.104546000	-2.657040000	3.491035000
1	2.025678000	-3.238053000	3.498485000
6	0.488982000	-2.298215000	4.689909000
1	0.928768000	-2.596809000	5.639189000
6	-0.690310000	-1.559924000	4.668556000
1	-1.176602000	-1.281164000	5.601188000
6	-1.257846000	-1.177413000	3.453706000
1	-2.180401000	-0.597500000	3.451039000
6	-1.751650000	-2.539223000	-0.189339000
6	-2.183946000	-3.632861000	0.569635000
1	-2.208161000	-3.568314000	1.657223000
6	-2.582891000	-4.811837000	-0.053904000
1	-2.915243000	-5.654434000	0.548718000
6	-2.550453000	-4.914300000	-1.442599000
1	-2.856584000	-5.838527000	-1.927998000
6	-2.116759000	-3.834218000	-2.206662000
1	-2.079858000	-3.909496000	-3.291534000
6	-1.720218000	-2.654586000	-1.582519000
1	-1.368954000	-1.818509000	-2.185923000
6	-0.128290000	3.837888000	-0.223790000
6	-0.725305000	5.073320000	-0.479549000
1	-1.737113000	5.118321000	-0.880886000
6	-0.018631000	6.244130000	-0.223764000
1	-0.483173000	7.207671000	-0.421271000
6	1.280953000	6.181920000	0.274583000
1	1.832887000	7.098952000	0.468505000
6	1.874461000	4.947228000	0.521919000
1	2.889542000	4.894432000	0.912583000
6	1.170988000	3.770705000	0.276527000
1	1.627582000	2.798232000	0.463885000
1	1.523805000	1.163908000	-1.387545000
8	5.442299000	-1.476321000	-2.533085000
6	6.519675000	-0.554902000	-2.591474000
1	7.450104000	-1.041307000	-2.911414000
1	6.309857000	0.282456000	-3.271759000
1	6.667636000	-0.158462000	-1.582436000
1	5.308754000	-1.846384000	-3.414972000
8	2.866566000	-3.480243000	-1.394306000
6	1.544283000	-3.802668000	-1.790340000
1	1.093489000	-4.564816000	-1.137695000
1	0.892480000	-2.917435000	-1.797187000
1	1.584052000	-4.205227000	-2.808027000
1	2.862751000	-3.234992000	-0.449410000

6-Na

E (BS1) = -3024.489450
E (BS2) = -4165.050015
$G_{298.15, 1M}$ (BS2) = -4164.284615

1	2.208552000	0.744343000	2.685245000
1	1.020616000	-0.587246000	-1.164587000
1	-0.848335000	0.010566000	-2.089660000
11	3.938021000	-0.855581000	-0.891423000
1	3.851947000	-1.266884000	1.360870000
8	4.126658000	-2.209149000	1.144375000
6	5.399949000	-2.438412000	1.706254000
1	5.864699000	-3.293563000	1.200708000
1	6.063912000	-1.570044000	1.581367000
1	5.347320000	-2.676852000	2.780952000
1	2.225638000	0.701000000	-2.197098000
8	3.173499000	0.734142000	-2.446631000
6	3.648315000	2.018276000	-2.075480000
1	4.652228000	2.144117000	-2.495971000
1	3.009671000	2.817570000	-2.477567000
1	3.710550000	2.127867000	-0.980407000
8	3.524962000	0.327360000	1.150347000
6	3.246533000	0.982251000	2.343557000
6	3.328041000	2.495679000	2.176152000
6	4.191716000	0.533925000	3.453946000
1	2.632781000	2.847586000	1.400619000
1	4.346194000	2.784369000	1.870424000
1	3.091413000	3.028360000	3.107542000
1	3.954494000	1.005971000	4.417602000
1	4.144691000	-0.554920000	3.593008000
1	5.229356000	0.795541000	3.192418000
8	5.840241000	0.525488000	0.002324000
6	6.511216000	1.761032000	0.010709000
1	5.027902000	0.585163000	0.592643000
1	7.275393000	1.756862000	-0.776683000
1	5.836338000	2.610328000	-0.185663000
1	7.023669000	1.952933000	0.967054000
1	1.331738000	1.105185000	0.267471000
1	0.647616000	1.259863000	0.727797000
26	-4.470970000	-0.379049000	-0.052665000
77	-0.046256000	0.565875000	-0.820986000
15	-1.117740000	-1.014661000	0.570283000
16	-1.847601000	2.263231000	-0.614893000
6	-2.806007000	-0.599048000	1.092456000
6	-3.328724000	0.738017000	1.225029000
6	-4.666649000	0.629837000	1.714281000
1	-5.333534000	1.468912000	1.887518000
6	-4.974817000	-0.746946000	1.893087000
1	-5.930709000	-1.150674000	2.209241000
6	-3.836101000	-1.506196000	1.510192000
1	-3.773668000	-2.589137000	1.489088000
6	-4.855027000	0.664328000	-1.774670000
1	-4.627962000	1.713396000	-1.929341000
6	-3.981158000	-0.429495000	-2.040342000
1	-2.968363000	-0.354615000	-2.424731000
6	-4.646170000	-1.630933000	-1.653297000
1	-4.228636000	-2.633004000	-1.691360000
6	-5.933158000	-1.276804000	-1.152387000
1	-6.666805000	-1.961258000	-0.739952000
6	-6.060964000	0.141168000	-1.225102000
1	-6.910703000	0.722457000	-0.883585000
6	-2.625740000	2.039954000	1.047577000
1	-1.820757000	2.163346000	1.786516000
1	-3.340854000	2.861650000	1.171453000
6	-0.269219000	-1.292872000	2.174691000
6	0.978346000	-1.927797000	2.173991000
1	1.408029000	-2.291420000	1.238695000
6	1.677724000	-2.104360000	3.362217000
1	2.642423000	-2.608785000	3.345195000
6	1.152358000	-1.625930000	4.561742000
1	1.705378000	-1.757417000	5.489551000
6	-0.079059000	-0.978868000	4.567308000
1	-0.495764000	-0.604200000	5.499943000
6	-0.791717000	-0.817038000	3.379930000
1	-1.761171000	-0.319637000	3.400298000
6	-1.288272000	-2.717243000	-0.096462000
6	-1.544705000	-3.797647000	0.757212000
1	-1.602019000	-3.640762000	1.834059000
6	-1.722497000	-5.076639000	0.241010000
1	-1.923392000	-5.907273000	0.914047000
6	-1.635782000	-5.294341000	-1.132728000

1	-1.769572000	-6.296379000	-1.534491000
6	-1.366055000	-4.230100000	-1.987661000
1	-1.287297000	-4.395669000	-3.060218000
6	-1.192993000	-2.947213000	-1.472276000
1	-0.981805000	-2.115968000	-2.143534000
6	-1.193852000	3.920633000	-0.346904000
6	-2.106578000	4.977666000	-0.310992000
1	-3.170669000	4.786349000	-0.443807000
6	-1.650723000	6.275130000	-0.107777000
1	-2.362989000	7.096593000	-0.076652000
6	-0.287897000	6.521898000	0.045133000
1	0.066284000	7.538703000	0.199144000
6	0.619088000	5.468394000	-0.006584000
1	1.685091000	5.655801000	0.106581000
6	0.170285000	4.163765000	-0.201558000
1	0.878150000	3.339422000	-0.253751000
1	0.648113000	1.548057000	-1.968092000
8	5.530177000	-1.843584000	-2.327575000
6	6.053440000	-0.945091000	-3.293944000
1	6.865270000	-1.401792000	-3.874869000
1	5.280637000	-0.587256000	-3.988409000
1	6.461792000	-0.085222000	-2.753106000
1	5.149427000	-2.601652000	-2.789203000
8	2.538945000	-2.575246000	-1.805940000
6	2.131240000	-2.441933000	-3.158440000
1	1.661000000	-3.361009000	-3.532880000
1	1.433481000	-1.602717000	-3.297988000
1	3.029088000	-2.246035000	-3.754996000
1	1.748367000	-2.722775000	-1.267922000

$TS_{6\text{-}7}$-Na
E (BS1) = -3024.482204
E (BS2) = -4165.042405
$G_{298.15,\ 1M}$ (BS2) = -4164.277682

1	1.989622000	1.085539000	2.873213000
1	1.147482000	-0.705940000	-1.050348000
1	-0.643727000	0.034359000	-2.102672000
11	3.741178000	-1.018579000	-0.945486000
1	3.508473000	-1.248826000	1.419883000
8	3.787431000	-2.154522000	1.131428000
6	5.034910000	-2.485770000	1.707030000
1	5.344102000	-3.458630000	1.309737000
1	5.815925000	-1.752006000	1.459886000
1	4.977055000	-2.573891000	2.802398000
1	2.275609000	0.630943000	-2.341691000
8	3.219830000	0.598404000	-2.608566000
6	3.765265000	1.866936000	-2.284102000
1	4.811379000	1.878995000	-2.609599000
1	3.235046000	2.679917000	-2.800430000
1	3.736730000	2.059759000	-1.199237000
8	3.015101000	0.445822000	1.202811000
6	3.011210000	1.132149000	2.429010000
6	3.348022000	2.603373000	2.227741000
6	3.970683000	0.488036000	3.415860000
1	2.647542000	3.070472000	1.521961000
1	4.363422000	2.711138000	1.816547000
1	3.303291000	3.165001000	3.170335000
1	3.965292000	1.010719000	4.381382000
1	3.702268000	-0.560758000	3.601100000
1	4.998322000	0.516501000	3.022943000
8	5.445224000	0.472750000	0.067502000
6	6.273720000	1.611912000	0.078998000
1	4.615851000	0.654292000	0.579848000
1	7.112774000	1.440037000	-0.605776000
1	5.749047000	2.520117000	-0.256004000
1	6.695873000	1.809665000	1.076269000
1	1.698996000	0.745783000	0.700947000
1	0.869944000	1.120584000	0.768738000
26	-4.429027000	-0.176692000	-0.150366000
77	0.103651000	0.489468000	-0.741884000
15	-1.128200000	-1.022863000	0.568743000
16	-1.605953000	2.258664000	-0.605783000
6	-2.810627000	-0.511473000	1.032850000
6	-3.254459000	0.852416000	1.170199000
6	-4.609175000	0.822230000	1.623224000
1	-5.227784000	1.698931000	1.789412000
6	-5.005374000	-0.535379000	1.776510000
1	-5.991643000	-0.883629000	2.064367000
6	-3.904203000	-1.358081000	1.414183000
1	-3.904891000	-2.442771000	1.383659000
6	-4.650861000	0.900396000	-1.878253000
1	-4.315112000	1.922531000	-2.017243000
6	-3.877193000	-0.272397000	-2.117977000
1	-2.846771000	-0.293583000	-2.460872000
6	-4.668708000	-1.404485000	-1.760541000
1	-4.350324000	-2.442245000	-1.789771000
6	-5.932513000	-0.928679000	-1.302790000
1	-6.743131000	-1.539867000	-0.920424000
6	-5.920621000	0.495231000	-1.373987000
1	-6.722465000	1.155021000	-1.060380000
6	-2.466734000	2.108091000	1.020853000
1	-1.690070000	2.186484000	1.795847000
1	-3.134706000	2.972939000	1.108055000
6	-0.381515000	-1.339248000	2.218241000
6	0.825021000	-2.044199000	2.279942000
1	1.272838000	-2.441398000	1.367865000
6	1.462363000	-2.244761000	3.499111000
1	2.394087000	-2.807348000	3.532767000
6	0.914182000	-1.722026000	4.669676000
1	1.417791000	-1.872490000	5.622330000
6	-0.277664000	-1.006658000	4.614171000
1	-0.712065000	-0.596701000	5.523694000
6	-0.927613000	-0.819930000	3.394956000
1	-1.866058000	-0.266585000	3.368312000
6	-1.389241000	-2.722671000	-0.081824000
6	-1.714763000	-3.783964000	0.771939000
1	-1.777528000	-3.619428000	1.847306000
6	-1.956312000	-5.053604000	0.258164000
1	-2.208378000	-5.869841000	0.931758000
6	-1.869733000	-5.280300000	-1.114040000
1	-2.054649000	-6.274982000	-1.513946000
6	-1.536471000	-4.234856000	-1.969970000
1	-1.459182000	-4.408422000	-3.041355000
6	-1.295466000	-2.962222000	-1.456166000
1	-1.032410000	-2.143701000	-2.125960000
6	-0.917444000	3.904568000	-0.358453000
6	-1.801874000	4.986123000	-0.380080000
1	-2.867474000	4.817938000	-0.532263000
6	-1.316149000	6.277841000	-0.213927000
1	-2.006575000	7.118238000	-0.228649000
6	0.049735000	6.494659000	-0.042308000
1	0.428194000	7.507040000	0.080025000
6	0.928760000	5.416448000	-0.035691000
1	1.996925000	5.581067000	0.092179000
6	0.448815000	4.117423000	-0.191534000
1	1.130114000	3.268640000	-0.198097000
1	0.980743000	1.519844000	-1.710196000

8	5.479559000	-1.998285000	-2.161907000
6	6.147174000	-1.079678000	-3.013738000
1	7.030195000	-1.531172000	-3.484405000
1	5.486352000	-0.692224000	-3.801677000
1	6.480593000	-0.241689000	-2.392733000
1	5.168228000	-2.738847000	-2.698058000
8	2.454586000	-2.814928000	-1.877946000
6	2.115714000	-2.614956000	-3.241477000
1	1.602858000	-3.488851000	-3.665077000
1	1.479277000	-1.728830000	-3.381791000
1	3.049699000	-2.464053000	-3.794094000
1	1.632859000	-2.909078000	-1.375113000

7-Na

E (BS1) = -3024.512521

E (BS2) = -4165.069808

$G_{298.15, 1M}$ (BS2) = -4164.297817

1	2.114473000	0.958716000	3.669782000
1	1.592699000	-0.212126000	-0.310599000
1	0.044525000	0.187783000	-1.990934000
11	3.660534000	-0.722061000	-1.226216000
1	3.642375000	-1.055570000	1.435567000
8	4.065451000	-1.766889000	0.912133000
6	5.417201000	-1.858932000	1.321893000
1	5.912334000	-2.608544000	0.695918000
1	5.953076000	-0.905244000	1.203065000
1	5.507812000	-2.181086000	2.369572000
1	1.748653000	0.418196000	-2.566808000
8	2.584792000	0.329352000	-3.078846000
6	2.833837000	1.568445000	-3.712892000
1	3.846858000	1.545148000	-4.130069000
1	2.131557000	1.753520000	-4.538746000
1	2.765681000	2.412259000	-3.010312000
8	3.027603000	0.609496000	1.853006000
6	3.048727000	1.226306000	3.144711000
6	3.140925000	2.729591000	2.993533000
6	4.228830000	0.645667000	3.886368000
1	2.297271000	3.118376000	2.408597000
1	4.072849000	2.999307000	2.476228000
1	3.135740000	3.225842000	3.971518000
1	4.296641000	1.061030000	4.898238000
1	4.143244000	-0.445536000	3.968046000
1	5.162492000	0.883567000	3.356608000
8	4.713063000	1.157999000	-0.240434000
6	4.564779000	2.502853000	-0.656589000
1	4.251472000	1.063308000	0.620514000
1	4.919534000	2.582877000	-1.689993000
1	3.513899000	2.832093000	-0.619663000
1	5.166948000	3.188033000	-0.042236000
1	2.170742000	0.807693000	1.401061000
1	0.571441000	1.361024000	1.165125000
26	-4.252633000	-0.679971000	-0.068717000
77	0.280585000	0.730441000	-0.396362000
15	-0.868030000	-1.016599000	0.650490000
16	-1.732009000	2.095062000	-0.654551000
6	-2.602095000	-0.704294000	1.122052000
6	-3.247141000	0.584606000	1.175812000
6	-4.582937000	0.379452000	1.641601000
1	-5.327372000	1.161995000	1.753593000
6	-4.773260000	-1.009834000	1.880078000
1	-5.696990000	-1.482977000	2.195853000
6	-3.561498000	-1.678464000	1.558745000
1	-3.400707000	-2.750927000	1.594863000
6	-4.624121000	0.276045000	-1.839176000
1	-4.439382000	1.329860000	-2.020744000
6	-3.687948000	-0.781352000	-2.031507000
1	-2.667533000	-0.672328000	-2.385675000
6	-4.307303000	-2.000388000	-1.623047000
1	-3.839627000	-2.980708000	-1.610998000
6	-5.627849000	-1.694116000	-1.181292000
1	-6.342442000	-2.399435000	-0.770385000
6	-5.822837000	-0.287791000	-1.313366000
1	-6.711966000	0.261962000	-1.023456000
6	-2.691009000	1.942696000	0.915860000
1	-2.013754000	2.256989000	1.723394000
1	-3.513720000	2.666269000	0.848388000
6	-0.107287000	-1.418146000	2.278736000
6	1.103206000	-2.117948000	2.311725000
1	1.532659000	-2.507533000	1.387677000
6	1.779195000	-2.307144000	3.512520000
1	2.722159000	-2.852295000	3.519382000
6	1.254577000	-1.794914000	4.698176000
1	1.785498000	-1.938204000	5.637166000
6	0.049644000	-1.097627000	4.674562000
1	-0.367778000	-0.697199000	5.596293000
6	-0.628453000	-0.909809000	3.471458000
1	-1.566131000	-0.353248000	3.465712000
6	-0.972735000	-2.679644000	-0.141929000
6	-1.214025000	-3.841279000	0.602684000
1	-1.293884000	-3.788768000	1.688012000
6	-1.347013000	-5.072576000	-0.031340000
1	-1.534534000	-5.966308000	0.560162000
6	-1.232720000	-5.163008000	-1.417358000
1	-1.333106000	-6.127421000	-1.910626000
6	-0.980313000	-4.017660000	-2.166360000
1	-0.881974000	-4.080809000	-3.248481000
6	-0.849742000	-2.783585000	-1.530649000
1	-0.646042000	-1.884970000	-2.115128000
6	-1.435146000	3.868777000	-0.534495000
6	-2.375023000	4.718347000	-1.119946000
1	-3.239425000	4.299181000	-1.634220000
6	-2.200249000	6.096962000	-1.044311000
1	-2.929896000	6.759618000	-1.504442000
6	-1.092479000	6.623602000	-0.384890000
1	-0.954444000	7.701236000	-0.330372000
6	-0.159810000	5.772156000	0.201983000
1	0.705559000	6.182532000	0.718214000
6	-0.326773000	4.391897000	0.128657000
1	0.399874000	3.715587000	0.576146000
1	1.269869000	1.937777000	-0.981896000
8	5.505557000	-1.560540000	-2.370765000
6	5.794533000	-0.754915000	-3.504953000
1	6.715232000	-1.079339000	-4.006750000
1	4.972703000	-0.761315000	-4.234527000
1	5.943573000	0.270844000	-3.150428000
1	5.348549000	-2.464624000	-2.673207000
8	2.479022000	-2.702482000	-1.846920000
6	2.291288000	-3.023437000	-3.214668000
1	2.084624000	-4.092639000	-3.358946000
1	1.479607000	-2.439441000	-3.670813000
1	3.223137000	-2.784004000	-3.739086000
1	1.639735000	-2.842810000	-1.384491000

B4. Potassium system

1-K

E (BS1) = -3267.835241

E (BS2) = -4408.348321
$G_{298.15,1M}$ (BS2) = -4407.688999

19	3.848732000	-0.575519000	-0.120530000
1	2.204056000	-3.116164000	1.169109000
8	3.128629000	-2.851782000	1.292108000
6	3.251610000	-2.266427000	2.574801000
1	4.301755000	-1.975846000	2.706846000
1	2.618913000	-1.370726000	2.683086000
1	2.996612000	-2.969207000	3.380813000
1	4.397455000	1.311850000	-2.671381000
1	2.467191000	-2.645583000	-2.027102000
8	3.433451000	-2.586274000	-2.037102000
8	4.873183000	1.388403000	-1.834462000
6	3.811782000	-1.825097000	-3.169471000
1	4.884053000	-1.613525000	-3.083321000
1	3.645120000	-2.368717000	-4.109907000
1	3.267170000	-0.869158000	-3.226230000
6	4.512036000	2.627423000	-1.245947000
1	5.088907000	2.737080000	-0.321409000
1	4.752414000	3.476830000	-1.900519000
1	3.438130000	2.668410000	-1.003620000
26	-4.046254000	1.025813000	0.403659000
77	0.444921000	0.161126000	-0.813105000
15	-1.306039000	-1.099957000	0.076195000
16	-0.581440000	2.299377000	-0.164343000
6	-2.545985000	-0.183349000	1.062714000
6	-2.392668000	1.149728000	1.594326000
6	-3.571407000	1.458181000	2.340377000
1	-3.762905000	2.407020000	2.832405000
6	-4.448503000	0.340119000	2.286057000
1	-5.442396000	0.285761000	2.717330000
6	-3.824250000	-0.666861000	1.502144000
1	-4.262703000	-1.623852000	1.240586000
6	-4.062485000	2.590240000	-0.916917000
1	-3.381758000	3.433917000	-0.882257000
6	-3.836663000	1.361462000	-1.602954000
1	-2.952694000	1.108272000	-2.180449000
6	-4.948701000	0.503730000	-1.350200000
1	-5.061207000	-0.517314000	-1.702508000
6	-5.861195000	1.205522000	-0.509158000
1	-6.787235000	0.811272000	-0.104238000
6	-5.312491000	2.493525000	-0.239737000
1	-5.749773000	3.250338000	0.402749000
6	-1.216923000	2.067794000	1.537815000
1	-0.397693000	1.666616000	2.149991000
1	-1.502678000	3.054637000	1.923598000
6	-0.735019000	-2.348238000	1.309484000
6	-0.167059000	-3.550792000	0.870388000
1	-0.122367000	-3.775409000	-0.196211000
6	0.333735000	-4.473754000	1.786219000
1	0.759415000	-5.409932000	1.430173000
6	0.290172000	-4.198134000	3.152961000
1	0.681815000	-4.918529000	3.867867000
6	-0.258964000	-2.998681000	3.595774000
1	-0.296704000	-2.775516000	4.660155000
6	-0.769999000	-2.080418000	2.680344000
1	-1.200697000	-1.146618000	3.043471000
6	-2.376763000	-2.138242000	-1.015495000
6	-3.147014000	-3.195169000	-0.512276000
1	-3.084976000	-3.465226000	0.541354000
6	-3.990151000	-3.918460000	-1.349390000
1	-4.582060000	-4.736197000	-0.943259000
6	-4.069252000	-3.602921000	-2.704678000
1	-4.725629000	-4.172464000	-3.359325000
6	-3.295745000	-2.566946000	-3.218646000
1	-3.341930000	-2.322993000	-4.278091000
6	-2.453127000	-1.841844000	-2.378245000
1	-1.838610000	-1.036792000	-2.781288000
6	0.721260000	3.503976000	0.106204000
6	1.099979000	4.246555000	-1.015165000
1	0.593426000	4.086731000	-1.966820000
6	2.122091000	5.183563000	-0.917330000
1	2.411052000	5.755311000	-1.796709000
6	2.768696000	5.392520000	0.299090000
1	3.564883000	6.129228000	0.376710000
6	2.380929000	4.660944000	1.417568000
1	2.873491000	4.824369000	2.374354000
6	1.359589000	3.713727000	1.329565000
1	1.074103000	3.155623000	2.219518000
1	1.162838000	-1.207237000	-1.292464000
1	1.798050000	0.931508000	-1.387025000
1	1.251820000	0.041149000	0.678737000
1	-0.192453000	0.321513000	-2.381647000
8	3.801140000	1.481357000	1.802662000
6	3.289181000	1.345975000	3.115996000
1	3.314869000	2.295946000	3.668801000
1	2.255931000	0.963386000	3.122161000
1	3.923993000	0.631643000	3.649985000
1	3.251725000	2.132337000	1.339949000
8	6.292258000	-1.650907000	-0.530465000
6	6.358885000	-3.061149000	-0.418046000
1	7.298435000	-3.391907000	0.045774000
1	5.531505000	-3.382400000	0.223784000
1	6.254315000	-3.561472000	-1.390874000
1	7.011689000	-1.359015000	-1.103044000

2-K

E (BS1) = -3460.891508
E (BS2) = -4601.481785
G298.15,1M (BS2) = -4600.739842

19	3.606669000	-1.037973000	-0.650219000
1	1.811528000	-1.779417000	1.730122000
8	2.532990000	-2.385995000	1.491484000
6	3.362890000	-2.496405000	2.628578000
1	4.146826000	-3.228553000	2.408122000
1	3.844230000	-1.539068000	2.886938000
1	2.807405000	-2.850905000	3.510933000
1	4.750726000	0.647542000	-3.204694000
1	1.971477000	-3.326841000	-2.038245000
8	2.938477000	-3.353958000	-2.055117000
8	5.043140000	0.657766000	-2.284387000
6	3.367818000	-2.928874000	-3.337370000
1	4.462502000	-2.883162000	-3.323133000
1	3.068259000	-3.630677000	-4.127403000
1	2.977355000	-1.932832000	-3.600513000
6	4.705639000	1.922733000	-1.735086000
1	5.043793000	1.931048000	-0.691657000
1	5.210247000	2.743374000	-2.263582000
1	3.619725000	2.105933000	-1.751090000
26	-3.743426000	1.327548000	0.866270000
77	0.274645000	-0.027187000	-1.487541000
15	-1.507492000	-1.072661000	-0.402485000
16	-0.436689000	2.204583000	-0.697050000
6	-2.282102000	-0.099264000	0.942379000
6	-1.811973000	1.153190000	1.484607000
6	-2.661929000	1.496460000	2.582632000

1	-2.583573000	2.409529000	3.165692000
6	-3.651850000	0.484495000	2.725921000
1	-4.468551000	0.486228000	3.440039000
6	-3.426918000	-0.489376000	1.716283000
1	-4.034671000	-1.370740000	1.539199000
6	-3.918830000	3.040308000	-0.236639000
1	-3.140920000	3.790707000	-0.331288000
6	-4.083192000	1.895768000	-1.069557000
1	-3.455956000	1.627828000	-1.913899000
6	-5.184678000	1.138947000	-0.569804000
1	-5.541707000	0.193347000	-0.966399000
6	-5.701503000	1.819388000	0.571226000
1	-6.516475000	1.479983000	1.201785000
6	-4.918982000	2.993225000	0.777569000
1	-5.034953000	3.700973000	1.591586000
6	-0.633923000	1.989353000	1.110360000
1	0.288191000	1.517885000	1.472528000
1	-0.736927000	2.985824000	1.560939000
6	-0.962231000	-2.583123000	0.507137000
6	-0.585154000	-3.710633000	-0.233452000
1	-0.681637000	-3.702794000	-1.320294000
6	-0.095606000	-4.845780000	0.402884000
1	0.186213000	-5.716061000	-0.186837000
6	0.031681000	-4.870243000	1.791582000
1	0.415601000	-5.758217000	2.289365000
6	-0.337108000	-3.754406000	2.534973000
1	-0.244714000	-3.765565000	3.620347000
6	-0.833094000	-2.618052000	1.896809000
1	-1.117460000	-1.752000000	2.493455000
6	-2.988971000	-1.722798000	-1.298560000
6	-3.804409000	-2.724417000	-0.756761000
1	-3.541551000	-3.189377000	0.192882000
6	-4.948950000	-3.145011000	-1.426689000
1	-5.571895000	-3.925222000	-0.994050000
6	-5.291495000	-2.574900000	-2.651436000
1	-6.184682000	-2.907025000	-3.176435000
6	-4.479108000	-1.590744000	-3.207032000
1	-4.733207000	-1.150684000	-4.169205000
6	-3.332391000	-1.172140000	-2.535371000
1	-2.687811000	-0.411605000	-2.977125000
6	0.991541000	3.292445000	-0.751863000
6	1.202263000	3.957478000	-1.963639000
1	0.511289000	3.805996000	-2.793030000
6	2.292907000	4.806089000	-2.110386000
1	2.448995000	5.320132000	-3.056424000
6	3.174303000	5.006447000	-1.048983000
1	4.023845000	5.676172000	-1.162204000
6	2.959788000	4.345726000	0.155825000
1	3.644751000	4.493058000	0.989450000
6	1.872294000	3.487110000	0.311632000
1	1.737738000	2.972186000	1.260260000
1	0.832142000	-1.453490000	-1.992671000
1	1.678061000	0.579968000	-2.135435000
1	1.123629000	-0.252758000	-0.047050000
1	-0.436979000	0.199113000	-3.029810000
8	3.541886000	0.790065000	1.435875000
6	4.118353000	1.576248000	2.455155000
1	3.510437000	2.463719000	2.693150000
1	4.266752000	1.007664000	3.387379000
1	5.098180000	1.927773000	2.111776000
1	2.676134000	0.490972000	1.772908000
8	5.973561000	-1.939270000	0.258870000
6	6.536214000	-0.799795000	0.883340000
1	7.634094000	-0.840843000	0.903060000
1	6.247424000	0.079025000	0.292939000
1	6.172322000	-0.669154000	1.912927000
1	6.190211000	-2.714568000	0.790679000
6	0.801560000	0.258718000	3.908985000
8	1.334498000	-0.215398000	2.907778000
6	0.959129000	1.697828000	4.272164000
6	-0.036899000	-0.577786000	4.816042000
1	1.340615000	2.283690000	3.430103000
1	0.123267000	-1.644039000	4.632327000
1	1.675047000	1.765230000	5.103294000
1	0.014790000	2.118038000	4.637370000
1	-1.093344000	-0.332775000	4.629976000
1	0.160417000	-0.334246000	5.866103000

$TS_{2\text{-}3}$-K

E (BS1) = -3460.875104

E (BS2) = -4601.461738

$G_{298.15,1M}$ (BS2) = -4600.724691

19	-4.117698000	-0.570067000	0.958355000
1	-4.284571000	-2.203128000	-1.172297000
8	-4.925985000	-2.773691000	-0.698403000
6	-6.225770000	-2.428912000	-1.132723000
1	-6.915671000	-3.219761000	-0.817206000
1	-6.578513000	-1.480679000	-0.698093000
1	-6.285845000	-2.350910000	-2.228093000
1	-3.690577000	1.655287000	3.434586000
1	-1.828962000	-2.229627000	1.653334000
8	-2.607650000	-2.600972000	2.115391000
8	-4.264147000	1.524150000	2.669227000
6	-2.499145000	-2.211863000	3.471755000
1	-3.330270000	-2.663297000	4.022960000
1	-1.559071000	-2.556857000	3.925822000
1	-2.551555000	-1.118116000	3.603479000
6	-4.262613000	2.718216000	1.904266000
1	-4.826841000	2.524084000	0.982654000
1	-4.753834000	3.545900000	2.434917000
1	-3.244685000	3.030509000	1.628444000
26	4.142387000	1.722851000	-0.297589000
77	-0.065517000	-0.304256000	0.668519000
15	2.034499000	-1.050517000	-0.063881000
16	0.414100000	2.004475000	0.033615000
6	3.061017000	0.141582000	-0.991190000
6	2.608575000	1.370847000	-1.592860000
6	3.719526000	1.954351000	-2.277273000
1	3.696801000	2.907617000	-2.796730000
6	4.849218000	1.105719000	-2.113597000
1	5.852158000	1.299592000	-2.478728000
6	4.451117000	-0.003545000	-1.320567000
1	5.098423000	-0.808026000	-0.987885000
6	3.629967000	3.280620000	0.923834000
1	2.749784000	3.900917000	0.789397000
6	3.691344000	2.065791000	1.666980000
1	2.865847000	1.601240000	2.197540000
6	5.010482000	1.536531000	1.540804000
1	5.366248000	0.600931000	1.961907000
6	5.763389000	2.427269000	0.721026000
1	6.790992000	2.286622000	0.403108000
6	4.910331000	3.503929000	0.339254000
1	5.176143000	4.324855000	-0.318215000
6	1.248648000	1.977609000	-1.613005000
1	0.577037000	1.443688000	-2.300539000
1	1.321371000	3.022999000	-1.939841000
6	1.958953000	-2.457708000	-1.248467000

6	1.629142000	-3.732755000	-0.772708000
1	1.478651000	-3.892475000	0.295683000
6	1.493286000	-4.801493000	-1.651816000
1	1.245353000	-5.788791000	-1.267574000
6	1.668539000	-4.607311000	-3.021278000
1	1.559508000	-5.442636000	-3.709549000
6	1.982489000	-3.340889000	-3.504296000
1	2.121319000	-3.181605000	-4.571734000
6	2.132169000	-2.271487000	-2.622331000
1	2.385762000	-1.285968000	-3.013622000
6	3.237717000	-1.694626000	1.182209000
6	4.299619000	-2.530099000	0.810732000
1	4.395986000	-2.868859000	-0.220134000
6	5.237016000	-2.945147000	1.750963000
1	6.055879000	-3.593441000	1.445850000
6	5.122862000	-2.539877000	3.079419000
1	5.853715000	-2.869707000	3.814767000
6	4.063221000	-1.723770000	3.462816000
1	3.959905000	-1.412191000	4.500281000
6	3.126875000	-1.306247000	2.519450000
1	2.294518000	-0.671640000	2.821622000
6	-1.005542000	2.984516000	-0.481299000
6	-0.885576000	4.371826000	-0.374468000
1	0.026915000	4.806857000	0.032417000
6	-1.929764000	5.191166000	-0.791831000
1	-1.837177000	6.271405000	-0.704352000
6	-3.086546000	4.625767000	-1.322057000
1	-3.902027000	5.265269000	-1.653087000
6	-3.202245000	3.242257000	-1.424666000
1	-4.111731000	2.795417000	-1.819923000
6	-2.168027000	2.412733000	-0.995480000
1	-2.266949000	1.327884000	-1.055836000
1	-0.294037000	-1.814754000	1.219455000
1	-1.557413000	0.137356000	1.252547000
1	-0.905520000	-0.735028000	-0.856014000
1	0.481077000	0.062287000	2.178309000
8	-5.265436000	0.751361000	-1.135104000
6	-6.362672000	1.204812000	-1.896285000
1	-6.047949000	1.796045000	-2.768074000
1	-6.994301000	0.377140000	-2.254571000
1	-6.976256000	1.849694000	-1.256298000
1	-4.617642000	0.311306000	-1.715068000
8	-6.608507000	-0.937580000	1.925557000
6	-7.381445000	0.192279000	1.562820000
1	-8.250281000	0.325239000	2.222860000
1	-6.743342000	1.079027000	1.665982000
1	-7.737349000	0.134695000	0.522892000
1	-7.138946000	-1.729695000	1.773570000
6	-2.000819000	-1.288670000	-2.016953000
8	-3.152637000	-0.894627000	-1.665996000
6	-1.332078000	-0.594031000	-3.174815000
6	-1.670427000	-2.747004000	-1.843072000
1	-1.491398000	0.488689000	-3.130822000
1	-1.981873000	-3.109405000	-0.856864000
1	-1.809090000	-0.966531000	-4.094805000
1	-0.261037000	-0.816049000	-3.234561000
1	-0.606859000	-2.952621000	-1.990218000
1	-2.237985000	-3.303173000	-2.605782000

3-K

E (BS1) = -3460.892269

E (BS2) = -4601.482070

$G_{298.15,1M}$ (BS2) = -4600.736831

19	4.123739000	-0.504878000	-1.129443000
1	4.328134000	-2.183200000	0.951453000
8	4.997840000	-2.819236000	0.555488000
6	6.252486000	-2.429238000	1.058903000
1	7.003508000	-3.174313000	0.767586000
1	6.585342000	-1.451836000	0.667772000
1	6.256808000	-2.371297000	2.159848000
1	3.560440000	1.829469000	-3.569121000
1	1.787853000	-2.216628000	-1.657237000
8	2.567093000	-2.581472000	-2.120106000
8	4.079663000	1.613761000	-2.784974000
6	2.426696000	-2.237007000	-3.486241000
1	3.248441000	-2.702370000	-4.039573000
1	1.479840000	-2.602298000	-3.908589000
1	2.470376000	-1.147544000	-3.653696000
6	4.120509000	2.758538000	-1.949111000
1	4.637115000	2.475436000	-1.023174000
1	4.679263000	3.584297000	-2.411039000
1	3.113672000	3.115681000	-1.687321000
26	-4.213538000	1.642918000	0.279284000
77	-0.000856000	-0.242047000	-0.725186000
15	-2.042645000	-1.061601000	0.118167000
16	-0.505297000	2.068163000	-0.029700000
6	-3.103156000	0.110333000	1.023194000
6	-2.681154000	1.364354000	1.595978000
6	-3.809502000	1.940088000	2.257091000
1	-3.810804000	2.905198000	2.754438000
6	-4.918168000	1.061698000	2.108949000
1	-5.927869000	1.242621000	2.461807000
6	-4.490656000	-0.060258000	1.349804000
1	-5.119116000	-0.885369000	1.031967000
6	-3.767182000	3.199089000	-0.972070000
1	-2.929261000	3.875244000	-0.838694000
6	-3.743104000	1.964755000	-1.684443000
1	-2.881864000	1.537404000	-2.188917000
6	-5.032139000	1.362891000	-1.569302000
1	-5.323540000	0.396195000	-1.969949000
6	-5.851204000	2.228520000	-0.787333000
1	-6.874713000	2.035457000	-0.484062000
6	-5.069439000	3.361725000	-0.417268000
1	-5.394942000	4.181564000	0.214079000
6	-1.326849000	1.982591000	1.624467000
1	-0.643500000	1.432717000	2.289102000
1	-1.402185000	3.016939000	1.982971000
6	-1.824207000	-2.403340000	1.354071000
6	-1.483058000	-3.690739000	0.921799000
1	-1.403312000	-3.905416000	-0.144454000
6	-1.254630000	-4.704954000	1.844878000
1	-1.001449000	-5.704426000	1.497594000
6	-1.345375000	-4.441269000	3.210791000
1	-1.165414000	-5.235297000	3.932174000
6	-1.663710000	-3.159302000	3.648731000
1	-1.733054000	-2.946307000	4.713502000
6	-1.905588000	-2.144103000	2.725094000
1	-2.163565000	-1.145954000	3.080391000
6	-3.213326000	-1.812927000	-1.085994000
6	-4.238532000	-2.670105000	-0.666468000
1	-4.316352000	-2.959215000	0.380952000
6	-5.161097000	-3.167919000	-1.580545000
1	-5.953016000	-3.831737000	-1.240292000
6	-5.066335000	-2.824420000	-2.927780000
1	-5.786261000	-3.217770000	-3.642266000
6	-4.041246000	-1.987754000	-3.357838000
1	-3.954124000	-1.724680000	-4.409891000

6	-3.119522000	-1.486294000	-2.441612000
1	-2.318934000	-0.832410000	-2.783809000
6	0.920208000	3.029385000	0.494286000
6	0.806004000	4.419860000	0.434844000
1	-0.106414000	4.873858000	0.049626000
6	1.858018000	5.217575000	0.873340000
1	1.772036000	6.300664000	0.823159000
6	3.013125000	4.627054000	1.379525000
1	3.833032000	5.250186000	1.730061000
6	3.121944000	3.240313000	1.434927000
1	4.028872000	2.772356000	1.810733000
6	2.082178000	2.432441000	0.980008000
1	2.180825000	1.345475000	0.998752000
1	0.224262000	-1.720676000	-1.350192000
1	1.434294000	0.255706000	-1.412286000
1	1.418917000	-0.732479000	1.127592000
1	-0.655250000	0.123156000	-2.084240000
8	5.278173000	0.859305000	1.058094000
6	6.176757000	1.101077000	2.112635000
1	5.668330000	1.470000000	3.017554000
1	6.749193000	0.201757000	2.395520000
1	6.895574000	1.868497000	1.800597000
1	4.537243000	0.281572000	1.394675000
8	6.678820000	-0.834340000	-1.939651000
6	7.403726000	0.330509000	-1.586319000
1	8.327733000	0.432420000	-2.171936000
1	6.767027000	1.196702000	-1.802828000
1	7.661627000	0.348921000	-0.517478000
1	7.187480000	-1.604476000	-1.657197000
6	2.168349000	-1.192030000	1.857707000
8	3.452698000	-0.935732000	1.458590000
6	1.852858000	-0.575172000	3.217854000
6	1.872654000	-2.687290000	1.869150000
1	2.068059000	0.502714000	3.217126000
1	1.994677000	-3.120499000	0.865735000
1	2.486451000	-1.039747000	3.988045000
1	0.801584000	-0.721726000	3.507537000
1	0.855331000	-2.904252000	2.219110000
1	2.580555000	-3.192809000	2.543115000

4-K

E (BS1) = -3460.912045

E (BS2) = -4601.499135

$G_{298.15,1M}$ (BS2) = -4600.747812

1	-0.086824000	0.862742000	2.847229000
1	1.618746000	-0.779989000	-0.511401000
1	0.046467000	0.150967000	-1.862662000
19	4.276553000	-0.176082000	-0.793631000
1	2.626982000	-0.576985000	1.728570000
8	3.312114000	-1.289575000	1.574762000
6	4.081515000	-1.494093000	2.735822000
1	4.796544000	-2.301564000	2.539186000
1	4.653125000	-0.598023000	3.023357000
1	3.468266000	-1.797179000	3.599420000
1	1.967212000	-0.262683000	-2.424638000
8	2.646888000	-0.428688000	-3.110540000
6	2.801674000	0.769249000	-3.845359000
1	3.411841000	0.554908000	-4.729202000
1	1.836743000	1.170137000	-4.186963000
1	3.308306000	1.561691000	-3.266476000
8	1.598891000	0.747593000	1.661265000
6	0.969164000	1.211028000	2.822574000
6	0.956944000	2.731114000	2.890753000
6	1.666029000	0.642507000	4.050490000
1	0.456083000	3.164846000	2.016702000
1	1.988740000	3.115091000	2.913309000
1	0.441180000	3.090998000	3.791821000
1	1.186336000	0.975768000	4.981035000
1	1.654232000	-0.455267000	4.038618000
1	2.715616000	0.974530000	4.070100000
8	4.023988000	1.749412000	1.220141000
6	4.005995000	3.024126000	0.622830000
1	3.082845000	1.456243000	1.393661000
1	5.017714000	3.445740000	0.654376000
1	3.688573000	2.993583000	-0.436450000
1	3.338898000	3.724748000	1.150247000
26	-4.162840000	-0.110030000	-0.467903000
77	0.568677000	0.423457000	-0.378860000
15	-0.976683000	-1.157223000	0.454270000
16	-1.193740000	2.178326000	-0.482983000
6	-2.668045000	-0.584590000	0.824849000
6	-3.086783000	0.782578000	1.019994000
6	-4.477613000	0.763637000	1.348914000
1	-5.086277000	1.647164000	1.516249000
6	-4.923515000	-0.586960000	1.368817000
1	-5.940825000	-0.920884000	1.543199000
6	-3.817428000	-1.416793000	1.044087000
1	-3.844106000	-2.496458000	0.939156000
6	-4.220292000	1.126770000	-2.095617000
1	-3.877325000	2.156348000	-2.095792000
6	-3.421994000	-0.021806000	-2.370446000
1	-2.363556000	-0.018658000	-2.612919000
6	-4.240466000	-1.179138000	-2.204617000
1	-3.914931000	-2.210900000	-2.302895000
6	-5.544741000	-0.743028000	-1.829214000
1	-6.386686000	-1.383638000	-1.588977000
6	-5.531877000	0.681065000	-1.760893000
1	-6.363145000	1.311524000	-1.463813000
6	-2.291856000	2.046131000	0.991856000
1	-1.663439000	2.146408000	1.887804000
1	-2.983755000	2.895739000	0.960619000
6	-0.495207000	-1.919811000	2.060826000
6	0.588482000	-2.805270000	2.091139000
1	1.100819000	-3.069614000	1.165452000
6	1.014685000	-3.363108000	3.291805000
1	1.853990000	-4.056644000	3.296608000
6	0.368982000	-3.034735000	4.482825000
1	0.702349000	-3.469955000	5.422581000
6	-0.704804000	-2.149714000	4.464313000
1	-1.215940000	-1.890704000	5.389470000
6	-1.137090000	-1.597613000	3.259867000
1	-1.982514000	-0.909854000	3.261054000
6	-1.263342000	-2.655030000	-0.584360000
6	-1.750058000	-3.846566000	-0.031821000
1	-1.951917000	-3.912283000	1.036644000
6	-1.968237000	-4.962597000	-0.833484000
1	-2.345465000	-5.880741000	-0.388034000
6	-1.694475000	-4.908171000	-2.198584000
1	-1.856913000	-5.784749000	-2.822213000
6	-1.204056000	-3.732069000	-2.757733000
1	-0.978909000	-3.683387000	-3.821171000
6	-0.991353000	-2.613778000	-1.954968000
1	-0.595626000	-1.699086000	-2.394234000
6	-0.676576000	3.896555000	-0.344021000
6	-1.640591000	4.907682000	-0.367426000
1	-2.700419000	4.660906000	-0.422944000
6	-1.241527000	6.239514000	-0.335539000

1	-1.994126000	7.025046000	-0.346738000
6	0.112640000	6.567602000	-0.308244000
1	0.418938000	7.611145000	-0.291716000
6	1.069936000	5.557884000	-0.310765000
1	2.129998000	5.806035000	-0.296372000
6	0.677840000	4.221643000	-0.324321000
1	1.415708000	3.420711000	-0.317156000
1	1.769167000	1.391675000	-1.008065000
8	6.473234000	-1.201817000	-2.045342000
6	6.046937000	-1.095670000	-3.395605000
1	6.755640000	-1.570477000	-4.086043000
1	5.051752000	-1.537846000	-3.555273000
1	6.001937000	-0.029468000	-3.644320000
1	6.624273000	-2.135738000	-1.852134000
8	3.562372000	-2.924240000	-0.652834000
6	2.490662000	-3.416602000	-1.433698000
1	1.973903000	-4.262568000	-0.952907000
1	1.745887000	-2.637970000	-1.655607000
1	2.904804000	-3.771413000	-2.384232000
1	3.223091000	-2.577824000	0.195331000

5-K

E (BS1) = -3462.085443

E (BS2) = -4602.67453

$G_{298.15, 1M}$ (BS2) = -4601.913121

1	-0.129532000	0.868898000	2.779211000
1	1.641254000	-0.786350000	-0.504219000
1	0.077232000	0.129855000	-1.882407000
19	4.290077000	-0.176800000	-0.765290000
1	2.615371000	-0.559863000	1.742554000
8	3.315513000	-1.263095000	1.613835000
6	4.061457000	-1.428583000	2.796594000
1	4.846281000	-2.171017000	2.610500000
1	4.548696000	-0.494791000	3.116513000
1	3.445560000	-1.795047000	3.633608000
1	1.994279000	-0.281470000	-2.423505000
8	2.684557000	-0.465750000	-3.094156000
6	2.869100000	0.717373000	-3.845613000
1	3.523278000	0.485828000	-4.693040000
1	1.920372000	1.106411000	-4.242193000
1	3.344070000	1.523724000	-3.259171000
8	1.591963000	0.762179000	1.653079000
6	0.923441000	1.226979000	2.791777000
6	0.897721000	2.747252000	2.853635000
6	1.579392000	0.672991000	4.048379000
1	0.420464000	3.175225000	1.964062000
1	1.926194000	3.137460000	2.905688000
1	0.354765000	3.107388000	3.738908000
1	1.049811000	0.993258000	4.956932000
1	1.596523000	-0.424603000	4.035516000
1	2.617995000	1.032256000	4.116896000
8	4.012298000	1.778627000	1.208543000
6	3.988321000	3.053995000	0.612387000
1	3.073419000	1.482978000	1.388118000
1	5.001244000	3.473824000	0.633050000
1	3.660379000	3.022970000	-0.443508000
1	3.327022000	3.754229000	1.147461000
1	-1.698930000	1.216944000	4.293439000
1	-2.303328000	1.502004000	4.626116000
26	-4.146127000	-0.134079000	-0.525815000
77	0.585959000	0.414873000	-0.395878000
15	-0.961357000	-1.168019000	0.430869000
16	-1.188247000	2.159106000	-0.526900000
6	-2.659094000	-0.600071000	0.777023000
6	-3.084746000	0.765473000	0.968708000
6	-4.477380000	0.740636000	1.289541000
1	-5.090807000	1.621745000	1.452499000
6	-4.917787000	-0.611846000	1.307013000
1	-5.934740000	-0.950173000	1.474928000
6	-3.806181000	-1.437075000	0.989772000
1	-3.826793000	-2.517061000	0.886254000
6	-4.191831000	1.095116000	-2.160367000
1	-3.849179000	2.124786000	-2.164740000
6	-3.390632000	-0.054117000	-2.423275000
1	-2.330410000	-0.051381000	-2.657837000
6	-4.209302000	-1.211290000	-2.257749000
1	-3.881623000	-2.243070000	-2.348325000
6	-5.516876000	-0.774332000	-1.894549000
1	-6.360051000	-1.414464000	-1.657380000
6	-5.505605000	0.650122000	-1.833385000
1	-6.339789000	1.281479000	-1.546616000
6	-2.295011000	2.032310000	0.941879000
1	-1.672871000	2.137149000	1.841695000
1	-2.988832000	2.880061000	0.901871000
6	-0.503679000	-1.915755000	2.050932000
6	0.591387000	-2.784958000	2.115121000
1	1.127412000	-3.052689000	1.204010000
6	0.999399000	-3.321047000	3.332140000
1	1.847628000	-4.002852000	3.363544000
6	0.325922000	-2.984774000	4.505699000
1	0.647695000	-3.401072000	5.457998000
6	-0.760432000	-2.116500000	4.452947000
1	-1.293483000	-1.847924000	5.363388000
6	-1.176829000	-1.590838000	3.231543000
1	-2.035073000	-0.920250000	3.204733000
6	-1.227531000	-2.673800000	-0.601475000
6	-1.711772000	-3.864961000	-0.046045000
1	-1.924083000	-3.924493000	1.020809000
6	-1.913456000	-4.988000000	-0.842335000
1	-2.289037000	-5.905694000	-0.394520000
6	-1.624720000	-4.940864000	-2.204570000
1	-1.773553000	-5.822659000	-2.824212000
6	-1.135803000	-3.765249000	-2.766168000
1	-0.899239000	-3.722225000	-3.827372000
6	-0.940649000	-2.639748000	-1.969225000
1	-0.546436000	-1.725288000	-2.410415000
6	-0.682512000	3.881667000	-0.403619000
6	-1.650649000	4.887674000	-0.456352000
1	-2.708330000	4.634252000	-0.522529000
6	-1.257606000	6.221663000	-0.439845000
1	-2.013056000	7.003735000	-0.474498000
6	0.094700000	6.556292000	-0.398462000
1	0.396314000	7.601311000	-0.394256000
6	1.056260000	5.550977000	-0.370688000
1	2.115136000	5.803246000	-0.344351000
6	0.669876000	4.213264000	-0.368960000
1	1.410379000	3.415506000	-0.337548000
1	1.785676000	1.383563000	-1.024551000
8	6.496619000	-1.193472000	-2.006582000
6	6.070807000	-1.048681000	-3.353346000
1	6.794507000	-1.475547000	-4.059505000
1	5.089433000	-1.513753000	-3.531507000
1	5.992601000	0.024624000	-3.560505000
1	6.640309000	-2.133440000	-1.838600000
8	3.595065000	-2.925790000	-0.590707000
6	2.528265000	-3.445079000	-1.361050000
1	2.000427000	-4.265389000	-0.848963000

1	1.791308000	-2.672117000	-1.624849000
1	2.950641000	-3.844705000	-2.289894000
1	3.252898000	-2.569597000	0.252235000

$TS_{5\text{-}6}$-K

E (BS1) = -3462.074770

E (BS2) = -4602.665056

$G_{298.15,\ 1M}$ (BS2) = -4601.905559

1	1.719350000	-0.135407000	3.706653000
1	1.249284000	-0.944852000	-0.624026000
1	-0.247952000	0.201489000	-1.817450000
19	3.815574000	-1.170200000	-1.598097000
1	3.046443000	-1.553427000	1.343364000
8	3.337195000	-2.414076000	0.878676000
6	4.679573000	-2.721300000	1.170957000
1	5.044005000	-3.456538000	0.438751000
1	5.333784000	-1.835676000	1.117775000
1	4.804101000	-3.169883000	2.169405000
1	1.675895000	0.369392000	-2.676196000
8	2.269314000	0.192591000	-3.435253000
6	3.038757000	1.363157000	-3.637966000
1	3.658834000	1.213977000	-4.527688000
1	2.405631000	2.245971000	-3.804126000
1	3.706727000	1.585268000	-2.786353000
8	2.614633000	-0.134272000	1.846754000
6	2.703652000	0.068670000	3.223014000
6	3.073277000	1.511744000	3.544741000
6	3.711271000	-0.876802000	3.867695000
1	2.367643000	2.214380000	3.079443000
1	4.078436000	1.735962000	3.155365000
1	3.080116000	1.705536000	4.626286000
1	3.773624000	-0.726541000	4.954099000
1	3.435694000	-1.924502000	3.688600000
1	4.712677000	-0.707628000	3.442556000
8	4.608999000	0.651758000	0.350918000
6	4.521913000	2.042978000	0.154206000
1	3.867618000	0.385899000	0.983348000
1	5.368876000	2.372607000	-0.461892000
1	3.594132000	2.341432000	-0.364694000
1	4.573100000	2.603404000	1.102228000
1	0.119906000	1.086876000	2.326506000
1	-0.046137000	1.298899000	3.026890000
26	-4.376950000	0.501142000	-0.317345000
77	0.363946000	0.381052000	-0.400277000
15	-1.344882000	-0.972251000	0.484047000
16	-1.039391000	2.417647000	-0.378939000
6	-2.911465000	-0.161173000	0.932859000
6	-3.144552000	1.253213000	1.116818000
6	-4.514360000	1.414522000	1.496190000
1	-4.999149000	2.370493000	1.670146000
6	-5.129511000	0.133948000	1.548900000
1	-6.174355000	-0.064300000	1.762296000
6	-4.152566000	-0.834074000	1.196349000
1	-4.319997000	-1.902743000	1.107846000
6	-4.303482000	1.730044000	-1.949933000
1	-3.799363000	2.690681000	-1.975702000
6	-3.714165000	0.467777000	-2.251030000
1	-2.682439000	0.302884000	-2.545689000
6	-4.699855000	-0.542899000	-2.040610000
1	-4.549798000	-1.613493000	-2.145510000
6	-5.899157000	0.098226000	-1.612891000
1	-6.821851000	-0.398393000	-1.331720000
6	-5.653856000	1.501670000	-1.555075000
1	-6.357768000	2.257550000	-1.223555000
6	-2.218305000	2.420184000	1.047130000
1	-1.609037000	2.500216000	1.955973000
1	-2.808500000	3.340365000	0.952060000
6	-0.830175000	-1.789558000	2.047325000
6	0.328710000	-2.573806000	2.003158000
1	0.855715000	-2.714732000	1.059328000
6	0.817406000	-3.178125000	3.155251000
1	1.717983000	-3.788528000	3.102180000
6	0.161607000	-2.993540000	4.371303000
1	0.546762000	-3.461132000	5.274904000
6	-0.986735000	-2.209341000	4.425060000
1	-1.504057000	-2.063420000	5.370882000
6	-1.483045000	-1.609472000	3.268284000
1	-2.382217000	-0.996709000	3.324974000
6	-1.887774000	-2.404941000	-0.537884000
6	-2.471981000	-3.536149000	0.044267000
1	-2.596525000	-3.591042000	1.125566000
6	-2.891114000	-4.601467000	-0.747435000
1	-3.344303000	-5.474061000	-0.281707000
6	-2.721695000	-4.554987000	-2.129660000
1	-3.042561000	-5.392022000	-2.746163000
6	-2.129056000	-3.440782000	-2.717426000
1	-1.980497000	-3.403063000	-3.794752000
6	-1.714544000	-2.373473000	-1.924890000
1	-1.238130000	-1.511035000	-2.389209000
6	-0.108735000	3.877562000	0.107345000
6	-0.654246000	5.134493000	-0.158299000
1	-1.623606000	5.215514000	-0.649212000
6	0.050645000	6.279014000	0.200845000
1	-0.371522000	7.259721000	-0.007444000
6	1.296238000	6.168134000	0.814770000
1	1.848064000	7.064802000	1.087826000
6	1.838279000	4.911810000	1.074198000
1	2.812251000	4.824391000	1.552747000
6	1.136715000	3.761347000	0.723211000
1	1.556151000	2.771948000	0.909619000
1	1.639509000	1.237034000	-1.052273000
8	5.871444000	-0.513452000	-3.168319000
6	6.566829000	0.608543000	-2.651884000
1	7.646088000	0.549229000	-2.847461000
1	6.189076000	1.555218000	-3.064677000
1	6.418023000	0.618231000	-1.565855000
1	5.981927000	-0.526375000	-4.126913000
8	2.716153000	-3.760142000	-1.430360000
6	1.396246000	-3.733427000	-1.938786000
1	0.704069000	-4.329657000	-1.325782000
1	0.995715000	-2.710111000	-2.013625000
1	1.408683000	-4.163333000	-2.945658000
1	2.701906000	-3.445828000	-0.503852000

6-K

E (BS1) = -3462.079752

E (BS2) = -4602.668907

$G_{298.15,\ 1M}$ (BS2) = -4601.902112

1	2.182775000	1.131817000	2.746159000
1	0.941110000	-0.627767000	-1.230807000
1	-0.965405000	-0.070431000	-2.082390000
19	3.615199000	-1.098617000	-0.897950000
1	3.968283000	-1.024850000	1.682427000
8	4.294221000	-1.973797000	1.641385000
6	5.551652000	-2.049276000	2.267525000
1	6.006159000	-3.019315000	2.032340000

1	6.237808000	-1.262659000	1.916759000
1	5.482080000	-1.975085000	3.365864000
1	1.994629000	0.766568000	-2.559172000
8	2.933420000	0.770723000	-2.836671000
6	3.522020000	1.917142000	-2.249700000
1	4.553445000	1.990172000	-2.613790000
1	2.997897000	2.841343000	-2.535269000
1	3.547570000	1.857039000	-1.147305000
8	3.464679000	0.494306000	1.258936000
6	3.215418000	1.311116000	2.351591000
6	3.291640000	2.787328000	1.974196000
6	4.180108000	1.020135000	3.497340000
1	2.588298000	3.022338000	1.162602000
1	4.305790000	3.033318000	1.622551000
1	3.059073000	3.445524000	2.822925000
1	3.986300000	1.650190000	4.376690000
1	4.108210000	-0.029907000	3.813089000
1	5.215407000	1.203117000	3.167957000
8	5.766173000	0.566687000	0.033087000
6	6.386295000	1.822372000	-0.070952000
1	4.943999000	0.657203000	0.607051000
1	7.138988000	1.786278000	-0.869467000
1	5.676072000	2.627612000	-0.324314000
1	6.906453000	2.117975000	0.855455000
1	1.303415000	1.059590000	0.178386000
1	0.631334000	1.293979000	0.621457000
26	-4.519204000	-0.396044000	0.002653000
77	-0.113485000	0.532195000	-0.867968000
15	-1.136063000	-0.994102000	0.623648000
16	-1.911713000	2.223628000	-0.662584000
6	-2.830129000	-0.584293000	1.122172000
6	-3.363694000	0.749248000	1.238080000
6	-4.690685000	0.635908000	1.755727000
1	-5.364743000	1.470161000	1.924572000
6	-4.980837000	-0.741310000	1.963544000
1	-5.926781000	-1.148643000	2.304056000
6	-3.840887000	-1.495340000	1.573640000
1	-3.760085000	-2.577801000	1.574939000
6	-4.965424000	0.645691000	-1.702822000
1	-4.757306000	1.699463000	-1.854010000
6	-4.081384000	-0.432157000	-1.998030000
1	-3.081501000	-0.338849000	-2.410520000
6	-4.715462000	-1.646348000	-1.599772000
1	-4.283168000	-2.640649000	-1.658303000
6	-5.994090000	-1.316666000	-1.062712000
1	-6.705470000	-2.015209000	-0.635154000
6	-6.147620000	0.099276000	-1.124718000
1	-6.996766000	0.664347000	-0.755600000
6	-2.675658000	2.052155000	1.013037000
1	-1.867312000	2.211122000	1.741467000
1	-3.398775000	2.870354000	1.111585000
6	-0.284376000	-1.137822000	2.244583000
6	1.004012000	-1.684261000	2.282532000
1	1.455035000	-2.079926000	1.370385000
6	1.708744000	-1.744800000	3.479330000
1	2.710099000	-2.171234000	3.483809000
6	1.144696000	-1.237988000	4.649244000
1	1.702370000	-1.275940000	5.582874000
6	-0.129482000	-0.681815000	4.617813000
1	-0.576974000	-0.286242000	5.527252000
6	-0.844840000	-0.635320000	3.422104000
1	-1.846177000	-0.206016000	3.415473000
6	-1.271888000	-2.735825000	0.059224000
6	-1.346006000	-3.793366000	0.972808000
1	-1.310392000	-3.594929000	2.043489000
6	-1.461849000	-5.105286000	0.523157000
1	-1.513742000	-5.918886000	1.243389000
6	-1.506205000	-5.375830000	-0.842370000
1	-1.590310000	-6.402600000	-1.191570000
6	-1.439555000	-4.330037000	-1.759510000
1	-1.472704000	-4.534857000	-2.827667000
6	-1.321133000	-3.015992000	-1.311059000
1	-1.256941000	-2.200092000	-2.030542000
6	-1.262441000	3.891598000	-0.462177000
6	-2.180995000	4.943910000	-0.436004000
1	-3.247432000	4.742101000	-0.528724000
6	-1.727655000	6.250503000	-0.295884000
1	-2.444163000	7.068530000	-0.272693000
6	-0.362224000	6.510676000	-0.197796000
1	-0.010055000	7.534609000	-0.094941000
6	0.549805000	5.461164000	-0.239282000
1	1.617678000	5.659159000	-0.169837000
6	0.103867000	4.147538000	-0.370093000
1	0.815209000	3.325599000	-0.414237000
1	0.518048000	1.501650000	-2.056131000
8	5.484238000	-2.031415000	-2.616431000
6	5.836945000	-0.895740000	-3.389168000
1	6.772874000	-1.050398000	-3.942507000
1	5.047479000	-0.616098000	-4.101128000
1	5.991015000	-0.062201000	-2.693667000
1	5.376603000	-2.781981000	-3.213344000
8	2.050821000	-3.184248000	-1.741345000
6	1.813693000	-2.827413000	-3.093619000
1	1.351034000	-3.647016000	-3.659982000
1	1.178269000	-1.932350000	-3.183458000
1	2.785396000	-2.607972000	-3.552674000
1	1.202508000	-3.417227000	-1.337120000

TS$_{6\text{-}7}$-K
E (BS1) = -3462.072596
E (BS2) = -4602.660770
$G_{298.15,\ 1M}$ (BS2) = -4601.900799

1	2.018843000	1.898718000	2.432453000
1	1.025062000	-0.746599000	-1.064812000
1	-0.811860000	-0.146332000	-2.068965000
19	3.526603000	-1.445914000	-0.771695000
1	3.658761000	-0.762552000	1.816729000
8	3.993505000	-1.688732000	1.910975000
6	5.224255000	-1.682936000	2.597554000
1	5.610707000	-2.708110000	2.616266000
1	5.975491000	-1.047370000	2.104146000
1	5.121891000	-1.346658000	3.641516000
1	1.998588000	0.270554000	-2.620525000
8	2.898620000	0.145942000	-2.986545000
6	3.600509000	1.349743000	-2.737720000
1	4.619070000	1.233918000	-3.126954000
1	3.138631000	2.208169000	-3.247153000
1	3.665713000	1.578815000	-1.660727000
8	3.013224000	0.726527000	1.059243000
6	3.029557000	1.794311000	1.968782000
6	3.346455000	3.102089000	1.255920000
6	4.016470000	1.543175000	3.096655000
1	2.647835000	3.274678000	0.425339000
1	4.363879000	3.066059000	0.836861000
1	3.292999000	3.963566000	1.935672000
1	4.031120000	2.376970000	3.811104000
1	3.757702000	0.630878000	3.650729000

1	5.033895000	1.425694000	2.693386000
8	5.437007000	0.378457000	-0.133123000
6	6.342516000	1.436993000	-0.333171000
1	4.631683000	0.705729000	0.341289000
1	7.168643000	1.070914000	-0.954486000
1	5.885757000	2.292330000	-0.856202000
1	6.774663000	1.804508000	0.610974000
1	1.664652000	0.886141000	0.540834000
1	0.836832000	1.259905000	0.574139000
26	-4.490946000	-0.271810000	-0.169803000
77	-0.003157000	0.465510000	-0.812870000
15	-1.159485000	-0.883949000	0.714663000
16	-1.720264000	2.244838000	-0.772192000
6	-2.877670000	-0.411963000	1.067572000
6	-3.390729000	0.933616000	1.060602000
6	-4.750368000	0.877629000	1.496618000
1	-5.416170000	1.732373000	1.567458000
6	-5.081501000	-0.478234000	1.774243000
1	-6.055408000	-0.846662000	2.078585000
6	-3.933482000	-1.274462000	1.510527000
1	-3.872994000	-2.355640000	1.588180000
6	-4.743384000	0.636839000	-1.987483000
1	-4.450141000	1.655026000	-2.218520000
6	-3.924457000	-0.521495000	-2.122149000
1	-2.892610000	-0.532725000	-2.460718000
6	-4.672519000	-1.647364000	-1.667082000
1	-4.312575000	-2.669963000	-1.607545000
6	-5.956031000	-1.183218000	-1.254865000
1	-6.744248000	-1.789278000	-0.820992000
6	-5.998987000	0.228272000	-1.451067000
1	-6.826672000	0.881496000	-1.196289000
6	-2.649497000	2.204210000	0.821896000
1	-1.923035000	2.378822000	1.627454000
1	-3.348974000	3.048191000	0.795130000
6	-0.421456000	-0.843825000	2.399031000
6	0.862113000	-1.369986000	2.584349000
1	1.371143000	-1.868473000	1.757785000
6	1.495129000	-1.274058000	3.818392000
1	2.491595000	-1.695232000	3.939308000
6	0.864534000	-0.624870000	4.878581000
1	1.365146000	-0.539554000	5.840807000
6	-0.405522000	-0.087005000	4.700625000
1	-0.905646000	0.418836000	5.523999000
6	-1.049698000	-0.200798000	3.469440000
1	-2.048839000	0.217211000	3.350982000
6	-1.317595000	-2.677897000	0.352084000
6	-1.359342000	-3.640267000	1.366428000
1	-1.281133000	-3.337973000	2.410057000
6	-1.503047000	-4.988831000	1.052075000
1	-1.526901000	-5.728615000	1.849404000
6	-1.617627000	-5.389950000	-0.276676000
1	-1.729005000	-6.444367000	-0.519822000
6	-1.589172000	-4.438168000	-1.293429000
1	-1.678725000	-4.744437000	-2.333743000
6	-1.431510000	-3.089437000	-0.981045000
1	-1.392150000	-2.346857000	-1.778308000
6	-0.961356000	3.865916000	-0.600226000
6	-1.245940000	4.816471000	-1.579716000
1	-1.917590000	4.567718000	-2.399510000
6	-0.669884000	6.082238000	-1.500420000
1	-0.888466000	6.821829000	-2.267306000
6	0.171425000	6.399846000	-0.439043000
1	0.614487000	7.391000000	-0.373469000
6	0.447435000	5.448173000	0.541048000
1	1.106420000	5.694760000	1.370952000
6	-0.107751000	4.175460000	0.459324000
1	0.131698000	3.425270000	1.213098000
1	0.820174000	1.415185000	-1.913309000
8	5.290004000	-2.726065000	-2.326974000
6	5.743963000	-1.706500000	-3.202476000
1	6.726717000	-1.942988000	-3.631695000
1	5.036207000	-1.517850000	-4.022001000
1	5.846135000	-0.788614000	-2.610499000
1	5.200769000	-3.543544000	-2.831802000
8	1.899990000	-3.587361000	-1.281514000
6	1.689280000	-3.416737000	-2.673290000
1	1.199883000	-4.289140000	-3.127710000
1	1.090748000	-2.520459000	-2.898640000
1	2.673736000	-3.302220000	-3.143551000
1	1.035067000	-3.668123000	-0.854075000

7-K

E (BS1) = -3462.099059

E (BS2) = -4602.684397

$G_{298.15,1M}$ (BS2) = -4601.9204

1	2.113649000	1.738333000	2.816284000
1	1.282318000	-0.396592000	-0.611393000
1	-0.457868000	-0.064553000	-2.085216000
19	3.790957000	-1.261906000	-0.954887000
1	3.667471000	-0.965154000	1.861250000
8	4.021788000	-1.865281000	1.727793000
6	5.292809000	-1.952454000	2.341768000
1	5.678520000	-2.963273000	2.174703000
1	6.010988000	-1.237083000	1.914668000
1	5.241304000	-1.787872000	3.428358000
1	1.933921000	0.611189000	-2.409262000
8	2.796019000	0.508180000	-2.869593000
6	3.549584000	1.670245000	-2.582662000
1	4.523952000	1.574392000	-3.076416000
1	3.064978000	2.582162000	-2.960312000
1	3.723930000	1.800086000	-1.500584000
8	3.073248000	0.756338000	1.274027000
6	3.091360000	1.759163000	2.303088000
6	3.315151000	3.122509000	1.682814000
6	4.181873000	1.381947000	3.277566000
1	2.548607000	3.343030000	0.928672000
1	4.298462000	3.158669000	1.190487000
1	3.285646000	3.911193000	2.444558000
1	4.268256000	2.132373000	4.071366000
1	3.976720000	0.411500000	3.747916000
1	5.150065000	1.321811000	2.759919000
8	5.550500000	0.598177000	-0.047949000
6	6.435257000	1.677449000	-0.265395000
1	4.769541000	0.919671000	0.441611000
1	7.298169000	1.299265000	-0.823370000
1	5.974635000	2.483682000	-0.856174000
1	6.806000000	2.106620000	0.676891000
1	2.203788000	0.829913000	0.806928000
1	0.563956000	1.394076000	0.804778000
26	-4.459704000	-0.539164000	-0.179996000
77	0.022807000	0.631321000	-0.610026000
15	-1.094647000	-0.922443000	0.709857000
16	-1.889436000	2.160977000	-0.761673000
6	-2.846265000	-0.576641000	1.069821000
6	-3.461378000	0.726297000	1.072375000
6	-4.816095000	0.562776000	1.497465000
1	-5.545619000	1.363935000	1.569077000

6	-5.046829000	-0.817480000	1.755960000
1	-5.992364000	-1.262178000	2.047735000
6	-3.840044000	-1.520724000	1.491119000
1	-3.700563000	-2.595408000	1.556059000
6	-4.749610000	0.385950000	-1.983168000
1	-4.517121000	1.425121000	-2.188592000
6	-3.856481000	-0.715088000	-2.128434000
1	-2.822865000	-0.655140000	-2.457061000
6	-4.538127000	-1.894631000	-1.704709000
1	-4.118622000	-2.894738000	-1.660700000
6	-5.852990000	-1.520523000	-1.300190000
1	-6.606287000	-2.184090000	-0.888965000
6	-5.982799000	-0.111225000	-1.470467000
1	-6.852601000	0.484024000	-1.213777000
6	-2.836394000	2.055346000	0.820315000
1	-2.135936000	2.314209000	1.626622000
1	-3.613253000	2.828400000	0.771157000
6	-0.386907000	-1.031543000	2.406913000
6	0.896864000	-1.565636000	2.568732000
1	1.416559000	-2.001043000	1.713500000
6	1.521770000	-1.546343000	3.810512000
1	2.519522000	-1.970652000	3.915144000
6	0.876918000	-0.977063000	4.908208000
1	1.368278000	-0.954117000	5.878603000
6	-0.397199000	-0.440251000	4.756187000
1	-0.908753000	0.002266000	5.608660000
6	-1.029168000	-0.470948000	3.513427000
1	-2.028561000	-0.047902000	3.412435000
6	-1.166149000	-2.691164000	0.191604000
6	-1.231325000	-3.737278000	1.119287000
1	-1.211139000	-3.522615000	2.187101000
6	-1.324190000	-5.058337000	0.689761000
1	-1.367690000	-5.861209000	1.422807000
6	-1.361547000	-5.351711000	-0.671327000
1	-1.432899000	-6.384802000	-1.004655000
6	-1.304204000	-4.318659000	-1.603375000
1	-1.331347000	-4.539324000	-2.668775000
6	-1.199799000	-2.996905000	-1.173169000
1	-1.134687000	-2.188264000	-1.901844000
6	-1.363540000	3.876547000	-0.600567000
6	-1.886199000	4.800902000	-1.503578000
1	-2.589810000	4.475433000	-2.268034000
6	-1.503161000	6.138204000	-1.421706000
1	-1.906810000	6.858577000	-2.129721000
6	-0.612723000	6.549405000	-0.435278000
1	-0.315695000	7.593932000	-0.371677000
6	-0.100399000	5.622988000	0.471867000
1	0.597743000	5.941242000	1.243778000
6	-0.469221000	4.284848000	0.389398000
1	-0.054924000	3.552372000	1.081164000
1	0.901733000	1.763065000	-1.472615000
8	5.426798000	-2.350808000	-2.780629000
6	5.699441000	-1.251847000	-3.636181000
1	6.538130000	-1.459146000	-4.313884000
1	4.823241000	-0.966371000	-4.236184000
1	5.984369000	-0.402322000	-3.003058000
1	5.206272000	-3.114428000	-3.328591000
8	2.107909000	-3.321019000	-1.490520000
6	1.845362000	-3.001139000	-2.848272000
1	1.313023000	-3.809794000	-3.367811000
1	1.265351000	-2.070386000	-2.952286000
1	2.814015000	-2.865866000	-3.346517000
1	1.258954000	-3.419055000	-1.034373000

C. 3MeOH model

C1. Lithium system

1-Li-3MeOH

E (BS1) = -2444.1636623
E (BS2) = -3584.5451697
$G_{298.15,1M}$ (BS2) = -3583.9740709

Li	-3.125906	1.686660	-0.138202
H	-2.861620	2.743373	2.111043
O	-3.443256	2.029425	1.804919
C	-3.353688	0.956732	2.736861
H	-4.033160	0.167822	2.398787
H	-2.335900	0.546411	2.789545
H	-3.661781	1.279636	3.739100
H	-4.628844	0.838722	-1.880843
H	-2.082648	3.681196	-1.148403
O	-3.015456	3.505591	-0.960634
O	-4.787846	0.938710	-0.931383
C	-3.748397	3.711022	-2.166216
H	-4.809185	3.588000	-1.933024
H	-3.592743	4.724630	-2.552817
H	-3.463737	2.985267	-2.940271
C	-5.185339	-0.332945	-0.419956
H	-5.425366	-0.199877	0.639671
H	-6.080401	-0.701355	-0.935049
H	-4.378247	-1.071385	-0.518536
Fe	3.133406	-1.602850	0.660966
Ir	-0.867999	0.001085	-1.131983
P	0.900867	0.975915	0.049219
S	-0.460819	-2.254000	-0.228381
C	1.838953	-0.117533	1.171991
C	1.378263	-1.386222	1.680759
C	2.394854	-1.904227	2.540466
H	2.347914	-2.861989	3.049799
C	3.473291	-0.977775	2.576089
H	4.411604	-1.109906	3.104320
C	3.137168	0.118593	1.736497
H	3.778214	0.966044	1.518740
C	2.978498	-3.149697	-0.671383
H	2.146356	-3.843618	-0.719613
C	3.071964	-1.905752	-1.361110
H	2.318602	-1.486563	-2.021688
C	4.297869	-1.281559	-0.982007
H	4.642454	-0.303022	-1.303750
C	4.961795	-2.143551	-0.060324
H	5.899023	-1.936130	0.445198
C	4.145718	-3.296418	0.133089
H	4.356509	-4.120272	0.806545
C	0.042698	-2.030919	1.521105
H	-0.721079	-1.410006	2.007204
H	0.047866	-3.022073	1.991123
C	0.318729	2.286130	1.208030
C	-0.049572	3.537813	0.699681
H	0.133897	3.772126	-0.350591
C	-0.646363	4.489699	1.519634
H	-0.925025	5.458586	1.109843
C	-0.886648	4.202043	2.863929
H	-1.353762	4.944891	3.506801
C	-0.514078	2.963261	3.380493
H	-0.692357	2.733440	4.429332
C	0.088573	2.012100	2.558037

H	0.368133	1.044154	2.975267
C	2.249135	1.847812	-0.858560
C	3.091997	2.768304	-0.221725
H	2.925393	3.031498	0.822466
C	4.143448	3.359711	-0.914065
H	4.791461	4.071563	-0.406889
C	4.361202	3.048052	-2.255092
H	5.181980	3.514426	-2.795920
C	3.517839	2.150325	-2.902174
H	3.674137	1.912055	-3.952286
C	2.466777	1.556128	-2.206909
H	1.801110	0.855446	-2.710643
C	-2.077676	-3.013579	-0.019805
C	-2.558522	-3.730118	-1.118710
H	-1.940971	-3.836771	-2.010355
C	-3.827302	-4.296360	-1.077684
H	-4.195526	-4.849239	-1.939178
C	-4.617983	-4.165771	0.062677
H	-5.608409	-4.613774	0.095883
C	-4.128959	-3.466312	1.161159
H	-4.735769	-3.361450	2.058552
C	-2.862701	-2.884503	1.125438
H	-2.512100	-2.330583	1.992530
H	-1.188160	1.440603	-1.801270
H	-2.174187	-0.561041	-1.997967
H	-1.898832	0.269343	0.201626
H	0.043587	-0.331056	-2.510774

2- Li-3MeOH

E (BS1) = -2637.2162287

E (BS2) = -3777.672034

$G_{298.15,1M}$ (BS2) = -3777.0235839

Li	-3.438811	1.393132	-0.393255
H	-2.630728	3.521658	0.714580
O	-3.458446	3.025505	0.811601
C	-4.064735	3.376463	2.043604
H	-4.998831	2.811720	2.126071
H	-3.425399	3.130977	2.902950
H	-4.303288	4.447363	2.079961
H	-4.009170	0.693987	-2.688570
H	-1.662453	2.069443	-1.756735
O	-2.436576	2.675522	-1.774167
O	-4.537295	0.551565	-1.891432
C	-2.666157	3.033623	-3.123612
H	-3.647642	3.514240	-3.191428
H	-1.909883	3.742848	-3.488983
H	-2.659832	2.158846	-3.792073
C	-4.833379	-0.841588	-1.818135
H	-5.523585	-0.988943	-0.983749
H	-5.316222	-1.190292	-2.739177
H	-3.923441	-1.431675	-1.642000
O	-4.316054	0.204053	1.005385
C	-4.128654	-0.071310	2.187713
C	-5.166627	-0.786749	2.985814
C	-2.869277	0.303399	2.895596
H	-4.717941	-1.598510	3.570405
H	-5.963802	-1.169290	2.343157
H	-5.595339	-0.080379	3.709831
H	-2.346846	-0.610391	3.212111
H	-2.218749	0.900649	2.249178
H	-3.103391	0.854275	3.815987
Fe	3.270574	-1.629718	1.075602
Ir	-0.273843	-0.060845	-1.479977
P	1.281486	0.962972	-0.068563
S	-0.165552	-2.229155	-0.324833
C	1.982918	-0.065394	1.265417
C	1.386444	-1.272217	1.782658
C	2.228931	-1.758955	2.828392
H	2.056418	-2.672075	3.390049
C	3.331753	-0.872092	2.971524
H	4.167406	-0.996867	3.651928
C	3.185373	0.167615	2.013502
H	3.892014	0.972708	1.842113
C	3.268240	-3.278087	-0.139540
H	2.430558	-3.956172	-0.260651
C	3.504362	-2.097404	-0.902078
H	2.869900	-1.716992	-1.697730
C	4.678769	-1.468884	-0.390092
H	5.099094	-0.526701	-0.730077
C	5.167940	-2.264754	0.686996
H	6.024650	-2.034631	1.311525
C	4.295172	-3.381398	0.843154
H	4.374746	-4.150930	1.603617
C	0.060517	-1.875360	1.458870
H	-0.739785	-1.186433	1.760529
H	-0.057083	-2.821737	2.001210
C	0.554822	2.363656	0.886020
C	0.238220	3.550728	0.214849
H	0.496404	3.666370	-0.838820
C	-0.402016	4.591761	0.881225
H	-0.631564	5.513216	0.349569
C	-0.747324	4.454287	2.226789
H	-1.252620	5.265871	2.746788
C	-0.442674	3.273723	2.898810
H	-0.709151	3.157314	3.948077
C	0.209683	2.236635	2.233956
H	0.441185	1.317673	2.774152
C	2.786043	1.751556	-0.785977
C	3.529949	2.698047	-0.069443
H	3.194735	3.026503	0.914138
C	4.698090	3.230832	-0.604446
H	5.267719	3.962911	-0.035673
C	5.132021	2.835051	-1.868445
H	6.043573	3.255944	-2.287641
C	4.388927	1.911740	-2.597234
H	4.714549	1.608742	-3.590298
C	3.221557	1.374619	-2.058412
H	2.636331	0.653090	-2.628051
C	-1.801020	-2.972685	-0.329557
C	-2.160375	-3.632980	-1.508753
H	-1.459522	-3.676192	-2.342099
C	-3.410684	-4.228140	-1.619336
H	-3.681364	-4.738115	-2.541341
C	-4.307568	-4.181477	-0.552889
H	-5.283706	-4.653403	-0.637570
C	-3.941685	-3.535838	0.622884
H	-4.631278	-3.504305	1.464711
C	-2.692253	-2.926777	0.741616
H	-2.429724	-2.431848	1.673934
H	-0.267944	1.294141	-2.371947
H	-1.439118	-0.636486	-2.519651
H	-1.540822	0.445384	-0.420156
H	0.860019	-0.616239	-2.593787

$TS_{2\text{-}3}$- Li-3MeOH

E (BS1) = -2637.2059563

E (BS2) = -3777.6594488

$G_{298.15,1M}$ (BS2) = -3777.0097489

Li	4.701082	-0.798328	-0.388875
H	7.002205	-1.497856	-1.150439
O	6.510008	-1.524770	-0.319353
C	6.533013	-2.857530	0.182300
H	5.934997	-2.868692	1.099213
H	6.102081	-3.569103	-0.533806
H	7.554075	-3.170420	0.429986
H	3.960052	1.093567	-1.713769
H	2.710018	-1.718337	-1.273365
O	3.622893	-2.060027	-1.410026
O	4.865291	0.872234	-1.437921
C	3.868707	-2.091060	-2.807671
H	4.929820	-2.319751	-2.957111
H	3.272335	-2.869356	-3.301687
H	3.647473	-1.124952	-3.282901
C	5.351869	1.913546	-0.596123
H	6.255293	1.540662	-0.101656
H	5.619624	2.803597	-1.179129
H	4.616761	2.189754	0.171460
O	3.895190	-0.408340	1.264074
C	2.814413	-0.853111	1.753628
C	2.197305	-0.087310	2.895593
C	2.593430	-2.343782	1.793935
H	1.159292	-0.380963	3.088269
H	2.260830	0.991283	2.714996
H	2.787881	-0.312896	3.797049
H	1.563569	-2.598600	2.061371
H	2.859708	-2.815958	0.842953
H	3.257527	-2.746642	2.574562
Fe	-3.622324	1.466119	0.501340
Ir	0.684484	-0.142096	-0.830150
P	-1.290461	-1.087763	0.003760
S	0.022725	2.112938	-0.100728
C	-2.332885	-0.013806	1.050821
C	-1.951907	1.243301	1.647607
C	-3.049322	1.695214	2.443506
H	-3.074416	2.634159	2.988245
C	-4.100328	0.741506	2.352343
H	-5.080881	0.826744	2.808326
C	-3.667750	-0.304047	1.494226
H	-4.258915	-1.163688	1.196154
C	-3.391414	3.101546	-0.704587
H	-2.575154	3.812038	-0.628527
C	-3.392367	1.907404	-1.482396
H	-2.578183	1.553555	-2.106909
C	-4.630744	1.234716	-1.259175
H	-4.922412	0.277396	-1.680742
C	-5.394808	2.016728	-0.344509
H	-6.367447	1.756109	0.059134
C	-4.628727	3.169027	-0.000881
H	-4.918000	3.937988	0.707486
C	-0.656078	1.982070	1.594402
H	0.095177	1.486363	2.222892
H	-0.811054	3.003728	1.964316
C	-1.006461	-2.528444	1.113804
C	-0.540748	-3.723942	0.552254
H	-0.407059	-3.801008	-0.527522
C	-0.252988	-4.818460	1.359134
H	0.099530	-5.744075	0.908733
C	-0.414410	-4.729835	2.741306
H	-0.188546	-5.586372	3.372845
C	-0.864632	-3.542356	3.309025
H	-0.994031	-3.466375	4.386735
C	-1.162437	-2.446423	2.499568
H	-1.521637	-1.524747	2.957251
C	-2.523766	-1.787509	-1.179598
C	-3.475131	-2.731495	-0.771850
H	-3.461587	-3.118801	0.246276
C	-4.441347	-3.192032	-1.660514
H	-5.173849	-3.924676	-1.328163
C	-4.465643	-2.723461	-2.972717
H	-5.218945	-3.087762	-3.668075
C	-3.514371	-1.799100	-3.393100
H	-3.518228	-1.437737	-4.419427
C	-2.548561	-1.337013	-2.501647
H	-1.798976	-0.619061	-2.833140
C	1.452094	3.166725	0.165003
C	2.048844	3.675936	-0.993716
H	1.659920	3.397782	-1.973105
C	3.127446	4.544685	-0.894060
H	3.582251	4.940052	-1.799951
C	3.620957	4.912455	0.357238
H	4.463597	5.595790	0.433724
C	3.028026	4.401943	1.505613
H	3.403442	4.683734	2.487055
C	1.942156	3.529609	1.418372
H	1.489047	3.156107	2.332813
H	1.030994	-1.598068	-1.457157
H	2.106233	0.451066	-1.468817
H	1.606003	-0.522148	0.664459
H	0.029092	0.196340	-2.299342

3- Li-3MeOH

E (BS1) = -2637.2194309

E (BS2) = -3777.6745058

$G_{298.15,1M}$ (BS2) = -3777.0203160

Li	4.684723	-0.764377	-0.420114
H	6.907423	-1.419060	-1.455764
O	6.519976	-1.512381	-0.575607
C	6.582450	-2.882551	-0.195313
H	6.091787	-2.971561	0.779263
H	6.060656	-3.529181	-0.913026
H	7.620225	-3.222904	-0.096161
H	3.928316	1.173265	-1.711929
H	2.670240	-1.678123	-1.341198
O	3.586434	-1.984500	-1.514814
O	4.836763	0.932387	-1.468480
C	3.791695	-1.935465	-2.917968
H	4.857523	-2.100499	-3.110284
H	3.221562	-2.719360	-3.434236
H	3.507016	-0.960801	-3.338650
C	5.350086	1.932330	-0.594056
H	6.270429	1.538023	-0.149635
H	5.596965	2.851595	-1.140261
H	4.642557	2.165997	0.213119
O	4.061855	-0.538325	1.270206
C	2.813934	-0.951948	1.619356
C	2.233859	-0.137576	2.773142
C	2.763663	-2.443336	1.949527
H	1.197055	-0.423820	3.008351
H	2.258681	0.933828	2.530146
H	2.843374	-0.290151	3.676663
H	1.775243	-2.767887	2.302082
H	3.034941	-3.050679	1.074425
H	3.494521	-2.654249	2.745009

Fe	-3.650311	1.463851	0.370108
Ir	0.643564	-0.119219	-0.821831
P	-1.313089	-1.087669	0.080836
S	-0.005288	2.144205	-0.035274
C	-2.403871	0.005493	1.048371
C	-2.052427	1.281276	1.624714
C	-3.193746	1.763150	2.336064
H	-3.246458	2.718858	2.848524
C	-4.241349	0.809343	2.214566
H	-5.247343	0.912488	2.606914
C	-3.763034	-0.268171	1.422581
H	-4.340839	-1.133935	1.116416
C	-3.347289	3.038674	-0.903209
H	-2.533731	3.751722	-0.824281
C	-3.314919	1.806665	-1.618719
H	-2.470605	1.417080	-2.179173
C	-4.567224	1.150306	-1.426358
H	-4.841542	0.172749	-1.812105
C	-5.372570	1.979896	-0.592648
H	-6.366318	1.743161	-0.227699
C	-4.618367	3.145455	-0.268461
H	-4.939155	3.951044	0.383278
C	-0.743537	1.998804	1.635782
H	-0.024006	1.481777	2.285330
H	-0.893436	3.017875	2.013516
C	-0.978041	-2.451369	1.265436
C	-0.475061	-3.661468	0.770914
H	-0.331991	-3.793097	-0.302335
C	-0.168859	-4.703471	1.638168
H	0.209920	-5.643103	1.241547
C	-0.342758	-4.542955	3.012155
H	-0.101528	-5.358067	3.690888
C	-0.823495	-3.337214	3.512788
H	-0.960828	-3.205958	4.584071
C	-1.144501	-2.295305	2.643778
H	-1.531925	-1.359952	3.047846
C	-2.481239	-1.881757	-1.099962
C	-3.420729	-2.826999	-0.669371
H	-3.433448	-3.151429	0.370599
C	-4.339811	-3.366819	-1.563119
H	-5.065071	-4.098786	-1.214222
C	-4.325899	-2.977967	-2.901224
H	-5.042772	-3.403746	-3.600042
C	-3.384269	-2.053713	-3.343076
H	-3.359127	-1.754894	-4.388868
C	-2.466059	-1.510831	-2.447304
H	-1.729125	-0.790349	-2.798804
C	1.425519	3.184875	0.276395
C	2.049520	3.707717	-0.861253
H	1.678172	3.451574	-1.853361
C	3.133303	4.565005	-0.723212
H	3.610231	4.972706	-1.612086
C	3.602951	4.906193	0.544577
H	4.450181	5.579694	0.650777
C	2.980473	4.383006	1.671563
H	3.337064	4.644983	2.665339
C	1.889108	3.522612	1.546504
H	1.412940	3.136332	2.443814
H	0.954501	-1.542304	-1.530081
H	2.004322	0.506425	-1.549381
H	2.077243	-0.817682	0.739815
H	-0.098682	0.252229	-2.141014

4- Li-3MeOH

E (BS1) = -2637.2317815
E (BS2) = -3777.6850502
$G_{298.15,1M}$ (BS2) = -3777.0273784

H	0.755663	-1.050083	2.184355
Li	3.786570	-0.868872	-0.104057
H	4.160921	1.383744	-1.055916
H	2.580372	-2.094821	-1.560877
O	3.542215	-2.281107	-1.505026
O	4.814231	0.703106	-0.827476
C	4.109794	-2.048599	-2.785252
H	5.198124	-2.003894	-2.672090
H	3.867235	-2.860113	-3.483697
H	3.762836	-1.098797	-3.216524
C	5.734204	1.260853	0.104883
H	6.574266	0.565992	0.200483
H	6.124127	2.222927	-0.247603
H	5.274876	1.403751	1.094016
O	2.155565	-0.548340	0.751712
C	1.830277	-1.209255	1.935223
C	2.646845	-0.641343	3.091523
C	2.050717	-2.715484	1.835397
H	2.390418	-1.116643	4.048381
H	2.484653	0.440754	3.194856
H	3.720258	-0.803540	2.910829
H	1.754100	-3.233946	2.758130
H	1.469947	-3.142920	1.007052
H	3.114138	-2.931287	1.654980
O	5.303809	-1.733381	0.941485
C	6.315011	-2.332938	0.141003
H	5.727597	-1.162573	1.595220
H	5.829535	-3.077281	-0.497328
H	6.822766	-1.595615	-0.497514
H	7.064116	-2.839998	0.760383
Fe	-3.259831	1.772448	0.817720
Ir	0.702558	-0.287676	-1.027710
P	-1.349803	-1.027436	-0.110019
S	0.318408	2.004808	-0.192751
C	-2.135181	0.086539	1.107517
C	-1.523043	1.230131	1.738480
C	-2.455930	1.752233	2.687199
H	-2.292995	2.637832	3.294035
C	-3.634539	0.956222	2.650990
H	-4.539974	1.130136	3.222519
C	-3.445914	-0.058406	1.674678
H	-4.177149	-0.807192	1.387998
C	-2.943908	3.489308	-0.249248
H	-2.039774	4.086647	-0.198046
C	-3.175957	2.394494	-1.131183
H	-2.481358	2.018356	-1.875264
C	-4.461284	1.850840	-0.834058
H	-4.914931	0.986987	-1.310341
C	-5.023104	2.613568	0.230643
H	-5.975161	2.426886	0.716235
C	-4.085571	3.624735	0.592541
H	-4.200825	4.340405	1.399695
C	-0.158310	1.809343	1.564831
H	0.604826	1.176672	2.038119
H	-0.138712	2.804360	2.026155
C	-1.307334	-2.632908	0.794930
C	-1.020217	-3.787907	0.055658
H	-0.864388	-3.717809	-1.021883
C	-0.938306	-5.026836	0.679894
H	-0.721869	-5.915745	0.090772

C	-1.130288	-5.128264	2.057098
H	-1.063735	-6.096857	2.547901
C	-1.408820	-3.986258	2.800493
H	-1.562602	-4.057998	3.875385
C	-1.500698	-2.744163	2.173172
H	-1.725538	-1.860382	2.769051
C	-2.747684	-1.352890	-1.280306
C	-3.809893	-2.193564	-0.921288
H	-3.792302	-2.720396	0.031997
C	-4.892566	-2.375770	-1.776034
H	-5.707091	-3.033786	-1.480358
C	-4.929363	-1.726198	-3.008066
H	-5.773526	-1.873748	-3.678212
C	-3.875310	-0.897819	-3.380551
H	-3.890006	-0.392679	-4.344143
C	-2.793076	-0.715052	-2.522852
H	-1.970628	-0.069113	-2.824397
C	1.882346	2.870792	-0.039240
C	2.465891	3.287349	-1.242012
H	1.990353	3.042783	-2.191390
C	3.645495	4.022482	-1.224783
H	4.088627	4.344984	-2.164487
C	4.248106	4.356752	-0.011792
H	5.166385	4.939285	0.000627
C	3.668021	3.937632	1.179343
H	4.132778	4.187006	2.131171
C	2.489291	3.191142	1.174611
H	2.058825	2.879912	2.122454
H	0.899133	-1.769205	-1.658502
H	2.064659	0.186928	-1.850916
H	-0.062313	0.041861	-2.380111

5- Li-3MeOH

E (BS1) = -2638.4056006

E (BS2) = -3778.8620721

$G_{298.15,1M}$ (BS2) = -3778.1948582

H	1.079202	-0.097085	2.028690
H	1.243888	-1.126630	-1.916086
H	-0.302353	0.391558	-2.397274
Li	3.871890	-0.910948	-0.313745
H	2.975120	-3.198527	-0.803432
O	3.870167	-2.886240	-0.610983
C	4.461867	-3.771288	0.331076
H	5.468926	-3.400453	0.544287
H	3.893148	-3.807088	1.271122
H	4.543648	-4.787134	-0.074989
H	3.113886	0.158792	-2.144195
O	4.077682	-0.041993	-2.107786
C	4.760491	1.201484	-2.042080
H	5.804516	1.036796	-2.326755
H	4.329294	1.932425	-2.739253
H	4.737234	1.629714	-1.026613
O	2.372682	-0.073996	0.421171
C	2.166684	-0.030464	1.797955
C	2.693465	1.266632	2.405181
C	2.837137	-1.216334	2.486246
H	2.193064	2.148634	1.985240
H	3.768621	1.366622	2.185567
H	2.567895	1.292450	3.497024
H	2.614981	-1.246944	3.562928
H	2.500656	-2.161826	2.040552
H	3.930881	-1.154940	2.373994
O	5.676827	-0.708678	0.489953
C	6.863394	-0.950789	-0.252400
H	5.862767	-0.062005	1.181821
H	6.596800	-1.580402	-1.107457
H	7.305601	-0.018951	-0.626792
H	7.612477	-1.479985	0.350511
H	0.117721	0.045394	4.000837
H	-0.217543	0.303953	4.615866
Fe	-3.688314	0.858446	0.687587
Ir	0.686734	0.197184	-1.174500
P	-0.918408	-1.135798	-0.089130
S	-0.317066	2.274532	-0.289416
C	-2.055092	-0.302633	1.073923
C	-1.901633	1.012736	1.649072
C	-2.981818	1.212538	2.564187
H	-3.157426	2.125590	3.125254
C	-3.801385	0.050530	2.563273
H	-4.723656	-0.081477	3.118946
C	-3.242859	-0.875840	1.643295
H	-3.657620	-1.846000	1.390477
C	-3.936263	2.508588	-0.493395
H	-3.286283	3.377628	-0.478470
C	-3.759957	1.345006	-1.297346
H	-2.954750	1.176498	-2.005519
C	-4.796760	0.420145	-0.971352
H	-4.917431	-0.575902	-1.387083
C	-5.614448	1.015579	0.032979
H	-6.463518	0.550607	0.522660
C	-5.082029	2.304671	0.329226
H	-5.456414	2.990424	1.081835
C	-0.829656	2.032846	1.466808
H	0.069354	1.771978	2.039639
H	-1.196297	3.001955	1.826958
C	-0.241048	-2.502292	0.946802
C	0.498135	-3.501007	0.299602
H	0.624480	-3.462748	-0.784258
C	1.048077	-4.555873	1.019685
H	1.604615	-5.334192	0.500049
C	0.880918	-4.618513	2.403285
H	1.313397	-5.441555	2.968204
C	0.154354	-3.627897	3.054923
H	0.016313	-3.670980	4.133538
C	-0.408404	-2.576990	2.330787
H	-0.984449	-1.815771	2.855343
C	-2.091280	-2.083966	-1.159558
C	-2.750945	-3.222930	-0.678960
H	-2.535799	-3.599219	0.320559
C	-3.679838	-3.892074	-1.469473
H	-4.183149	-4.774326	-1.079351
C	-3.956590	-3.440446	-2.758152
H	-4.679158	-3.967230	-3.377808
C	-3.294648	-2.321028	-3.252721
H	-3.494715	-1.968570	-4.262498
C	-2.367713	-1.649315	-2.458719
H	-1.849164	-0.777192	-2.853124
C	0.840728	3.632278	-0.044603
C	0.341851	4.865110	0.382539
H	-0.722812	4.986620	0.581608
C	1.208890	5.940415	0.544355
H	0.820427	6.898630	0.882382
C	2.565441	5.795700	0.259744
H	3.239252	6.641014	0.380276
C	3.055278	4.570701	-0.183366
H	4.113219	4.451912	-0.411266
C	2.196113	3.484947	-0.332410

H	2.566641	2.517341	-0.664798
H	1.744086	1.081009	-2.112326

$TS_{5\text{-}6}$- Li-3MeOH

E (BS1) = -2638.3942503
E (BS2) = -3778.8501325
$G_{298.15,1M}$ (BS2) = -3778.1807046

H	3.002681	1.143481	2.175316
H	1.104761	-1.072700	-1.683487
H	-0.540264	0.295870	-2.133700
Li	3.981371	-0.953120	-0.741286
H	2.336200	-2.559158	-1.306167
O	3.288407	-2.746094	-1.352337
C	3.614628	-3.743270	-0.391707
H	4.646446	-4.057295	-0.578912
H	3.546488	-3.353239	0.635049
H	2.966329	-4.622830	-0.489403
H	2.820205	0.286862	-2.344598
O	3.789550	0.134910	-2.413776
C	4.416527	1.405005	-2.330022
H	5.499620	1.248649	-2.281211
H	4.198601	2.021153	-3.212731
H	4.101293	1.958639	-1.432289
O	2.985050	-0.291012	0.682851
C	3.549113	0.230826	1.838698
C	4.998792	0.665340	1.623218
C	3.485482	-0.760180	2.997193
H	5.080858	1.319743	0.742164
H	5.639952	-0.214154	1.459975
H	5.399756	1.210346	2.489661
H	3.914660	-0.344190	3.920221
H	2.449479	-1.057007	3.210224
H	4.050428	-1.671174	2.742063
O	5.941951	-1.379544	-0.822310
C	6.414932	-1.917867	-2.052885
H	6.522051	-0.650381	-0.566372
H	5.730140	-2.718439	-2.347092
H	6.431768	-1.161248	-2.847853
H	7.420351	-2.340688	-1.939641
H	0.833526	0.655638	2.694318
H	0.872839	1.147021	3.255934
Fe	-3.922964	0.819250	0.145937
Ir	0.513479	0.270800	-0.994570
P	-0.980933	-1.026693	0.281757
S	-0.473087	2.418003	-0.363988
C	-2.352057	-0.103726	1.055948
C	-2.384783	1.301340	1.391037
C	-3.641656	1.565498	2.020190
H	-3.972202	2.544894	2.352817
C	-4.384078	0.355401	2.083283
H	-5.394114	0.244383	2.462918
C	-3.600317	-0.668392	1.488619
H	-3.909911	-1.698282	1.346825
C	-3.965358	2.152975	-1.405793
H	-3.341669	3.037673	-1.479707
C	-3.630740	0.851137	-1.879140
H	-2.702984	0.574755	-2.371206
C	-4.697839	-0.032535	-1.538508
H	-4.726441	-1.101829	-1.726268
C	-5.694361	0.727294	-0.858264
H	-6.614074	0.337945	-0.434773
C	-5.240969	2.076578	-0.774484
H	-5.756835	2.891991	-0.278823
C	-1.342722	2.358279	1.264887
H	-0.564221	2.243272	2.028484
H	-1.809190	3.342512	1.399215
C	-0.291181	-1.972105	1.700364
C	0.624928	-2.994004	1.422407
H	0.879446	-3.222605	0.385940
C	1.191388	-3.736154	2.451892
H	1.891864	-4.537001	2.220991
C	0.863033	-3.453313	3.777002
H	1.309073	-4.030258	4.584253
C	-0.036675	-2.431497	4.063389
H	-0.298404	-2.207447	5.095484
C	-0.616826	-1.697043	3.030128
H	-1.326681	-0.905642	3.268536
C	-1.872488	-2.359878	-0.630053
C	-2.488849	-3.416829	0.051899
H	-2.401873	-3.492476	1.135406
C	-3.211999	-4.381333	-0.642149
H	-3.687631	-5.194368	-0.097731
C	-3.319127	-4.311259	-2.029556
H	-3.880852	-5.069039	-2.571598
C	-2.693515	-3.277339	-2.719321
H	-2.760550	-3.223508	-3.803888
C	-1.973527	-2.308856	-2.023387
H	-1.483266	-1.504328	-2.568982
C	0.818222	3.572663	0.120783
C	0.521673	4.935263	0.159123
H	-0.474568	5.285157	-0.108457
C	1.508603	5.841234	0.536172
H	1.281556	6.904626	0.565212
C	2.783068	5.387756	0.868903
H	3.553026	6.098533	1.160772
C	3.073302	4.026457	0.825976
H	4.069005	3.669635	1.086555
C	2.092056	3.111821	0.450643
H	2.311069	2.042305	0.405863
H	1.440988	1.182022	-2.044787

6- Li-3MeOH

E (BS1) = -2638.4017945
E (BS2) = -3778.8594241
$G_{298.15,1M}$ (BS2) = -3778.1865462

H	2.656267	-0.739947	1.861095
H	1.016992	-1.078495	-1.857407
H	-0.629750	0.263615	-2.384155
Li	4.407042	-0.519241	-0.451491
H	3.009695	-2.398388	0.486403
O	3.522632	-2.298871	-0.330098
C	4.228876	-3.504180	-0.578932
H	4.749805	-3.387664	-1.534333
H	4.968175	-3.718104	0.205671
H	3.545391	-4.358752	-0.667150
H	2.827668	0.111046	-2.173056
O	3.803941	0.068657	-2.259552
C	4.262961	1.388504	-2.516189
H	5.342814	1.342686	-2.692734
H	3.787555	1.811983	-3.410525
H	4.074484	2.060488	-1.664397
O	4.005928	0.561718	0.976168
C	3.517802	-0.058489	2.106538
C	2.962973	0.945772	3.116209
C	4.567419	-0.946115	2.777962
H	2.143215	1.530398	2.673758

H	3.750895	1.654271	3.414943
H	2.578946	0.458715	4.024416
H	4.175699	-1.462385	3.666729
H	4.932618	-1.711287	2.076936
H	5.431022	-0.340590	3.092644
O	6.327634	-0.799440	-0.789440
C	6.909743	-1.443031	-1.912607
H	6.974273	-0.756803	-0.074183
H	6.148580	-1.487193	-2.697856
H	7.770610	-0.880814	-2.295643
H	7.230470	-2.465470	-1.674676
H	1.931568	0.325439	-0.134347
H	1.338139	0.069033	0.394405
Fe	-3.924151	0.686323	0.507651
Ir	0.404897	0.239782	-1.158136
P	-1.033613	-1.168108	0.103674
S	-0.650084	2.324659	-0.369530
C	-2.277136	-0.325874	1.129091
C	-2.224317	1.045834	1.575483
C	-3.379288	1.279855	2.383356
H	-3.634756	2.232770	2.836701
C	-4.139335	0.080075	2.449238
H	-5.093310	-0.044739	2.950213
C	-3.468378	-0.907678	1.679937
H	-3.822543	-1.916764	1.498636
C	-4.186127	2.162521	-0.886836
H	-3.600798	3.074439	-0.938073
C	-3.868846	0.927237	-1.522983
H	-2.996765	0.735913	-2.141100
C	-4.864710	-0.027119	-1.156589
H	-4.883811	-1.073768	-1.446513
C	-5.797542	0.622104	-0.295810
H	-6.649513	0.156414	0.187980
C	-5.377179	1.974024	-0.127956
H	-5.855698	2.716614	0.501482
C	-1.154505	2.065377	1.387216
H	-0.243310	1.795075	1.940865
H	-1.505227	3.038465	1.753061
C	-0.102793	-2.151138	1.347423
C	0.658061	-3.250976	0.932136
H	0.616689	-3.582921	-0.106544
C	1.471180	-3.926878	1.838483
H	2.048858	-4.788545	1.508195
C	1.544213	-3.502495	3.165123
H	2.182589	-4.028774	3.871442
C	0.795547	-2.406462	3.582014
H	0.845539	-2.072380	4.616426
C	-0.025534	-1.733596	2.678645
H	-0.604283	-0.874324	3.018428
C	-2.010119	-2.431916	-0.798189
C	-2.567708	-3.527970	-0.127308
H	-2.373935	-3.676374	0.934614
C	-3.367869	-4.437597	-0.809990
H	-3.798248	-5.283318	-0.278189
C	-3.611310	-4.269793	-2.171895
H	-4.235263	-4.984027	-2.704801
C	-3.044434	-3.195596	-2.850637
H	-3.220517	-3.067130	-3.916408
C	-2.245604	-2.281776	-2.167228
H	-1.799689	-1.443255	-2.700026
C	0.556418	3.627737	-0.066867
C	0.126017	4.947515	-0.208618
H	-0.900239	5.156015	-0.507999
C	1.014609	5.991505	0.034366
H	0.680958	7.020381	-0.080333
C	2.323984	5.718683	0.421683
H	3.016286	6.536322	0.609896
C	2.746376	4.399642	0.567430
H	3.767742	4.175914	0.872094
C	1.866550	3.350075	0.320529
H	2.213749	2.325038	0.433316
H	1.304577	1.165168	-2.202470

TS$_{6-7}$- Li-3MeOH

E (BS1) = -2638.3974699

E (BS2) = -3778.8530698

$G_{298.15,1M}$ (BS2) = -3778.1861640

H	2.601015	-0.895834	1.960381
H	1.411408	-0.779720	-1.528587
H	-0.339591	0.307240	-2.345123
Li	4.395493	-0.371435	-0.565308
H	2.955296	-2.256333	-0.048923
O	3.650596	-2.215413	-0.724324
C	4.401589	-3.419532	-0.671766
H	5.166577	-3.367767	-1.452774
H	4.902124	-3.551188	0.297952
H	3.767786	-4.293474	-0.870018
H	2.903354	0.584042	-2.129353
O	3.879586	0.552179	-2.237589
C	4.345286	1.892738	-2.281412
H	5.439362	1.867941	-2.322247
H	3.977337	2.416988	-3.173248
H	4.043198	2.461773	-1.389669
O	3.605491	0.532483	0.864751
C	3.442121	-0.169851	2.061026
C	3.076091	0.768402	3.204918
C	4.680722	-0.983013	2.413688
H	2.146946	1.312381	2.982160
H	3.872568	1.512739	3.353296
H	2.933370	0.229334	4.152129
H	4.542958	-1.544890	3.348221
H	4.917648	-1.705795	1.620227
H	5.550984	-0.321770	2.542966
O	6.322181	-0.645117	-0.715098
C	6.916757	-0.962706	-1.966708
H	6.969838	-0.197649	-0.156969
H	6.116445	-1.282218	-2.641982
H	7.418401	-0.093750	-2.409449
H	7.641796	-1.780644	-1.869363
H	2.149308	0.628698	0.442001
H	1.290909	0.651091	0.630497
Fe	-3.985320	0.236232	0.419982
Ir	0.500752	0.441514	-0.982991
P	-0.822863	-1.163543	0.112683
S	-0.946423	2.322877	-0.337156
C	-2.223799	-0.526984	1.090704
C	-2.393993	0.830396	1.549263
C	-3.597868	0.878996	2.317396
H	-4.009753	1.777526	2.766644
C	-4.170835	-0.422172	2.347026
H	-5.111614	-0.695385	2.812928
C	-3.332796	-1.287102	1.593617
H	-3.524346	-2.335679	1.392371
C	-4.418388	1.678195	-0.966586
H	-3.973260	2.667304	-0.988787
C	-3.902155	0.512983	-1.604992
H	-2.994599	0.460541	-2.198637

C	-4.756700	-0.583380	-1.281998
H	-4.610895	-1.616577	-1.583992
C	-5.802026	-0.092427	-0.446315
H	-6.590429	-0.686022	0.004131
C	-5.591890	1.303909	-0.250078
H	-6.194541	1.957258	0.371792
C	-1.490788	2.005430	1.398048
H	-0.576407	1.886089	1.997797
H	-2.009740	2.912963	1.731396
C	0.113851	-2.112633	1.380534
C	0.983094	-3.136982	0.982790
H	1.021230	-3.445180	-0.064422
C	1.793228	-3.778403	1.916164
H	2.456159	-4.580368	1.595902
C	1.744503	-3.404529	3.258831
H	2.375241	-3.907627	3.988543
C	0.883049	-2.388772	3.660775
H	0.837265	-2.094010	4.707303
C	0.073315	-1.744001	2.727032
H	-0.590174	-0.943292	3.055692
C	-1.601322	-2.494650	-0.890454
C	-2.058535	-3.679389	-0.298601
H	-1.897465	-3.858556	0.763902
C	-2.714003	-4.641381	-1.059693
H	-3.065377	-5.555977	-0.586861
C	-2.913128	-4.437624	-2.423917
H	-3.423026	-5.192707	-3.018421
C	-2.446545	-3.272733	-3.024831
H	-2.587741	-3.112293	-4.091584
C	-1.792369	-2.307966	-2.261902
H	-1.424300	-1.398324	-2.734229
C	-0.007157	3.823459	-0.004196
C	-0.661578	5.041762	-0.190127
H	-1.694866	5.055644	-0.534801
C	0.012273	6.232900	0.064724
H	-0.496633	7.182660	-0.083993
C	1.332203	6.205783	0.506140
H	1.859191	7.137018	0.702004
C	1.979447	4.987170	0.696090
H	3.010920	4.963374	1.041620
C	1.314083	3.791487	0.441481
H	1.821283	2.839432	0.585641
H	1.417270	1.506465	-1.875025

7- Li-3MeOH
E (BS1) = -2638.4242141
E (BS2) = -3778.8762937
$G_{298.15,1M}$ (BS2) = -3778.2062948

H	2.820167	-1.152131	1.874688
H	1.440146	-0.609720	-1.598327
H	-0.406172	0.448808	-2.488819
Li	4.619270	-0.311109	-0.545730
H	3.040974	-2.170860	-0.279634
O	3.838153	-2.085193	-0.826001
C	4.532869	-3.324579	-0.819125
H	5.448903	-3.192602	-1.403406
H	4.808508	-3.635580	0.197820
H	3.936262	-4.117600	-1.287605
H	2.929254	0.817375	-1.864844
O	3.911192	0.859455	-1.930097
C	4.293019	2.222065	-1.811166
H	5.386970	2.263913	-1.775608
H	3.952481	2.809759	-2.673526
H	3.897453	2.683017	-0.894558
O	3.867013	0.446651	1.092177
C	3.607552	-0.446300	2.189859
C	3.123346	0.339831	3.388042
C	4.878443	-1.210800	2.472179
H	2.203566	0.891649	3.152374
H	3.887766	1.062107	3.704398
H	2.912527	-0.327798	4.232659
H	4.732093	-1.891733	3.318998
H	5.184474	-1.812191	1.606176
H	5.696600	-0.521871	2.723678
O	6.531732	-0.445299	-0.713272
C	6.991635	-0.625882	-2.050389
H	7.080310	0.221473	-0.279838
H	6.300275	-1.316702	-2.543510
H	7.001281	0.318991	-2.607226
H	7.996569	-1.062960	-2.066848
H	2.987896	0.747546	0.768188
H	1.403396	0.729535	0.366562
Fe	-3.981050	0.107533	0.587203
Ir	0.439320	0.543999	-1.044749
P	-0.782315	-1.150818	-0.004807
S	-1.066692	2.325495	-0.289176
C	-2.143146	-0.612934	1.091930
C	-2.335552	0.713741	1.625790
C	-3.481130	0.675745	2.479722
H	-3.895983	1.534705	2.998489
C	-3.998842	-0.648790	2.486480
H	-4.891535	-0.983472	3.004126
C	-3.184041	-1.440347	1.633472
H	-3.347264	-2.486376	1.396905
C	-4.560689	1.604824	-0.680157
H	-4.150195	2.609070	-0.673482
C	-4.051622	0.497265	-1.418993
H	-3.185773	0.512654	-2.074004
C	-4.843544	-0.646898	-1.102068
H	-4.686323	-1.654981	-1.474592
C	-5.843088	-0.243286	-0.169354
H	-6.577729	-0.890473	0.297932
C	-5.667434	1.147115	0.092137
H	-6.246301	1.741916	0.790666
C	-1.504267	1.939293	1.460164
H	-0.556703	1.850713	2.010313
H	-2.052777	2.808208	1.845192
C	0.212756	-2.190397	1.149452
C	1.062431	-3.180458	0.637675
H	1.069696	-3.392029	-0.433720
C	1.884269	-3.917114	1.487341
H	2.525029	-4.695775	1.077221
C	1.872608	-3.671584	2.859974
H	2.510574	-4.251045	3.523869
C	1.035075	-2.687291	3.374894
H	1.015705	-2.491765	4.445309
C	0.211760	-1.949512	2.525165
H	-0.433342	-1.176785	2.944791
C	-1.611068	-2.441765	-1.036051
C	-2.003165	-3.675576	-0.499261
H	-1.764316	-3.925130	0.533989
C	-2.691975	-4.599990	-1.277814
H	-2.990184	-5.553122	-0.845990
C	-2.991620	-4.310216	-2.607687
H	-3.526965	-5.035851	-3.216471
C	-2.591379	-3.095913	-3.156127
H	-2.810497	-2.866524	-4.197002

C	-1.903485	-2.170074	-2.374612
H	-1.583868	-1.221132	-2.804474
C	-0.187219	3.864770	0.031071
C	-0.810165	5.053130	-0.348311
H	-1.789869	5.024355	-0.822941
C	-0.174155	6.270933	-0.117255
H	-0.658094	7.197242	-0.418801
C	1.072281	6.299980	0.500191
H	1.567958	7.251227	0.681329
C	1.687017	5.110241	0.886648
H	2.661477	5.130875	1.370389
C	1.063325	3.889964	0.649063
H	1.546221	2.955739	0.932920
H	1.343476	1.689221	-1.858970

C2. Sodium system

1-Na-3MeOH

E (BS1) = -2598.8919954

E (BS2) = -3739.2869435

$G_{298.15,1M}$ (BS2) = -3738.7235326

Na	-3.589363	1.472006	-0.246920
H	-4.527844	1.265335	2.522127
O	-3.967203	1.877372	2.028869
C	-2.854112	2.221549	2.842676
H	-2.207634	2.882610	2.255131
H	-3.168273	2.759487	3.747154
H	-2.273546	1.336975	3.140547
H	-6.235335	0.569833	-0.992273
H	-3.209501	3.381591	-2.301690
O	-4.007690	2.886051	-2.075138
O	-5.431100	0.114624	-0.711904
C	-4.403771	2.141928	-3.221046
H	-5.316942	1.598958	-2.957196
H	-4.624074	2.801023	-4.070170
H	-3.634686	1.417333	-3.523349
C	-5.284670	-1.058046	-1.501123
H	-4.357582	-1.551212	-1.189126
H	-6.120036	-1.753645	-1.347180
H	-5.211013	-0.822929	-2.572102
Fe	3.145151	-1.870689	0.403322
Ir	-0.813654	0.295054	-0.872317
P	1.202939	0.992055	0.079475
S	-0.596550	-2.017849	-0.087111
C	2.113418	-0.247254	1.065284
C	1.558027	-1.458028	1.618974
C	2.594069	-2.113728	2.353173
H	2.486589	-3.069046	2.858025
C	3.776848	-1.328480	2.269389
H	4.744995	-1.585235	2.686102
C	3.486658	-0.184075	1.478456
H	4.197994	0.583889	1.193909
C	2.635830	-3.355400	-0.908978
H	1.718434	-3.932118	-0.861980
C	2.811519	-2.119971	-1.598015
H	2.047350	-1.592016	-2.160859
C	4.144503	-1.668205	-1.362089
H	4.575203	-0.736326	-1.716717
C	4.791522	-2.628139	-0.529564
H	5.800498	-2.555069	-0.137693
C	3.859046	-3.669462	-0.248304
H	4.036629	-4.527587	0.391090
C	0.151956	-1.951904	1.596407
H	-0.492384	-1.306186	2.209533
H	0.113082	-2.971354	1.999648
C	0.955614	2.331124	1.322771
C	0.611748	3.614779	0.881600
H	0.589444	3.834384	-0.186628
C	0.298610	4.616156	1.794406
H	0.039925	5.611099	1.437429
C	0.313744	4.345881	3.162613
H	0.065243	5.128534	3.876223
C	0.650575	3.072234	3.609467
H	0.667798	2.853780	4.675503
C	0.973310	2.070414	2.694785
H	1.232832	1.076564	3.060268
C	2.538378	1.701113	-0.978250
C	3.556010	2.501684	-0.443231
H	3.541955	2.770668	0.612685
C	4.589356	2.964942	-1.251024
H	5.374470	3.583729	-0.821366
C	4.614209	2.644481	-2.607241
H	5.421302	3.010288	-3.238601
C	3.596498	1.868107	-3.152992
H	3.602062	1.625980	-4.213823
C	2.563854	1.401647	-2.342396
H	1.762623	0.797793	-2.768776
C	-2.218605	-2.647054	0.382438
C	-2.630041	-3.855482	-0.177479
H	-1.970917	-4.390101	-0.859478
C	-3.882897	-4.375940	0.143239
H	-4.203664	-5.316655	-0.298768
C	-4.711870	-3.701685	1.033850
H	-5.685605	-4.113188	1.290360
C	-4.293744	-2.496555	1.597302
H	-4.940439	-1.964605	2.292831
C	-3.054035	-1.960591	1.265654
H	-2.740784	-1.004497	1.682330
H	-1.037726	1.794047	-1.453982
H	-2.305341	-0.071254	-1.516488
H	-1.580311	0.735441	0.575044
H	-0.175068	-0.105479	-2.397021

2- Na-3MeOH

E (BS1) = -2791.9493702

E (BS2) = -3932.4170488

$G_{298.15,1M}$ (BS2) = -3931.7735209

Na	3.357499	-1.145997	-0.957377
H	2.463838	-3.875675	-0.226068
O	3.171775	-3.274492	0.038403
C	3.934800	-3.899000	1.056472
H	4.730357	-3.204634	1.344169
H	3.328338	-4.121744	1.946935
H	4.398729	-4.830237	0.705559
H	4.225837	0.617311	-2.964004
H	2.422508	-2.911752	-2.878875
O	3.253368	-2.439369	-3.025153
O	4.721789	0.459487	-2.149235
C	3.154705	-1.774998	-4.276294
H	4.126284	-1.317446	-4.486573
H	2.927362	-2.476195	-5.089453
H	2.385432	-0.989284	-4.263719
C	4.747152	1.684946	-1.426529
H	5.345159	1.525676	-0.523900
H	5.210867	2.489299	-2.012954
H	3.735275	1.996112	-1.129091

O	4.561083	-0.506037	0.996164
C	4.197243	-0.300492	2.150849
C	4.946937	0.632770	3.045436
C	3.002225	-0.978802	2.740817
H	4.257832	1.308095	3.567068
H	5.693308	1.202170	2.484452
H	5.453495	0.045908	3.823275
H	2.257673	-0.219667	3.018730
H	2.559873	-1.691569	2.037936
H	3.286308	-1.489290	3.670969
Fe	-3.669039	1.472246	0.579177
Ir	0.443982	0.064125	-1.068292
P	-1.254494	-0.990721	0.135707
S	-0.071692	2.294998	-0.186657
C	-2.309926	0.078079	1.175197
C	-1.945333	1.380549	1.674482
C	-3.026076	1.855920	2.479097
H	-3.057033	2.825118	2.967653
C	-4.049384	0.868019	2.491954
H	-5.015210	0.955566	2.978237
C	-3.614625	-0.223210	1.691603
H	-4.194257	-1.112231	1.466590
C	-3.553119	2.989326	-0.792248
H	-2.760112	3.727908	-0.835044
C	-3.554469	1.723965	-1.448108
H	-2.754491	1.330358	-2.068565
C	-4.756435	1.042846	-1.091262
H	-5.035072	0.037574	-1.394620
C	-5.497955	1.891091	-0.217081
H	-6.439315	1.645440	0.263042
C	-4.753236	3.092532	-0.030711
H	-5.031848	3.921737	0.610783
C	-0.645541	2.099778	1.550366
H	0.134727	1.554475	2.097841
H	-0.734950	3.107624	1.973890
C	-0.632893	-2.196946	1.384777
C	-0.098332	-3.415276	0.949385
H	-0.131584	-3.674573	-0.110210
C	0.472839	-4.302255	1.855752
H	0.877168	-5.250058	1.503209
C	0.528505	-3.979450	3.211567
H	0.977962	-4.672586	3.919787
C	0.001777	-2.769940	3.652821
H	0.036421	-2.511659	4.709527
C	-0.578628	-1.884567	2.745227
H	-0.987488	-0.940794	3.107091
C	-2.515117	-2.001794	-0.757776
C	-3.294142	-2.959401	-0.095292
H	-3.124277	-3.166853	0.960950
C	-4.287123	-3.656756	-0.775356
H	-4.886467	-4.395544	-0.247212
C	-4.508426	-3.415390	-2.130119
H	-5.283731	-3.963575	-2.661358
C	-3.726601	-2.481192	-2.802478
H	-3.885253	-2.296616	-3.863063
C	-2.734711	-1.780591	-2.119379
H	-2.119338	-1.049630	-2.643789
C	1.458549	3.193431	0.107193
C	1.729676	4.260089	-0.750496
H	1.012783	4.527870	-1.525519
C	2.912584	4.981183	-0.610333
H	3.120898	5.809197	-1.284409
C	3.812996	4.654898	0.399401
H	4.730711	5.226425	0.518950
C	3.535418	3.593131	1.258232
H	4.239288	3.333114	2.046912
C	2.368691	2.849204	1.108104
H	2.185809	1.997826	1.763370
H	0.818596	-1.359065	-1.744326
H	1.729564	0.681069	-1.930987
H	1.521902	-0.222790	0.206363
H	-0.509733	0.381501	-2.440687

$TS_{2\text{-}3}$- Na-3MeOH

E (BS1) = -2791.9310242

E (BS2) = -3932.3981819

$G_{298.15,1M}$ (BS2) = -3931.7539050

H	-1.630674	-0.310127	-0.941964
Na	-3.884953	-0.949298	0.736432
H	-4.264451	-3.884383	0.997534
O	-4.640022	-3.108270	0.564717
C	-5.463981	-3.535129	-0.509289
H	-5.787334	-2.641502	-1.053296
H	-4.922595	-4.188769	-1.205679
H	-6.356044	-4.063759	-0.149159
H	-5.973599	0.362182	2.285576
H	-2.271843	-0.858676	2.657543
O	-3.104040	-1.102715	3.110831
O	-5.692464	0.381302	1.362372
C	-3.502979	0.007007	3.896349
H	-4.427420	-0.262524	4.416895
H	-2.747696	0.263839	4.652326
H	-3.695768	0.901838	3.282981
C	-5.503401	1.736980	0.980127
H	-5.164838	1.734434	-0.060957
H	-6.438977	2.308091	1.041765
H	-4.742848	2.235181	1.598846
O	-3.882692	-0.144049	-1.435444
C	-2.799175	-0.542593	-1.969672
C	-2.201070	0.287484	-3.085663
C	-2.602308	-2.033317	-2.144340
H	-1.171361	-0.009176	-3.318434
H	-2.236022	1.356366	-2.848132
H	-2.813431	0.121822	-3.985370
H	-1.581146	-2.284565	-2.447540
H	-2.863399	-2.586989	-1.234279
H	-3.286460	-2.357155	-2.944187
Fe	3.729079	1.272315	-0.274519
Ir	-0.780041	-0.064771	0.621537
P	1.187739	-1.123739	-0.104787
S	0.100878	2.159616	0.110725
C	2.389949	-0.080614	-1.001062
C	2.146762	1.227426	-1.558812
C	3.335125	1.634789	-2.240592
H	3.470832	2.594375	-2.730357
C	4.306121	0.602805	-2.118268
H	5.324405	0.636382	-2.490757
C	3.732795	-0.447786	-1.353720
H	4.237209	-1.360934	-1.055603
C	3.517289	2.870714	0.982679
H	2.769038	3.648282	0.867540
C	3.352393	1.650141	1.699980
H	2.456830	1.338738	2.228721
C	4.549613	0.887164	1.555208
H	4.724779	-0.107314	1.954602
C	5.454098	1.639655	0.750577
H	6.436540	1.316417	0.423025

C	4.816092	2.864316	0.395863
H	5.229154	3.634799	-0.246479
C	0.912346	2.062273	-1.542909
H	0.156985	1.683734	-2.245955
H	1.168780	3.090783	-1.827509
C	0.896534	-2.467446	-1.328297
C	0.353839	-3.681391	-0.890178
H	0.167054	-3.841892	0.172361
C	0.054707	-4.690163	-1.799087
H	-0.356416	-5.632824	-1.443594
C	0.281375	-4.494616	-3.160828
H	0.047412	-5.284061	-3.871772
C	0.808049	-3.286428	-3.606355
H	0.987602	-3.126370	-4.667524
C	1.116677	-2.277496	-2.694988
H	1.533517	-1.337860	-3.057865
C	2.262040	-1.978680	1.130020
C	3.173976	-2.969175	0.742521
H	3.215990	-3.299533	-0.294653
C	4.028813	-3.548480	1.674571
H	4.731606	-4.316040	1.357258
C	3.979019	-3.153695	3.010128
H	4.645384	-3.610028	3.739084
C	3.065196	-2.183319	3.409132
H	3.011611	-1.877954	4.452103
C	2.211114	-1.602030	2.474378
H	1.492321	-0.846465	2.789826
C	-1.149483	3.381678	-0.326408
C	-0.751891	4.720835	-0.345297
H	0.275963	4.986812	-0.098893
C	-1.672223	5.709826	-0.675340
H	-1.361985	6.752320	-0.688921
C	-2.988053	5.365336	-0.976695
H	-3.708295	6.140483	-1.228972
C	-3.381733	4.030658	-0.949353
H	-4.410343	3.758686	-1.181243
C	-2.464599	3.032688	-0.625008
H	-2.768772	1.987073	-0.600332
H	-1.341210	-1.528412	1.049279
H	-2.180493	0.627716	1.173970
H	-0.192544	0.142004	2.140729

3-Na-3MeOH

E (BS1) = -2791.9387533

E (BS2) = -3932.4087147

$G_{298.15,1M}$ (BS2) = -3931.7578258

H	2.024325	-0.406188	1.198350
Na	4.015256	-0.814280	-0.764627
H	4.150712	-3.698832	-1.267124
O	4.719376	-3.034540	-0.858387
C	5.549753	-3.675198	0.096899
H	6.131465	-2.898444	0.603435
H	4.966151	-4.217897	0.852543
H	6.248334	-4.375728	-0.379077
H	6.006715	0.655534	-2.448869
H	2.313702	-0.754390	-2.636985
O	3.137783	-1.045576	-3.075891
O	5.745107	0.570433	-1.523827
C	3.570759	0.003211	-3.924260
H	4.493652	-0.321064	-4.415792
H	2.830850	0.230536	-4.703844
H	3.784292	0.928043	-3.365634
C	5.531474	1.871507	-0.992105
H	5.130479	1.737404	0.018120
H	6.467171	2.443170	-0.930364
H	4.806546	2.444364	-1.588228
O	4.063603	-0.060912	1.365833
C	2.923264	-0.554777	1.908880
C	2.516657	0.147758	3.207585
C	2.968087	-2.069697	2.137049
H	1.543631	-0.200072	3.586712
H	2.466500	1.235700	3.063989
H	3.271454	-0.048414	3.984419
H	2.050072	-2.453362	2.605337
H	3.120777	-2.609558	1.190416
H	3.813610	-2.309419	2.800470
Fe	-3.811610	1.233309	0.197252
Ir	0.664705	-0.026247	-0.676306
P	-1.247313	-1.109060	0.174768
S	-0.205382	2.215454	-0.162332
C	-2.476430	-0.062326	1.018224
C	-2.254672	1.274543	1.513050
C	-3.458997	1.704795	2.150385
H	-3.610797	2.685340	2.591167
C	-4.417407	0.657696	2.063935
H	-5.442637	0.699767	2.415722
C	-3.820251	-0.426944	1.367500
H	-4.312868	-1.357332	1.105750
C	-3.584019	2.740405	-1.168218
H	-2.836059	3.523632	-1.103492
C	-3.415758	1.471013	-1.794603
H	-2.516740	1.118005	-2.290604
C	-4.616755	0.723224	-1.607182
H	-4.791485	-0.297352	-1.934761
C	-5.526037	1.533369	-0.866510
H	-6.512300	1.236420	-0.526022
C	-4.887684	2.778544	-0.594143
H	-5.304809	3.594684	-0.013992
C	-1.014395	2.099103	1.496294
H	-0.255224	1.700819	2.186110
H	-1.252445	3.127284	1.796831
C	-0.825918	-2.329868	1.481265
C	-0.271305	-3.562640	1.116278
H	-0.149679	-3.814628	0.062273
C	0.115087	-4.476440	2.090211
H	0.531861	-5.436929	1.794274
C	-0.029425	-4.162247	3.440690
H	0.274646	-4.877023	4.202234
C	-0.560714	-2.930683	3.811671
H	-0.673933	-2.678680	4.864072
C	-0.961423	-2.018526	2.837003
H	-1.385484	-1.060723	3.140152
C	-2.277739	-2.093991	-0.986484
C	-3.155674	-3.083239	-0.525363
H	-3.192759	-3.330716	0.535032
C	-3.981002	-3.763469	-1.414566
H	-4.659637	-4.528356	-1.043186
C	-3.933671	-3.471413	-2.776421
H	-4.577759	-4.006815	-3.470685
C	-3.052251	-2.502847	-3.246660
H	-3.002126	-2.278072	-4.309914
C	-2.227734	-1.819639	-2.355925
H	-1.537776	-1.064016	-2.728804
C	1.100419	3.356445	0.324039
C	0.815100	4.721988	0.273715
H	-0.159921	5.061620	-0.074716
C	1.780206	5.641934	0.671711

H	1.559905	6.706310	0.630343
C	3.022353	5.199810	1.121000
H	3.774525	5.920267	1.434659
C	3.300992	3.836507	1.168931
H	4.267853	3.483331	1.525369
C	2.343659	2.908232	0.766420
H	2.577444	1.843845	0.798318
H	1.216480	-1.482724	-1.135984
H	2.026387	0.673413	-1.325418
H	0.014211	0.125840	-2.082492

4-Na-3MeOH

E (BS1) = -2791.9513551

E (BS2) = -3932.4174148

$G_{298.15,1M}$ (BS2) = -3931.7670999

H	1.028939	-1.672657	2.093082
Na	3.671169	-0.861756	-0.531056
H	3.828224	0.911588	-2.616214
H	2.727194	-3.122871	-1.829608
O	3.598116	-2.731025	-1.983639
O	4.634154	0.648877	-2.148226
C	3.656898	-2.326345	-3.345234
H	4.652438	-1.907974	-3.524622
H	3.511846	-3.176076	-4.024248
H	2.903816	-1.558640	-3.575723
C	4.998329	1.713713	-1.280415
H	5.961353	1.457112	-0.827637
H	5.113598	2.659847	-1.824192
H	4.258862	1.860997	-0.476237
O	2.142915	-0.290346	1.040764
C	2.035048	-1.222765	2.073795
C	2.234107	-0.560814	3.432284
C	3.020173	-2.376689	1.898340
H	2.165617	-1.288125	4.253992
H	1.467352	0.209433	3.602858
H	3.219654	-0.072019	3.486834
H	2.928553	-3.123648	2.699458
H	2.844123	-2.902705	0.946270
H	4.059030	-2.009781	1.907837
O	5.908808	-1.276049	0.054826
C	6.757466	-1.689464	-1.005881
H	6.342659	-0.550935	0.522313
H	6.255487	-2.510751	-1.526600
H	6.945234	-0.879666	-1.724444
H	7.721003	-2.058199	-0.631093
Fe	-3.635817	1.600604	0.012815
Ir	0.625021	-0.093421	-0.675693
P	-1.358538	-1.032894	0.187276
S	0.023792	2.200245	0.032420
C	-2.546911	0.142820	0.923355
C	-2.209659	1.430607	1.476251
C	-3.405061	2.004142	2.005339
H	-3.473753	2.987534	2.460356
C	-4.474327	1.088096	1.802148
H	-5.515601	1.255520	2.056511
C	-3.952443	-0.054919	1.137527
H	-4.530091	-0.910809	0.804964
C	-3.127116	3.063665	-1.329280
H	-2.301512	3.755931	-1.208106
C	-3.057296	1.778791	-1.941749
H	-2.164758	1.321174	-2.358557
C	-4.346755	1.174945	-1.849706
H	-4.611246	0.176896	-2.187123
C	-5.212310	2.090384	-1.181708
H	-6.249711	1.910700	-0.920012
C	-4.458040	3.256215	-0.858480
H	-4.823125	4.120590	-0.314180
C	-0.858783	2.044663	1.633198
H	-0.242760	1.426369	2.301101
H	-0.948506	3.050239	2.061708
C	-1.196197	-2.261225	1.548551
C	-0.636111	-3.512518	1.262440
H	-0.419266	-3.787626	0.228959
C	-0.334363	-4.400922	2.287797
H	0.101903	-5.369955	2.053505
C	-0.580449	-4.046930	3.614291
H	-0.335983	-4.738594	4.417649
C	-1.145072	-2.809293	3.906304
H	-1.346875	-2.531181	4.938826
C	-1.456212	-1.920856	2.878382
H	-1.886167	-0.949134	3.121881
C	-2.428229	-1.959368	-1.001331
C	-3.421865	-2.832445	-0.538244
H	-3.531946	-3.017628	0.529754
C	-4.274680	-3.475301	-1.429362
H	-5.042125	-4.147512	-1.051278
C	-4.140985	-3.265349	-2.800279
H	-4.805899	-3.771126	-3.497241
C	-3.145649	-2.416406	-3.273310
H	-3.025454	-2.256063	-4.342790
C	-2.295291	-1.768965	-2.379418
H	-1.516960	-1.107578	-2.754978
C	1.506765	3.079076	0.530744
C	2.097291	3.884043	-0.446813
H	1.639274	3.965707	-1.432273
C	3.263918	4.583552	-0.158562
H	3.716350	5.211596	-0.923202
C	3.844480	4.486361	1.103999
H	4.753435	5.038659	1.331650
C	3.250687	3.686568	2.076696
H	3.695457	3.609308	3.066778
C	2.083425	2.978945	1.797188
H	1.636299	2.358673	2.569875
H	1.034096	-1.537168	-1.282457
H	1.933466	0.524217	-1.501420
H	-0.195021	0.165106	-2.015964

5-Na-3MeOH

E (BS1) = -2793.1281305

E (BS2) = -3933.5974031

$G_{298.15,1M}$ (BS2) = -3932.9308432

H	1.014427	0.052580	2.057497
H	1.339790	-0.835034	-1.863821
H	-0.428431	0.430364	-2.374344
Na	3.701896	-0.940879	-0.581767
H	3.677642	-3.864533	-0.775937
O	4.299489	-3.182746	-0.491054
C	4.913618	-3.607982	0.717274
H	5.608899	-2.820659	1.026327
H	4.177611	-3.758138	1.520962
H	5.478994	-4.537980	0.576646
H	3.012439	0.822786	-2.256922
O	3.989845	0.726304	-2.285610
C	4.533069	1.916759	-1.740206
H	5.618842	1.896732	-1.884078
H	4.140967	2.809694	-2.247402

H	4.322414	2.007451	-0.662078
O	2.252773	0.423995	0.441985
C	2.080267	0.269372	1.811322
C	2.480586	1.530810	2.574977
C	2.901208	-0.909206	2.335657
H	1.853150	2.390187	2.303483
H	3.522508	1.792527	2.327380
H	2.416859	1.394301	3.663757
H	2.731896	-1.090484	3.407297
H	2.642802	-1.833484	1.797646
H	3.976768	-0.712192	2.197784
O	5.835117	-0.380896	0.272320
C	6.938576	-0.648103	-0.580682
H	5.829685	0.563832	0.476133
H	6.985973	-1.731513	-0.730486
H	6.827634	-0.165953	-1.562457
H	7.885226	-0.322337	-0.131988
H	0.058998	0.097223	4.035789
H	-0.327630	0.333108	4.629385
Fe	-3.850397	0.451105	0.643748
Ir	0.572503	0.395641	-1.138129
P	-0.831216	-1.166465	-0.071853
S	-0.720352	2.308384	-0.264715
C	-2.092554	-0.494885	1.069401
C	-2.120269	0.826067	1.651895
C	-3.239403	0.884974	2.538829
H	-3.541075	1.766699	3.096289
C	-3.906670	-0.370530	2.514757
H	-4.818359	-0.619731	3.047277
C	-3.212815	-1.215503	1.608131
H	-3.497523	-2.228280	1.342786
C	-4.280135	2.053492	-0.553395
H	-3.748383	2.999098	-0.529117
C	-3.935276	0.919755	-1.344976
H	-3.095387	0.853040	-2.029614
C	-4.852166	-0.129835	-1.037703
H	-4.831822	-1.134975	-1.448502
C	-5.764218	0.358673	-0.057348
H	-6.558189	-0.209937	0.415051
C	-5.409625	1.706561	0.243243
H	-5.887696	2.341318	0.981746
C	-1.181365	1.972308	1.489618
H	-0.248541	1.810518	2.046593
H	-1.658209	2.885177	1.868771
C	-0.008766	-2.444317	0.972620
C	0.861727	-3.341494	0.340325
H	1.027230	-3.268681	-0.736204
C	1.494665	-4.343934	1.067666
H	2.151178	-5.050365	0.560858
C	1.286999	-4.448462	2.442691
H	1.784293	-5.231544	3.011307
C	0.439121	-3.549558	3.081457
H	0.271083	-3.623497	4.154076
C	-0.210801	-2.556444	2.349841
H	-0.883942	-1.870620	2.862572
C	-1.851494	-2.258727	-1.164495
C	-2.371515	-3.471262	-0.691910
H	-2.129563	-3.816695	0.312640
C	-3.194450	-4.253379	-1.496056
H	-3.590188	-5.190842	-1.110760
C	-3.502011	-3.843531	-2.791721
H	-4.141085	-4.458377	-3.421979
C	-2.976525	-2.651058	-3.278764
H	-3.200424	-2.328013	-4.293412
C	-2.156858	-1.866292	-2.470726
H	-1.746924	-0.936210	-2.860022
C	0.276678	3.781364	0.016241
C	-0.382755	4.991845	0.239064
H	-1.472141	5.025627	0.254044
C	0.358695	6.152806	0.434781
H	-0.153753	7.096599	0.607989
C	1.750901	6.108105	0.396754
H	2.327030	7.018985	0.544731
C	2.403316	4.899726	0.168353
H	3.491191	4.860378	0.141107
C	1.668844	3.731919	-0.022223
H	2.160586	2.773337	-0.177640
H	1.474412	1.444461	-2.069358

$TS_{5\text{-}6}$-Na-3MeOH

E (BS1) = -2793.1070306

E (BS2) = -3933.5793033

$G_{298.15,1M}$ (BS2) = -3932.9169714

H	2.543108	-0.667270	2.347825
H	1.404125	-0.739871	-1.322811
H	-0.382099	0.247966	-2.115803
Na	3.912595	-0.495895	-0.806812
H	3.301820	-3.080649	0.053273
O	3.681101	-2.849284	-0.805342
C	4.794818	-3.701764	-1.032587
H	5.188686	-3.474916	-2.027756
H	5.592685	-3.542593	-0.293973
H	4.500915	-4.758835	-1.013354
H	2.769706	1.143124	-2.427602
O	3.741211	1.135568	-2.546743
C	4.240128	2.300398	-1.903914
H	5.307868	2.381771	-2.132264
H	3.740420	3.209809	-2.265358
H	4.122353	2.250616	-0.809402
O	3.567322	0.605777	1.077122
C	3.514805	-0.118235	2.249410
C	3.612126	0.774128	3.489623
C	4.607791	-1.189782	2.323737
H	2.846269	1.561644	3.462449
H	4.595034	1.269317	3.529286
H	3.480606	0.203987	4.422083
H	4.555510	-1.776398	3.253582
H	4.533049	-1.897272	1.483148
H	5.602821	-0.718838	2.278604
O	6.212956	-0.670653	-1.263390
C	6.553997	-0.755669	-2.638479
H	6.781829	-0.010075	-0.847788
H	5.822139	-1.411969	-3.122220
H	6.518219	0.223069	-3.135577
H	7.552727	-1.188778	-2.782617
H	0.886419	1.130082	1.742939
H	0.794826	0.878904	2.443699
Fe	-4.130130	0.168354	0.072077
Ir	0.433278	0.463891	-0.819048
P	-0.921036	-1.107883	0.302994
S	-1.071546	2.357621	-0.350554
C	-2.458686	-0.463031	1.044619
C	-2.755864	0.911948	1.375894
C	-4.062617	0.942899	1.955246
H	-4.576106	1.844854	2.274165
C	-4.577034	-0.381406	1.991578
H	-5.564184	-0.674811	2.332356

C	-3.599420	-1.245489	1.431506
H	-3.715075	-2.313054	1.278814
C	-4.398424	1.510855	-1.446982
H	-3.991481	2.516445	-1.457940
C	-3.747081	0.338143	-1.928970
H	-2.755150	0.298688	-2.369411
C	-4.599944	-0.777641	-1.674352
H	-4.372727	-1.817720	-1.889468
C	-5.779185	-0.291017	-1.038245
H	-6.605719	-0.895343	-0.679844
C	-5.653906	1.121953	-0.896209
H	-6.368992	1.779376	-0.413343
C	-1.910541	2.135347	1.279672
H	-1.124924	2.139416	2.046375
H	-2.538135	3.023079	1.426084
C	-0.101860	-1.927758	1.728806
C	0.902840	-2.868259	1.469850
H	1.101064	-3.175065	0.440930
C	1.633494	-3.427618	2.512773
H	2.406355	-4.164628	2.298759
C	1.377830	-3.042263	3.828532
H	1.953218	-3.474179	4.644625
C	0.381701	-2.107203	4.093931
H	0.172906	-1.808171	5.119155
C	-0.358799	-1.554591	3.049891
H	-1.135769	-0.822165	3.271176
C	-1.512955	-2.544938	-0.683598
C	-1.992439	-3.704898	-0.061608
H	-1.964618	-3.794996	1.023818
C	-2.501327	-4.754680	-0.818882
H	-2.873926	-5.647577	-0.321446
C	-2.526122	-4.666168	-2.209380
H	-2.921623	-5.489268	-2.800710
C	-2.030815	-3.528375	-2.838604
H	-2.033564	-3.458004	-3.924300
C	-1.525801	-2.475110	-2.079582
H	-1.137179	-1.588933	-2.578266
C	-0.114463	3.823959	0.067340
C	-0.763905	5.059214	0.065964
H	-1.824633	5.117939	-0.175193
C	-0.046629	6.213204	0.365577
H	-0.551643	7.176546	0.363533
C	1.313854	6.135507	0.654757
H	1.873447	7.040656	0.880160
C	1.958389	4.901543	0.651410
H	3.021687	4.836270	0.874336
C	1.246502	3.740737	0.361860
H	1.746143	2.772009	0.355291
H	1.320864	1.525223	-1.745102

6-Na-3MeOH

E (BS1) = -2793.1181772

E (BS2) = -3933.5899235

$G_{298.15,1M}$ (BS2) = -3932.9246706

H	2.710478	-0.727278	1.882387
H	1.403261	-0.747481	-1.400837
H	-0.331408	0.309487	-2.271910
Na	4.001871	-0.267509	-0.635187
H	3.508600	-2.644999	0.702389
O	3.819140	-2.557437	-0.211016
C	4.924509	-3.426804	-0.398393
H	5.206648	-3.381499	-1.454545
H	5.791625	-3.135360	0.211256
H	4.663670	-4.466496	-0.160107
H	2.641933	0.688136	-2.575581
O	3.600313	0.665120	-2.772342
C	4.081149	1.995264	-2.643447
H	5.133809	2.004699	-2.942309
H	3.533498	2.689662	-3.294279
H	4.008541	2.366236	-1.607338
O	3.707900	0.956207	1.186849
C	3.459700	0.057189	2.198614
C	2.851187	0.734286	3.428175
C	4.713225	-0.721424	2.606655
H	1.906981	1.234775	3.166356
H	3.539857	1.503649	3.809192
H	2.645743	0.026999	4.245272
H	4.499793	-1.483255	3.371795
H	5.153150	-1.234334	1.737722
H	5.474292	-0.036242	3.010071
O	6.274538	-0.407383	-1.054392
C	6.854743	-0.828642	-2.276631
H	6.979022	-0.228011	-0.420160
H	6.038926	-1.020817	-2.981416
H	7.507339	-0.057821	-2.707849
H	7.435300	-1.753551	-2.157039
H	1.768383	0.750996	0.450771
H	1.051038	0.552535	0.832679
Fe	-4.088805	0.168839	0.319774
Ir	0.442766	0.453899	-0.880893
P	-0.911156	-1.196337	0.147238
S	-1.059593	2.306149	-0.298137
C	-2.352181	-0.573740	1.068104
C	-2.557805	0.781743	1.520354
C	-3.795407	0.813329	2.233962
H	-4.237958	1.705819	2.665687
C	-4.353251	-0.494510	2.236517
H	-5.310664	-0.779690	2.659159
C	-3.472645	-1.348504	1.520593
H	-3.641783	-2.399046	1.309622
C	-4.483719	1.608228	-1.081657
H	-4.055828	2.605146	-1.081606
C	-3.919178	0.454159	-1.698997
H	-2.985414	0.419482	-2.251901
C	-4.768915	-0.657462	-1.417956
H	-4.593330	-1.686913	-1.716520
C	-5.858735	-0.187116	-0.628730
H	-6.656314	-0.795720	-0.216102
C	-5.681515	1.211814	-0.419610
H	-6.321992	1.852840	0.176665
C	-1.664866	1.970046	1.412984
H	-0.773028	1.863014	2.048036
H	-2.210001	2.868285	1.729085
C	0.001866	-2.126363	1.442360
C	0.885916	-3.145825	1.072971
H	0.963863	-3.450589	0.028855
C	1.671290	-3.779004	2.032554
H	2.343848	-4.582160	1.734109
C	1.592370	-3.390930	3.369448
H	2.206761	-3.885290	4.118810
C	0.719961	-2.372487	3.741911
H	0.649380	-2.067493	4.784046
C	-0.071752	-1.742242	2.783985
H	-0.747464	-0.942149	3.088773
C	-1.618518	-2.523641	-0.905003
C	-2.089814	-3.718330	-0.345069
H	-1.982738	-3.902860	0.723193

C	-2.688841	-4.683762	-1.146919
H	-3.051687	-5.606729	-0.699857
C	-2.816971	-4.472481	-2.518468
H	-3.283021	-5.230238	-3.144648
C	-2.336053	-3.296817	-3.086157
H	-2.421683	-3.130876	-4.157878
C	-1.737658	-2.328448	-2.283323
H	-1.357936	-1.411095	-2.730495
C	-0.157532	3.822508	0.063208
C	-0.846862	5.022664	-0.117142
H	-1.877374	5.008866	-0.470265
C	-0.211436	6.230911	0.153325
H	-0.747353	7.166247	0.008605
C	1.105835	6.238744	0.603262
H	1.604223	7.183164	0.810448
C	1.787462	5.038369	0.786940
H	2.816705	5.042179	1.139331
C	1.161048	3.824438	0.518279
H	1.703529	2.891337	0.660952
H	1.338250	1.540664	-1.752287

TS_{6-7}-Na-3MeOH
E (BS1) = -2793.1170361
E (BS2) = -3933.5872241
$G_{298.15,1M}$ (BS2) = -3932.9224172

H	2.653302	-1.007178	1.872912
H	1.385679	-0.832069	-1.367471
H	-0.289490	0.317985	-2.246034
Na	3.952555	-0.446212	-0.870001
H	2.871770	-2.663159	0.307938
O	3.481970	-2.684963	-0.445729
C	4.315802	-3.822345	-0.302440
H	5.070402	-3.784600	-1.094040
H	4.830763	-3.833767	0.668811
H	3.748427	-4.756870	-0.412085
H	2.925735	1.113879	-2.653769
O	3.899093	1.061522	-2.684265
C	4.395560	2.251871	-2.085511
H	5.485779	2.248769	-2.186410
H	4.005557	3.148677	-2.584152
H	4.144345	2.310740	-1.014151
O	3.572971	0.654817	1.053283
C	3.459516	-0.254860	2.092495
C	3.051022	0.425752	3.397814
C	4.742660	-1.056461	2.296969
H	2.099811	0.964293	3.271963
H	3.813704	1.162137	3.693060
H	2.928379	-0.289529	4.224050
H	4.630612	-1.821980	3.079742
H	5.038133	-1.567186	1.370244
H	5.568317	-0.390012	2.589745
O	6.283668	-0.671559	-1.028211
C	6.855526	-0.681586	-2.328231
H	6.718762	0.018899	-0.510726
H	6.355136	-1.468013	-2.901595
H	6.715015	0.275065	-2.850367
H	7.928449	-0.910294	-2.296148
H	1.982377	0.632589	0.588226
H	1.166447	0.524282	0.834867
Fe	-4.080515	0.326535	0.295470
Ir	0.479757	0.408775	-0.844596
P	-0.959890	-1.172467	0.139866
S	-0.951431	2.321453	-0.262297
C	-2.388221	-0.505828	1.054298
C	-2.540111	0.850698	1.523257
C	-3.784193	0.928217	2.222086
H	-4.192266	1.833712	2.660699
C	-4.400203	-0.353140	2.197653
H	-5.374911	-0.601305	2.603904
C	-3.548835	-1.235349	1.480167
H	-3.762778	-2.273948	1.251836
C	-4.388132	1.810108	-1.080849
H	-3.914054	2.785644	-1.050050
C	-3.864782	0.644959	-1.713647
H	-2.922989	0.579660	-2.249891
C	-4.767265	-0.433418	-1.470219
H	-4.631627	-1.463184	-1.788205
C	-5.848662	0.068560	-0.688723
H	-6.679776	-0.512107	-0.302857
C	-5.613484	1.453779	-0.446987
H	-6.235169	2.110075	0.152625
C	-1.595439	1.999892	1.437551
H	-0.718201	1.848871	2.084108
H	-2.106106	2.918508	1.753134
C	-0.110217	-2.163836	1.435402
C	0.700902	-3.247355	1.076810
H	0.738186	-3.582883	0.038847
C	1.465240	-3.905615	2.037716
H	2.085283	-4.752445	1.747329
C	1.428390	-3.488858	3.367823
H	2.024491	-4.003866	4.117928
C	0.618972	-2.417119	3.731763
H	0.578688	-2.090678	4.768980
C	-0.144398	-1.756474	2.771227
H	-0.764730	-0.910344	3.069439
C	-1.717369	-2.453137	-0.939324
C	-2.258369	-3.630242	-0.405418
H	-2.178130	-3.836425	0.661189
C	-2.893659	-4.552472	-1.230180
H	-3.310021	-5.461686	-0.801809
C	-2.989563	-4.316436	-2.600322
H	-3.483899	-5.040507	-3.244426
C	-2.439555	-3.159502	-3.142878
H	-2.499398	-2.973917	-4.213187
C	-1.805375	-2.234583	-2.316687
H	-1.372782	-1.331763	-2.744829
C	0.004559	3.796078	0.134678
C	-0.619732	5.029003	-0.059293
H	-1.637495	5.067129	-0.445927
C	0.063721	6.203638	0.241483
H	-0.422089	7.164572	0.087528
C	1.364182	6.145683	0.734821
H	1.899479	7.063978	0.965920
C	1.981136	4.912790	0.931625
H	2.997483	4.864651	1.317129
C	1.305596	3.732762	0.633505
H	1.794627	2.772264	0.785250
H	1.493092	1.430287	-1.680095

7-Na-3MeOH
E (BS1) = -2793.1480261
E (BS2) = -3933.6140031
$G_{298.15,1M}$ (BS2) = -3932.9482752

H	2.709136	-1.008787	1.939702
H	1.582058	-0.499959	-1.222690
H	-0.124697	0.492542	-2.392433

Na	3.986648	-0.339411	-0.916961
H	3.571459	-2.804575	0.299459
O	4.029835	-2.612204	-0.532361
C	5.177691	-3.443465	-0.617252
H	5.676369	-3.227024	-1.566693
H	5.885418	-3.254970	0.201790
H	4.904572	-4.506526	-0.608472
H	2.770089	1.442883	-2.311811
O	3.740457	1.431069	-2.447571
C	4.259763	2.581131	-1.795859
H	5.341614	2.605386	-1.962447
H	3.828027	3.506074	-2.201189
H	4.073607	2.560798	-0.709530
O	3.636671	0.671017	1.169861
C	3.476654	-0.271793	2.240488
C	3.014584	0.440532	3.494349
C	4.795390	-0.982868	2.431585
H	2.060599	0.958781	3.323692
H	3.759378	1.185480	3.805536
H	2.873287	-0.268985	4.319396
H	4.718404	-1.720275	3.239926
H	5.097729	-1.512432	1.518463
H	5.586483	-0.267359	2.694715
O	6.296423	-0.148370	-0.976828
C	6.867008	-0.169860	-2.278070
H	6.637535	0.624526	-0.508010
H	6.416704	-1.005590	-2.823394
H	6.664125	0.756291	-2.832988
H	7.952048	-0.329788	-2.241313
H	2.725038	0.940178	0.914762
H	1.100304	0.769618	0.764877
Fe	-4.106000	-0.083154	0.367742
Ir	0.447373	0.591703	-0.804104
P	-0.807578	-1.170490	0.064265
S	-1.244836	2.266626	-0.271492
C	-2.283177	-0.701133	1.036102
C	-2.594897	0.613491	1.543622
C	-3.807549	0.512109	2.293398
H	-4.314459	1.347076	2.767699
C	-4.249900	-0.838933	2.262144
H	-5.165029	-1.222268	2.700769
C	-3.320486	-1.584623	1.489002
H	-3.404264	-2.638274	1.245549
C	-4.652337	1.382933	-0.949996
H	-4.297769	2.407812	-0.911051
C	-4.021727	0.306155	-1.638588
H	-3.105715	0.371156	-2.217405
C	-4.775480	-0.879610	-1.389535
H	-4.533262	-1.876518	-1.745492
C	-5.872749	-0.532590	-0.548319
H	-6.609355	-1.219583	-0.145348
C	-5.795919	0.864777	-0.275721
H	-6.464148	1.425961	0.368733
C	-1.830871	1.888214	1.435370
H	-0.938852	1.876802	2.078869
H	-2.472743	2.725467	1.738449
C	0.140543	-2.159201	1.299910
C	1.063570	-3.116689	0.862773
H	1.157573	-3.337130	-0.201557
C	1.864984	-3.797201	1.776406
H	2.565520	-4.553958	1.422650
C	1.761960	-3.522285	3.140035
H	2.387035	-4.054580	3.853832
C	0.849062	-2.569577	3.581650
H	0.757870	-2.351583	4.644006
C	0.043336	-1.892376	2.667390
H	-0.660967	-1.142260	3.029139
C	-1.475641	-2.495900	-1.034535
C	-1.865495	-3.745998	-0.535441
H	-1.719610	-3.986222	0.517105
C	-2.430536	-4.698686	-1.376727
H	-2.729027	-5.664675	-0.974638
C	-2.605832	-4.420363	-2.731151
H	-3.044760	-5.167860	-3.388663
C	-2.204129	-3.189725	-3.241107
H	-2.324948	-2.970167	-4.299949
C	-1.639636	-2.235669	-2.396925
H	-1.314401	-1.275165	-2.796588
C	-0.514990	3.872675	0.098503
C	-1.241915	5.004441	-0.269670
H	-2.205006	4.892782	-0.766168
C	-0.729864	6.271759	-0.001837
H	-1.294397	7.154339	-0.294263
C	0.498071	6.405388	0.638640
H	0.897763	7.395400	0.846980
C	1.216441	5.271712	1.014225
H	2.175575	5.375244	1.517431
C	0.715775	4.002455	0.742869
H	1.278210	3.112125	1.021864
H	1.438791	1.818042	-1.343291

C3. Potassium system

1-K-3MeOH

E (BS1) = -3036.4841071
E (BS2) = -4176.9047972
$G_{298.15,1M}$ (BS2) = -4176.346738

K	4.009921	-1.416105	0.007619
H	2.064604	-3.650659	1.276783
O	2.958440	-3.391670	1.546967
C	2.870087	-2.838703	2.850244
H	3.883678	-2.569093	3.167876
H	2.241134	-1.934961	2.876188
H	2.473868	-3.561472	3.576755
H	4.888948	0.477170	-2.406550
H	2.595067	-3.356033	-2.036163
O	3.539763	-3.151947	-2.060360
O	5.402331	0.346648	-1.598109
C	3.789061	-2.394294	-3.234359
H	4.856285	-2.149935	-3.251258
H	3.556217	-2.963245	-4.144315
H	3.209338	-1.458375	-3.251948
C	5.355134	1.555504	-0.852990
H	5.946294	1.407263	0.057461
H	5.793296	2.395922	-1.407910
H	4.324850	1.822191	-0.570448
Fe	-3.355209	1.669906	0.357591
Ir	0.922102	-0.052212	-0.675811
P	-1.087198	-0.960106	0.082986
S	0.339179	2.226451	0.035804
C	-2.174858	0.155390	1.040385
C	-1.787465	1.409946	1.638728
C	-2.922752	1.928340	2.334354
H	-2.948038	2.879062	2.858290
C	-4.003865	1.016912	2.181304
H	-5.012726	1.153262	2.556192
C	-3.550238	-0.069220	1.385536

H	-4.155908	-0.907207	1.057155
C	-2.972259	3.252062	-0.883458
H	-2.138776	3.936715	-0.770394
C	-2.957655	2.029742	-1.616360
H	-2.109643	1.622240	-2.158190
C	-4.233779	1.408997	-1.465892
H	-4.526999	0.445457	-1.872570
C	-5.035915	2.250774	-0.641078
H	-6.045953	2.039542	-0.306197
C	-4.255965	3.388375	-0.280065
H	-4.569881	4.194190	0.374851
C	-0.446585	2.062256	1.688068
H	0.229446	1.473591	2.321869
H	-0.539745	3.071569	2.107963
C	-0.846495	-2.343615	1.280004
C	-0.412825	-3.588675	0.805777
H	-0.281438	-3.747574	-0.265685
C	-0.157640	-4.633865	1.689620
H	0.164386	-5.599969	1.305390
C	-0.319214	-4.444028	3.062696
H	-0.120196	-5.260557	3.753423
C	-0.738597	-3.207527	3.541944
H	-0.867574	-3.051400	4.611063
C	-1.002517	-2.163449	2.656062
H	-1.333466	-1.201184	3.047444
C	-2.283204	-1.724654	-1.100923
C	-3.266319	-2.627368	-0.674576
H	-3.304511	-2.943894	0.367240
C	-4.198675	-3.135475	-1.573255
H	-4.956958	-3.834541	-1.226510
C	-4.156100	-2.757304	-2.914038
H	-4.883372	-3.158325	-3.616927
C	-3.172066	-1.876529	-3.352065
H	-3.124543	-1.586520	-4.399795
C	-2.240604	-1.366048	-2.450111
H	-1.464968	-0.681135	-2.792974
C	1.852569	3.090742	0.475874
C	2.318589	4.029764	-0.445521
H	1.746323	4.233071	-1.349894
C	3.511692	4.705205	-0.204939
H	3.871720	5.433743	-0.928201
C	4.231452	4.460020	0.961339
H	5.158015	4.996177	1.154282
C	3.757129	3.529622	1.883309
H	4.313699	3.335315	2.797960
C	2.574534	2.835352	1.642326
H	2.231035	2.097550	2.363952
H	1.376117	-1.520579	-1.176555
H	2.412441	0.470532	-1.187158
H	1.636032	-0.368195	0.843737
H	0.385912	0.253648	-2.254207

2-K-3MeOH
E (BS1) = -3229.5402238
E (BS2) = -4370.0352364
$G_{298.15,1M}$ (BS2) = -4369.394167

K	3.714415	-1.036359	-0.989118
H	2.622563	-3.942543	0.063778
O	3.347900	-3.355032	0.311736
C	4.023309	-3.925624	1.417619
H	4.769717	-3.201280	1.761041
H	3.338908	-4.137045	2.252594
H	4.542587	-4.855193	1.146380
H	4.161325	1.065886	-3.408406
H	2.017808	-2.700634	-2.988800
O	2.948668	-2.543262	-3.200279
O	4.767950	0.869942	-2.682605
C	2.992344	-1.667084	-4.315125
H	4.043353	-1.432439	-4.512064
H	2.571942	-2.132855	-5.217158
H	2.449279	-0.728890	-4.123859
C	4.709226	1.950958	-1.764049
H	5.382648	1.716084	-0.931392
H	5.044865	2.893948	-2.216444
H	3.693533	2.094904	-1.363677
O	4.566623	-0.061264	1.422962
C	4.075647	-0.124960	2.545638
C	4.622356	0.677851	3.683350
C	2.891208	-0.987749	2.850910
H	3.834885	1.325807	4.090455
H	5.476295	1.282067	3.365881
H	4.925340	0.009365	4.498958
H	2.043475	-0.344359	3.127151
H	2.613940	-1.606933	1.991008
H	3.098198	-1.618293	3.725241
Fe	-3.746349	1.455117	0.636963
Ir	0.368768	0.072532	-1.077089
P	-1.334766	-0.996708	0.100073
S	-0.159107	2.298111	-0.183754
C	-2.373105	0.051505	1.178215
C	-2.005509	1.345519	1.698432
C	-3.073967	1.797840	2.532878
H	-3.100522	2.756660	3.041757
C	-4.093053	0.805704	2.542020
H	-5.050414	0.878141	3.047142
C	-3.668204	-0.265253	1.709941
H	-4.248462	-1.151556	1.475940
C	-3.649226	3.008244	-0.693438
H	-2.856855	3.748127	-0.724998
C	-3.658174	1.761375	-1.383915
H	-2.866482	1.385301	-2.025602
C	-4.855049	1.069471	-1.030507
H	-5.137409	0.073034	-1.358238
C	-5.586125	1.892709	-0.124201
H	-6.521354	1.633228	0.360598
C	-4.839886	3.089352	0.085465
H	-5.110801	3.900332	0.753053
C	-0.720453	2.087691	1.559408
H	0.080206	1.562386	2.097528
H	-0.826368	3.093942	1.983298
C	-0.712097	-2.246269	1.305004
C	-0.170349	-3.443654	0.821459
H	-0.196910	-3.659163	-0.247944
C	0.402937	-4.363552	1.692713
H	0.810677	-5.295986	1.304358
C	0.452806	-4.095749	3.061055
H	0.901530	-4.815970	3.742291
C	-0.079468	-2.907091	3.549347
H	-0.047922	-2.691177	4.615629
C	-0.661994	-1.988275	2.676636
H	-1.074225	-1.060726	3.074879
C	-2.611347	-1.973331	-0.810691
C	-3.390449	-2.947238	-0.172447
H	-3.211962	-3.190134	0.874843
C	-4.394320	-3.615586	-0.865793
H	-4.993638	-4.367704	-0.356749
C	-4.626782	-3.328422	-2.209772

H	-5.410875	-3.853679	-2.751206
C	-3.843859	-2.378878	-2.859039
H	-4.010099	-2.159567	-3.911857
C	-2.840667	-1.708198	-2.162709
H	-2.221793	-0.967695	-2.669363
C	1.376689	3.184062	0.133276
C	1.577423	4.387509	-0.541610
H	0.802085	4.777067	-1.199562
C	2.770121	5.087642	-0.369115
H	2.925627	6.023603	-0.901282
C	3.750203	4.597919	0.488513
H	4.677986	5.148578	0.627608
C	3.541925	3.398353	1.168074
H	4.312068	3.003679	1.829222
C	2.363001	2.684175	0.985139
H	2.221157	1.727280	1.485437
H	0.773206	-1.341650	-1.755448
H	1.667920	0.704686	-1.908761
H	1.432630	-0.251832	0.197537
H	-0.566206	0.408019	-2.464337

TS$_{2\text{-}3}$-K-3MeOH
E (BS1) = -3229.5243378
E (BS2) = -4370.0201606
$G_{298.15,1M}$ (BS2) = -4369.376299

K	4.186627	-1.403993	-0.654790
H	1.923942	-3.411044	-0.552475
O	2.824364	-3.766189	-0.604850
C	2.845178	-5.025318	0.043096
H	3.847778	-5.446146	-0.079603
H	2.637503	-4.939001	1.120714
H	2.124145	-5.725264	-0.400340
H	5.900708	0.721370	-2.608740
H	2.152601	-0.926998	-2.586214
O	2.874671	-1.197345	-3.189822
O	5.767933	0.499261	-1.678973
C	3.250761	-0.050867	-3.927115
H	4.057661	-0.334299	-4.611229
H	2.418591	0.343036	-4.528867
H	3.618755	0.759995	-3.278569
C	5.333608	1.666382	-0.999119
H	5.113975	1.380656	0.036596
H	6.107442	2.446456	-0.985876
H	4.418203	2.085729	-1.443505
O	3.854908	-0.181525	1.709938
C	2.717825	-0.522325	2.166248
C	2.069868	0.369037	3.210096
C	2.468010	-1.999873	2.402708
H	1.018184	0.110688	3.385095
H	2.150230	1.426797	2.936066
H	2.616616	0.224758	4.154896
H	1.426063	-2.210974	2.665025
H	2.747454	-2.602002	1.530908
H	3.102688	-2.308753	3.247347
Fe	-3.658726	1.492163	0.140626
Ir	0.821745	-0.059681	-0.523485
P	-1.219530	-1.005672	0.167958
S	0.026898	2.217289	-0.105278
C	-2.412729	0.115672	0.977587
C	-2.137273	1.434518	1.494834
C	-3.335256	1.917635	2.106726
H	-3.450132	2.900948	2.552754
C	-4.343754	0.922786	1.981415
H	-5.374463	1.013175	2.307389
C	-3.784011	-0.180921	1.284622
H	-4.314909	-1.083208	0.999956
C	-3.311084	3.018174	-1.175681
H	-2.526744	3.759919	-1.063603
C	-3.183363	1.758593	-1.830326
H	-2.284968	1.375476	-2.304218
C	-4.424884	1.066515	-1.703739
H	-4.638015	0.065377	-2.066312
C	-5.319165	1.901514	-0.972389
H	-6.330402	1.645657	-0.674315
C	-4.630798	3.106248	-0.644825
H	-5.027642	3.926471	-0.056137
C	-0.868790	2.217072	1.507390
H	-0.166876	1.835524	2.261971
H	-1.093886	3.266023	1.739337
C	-1.023649	-2.315717	1.447029
C	-0.523456	-3.568727	1.070955
H	-0.324126	-3.782690	0.018820
C	-0.292416	-4.553595	2.025430
H	0.079344	-5.528906	1.716662
C	-0.542997	-4.294669	3.372397
H	-0.362869	-5.065107	4.118933
C	-1.027855	-3.048513	3.756597
H	-1.227369	-2.839277	4.805606
C	-1.270518	-2.064439	2.799204
H	-1.656255	-1.094702	3.114466
C	-2.273216	-1.870559	-1.077246
C	-3.243484	-2.804745	-0.691217
H	-3.349796	-3.083513	0.356442
C	-4.073987	-3.395450	-1.637921
H	-4.822774	-4.118670	-1.321589
C	-3.941433	-3.068894	-2.986262
H	-4.588652	-3.534676	-3.726415
C	-2.969842	-2.155324	-3.382707
H	-2.851431	-1.904179	-4.434785
C	-2.140128	-1.562381	-2.433364
H	-1.375221	-0.852167	-2.745763
C	1.313229	3.393411	0.351095
C	0.990461	4.751335	0.298664
H	-0.007698	5.062087	-0.009524
C	1.947736	5.701607	0.638008
H	1.696282	6.759105	0.596459
C	3.225326	5.298965	1.021133
H	3.974150	6.043529	1.282216
C	3.544306	3.944918	1.066165
H	4.541275	3.626615	1.366691
C	2.590157	2.986272	0.730282
H	2.835798	1.925839	0.761349
H	1.311201	-1.550426	-0.925871
H	2.277611	0.515921	-1.053529
H	1.658133	-0.315554	1.066156
H	0.277805	0.156303	-2.055170

3-K-3MeOH
E (BS1) = -3229.5285520
E (BS2) = -4370.0272006
$G_{298.15,1M}$ (BS2) = -4369.382144

K	4.310208	-1.358835	-0.590305
H	2.014548	-3.398844	-0.667504
O	2.917800	-3.748157	-0.711657
C	2.951639	-4.980478	-0.014303

H	3.963339	-5.387110	-0.106726
H	2.727101	-4.856705	1.056317
H	2.250360	-5.710725	-0.440523
H	5.973184	0.827121	-2.546003
H	2.276534	-0.888966	-2.518612
O	2.988797	-1.131442	-3.143340
O	5.829398	0.589823	-1.621753
C	3.320522	0.036587	-3.868180
H	4.121408	-0.215886	-4.571103
H	2.466749	0.417673	-4.447613
H	3.680291	0.846245	-3.214051
C	5.352786	1.736288	-0.934236
H	5.097536	1.424076	0.085542
H	6.113414	2.527686	-0.879254
H	4.448076	2.151094	-1.404203
O	3.833858	-0.100854	1.718631
C	2.611272	-0.459694	2.108639
C	2.088005	0.311140	3.327709
C	2.457801	-1.964765	2.350296
H	1.047069	0.050201	3.573239
H	2.149282	1.394967	3.162352
H	2.713860	0.071947	4.200773
H	1.443391	-2.243704	2.667107
H	2.705078	-2.540597	1.448992
H	3.157037	-2.264296	3.145663
Fe	-3.669890	1.499390	0.191647
Ir	0.799670	-0.095039	-0.517386
P	-1.265426	-1.025892	0.150183
S	0.019152	2.183714	-0.127725
C	-2.427744	0.098070	0.991645
C	-2.124237	1.408829	1.514492
C	-3.303490	1.900248	2.155177
H	-3.397557	2.880376	2.612822
C	-4.327074	0.919530	2.039984
H	-5.349665	1.020379	2.387629
C	-3.796531	-0.184339	1.321130
H	-4.342749	-1.078450	1.038786
C	-3.337160	3.046539	-1.103993
H	-2.548070	3.783365	-0.991912
C	-3.226105	1.798399	-1.782829
H	-2.338856	1.421457	-2.281669
C	-4.468078	1.108767	-1.647301
H	-4.691133	0.115093	-2.023872
C	-5.346286	1.934466	-0.886625
H	-6.353216	1.677383	-0.575367
C	-4.647420	3.130567	-0.549774
H	-5.030146	3.941274	0.061048
C	-0.847013	2.176948	1.504408
H	-0.130916	1.782431	2.239423
H	-1.053223	3.227218	1.747808
C	-1.058482	-2.367139	1.390541
C	-0.534795	-3.599107	0.976881
H	-0.325571	-3.775953	-0.079978
C	-0.288237	-4.607036	1.902293
H	0.103304	-5.565438	1.566344
C	-0.546134	-4.392055	3.255844
H	-0.352221	-5.180620	3.979601
C	-1.053917	-3.167067	3.676297
H	-1.258055	-2.992986	4.730741
C	-1.312496	-2.159111	2.748384
H	-1.714679	-1.205612	3.090992
C	-2.321210	-1.834710	-1.122182
C	-3.305210	-2.765110	-0.764223
H	-3.414650	-3.074297	0.274614
C	-4.144933	-3.311204	-1.729305
H	-4.905480	-4.031995	-1.436655
C	-4.007600	-2.942111	-3.066120
H	-4.663325	-3.372187	-3.820142
C	-3.021448	-2.032160	-3.434565
H	-2.899905	-1.748960	-4.477972
C	-2.181658	-1.484335	-2.467618
H	-1.406717	-0.777410	-2.760927
C	1.322685	3.344902	0.316573
C	1.041550	4.704602	0.166534
H	0.071060	5.020963	-0.215116
C	2.004206	5.649481	0.506663
H	1.785960	6.708494	0.388123
C	3.242990	5.237843	0.992809
H	3.996078	5.977136	1.256670
C	3.517316	3.881055	1.140057
H	4.483179	3.554973	1.523216
C	2.560094	2.926059	0.801757
H	2.787459	1.866112	0.922153
H	1.297590	-1.593600	-0.889989
H	2.250030	0.480692	-1.070925
H	1.785926	-0.203244	1.280475
H	0.270385	0.065557	-1.993988

4-K-3MeOH

E (BS1) = -3229.5408428
E (BS2) = -4370.0348408
$G_{298.15,1M}$ (BS2) = -4369.387543

H	0.251786	-0.868197	2.538742
K	3.951996	-0.966197	0.273025
H	3.853223	0.727374	-2.203053
H	3.041082	-3.646467	-1.354858
O	3.843726	-3.112939	-1.424918
O	4.755437	0.651704	-1.854650
C	3.878201	-2.543629	-2.724907
H	4.800486	-1.957520	-2.806140
H	3.892525	-3.314488	-3.506694
H	3.023431	-1.874508	-2.906324
C	5.032244	1.829932	-1.118699
H	6.025147	1.715236	-0.671078
H	5.045738	2.722866	-1.758820
H	4.302393	1.998598	-0.308416
O	1.741957	0.000192	1.395920
C	1.361526	-0.771486	2.482799
C	1.812022	-0.108972	3.781845
C	1.921817	-2.196166	2.434541
H	1.551653	-0.710454	4.663892
H	1.355964	0.882977	3.902823
H	2.904954	0.024843	3.771760
H	1.481544	-2.834875	3.212939
H	1.722339	-2.670033	1.462972
H	3.009991	-2.192734	2.613489
O	6.624801	-0.889846	0.478593
C	7.111332	-1.493086	-0.708766
H	7.234896	-0.187825	0.734805
H	6.401839	-2.278894	-0.996027
H	7.189585	-0.776123	-1.538510
H	8.091644	-1.964482	-0.557395
Fe	-3.735393	1.475975	0.381879
Ir	0.718829	-0.094532	-0.625155
P	-1.346344	-1.066861	-0.022899
S	-0.140721	2.181804	-0.134062
C	-2.453713	-0.006559	0.977380

C	-2.104301	1.253822	1.589195
C	-3.234276	1.696804	2.342989
H	-3.287632	2.634600	2.887601
C	-4.277118	0.738319	2.206650
H	-5.274887	0.816787	2.624983
C	-3.806073	-0.301116	1.360449
H	-4.378503	-1.165018	1.038178
C	-3.502534	3.159792	-0.755677
H	-2.719200	3.895523	-0.607255
C	-3.413797	1.995144	-1.571884
H	-2.553320	1.694479	-2.160767
C	-4.636061	1.270442	-1.442947
H	-4.867688	0.321274	-1.916978
C	-5.479002	1.989443	-0.547249
H	-6.461710	1.678946	-0.208236
C	-4.778519	3.155749	-0.121318
H	-5.136040	3.886276	0.596715
C	-0.813449	2.004542	1.561967
H	-0.042573	1.480492	2.141415
H	-0.964732	3.011213	1.971377
C	-1.255454	-2.614821	0.979843
C	-0.666410	-3.735195	0.380075
H	-0.308824	-3.674185	-0.649163
C	-0.535613	-4.929338	1.079724
H	-0.083244	-5.792788	0.596252
C	-0.982744	-5.018461	2.397417
H	-0.879497	-5.951573	2.947297
C	-1.563277	-3.909556	3.004254
H	-1.916896	-3.971116	4.031779
C	-1.702766	-2.714714	2.299048
H	-2.163599	-1.857501	2.787703
C	-2.483242	-1.643019	-1.367141
C	-3.488439	-2.584091	-1.106891
H	-3.575558	-3.032923	-0.117970
C	-4.380272	-2.966292	-2.104023
H	-5.153434	-3.699265	-1.883279
C	-4.277368	-2.420034	-3.381948
H	-4.970907	-2.724176	-4.162995
C	-3.275852	-1.494034	-3.655676
H	-3.180829	-1.069401	-4.653038
C	-2.385058	-1.110748	-2.655284
H	-1.598291	-0.392204	-2.878180
C	1.282141	3.236320	0.149331
C	1.779338	3.894300	-0.979981
H	1.288900	3.763150	-1.944559
C	2.895417	4.714687	-0.871143
H	3.275125	5.224055	-1.754412
C	3.519125	4.889200	0.363005
H	4.389154	5.536423	0.449634
C	3.016598	4.239411	1.485551
H	3.492913	4.375873	2.454228
C	1.900124	3.409133	1.387578
H	1.525852	2.913246	2.278856
H	1.318407	-1.547863	-0.983711
H	2.146821	0.532968	-1.189340
H	0.214505	-0.014454	-2.141382

5-K-3MeOH

E (BS1) = -3230.7179977

E (BS2) = -4371.2142854

$G_{298.15,1M}$ (BS2) = -4370.552576

H	0.970793	0.352661	2.060216
H	1.281988	-0.535377	-1.942463
H	-0.643384	0.492155	-2.387954
K	3.760193	-0.989247	-0.677225
H	5.359692	-3.911969	0.028748
O	5.365136	-2.959825	0.185481
C	5.707302	-2.735891	1.543289
H	5.736796	-1.651848	1.703271
H	4.967969	-3.170351	2.232645
H	6.697689	-3.142559	1.788117
H	2.736878	1.095914	-2.363055
O	3.704914	1.087294	-2.521023
C	4.223545	2.267916	-1.940487
H	5.308790	2.277903	-2.095933
H	3.812040	3.173925	-2.410407
H	4.022043	2.318988	-0.857876
O	2.080087	0.737030	0.354432
C	1.958533	0.759543	1.734054
C	2.080545	2.171565	2.305720
C	3.028520	-0.123889	2.378560
H	1.296615	2.834452	1.917373
H	3.050609	2.603386	2.010679
H	2.023933	2.184188	3.403820
H	2.951961	-0.139918	3.475754
H	2.945666	-1.164473	2.032725
H	4.029732	0.255313	2.117115
O	5.972152	0.392281	0.238174
C	7.013233	-0.022992	-0.629707
H	5.864453	1.347986	0.148627
H	7.227351	-1.074641	-0.409458
H	6.730780	0.057762	-1.691183
H	7.934468	0.552975	-0.474088
H	0.271143	0.240014	4.113339
H	-0.104800	0.278639	4.757274
Fe	-3.969376	-0.161147	0.720689
Ir	0.378513	0.562329	-1.164189
P	-0.743490	-1.222564	-0.115177
S	-1.199569	2.215389	-0.244491
C	-2.064635	-0.793724	1.072067
C	-2.295408	0.496684	1.677625
C	-3.376178	0.352199	2.602000
H	-3.805601	1.162565	3.183447
C	-3.817724	-0.999305	2.580510
H	-4.653214	-1.407478	3.139221
C	-3.021678	-1.703073	1.638570
H	-3.136256	-2.747723	1.369087
C	-4.715753	1.362507	-0.420650
H	-4.361621	2.387730	-0.383735
C	-4.197026	0.327821	-1.252258
H	-3.380938	0.430170	-1.960746
C	-4.900470	-0.876670	-0.950813
H	-4.714409	-1.851877	-1.391125
C	-5.855027	-0.583321	0.066450
H	-6.519137	-1.296868	0.542614
C	-5.740319	0.799529	0.394511
H	-6.301817	1.320139	1.162931
C	-1.571226	1.789744	1.511753
H	-0.618716	1.786321	2.058103
H	-2.193830	2.599521	1.911388
C	0.330204	-2.317698	0.909878
C	1.300697	-3.100028	0.268622
H	1.357949	-3.105761	-0.822299
C	2.171944	-3.895584	1.006808
H	2.914110	-4.506177	0.495227
C	2.091756	-3.912735	2.400119
H	2.769913	-4.537470	2.978096

C	1.134164	-3.136804	3.044555
H	1.062397	-3.145680	4.130662
C	0.255796	-2.347282	2.303713
H	-0.493948	-1.751898	2.823255
C	-1.574116	-2.470587	-1.193684
C	-1.873123	-3.754009	-0.717343
H	-1.564200	-4.051960	0.283956
C	-2.558676	-4.664543	-1.514785
H	-2.784083	-5.656599	-1.128927
C	-2.947209	-4.310426	-2.805213
H	-3.479235	-5.024906	-3.429798
C	-2.639657	-3.045042	-3.295343
H	-2.928100	-2.764717	-4.306349
C	-1.956010	-2.132532	-2.494629
H	-1.711543	-1.145477	-2.883657
C	-0.537049	3.870467	0.014265
C	-1.401948	4.867796	0.471745
H	-2.443314	4.632693	0.691152
C	-0.931447	6.165740	0.637201
H	-1.605112	6.940174	0.997546
C	0.391430	6.475948	0.327543
H	0.753942	7.494241	0.449936
C	1.245142	5.483450	-0.143970
H	2.278664	5.719771	-0.389904
C	0.785389	4.177700	-0.298250
H	1.445225	3.388945	-0.655028
H	1.160272	1.726492	-2.062016

$TS_{5\text{-}6}$-K-3MeOH

E (BS1) = -3230.701675
E (BS2) = -4371.1991823
$G_{298.15,1M}$ (BS2) = -4370.53696

H	2.304265	-0.070042	2.694457
H	1.163902	-0.702039	-1.554249
H	-0.742368	0.285149	-2.177318
K	3.922212	-0.861511	-0.986579
H	4.169945	-4.192027	-0.119939
O	4.572392	-3.315953	-0.167449
C	5.832148	-3.362817	0.482391
H	6.281949	-2.365105	0.411075
H	5.737026	-3.622300	1.546270
H	6.513200	-4.079356	0.003915
H	2.680093	1.377377	-2.359501
O	3.641163	1.386632	-2.542795
C	4.215898	2.389564	-1.721701
H	5.244932	2.557187	-2.058146
H	3.672533	3.342140	-1.804336
H	4.228417	2.094234	-0.659178
O	2.642494	0.670308	0.778562
C	3.064877	0.472819	2.073917
C	3.338989	1.795250	2.792719
C	4.326460	-0.397612	2.133914
H	2.439176	2.426097	2.806122
H	4.126911	2.349264	2.256976
H	3.669086	1.654023	3.832453
H	4.680047	-0.553191	3.163771
H	4.138903	-1.393550	1.704688
H	5.143193	0.079374	1.565480
O	6.564341	-0.183978	-0.926338
C	6.979047	-0.716514	-2.173149
H	6.821197	0.746215	-0.892517
H	6.737602	-1.786568	-2.175661
H	6.469186	-0.236981	-3.022349
H	8.062221	-0.621125	-2.323645
H	0.210471	1.195592	3.328616
H	0.373027	0.907118	2.658406
Fe	-4.248101	0.024793	0.209164
Ir	0.221232	0.484793	-0.974515
P	-0.978106	-1.114811	0.229929
S	-1.278002	2.360229	-0.401406
C	-2.487199	-0.534331	1.071920
C	-2.822656	0.829355	1.416264
C	-4.088683	0.804009	2.081892
H	-4.620445	1.683757	2.431568
C	-4.542592	-0.541041	2.152306
H	-5.491902	-0.875025	2.557065
C	-3.567643	-1.363327	1.528468
H	-3.641960	-2.436175	1.385395
C	-4.639248	1.350654	-1.297407
H	-4.242720	2.359499	-1.347266
C	-4.015603	0.178896	-1.816703
H	-3.059182	0.142897	-2.329646
C	-4.836809	-0.941666	-1.490209
H	-4.617848	-1.981488	-1.714181
C	-5.969749	-0.459134	-0.771967
H	-6.762134	-1.066736	-0.348056
C	-5.846827	0.956165	-0.651359
H	-6.529496	1.611873	-0.121477
C	-2.056787	2.097920	1.255588
H	-1.243497	2.166708	1.987599
H	-2.732108	2.948249	1.414795
C	-0.018544	-1.924413	1.575412
C	1.157099	-2.596558	1.215277
H	1.436921	-2.662238	0.162275
C	1.958036	-3.192446	2.181954
H	2.870255	-3.707536	1.883083
C	1.595732	-3.120272	3.526820
H	2.222414	-3.583852	4.285771
C	0.428204	-2.458954	3.893590
H	0.137145	-2.405995	4.940657
C	-0.377154	-1.863634	2.922951
H	-1.287411	-1.346322	3.224339
C	-1.578003	-2.578371	-0.718118
C	-1.930426	-3.768351	-0.069464
H	-1.805280	-3.859822	1.009208
C	-2.437023	-4.844625	-0.791100
H	-2.709971	-5.761138	-0.272261
C	-2.585208	-4.752162	-2.173480
H	-2.977689	-5.595603	-2.737515
C	-2.215320	-3.582968	-2.831165
H	-2.313860	-3.508014	-3.912147
C	-1.713359	-2.503911	-2.107498
H	-1.422975	-1.593088	-2.628291
C	-0.317837	3.815104	0.052817
C	-0.954514	5.056660	0.043298
H	-2.002936	5.129722	-0.243316
C	-0.242456	6.197610	0.401651
H	-0.737773	7.166014	0.396515
C	1.099993	6.098513	0.759070
H	1.656388	6.992012	1.034046
C	1.729747	4.856659	0.764952
H	2.779079	4.776377	1.045375
C	1.025257	3.706995	0.414711
H	1.518284	2.731193	0.434665
H	1.056905	1.562275	-1.952888

6-K-3MeOH

E (BS1) = -3230.7064495
E (BS2) = -4371,2070275
$G_{298.15,1M}$ (BS2) = -4370.542038

H	2.566841	-0.301194	1.889173
H	1.142472	-0.656578	-1.651885
H	-0.689500	0.336978	-2.400056
K	3.778615	-0.921141	-0.504154
H	5.192325	-2.852323	1.735532
O	5.255151	-2.870813	0.771489
C	6.628037	-2.836039	0.417753
H	6.688673	-2.860425	-0.675314
H	7.126031	-1.922823	0.773679
H	7.170999	-3.707438	0.807860
H	2.425385	0.656965	-2.562968
O	3.369588	0.606784	-2.811643
C	3.907324	1.903877	-2.626643
H	4.958731	1.882567	-2.932484
H	3.389724	2.652636	-3.244052
H	3.861332	2.225692	-1.574526
O	3.462459	1.296125	0.908220
C	3.210151	0.612890	2.067098
C	2.424953	1.442657	3.088058
C	4.487915	0.079756	2.728050
H	1.462200	1.767592	2.666377
H	2.993203	2.349064	3.348527
H	2.218474	0.889624	4.016749
H	4.276835	-0.528134	3.621282
H	5.069012	-0.540843	2.028800
H	5.133736	0.918234	3.030565
O	6.196740	0.151944	-0.980936
C	7.102825	-0.074023	-2.042441
H	6.594977	0.779773	-0.365600
H	6.606741	-0.724921	-2.770626
H	7.384112	0.856977	-2.554145
H	8.024002	-0.572370	-1.706550
H	1.596086	0.855896	0.125630
H	0.916394	0.655582	0.581185
Fe	-4.199713	-0.104575	0.551339
Ir	0.185716	0.512782	-1.065242
P	-0.971594	-1.219480	0.063977
S	-1.386710	2.263136	-0.353611
C	-2.349520	-0.707871	1.130662
C	-2.611675	0.634013	1.594307
C	-3.772648	0.580618	2.425681
H	-4.237360	1.441684	2.896162
C	-4.228033	-0.765135	2.487041
H	-5.115415	-1.115718	3.003012
C	-3.359589	-1.559445	1.691857
H	-3.468290	-2.621938	1.501497
C	-4.816445	1.280745	-0.824379
H	-4.452290	2.301180	-0.880090
C	-4.244293	0.150411	-1.477607
H	-3.366349	0.160604	-2.116443
C	-4.992523	-1.004329	-1.098963
H	-4.784049	-2.027181	-1.398508
C	-6.027699	-0.584428	-0.213465
H	-6.742813	-1.231387	0.283359
C	-5.917898	0.826616	-0.042738
H	-6.536425	1.440110	0.603561
C	-1.821880	1.882857	1.401941
H	-0.873017	1.844389	1.956999
H	-2.400947	2.741539	1.764141
C	0.165712	-2.062687	1.235767
C	1.155141	-2.916358	0.729743
H	1.164391	-3.170866	-0.331695
C	2.118732	-3.457093	1.575314
H	2.878967	-4.123896	1.172654
C	2.114444	-3.139243	2.934271
H	2.872143	-3.557157	3.594326
C	1.135704	-2.291220	3.441997
H	1.125004	-2.042103	4.501317
C	0.164015	-1.754811	2.597591
H	-0.591506	-1.083924	3.007773
C	-1.666148	-2.605581	-0.915541
C	-1.968930	-3.833796	-0.313785
H	-1.738918	-4.000245	0.738117
C	-2.556655	-4.853780	-1.054242
H	-2.788451	-5.802848	-0.575771
C	-2.839414	-4.662922	-2.405527
H	-3.296271	-5.463029	-2.983860
C	-2.524794	-3.452560	-3.015694
H	-2.732256	-3.302175	-4.072921
C	-1.938649	-2.429219	-2.274378
H	-1.689473	-1.483704	-2.754294
C	-0.561303	3.833550	-0.039086
C	-1.351777	4.983134	-0.080867
H	-2.413331	4.904570	-0.312805
C	-0.777069	6.224705	0.173277
H	-1.391816	7.121224	0.136811
C	0.580277	6.316034	0.469651
H	1.029703	7.287018	0.665799
C	1.363193	5.165643	0.513884
H	2.423803	5.233484	0.747154
C	0.798735	3.918875	0.258159
H	1.421133	3.026104	0.292388
H	1.002257	1.634788	-1.977451

TS_{6-7}-K-3MeOH
E (BS1) = -3230.7058804
E (BS2) = -4371.2050929
$G_{298.15,1M}$ (BS2) = -4370.539962

H	2.619569	-0.299783	1.993683
H	1.142464	-0.691660	-1.623811
H	-0.692629	0.300821	-2.395646
K	3.735257	-1.012559	-0.544099
H	5.195351	-2.791480	1.802219
O	5.216614	-2.864531	0.839226
C	6.572999	-2.879434	0.426631
H	6.585195	-2.947861	-0.666394
H	7.108567	-1.966966	0.724852
H	7.111069	-3.749349	0.826851
H	2.406073	0.585200	-2.571342
O	3.343825	0.527910	-2.845138
C	3.881268	1.831447	-2.719025
H	4.937030	1.794981	-3.008544
H	3.373558	2.549209	-3.380090
H	3.821171	2.206198	-1.685614
O	3.277673	1.230318	0.763081
C	3.216680	0.655497	2.014363
C	2.509722	1.551264	3.033056
C	4.598217	0.267332	2.544906
H	1.476279	1.758419	2.719676
H	3.034508	2.516711	3.104578
H	2.474752	1.103469	4.037337
H	4.531676	-0.250644	3.513698
H	5.125978	-0.399371	1.847496

H	5.220855	1.164902	2.676901
O	6.144851	0.102349	-0.989183
C	6.943098	-0.205658	-2.114699
H	6.641238	0.697660	-0.414471
H	6.343667	-0.831108	-2.786200
H	7.246403	0.694177	-2.667945
H	7.851144	-0.763943	-1.843324
H	1.724944	0.862678	0.262632
H	0.948762	0.657125	0.563726
Fe	-4.203891	-0.062851	0.495660
Ir	0.180536	0.487775	-1.059573
P	-0.976423	-1.209156	0.104226
S	-1.351318	2.273320	-0.365139
C	-2.376063	-0.675485	1.134363
C	-2.636506	0.675563	1.570963
C	-3.820790	0.647510	2.370346
H	-4.288056	1.520533	2.815646
C	-4.292180	-0.692341	2.439246
H	-5.197220	-1.026084	2.935377
C	-3.410052	-1.507663	1.680717
H	-3.527216	-2.571077	1.501579
C	-4.761254	1.301014	-0.926160
H	-4.378905	2.314041	-0.993674
C	-4.188488	0.148475	-1.538714
H	-3.292303	0.132713	-2.151636
C	-4.964078	-0.986796	-1.156506
H	-4.761783	-2.018963	-1.427280
C	-6.017059	-0.532629	-0.309576
H	-6.755514	-1.158079	0.180623
C	-5.890624	0.880156	-0.165990
H	-6.517573	1.516340	0.449530
C	-1.823917	1.910684	1.384541
H	-0.885043	1.859341	1.955117
H	-2.394237	2.780741	1.733066
C	0.142123	-2.011074	1.324426
C	1.131517	-2.892272	0.868523
H	1.147705	-3.198613	-0.179275
C	2.086566	-3.395684	1.746727
H	2.845917	-4.085101	1.382613
C	2.074034	-3.013151	3.088790
H	2.824800	-3.402234	3.773824
C	1.092014	-2.141586	3.548366
H	1.071873	-1.844165	4.594980
C	0.129329	-1.642857	2.671402
H	-0.628463	-0.952589	3.043478
C	-1.653491	-2.635355	-0.831855
C	-1.971128	-3.838458	-0.188535
H	-1.765086	-3.962327	0.874142
C	-2.543478	-4.887471	-0.899839
H	-2.787105	-5.816332	-0.388567
C	-2.795985	-4.751377	-2.263701
H	-3.241028	-5.573999	-2.819283
C	-2.465965	-3.566817	-2.915043
H	-2.649523	-3.459587	-3.981969
C	-1.895304	-2.514426	-2.202563
H	-1.633850	-1.588905	-2.714285
C	-0.494189	3.824228	-0.038086
C	-1.256160	4.992452	-0.093540
H	-2.314943	4.939346	-0.344638
C	-0.657334	6.220150	0.172254
H	-1.249950	7.131012	0.125110
C	0.695926	6.279332	0.494369
H	1.164347	7.239291	0.700407
C	1.450321	5.110427	0.551577
H	2.507842	5.152547	0.805335
C	0.862030	3.877220	0.283168
H	1.463047	2.969999	0.327542
H	1.017555	1.599380	-1.964690

7-K-3MeOH

E (BS1) = -3230.7453398

E (BS2) = -4371.2385163

$G_{298.15,1M}$ (BS2) = -4370.565439

H	2.740047	-0.981530	1.785665
H	0.969409	-0.512538	-2.134821
H	-0.976798	0.611131	-2.619219
K	3.594120	-1.136385	-1.055318
H	4.841046	-3.275385	1.084161
O	5.083622	-2.994717	0.192173
C	6.498302	-2.996204	0.097676
H	6.766673	-2.674708	-0.913671
H	6.962402	-2.306098	0.816980
H	6.914100	-4.000178	0.257378
H	2.434708	1.147463	-2.370979
O	3.403091	1.161074	-2.537744
C	3.890901	2.378932	-2.011416
H	4.946399	2.476150	-2.287760
H	3.351484	3.246994	-2.418667
H	3.824197	2.420651	-0.911853
O	3.573553	0.785813	1.087340
C	3.313976	-0.116750	2.170766
C	2.488884	0.570373	3.236334
C	4.648069	-0.602164	2.686044
H	1.523319	0.902982	2.830697
H	3.023644	1.449065	3.620640
H	2.289049	-0.106235	4.076646
H	4.500759	-1.345598	3.478998
H	5.234579	-1.065824	1.883122
H	5.226493	0.234555	3.101244
O	5.851369	0.228857	-0.410392
C	7.168621	0.670641	-0.166325
H	5.249555	0.678816	0.209702
H	7.842239	0.120516	-0.831727
H	7.291554	1.744278	-0.372541
H	7.484762	0.482491	0.870947
H	2.712183	0.997533	0.669936
H	1.299729	0.622133	-0.100937
Fe	-4.019887	0.022300	1.002432
Ir	0.090190	0.585689	-1.318543
P	-0.964032	-1.167221	-0.184733
S	-1.284098	2.302926	-0.240753
C	-2.119462	-0.695997	1.149631
C	-2.223185	0.598385	1.778798
C	-3.207774	0.500904	2.810145
H	-3.532974	1.327030	3.435314
C	-3.710854	-0.829068	2.834733
H	-4.501108	-1.201631	3.477812
C	-3.049982	-1.564365	1.814817
H	-3.243508	-2.600064	1.556148
C	-4.809479	1.565386	-0.085871
H	-4.403265	2.571029	-0.115287
C	-4.441165	0.489323	-0.944673
H	-3.705409	0.533909	-1.741589
C	-5.167048	-0.669631	-0.537891
H	-5.079964	-1.661722	-0.970836
C	-5.985925	-0.306632	0.571156
H	-6.628619	-0.974340	1.135079

C	-5.763790	1.073607	0.851298
H	-6.210187	1.638496	1.662686
C	-1.446297	1.847128	1.537512
H	-0.424234	1.765384	1.934070
H	-1.946440	2.689028	2.033326
C	0.202540	-2.266901	0.728916
C	1.028337	-3.131038	-0.003133
H	0.911799	-3.204341	-1.086312
C	1.977941	-3.919140	0.640089
H	2.604207	-4.594328	0.059522
C	2.119464	-3.851545	2.027581
H	2.859059	-4.470853	2.531675
C	1.300526	-3.000488	2.761728
H	1.399565	-2.947646	3.844515
C	0.347054	-2.211809	2.116731
H	-0.283194	-1.546280	2.706939
C	-1.952565	-2.411502	-1.127892
C	-2.238756	-3.678187	-0.601934
H	-1.828781	-3.976605	0.362461
C	-3.040057	-4.571885	-1.305330
H	-3.254289	-5.551652	-0.883363
C	-3.558912	-4.217155	-2.549391
H	-4.181814	-4.918845	-3.100162
C	-3.265301	-2.968910	-3.089253
H	-3.655368	-2.688951	-4.065676
C	-2.464655	-2.074016	-2.382802
H	-2.227666	-1.099070	-2.808251
C	-0.385715	3.845056	0.005079
C	-1.107851	5.034035	-0.095336
H	-2.174651	5.004609	-0.313756
C	-0.457512	6.253099	0.080525
H	-1.018716	7.181060	-0.004296
C	0.905148	6.281777	0.361687
H	1.412855	7.234550	0.495313
C	1.620703	5.090827	0.470522
H	2.686685	5.110733	0.689626
C	0.979038	3.869881	0.290558
H	1.532706	2.934924	0.359772
H	0.879591	1.789827	-2.157522

Isopropanol solvent

Me_2CO
E (BS1) = -193.0353908
E (BS2) = -193.110373
$G_{298.15,1M}$ (BS2) = -193.053438

O	-0.000001	1.402679	0.000000
C	0.000000	0.182028	-0.000000
C	-1.271959	-0.611983	0.000656
C	1.271960	-0.611982	-0.000656
H	-1.319987	-1.238427	-0.898996
H	-2.141637	0.049272	0.034567
H	-1.290419	-1.295751	0.858347
H	1.290420	-1.295749	-0.858347
H	2.141637	0.049275	-0.034567
H	1.319988	-1.238425	0.898997

D. 5iPrOH model

D1. Cation-free system

1-iso
E (BS1) = -2478.13045788
E (BS2) = -3618.531551
$G_{298.15,1M}$ (BS2) = -3617.905012

H	-2.677574	1.596714	-2.064227
O	-3.530116	2.055119	-2.198975
C	-3.758042	2.876964	-1.063732
H	-3.411524	2.345344	-0.156041
Fe	3.282931	-1.687833	0.543422
Ir	-0.630196	0.192863	-1.142320
P	1.119715	0.998278	0.170801
S	-0.317084	-2.145162	-0.403354
C	2.032979	-0.216483	1.182885
C	1.532450	-1.507892	1.580545
C	2.531411	-2.131370	2.389905
H	2.453917	-3.128405	2.813037
C	3.638163	-1.245433	2.505686
H	4.570774	-1.451562	3.020253
C	3.336276	-0.069850	1.765595
H	4.002130	0.774366	1.621708
C	3.086974	-3.112383	-0.913458
H	2.243406	-3.787526	-1.007206
C	3.185580	-1.814697	-1.494530
H	2.421577	-1.326558	-2.093515
C	4.426218	-1.241277	-1.084073
H	4.777946	-0.241996	-1.324146
C	5.092931	-2.187189	-0.250986
H	6.041295	-2.035437	0.253629
C	4.264262	-3.342516	-0.143983
H	4.475181	-4.224677	0.451094
C	0.182188	-2.102686	1.358753
H	-0.580858	-1.526600	1.902607
H	0.181157	-3.135497	1.728486
C	0.556421	2.184133	1.464465
C	0.108819	3.450128	1.069329
H	0.183864	3.748222	0.022491
C	-0.434237	4.330716	1.997567
H	-0.772960	5.313725	1.675509
C	-0.545855	3.954724	3.335601
H	-0.973984	4.642084	4.062217
C	-0.107602	2.697092	3.736495
H	-0.189135	2.397362	4.779560
C	0.443340	1.816127	2.806782
H	0.780567	0.832915	3.135617
C	2.494438	1.943699	-0.618899
C	3.331477	2.786249	0.123776
H	3.148086	2.938150	1.187279
C	4.397163	3.440245	-0.485803
H	5.041026	4.090025	0.103562
C	4.633203	3.271264	-1.849042
H	5.464227	3.786906	-2.325944
C	3.794694	2.453334	-2.599935
H	3.964922	2.327860	-3.667356
C	2.730224	1.795171	-1.987226
H	2.069064	1.155222	-2.571253
C	-1.904849	-2.976892	-0.283542
C	-2.528200	-3.275722	-1.499354
H	-2.053344	-2.991078	-2.437674
C	-3.755332	-3.924531	-1.511246
H	-4.232796	-4.148008	-2.462873
C	-4.370693	-4.289098	-0.314351
H	-5.332307	-4.797072	-0.325138

C	-3.742465	-4.003903	0.891544
H	-4.210347	-4.284741	1.833636
C	-2.511130	-3.348191	0.914949
H	-2.048645	-3.134554	1.874560
H	-0.803733	1.687108	-1.726939
H	-1.922017	-0.255616	-2.085944
H	-1.694005	0.579525	0.132592
H	0.322802	-0.146167	-2.498375
O	-4.185714	-0.416649	0.040996
C	-4.695771	0.077022	1.269295
H	-4.717644	1.185247	1.242478
H	-3.252420	-0.132483	-0.045810
C	-5.249899	3.103518	-0.959515
H	-5.618960	3.616312	-1.858604
H	-5.781175	2.147845	-0.870949
H	-5.496098	3.722149	-0.088028
C	-2.993867	4.181925	-1.187365
H	-3.157880	4.824744	-0.312681
H	-1.915969	3.994405	-1.279894
H	-3.323239	4.728246	-2.082131
C	-3.839624	-0.355323	2.446437
H	-4.238052	0.044623	3.387636
H	-3.816522	-1.452082	2.518538
H	-2.807749	0.004792	2.334807
C	-6.114285	-0.433242	1.393665
H	-6.724666	-0.111930	0.541188
H	-6.114068	-1.532123	1.416417
H	-6.585270	-0.067697	2.313559

```

**2-iso**

E (BS1) = -2671.17618164

E (BS2) = -3811.647398

$G_{298.15,1M}$ (BS2) = -3810.947705

| | | | |
|---|---|---|---|
| H | 3.176222 | -2.354428 | -0.176463 |
| O | 3.211495 | -3.169931 | -0.703138 |
| C | 4.481653 | -3.179985 | -1.341977 |
| H | 5.275126 | -3.099206 | -0.574376 |
| Fe | -3.936770 | 1.428109 | 0.260748 |
| Ir | 0.404029 | -0.106920 | -0.906401 |
| P | -1.462598 | -1.031113 | 0.130162 |
| S | -0.312634 | 2.211718 | -0.444485 |
| C | -2.587835 | 0.103435 | 1.023031 |
| C | -2.265615 | 1.446961 | 1.433014 |
| C | -3.389098 | 1.963944 | 2.150224 |
| H | -3.457590 | 2.969395 | 2.554873 |
| C | -4.398287 | 0.962311 | 2.195638 |
| H | -5.385956 | 1.069102 | 2.631776 |
| C | -3.911406 | -0.178335 | 1.501566 |
| H | -4.464876 | -1.094393 | 1.323950 |
| C | -3.770619 | 2.876579 | -1.174140 |
| H | -2.998782 | 3.639032 | -1.190865 |
| C | -3.676733 | 1.581121 | -1.760839 |
| H | -2.818941 | 1.186757 | -2.297224 |
| C | -4.879097 | 0.873152 | -1.461655 |
| H | -5.098889 | -0.154854 | -1.734326 |
| C | -5.714889 | 1.733601 | -0.691115 |
| H | -6.682220 | 1.476057 | -0.272775 |
| C | -5.029249 | 2.970939 | -0.512281 |
| H | -5.385287 | 3.819278 | 0.062607 |
| C | -0.995746 | 2.209455 | 1.271934 |
| H | -0.203516 | 1.800921 | 1.917292 |
| H | -1.168008 | 3.257255 | 1.548722 |
| C | -1.035955 | -2.221069 | 1.476355 |
| C | -0.497195 | -3.466734 | 1.132114 |
| H | -0.377564 | -3.734008 | 0.081288 |
| C | -0.113229 | -4.369491 | 2.117442 |
| H | 0.297802 | -5.335987 | 1.832361 |
| C | -0.248816 | -4.035818 | 3.464449 |
| H | 0.051688 | -4.742109 | 4.235638 |
| C | -0.764984 | -2.792860 | 3.816588 |
| H | -0.869970 | -2.521736 | 4.865451 |
| C | -1.158379 | -1.890564 | 2.828406 |
| H | -1.564051 | -0.921454 | 3.120032 |
| C | -2.667691 | -2.039989 | -0.848189 |
| C | -3.536024 | -2.953191 | -0.235766 |
| H | -3.470528 | -3.134868 | 0.836692 |
| C | -4.482600 | -3.643949 | -0.985640 |
| H | -5.150616 | -4.348995 | -0.494746 |
| C | -4.567858 | -3.441630 | -2.362000 |
| H | -5.305201 | -3.986065 | -2.948498 |
| C | -3.695795 | -2.553610 | -2.983915 |
| H | -3.745049 | -2.401285 | -4.060320 |
| C | -2.751316 | -1.859556 | -2.230356 |
| H | -2.059929 | -1.170019 | -2.714651 |
| C | 1.036082 | 3.362769 | -0.112158 |
| C | 0.724558 | 4.720637 | -0.002509 |
| H | -0.307690 | 5.052669 | -0.115520 |
| C | 1.734542 | 5.643811 | 0.245269 |
| H | 1.490116 | 6.700485 | 0.331751 |
| C | 3.056281 | 5.217537 | 0.366921 |
| H | 3.846390 | 5.942971 | 0.549207 |
| C | 3.363503 | 3.866403 | 0.244792 |
| H | 4.392755 | 3.517796 | 0.319159 |
| C | 2.354516 | 2.934245 | 0.010825 |
| H | 2.587224 | 1.875169 | -0.088981 |
| H | 0.875750 | -1.602914 | -1.263987 |
| H | 1.820548 | 0.341925 | -1.646696 |
| H | 1.290894 | -0.217247 | 0.537110 |
| H | -0.293058 | -0.021640 | -2.453919 |
| O | 4.962452 | 1.064977 | -0.542965 |
| C | 6.348887 | 0.774194 | -0.427759 |
| H | 6.506306 | -0.312884 | -0.568572 |
| H | 4.502173 | 0.432355 | 0.040986 |
| C | 3.411652 | -0.888253 | 2.066299 |
| O | 4.089440 | -1.044453 | 1.051866 |
| C | 3.312732 | 0.437660 | 2.746467 |
| C | 2.665618 | -2.025063 | 2.676384 |
| H | 3.694465 | 1.239404 | 2.105978 |
| H | 2.962812 | -2.974451 | 2.222438 |
| H | 3.904056 | 0.402341 | 3.671996 |
| H | 2.275282 | 0.644243 | 3.037684 |
| H | 1.594021 | -1.857472 | 2.493736 |
| H | 2.805429 | -2.051867 | 3.763794 |
| C | 4.615768 | -4.511180 | -2.045610 |
| H | 5.592985 | -4.598169 | -2.534533 |
| H | 4.507419 | -5.342124 | -1.338882 |
| H | 3.837810 | -4.610685 | -2.814342 |
| C | 4.613035 | -2.013807 | -2.302682 |
| H | 5.603418 | -2.001055 | -2.777870 |
| H | 3.853530 | -2.088253 | -3.093211 |
| H | 4.471612 | -1.055057 | -1.785315 |
| C | 6.866534 | 1.165683 | 0.943466 |
| H | 6.337253 | 0.617271 | 1.733164 |
| H | 7.937786 | 0.949007 | 1.041873 |
| H | 6.717499 | 2.241560 | 1.112499 |
| C | 7.052391 | 1.519027 | -1.538470 |
```

H	8.133416	1.339905	-1.513037
H	6.670422	1.205269	-2.517466
H	6.879266	2.599255	-1.433555

$TS_{2\text{-}3}$-iso
E (BS1) = -2671.16478397
E (BS2) = -3811.635879
$G_{298.15,1M}$ (BS2) = -3810.931473

H	3.698266	-1.614241	-1.174274
O	3.835995	-2.359699	-1.798003
C	5.077166	-2.942307	-1.433954
H	5.082270	-3.142607	-0.344061
Fe	-3.874409	1.582528	0.421890
Ir	0.210619	-0.328453	-1.070664
P	-1.620542	-1.066079	0.182802
S	-0.290289	2.037558	-0.655587
C	-2.558973	0.192052	1.122001
C	-2.103020	1.524204	1.430900
C	-3.111436	2.156042	2.222966
H	-3.067764	3.180537	2.580410
C	-4.180752	1.237612	2.413121
H	-5.111155	1.438567	2.933712
C	-3.847132	0.033935	1.735837
H	-4.480169	-0.843529	1.659292
C	-3.739086	2.952904	-1.091208
H	-2.916415	3.648976	-1.218429
C	-3.804550	1.631834	-1.621936
H	-3.039435	1.149088	-2.222357
C	-5.022445	1.036719	-1.176570
H	-5.347301	0.020583	-1.380489
C	-5.708928	1.992509	-0.371889
H	-6.647171	1.831607	0.148518
C	-4.915317	3.175908	-0.318101
H	-5.146134	4.072501	0.247350
C	-0.806194	2.184315	1.112074
H	0.012559	1.763398	1.713500
H	-0.884664	3.256515	1.331790
C	-1.162867	-2.239279	1.527721
C	-0.839567	-3.562123	1.204361
H	-0.927171	-3.905572	0.173118
C	-0.407506	-4.445083	2.187959
H	-0.167371	-5.472751	1.922682
C	-0.277777	-4.014313	3.507382
H	0.061875	-4.704828	4.276476
C	-0.581447	-2.696538	3.835580
H	-0.481237	-2.351957	4.863013
C	-1.024447	-1.813936	2.851661
H	-1.261836	-0.784517	3.122669
C	-2.980846	-1.994915	-0.657303
C	-3.874613	-2.801826	0.059302
H	-3.740332	-2.949014	1.130432
C	-4.934064	-3.432176	-0.585051
H	-5.619976	-4.054318	-0.013558
C	-5.110368	-3.275539	-1.958669
H	-5.936758	-3.772542	-2.462607
C	-4.216305	-2.494331	-2.683904
H	-4.337360	-2.378922	-3.759145
C	-3.157694	-1.860936	-2.036179
H	-2.452107	-1.255483	-2.603947
C	1.150683	3.114108	-0.534028
C	0.919705	4.491374	-0.487909
H	-0.100313	4.873181	-0.533823
C	1.992727	5.369737	-0.386855
H	1.811015	6.441811	-0.350478
C	3.294912	4.875907	-0.344089
H	4.134503	5.564029	-0.270457
C	3.522859	3.504183	-0.402564
H	4.536674	3.105842	-0.375320
C	2.451162	2.616613	-0.498106
H	2.622350	1.540761	-0.547073
H	0.530483	-1.863880	-1.463586
H	1.525638	0.027412	-2.014443
H	1.350081	-0.456238	0.293376
H	-0.675218	-0.217354	-2.455449
O	5.851004	1.150518	-0.171239
C	6.485109	0.890397	1.073580
H	6.234629	-0.139148	1.394512
H	4.957492	0.755727	-0.098140
C	2.809987	-0.784137	1.048271
O	3.726890	-0.502182	0.226466
C	2.572401	0.148420	2.209818
C	2.515827	-2.237371	1.320702
H	2.596659	1.195427	1.886649
H	2.515502	-2.815517	0.390999
H	3.395305	-0.007361	2.926112
H	1.629366	-0.061443	2.726941
H	1.567870	-2.374790	1.848741
H	3.326797	-2.615303	1.963700
C	5.198529	-4.256242	-2.172937
H	6.137972	-4.763934	-1.924428
H	4.366368	-4.924866	-1.923348
H	5.180740	-4.078846	-3.256867
C	6.226635	-2.004551	-1.756198
H	7.188504	-2.428110	-1.438138
H	6.272324	-1.825394	-2.839424
H	6.096659	-1.033926	-1.258419
C	5.998133	1.866873	2.127712
H	4.903880	1.831344	2.212709
H	6.426496	1.643185	3.113052
H	6.283707	2.892130	1.853376
C	7.978245	0.988460	0.856734
H	8.525313	0.809302	1.789717
H	8.315447	0.257821	0.111427
H	8.239254	1.991944	0.494134

3-iso
E (BS1) = -2671.17797359
E (BS2) = -3811.651214
$G_{298.15,1M}$ (BS2) = -3810.944088

H	3.736272	-1.363252	-1.224056
O	3.846796	-2.080433	-1.920695
C	5.104615	-2.667349	-1.663446
H	5.184776	-2.916909	-0.584886
Fe	-3.935656	1.509810	0.437073
Ir	0.136573	-0.312443	-1.144675
P	-1.618239	-1.076755	0.235067
S	-0.394447	2.070904	-0.712605
C	-2.575689	0.175105	1.154805
C	-2.150785	1.526457	1.422038
C	-3.164263	2.151299	2.212571
H	-3.142753	3.185112	2.543888
C	-4.205626	1.210008	2.441313
H	-5.133973	1.400305	2.969382
C	-3.850297	-0.002309	1.790801
H	-4.461267	-0.897424	1.744118
C	-3.868604	2.849357	-1.108237

H	-3.072987	3.570779	-1.262809
C	-3.895950	1.514813	-1.607683
H	-3.124307	1.045303	-2.210044
C	-5.086014	0.888544	-1.130299
H	-5.378961	-0.142571	-1.304933
C	-5.793720	1.839142	-0.338095
H	-6.717862	1.658292	0.200568
C	-5.040835	3.049750	-0.323212
H	-5.293667	3.950530	0.225869
C	-0.873056	2.204893	1.070003
H	-0.030791	1.798406	1.649465
H	-0.958031	3.276617	1.289101
C	-1.035953	-2.166195	1.596985
C	-0.678916	-3.489460	1.312384
H	-0.811816	-3.883385	0.304308
C	-0.160242	-4.309323	2.308130
H	0.103976	-5.339003	2.076133
C	0.026522	-3.812017	3.596644
H	0.434500	-4.453351	4.374968
C	-0.306434	-2.491329	3.883165
H	-0.160488	-2.096036	4.886462
C	-0.837871	-1.671684	2.889056
H	-1.100937	-0.640404	3.126918
C	-2.946545	-2.087613	-0.542885
C	-3.785983	-2.909195	0.220129
H	-3.619079	-3.016175	1.291453
C	-4.831506	-3.603386	-0.379592
H	-5.476464	-4.236600	0.226123
C	-5.046680	-3.494904	-1.752205
H	-5.862785	-4.040845	-2.220782
C	-4.205826	-2.698250	-2.522878
H	-4.358445	-2.619697	-3.597239
C	-3.160779	-2.000881	-1.920978
H	-2.502026	-1.382268	-2.528348
C	1.057564	3.131711	-0.596596
C	0.849794	4.512867	-0.613823
H	-0.157651	4.910275	-0.737283
C	1.932252	5.373957	-0.467369
H	1.771446	6.449854	-0.481553
C	3.215911	4.857686	-0.301777
H	4.060318	5.532991	-0.180188
C	3.418610	3.480680	-0.294231
H	4.416481	3.062028	-0.158690
C	2.340768	2.609751	-0.451086
H	2.518866	1.532588	-0.454155
H	0.434883	-1.837252	-1.585172
H	1.343422	0.083615	-2.202225
H	1.685608	-0.410186	0.433427
H	-0.815281	-0.225343	-2.376661
O	5.844283	1.169110	0.092332
C	6.515105	0.685334	1.240164
H	6.129130	-0.327843	1.473064
H	4.954883	0.712235	0.085737
C	2.725064	-0.642368	0.880325
O	3.713073	-0.325953	0.005546
C	2.780375	0.186242	2.162114
C	2.702658	-2.130636	1.221445
H	2.802348	1.260656	1.932390
H	2.598923	-2.734934	0.311055
H	3.700137	-0.059987	2.715140
H	1.924393	-0.013772	2.823829
H	1.890024	-2.392367	1.913178
H	3.659231	-2.395820	1.698880
C	5.201122	-3.947757	-2.464773
H	6.156412	-4.457658	-2.290512
H	4.388513	-4.635534	-2.201668
H	5.123049	-3.724591	-3.537923
C	6.228333	-1.704113	-2.007015
H	7.213045	-2.140066	-1.790144
H	6.192815	-1.452953	-3.076545
H	6.128638	-0.771645	-1.434843
C	6.256095	1.599779	2.425145
H	5.179235	1.705975	2.607758
H	6.726252	1.222882	3.342750
H	6.662402	2.599954	2.217737
C	7.995647	0.589499	0.934192
H	8.561724	0.242178	1.807190
H	8.185117	-0.101049	0.103554
H	8.382026	1.577840	0.649109

4-iso

E (BS1) = -2671.19344042

E (BS2) = -3811.667033

$G_{298.15,1M}$ (BS2) = -3810.956016

H	0.886844	-0.202007	2.157639
H	0.952060	-1.499005	-1.656926
H	-0.313664	0.197423	-2.266549
H	2.649905	-2.096324	0.161407
O	2.959492	-3.008715	-0.073534
C	3.583251	-2.977103	-1.346176
H	3.532112	-1.949558	-1.751437
O	2.198757	-0.495477	0.590838
C	1.977506	-0.281364	1.955680
C	2.625219	1.010433	2.447790
C	2.503215	-1.453268	2.778432
H	2.276936	1.879955	1.876605
H	3.717070	0.950179	2.333606
H	2.403886	1.189883	3.509717
H	2.321940	-1.304863	3.852372
H	2.023806	-2.393026	2.477579
H	3.586901	-1.562543	2.628011
O	4.909149	-0.426850	0.510232
C	5.440307	0.731549	-0.099297
H	3.919331	-0.406924	0.436076
H	4.932235	1.629498	0.307458
Fe	-3.669167	1.447928	0.327942
Ir	0.618156	-0.069886	-1.000193
P	-1.210017	-1.027665	0.143993
S	-0.061309	2.216685	-0.338799
C	-2.321183	0.103198	1.058714
C	-2.004059	1.436316	1.510477
C	-3.133624	1.928614	2.235408
H	-3.207236	2.922479	2.666799
C	-4.140973	0.924776	2.245826
H	-5.131299	1.017229	2.679021
C	-3.648781	-0.192770	1.520177
H	-4.198410	-1.105156	1.314430
C	-3.494931	2.936952	-1.063274
H	-2.718044	3.694565	-1.056131
C	-3.409839	1.659354	-1.689010
H	-2.554733	1.276469	-2.237891
C	-4.615961	0.949600	-1.410457
H	-4.841818	-0.068489	-1.713820
C	-5.445843	1.791539	-0.613671
H	-6.414173	1.527081	-0.202066
C	-4.752726	3.018874	-0.398321
H	-5.103208	3.851297	0.202609

C	-0.744210	2.223520	1.374580
H	0.037848	1.844104	2.045714
H	-0.949974	3.267603	1.638693
C	-0.825262	-2.279028	1.443586
C	-0.206503	-3.467821	1.041382
H	0.011582	-3.633295	-0.014207
C	0.125051	-4.445491	1.972816
H	0.603286	-5.365330	1.642067
C	-0.148605	-4.243173	3.324360
H	0.111617	-5.006621	4.054590
C	-0.753098	-3.059338	3.735889
H	-0.968631	-2.893455	4.789688
C	-1.092072	-2.082710	2.801010
H	-1.568312	-1.162379	3.138230
C	-2.418813	-1.987364	-0.879244
C	-3.285381	-2.921039	-0.295605
H	-3.218025	-3.137480	0.770064
C	-4.232446	-3.590149	-1.064153
H	-4.897656	-4.310824	-0.592709
C	-4.320797	-3.347265	-2.433276
H	-5.057620	-3.875266	-3.035072
C	-3.451502	-2.438716	-3.028354
H	-3.501924	-2.253393	-4.099421
C	-2.507248	-1.765115	-2.256147
H	-1.824701	-1.060041	-2.726773
C	1.247318	3.435331	-0.136082
C	0.906756	4.745458	0.210595
H	-0.133892	5.012675	0.392666
C	1.899391	5.714018	0.309641
H	1.632100	6.731699	0.586243
C	3.226648	5.387571	0.038086
H	3.999894	6.149493	0.108047
C	3.559016	4.087124	-0.328263
H	4.594244	3.826059	-0.544616
C	2.573024	3.106326	-0.410729
H	2.821270	2.079661	-0.679156
H	1.850294	0.467180	-1.968020
C	5.042017	-3.373648	-1.204381
H	5.558569	-2.681828	-0.528576
H	5.117821	-4.388422	-0.788449
H	5.557342	-3.363627	-2.173998
C	2.848019	-3.909070	-2.291830
H	3.324903	-3.937341	-3.280110
H	2.846896	-4.930413	-1.884841
H	1.805059	-3.592424	-2.421569
C	6.907909	0.804609	0.263247
H	7.041451	0.833571	1.351199
H	7.432561	-0.081440	-0.121555
H	7.378839	1.696317	-0.167820
C	5.239269	0.700874	-1.603876
H	5.616216	1.616811	-2.078939
H	5.776250	-0.155147	-2.038211
H	4.174250	0.597204	-1.856005

D2. Lithium system

1-Li-iso

E (BS1) = -3068.41398394

E (BS2) = -4209.024348

$G_{298.15,1M}$ (BS2) = -4208.087888

Li	-3.615260	0.789581	0.084573
H	-2.402836	2.318247	1.249765
O	-3.148516	1.986622	1.784744
C	-2.691733	1.669311	3.103244
H	-2.177095	2.556942	3.510378
H	-2.220236	-0.470538	-1.777307
H	-1.353247	1.630408	-0.903241
O	-2.084863	2.232160	-0.637450
O	-3.166591	-0.316489	-1.569895
C	-2.175721	3.331549	-1.546755
H	-1.172211	3.781066	-1.646141
C	-3.991027	-1.205385	-2.319815
H	-4.974595	-1.165264	-1.821967
Fe	4.497592	-1.140899	1.113607
Ir	0.517018	-0.174422	-1.328586
P	2.089322	1.070925	-0.125838
S	1.225326	-2.287588	-0.270687
C	2.965928	0.200118	1.221812
C	2.580853	-1.056724	1.814246
C	3.492616	-1.333554	2.878723
H	3.478071	-2.230609	3.490513
C	4.432027	-0.268277	2.959467
H	5.276332	-0.211426	3.638295
C	4.115090	0.671113	1.941760
H	4.673375	1.574078	1.717956
C	4.746457	-2.837278	-0.005261
H	4.016539	-3.635335	-0.086913
C	4.815834	-1.679857	-0.834023
H	4.147746	-1.443412	-1.656179
C	5.882408	-0.859511	-0.359743
H	6.165383	0.110740	-0.757173
C	6.471687	-1.512814	0.761927
H	7.280701	-1.126218	1.372675
C	5.768836	-2.733700	0.981685
H	5.952538	-3.438545	1.785568
C	1.403387	-1.923179	1.515887
H	0.479189	-1.431130	1.850407
H	1.511771	-2.882006	2.038961
C	1.360412	2.493849	0.791432
C	0.940273	3.620980	0.076092
H	1.130490	3.684147	-0.996432
C	0.282258	4.663816	0.719110
H	-0.034663	5.535432	0.148355
C	0.024048	4.590860	2.087600
H	-0.495206	5.404292	2.590269
C	0.444888	3.477150	2.808459
H	0.259375	3.415552	3.879720
C	1.112331	2.436144	2.164809
H	1.432712	1.569475	2.743417
C	3.493002	1.889667	-1.007763
C	4.192550	2.965441	-0.445102
H	3.872655	3.388309	0.506805
C	5.294542	3.511565	-1.095583
H	5.827113	4.346856	-0.645339
C	5.709145	2.996582	-2.322442
H	6.568903	3.427119	-2.831653
C	5.010566	1.940160	-2.898697
H	5.319507	1.540835	-3.862658
C	3.907965	1.393880	-2.245615
H	3.352679	0.573614	-2.700459
C	-0.133468	-3.460286	-0.245824
C	-0.254409	-4.275140	-1.375145
H	0.441920	-4.157059	-2.205323
C	-1.256866	-5.235589	-1.437108
H	-1.342547	-5.864396	-2.320724
C	-2.135794	-5.401743	-0.368572
H	-2.911780	-6.162914	-0.410069

C	-2.011368	-4.590563	0.754634
H	-2.687839	-4.723196	1.597836
C	-1.021083	-3.610232	0.820066
H	-0.948319	-2.986404	1.708379
H	0.060778	1.180304	-2.081850
H	-0.627525	-0.950819	-2.257311
H	-0.635581	0.024436	-0.071515
H	1.520846	-0.445413	-2.657345
O	-4.022954	-1.009287	1.077902
C	-5.019179	-1.790932	1.747355
H	-5.807875	-1.065477	2.003942
H	-3.349473	-1.608777	0.719554
O	-5.299877	1.507333	-0.722154
C	-6.648825	1.687588	-0.266988
H	-7.088850	2.533999	-0.819036
H	-5.315242	1.284225	-1.663492
C	-2.644443	2.872856	-2.912748
H	-2.664710	3.716422	-3.613947
H	-1.975573	2.105588	-3.323669
H	-3.658116	2.454002	-2.852802
C	-3.109855	4.346673	-0.928708
H	-4.114281	3.917327	-0.806683
H	-2.748134	4.666274	0.056995
H	-3.191572	5.233493	-1.568010
C	-3.457234	-2.619808	-2.256612
H	-2.504661	-2.693073	-2.800284
H	-3.280353	-2.924627	-1.217219
H	-4.161756	-3.329236	-2.708823
C	-4.131819	-0.710917	-3.745409
H	-4.557763	0.300207	-3.774209
H	-3.146286	-0.680294	-4.231848
H	-4.781096	-1.373374	-4.331121
C	-6.573211	2.030924	1.200129
H	-5.988328	2.943658	1.364125
H	-7.578429	2.187056	1.607346
H	-6.099916	1.208061	1.756029
C	-7.460855	0.438238	-0.526587
H	-7.054093	-0.405405	0.048975
H	-8.505578	0.584966	-0.227908
H	-7.449825	0.171173	-1.591419
C	-3.915137	1.381271	3.942035
H	-3.626567	1.087841	4.958269
H	-4.566739	2.260542	4.009106
H	-4.492283	0.558748	3.497603
C	-5.582709	-2.844766	0.816437
H	-4.802653	-3.573886	0.549367
H	-6.402116	-3.391223	1.298053
H	-5.964397	-2.396887	-0.109978
C	-4.456469	-2.400740	3.014745
H	-4.100326	-1.631351	3.709740
H	-5.215721	-3.002913	3.527881
H	-3.610088	-3.059312	2.771353
C	-1.716679	0.508392	3.071739
H	-2.224397	-0.411679	2.753042
H	-0.904644	0.710231	2.360388
H	-1.273057	0.343753	4.062647

2-Li-iso

E (BS1) = -3261.46173687

E (BS2) = -4402.147901

$G_{298.15,1M}$ (BS2) = -4401.130773

Li	3.186895000	-0.369915000	-0.069156000
H	1.918523000	-1.272488000	2.002245000
O	2.477853000	-1.722720000	1.344495000
C	3.473317000	-2.505074000	2.011356000
H	4.420131000	-1.929875000	2.016220000
H	2.187264000	0.780715000	-1.945321000
H	1.564886000	-1.489596000	-1.696928000
O	2.474309000	-1.815045000	-1.533856000
O	3.128417000	0.980340000	-1.754916000
C	2.962410000	-2.474253000	-2.698727000
H	4.017565000	-2.699658000	-2.468175000
C	3.307406000	2.372457000	-2.020672000
H	2.534921000	2.931124000	-1.469474000
Fe	-4.496686000	0.956360000	0.935738000
Ir	-0.540835000	0.290475000	-1.604211000
P	-1.980460000	-1.071964000	-0.376239000
S	-1.308812000	2.359173000	-0.508172000
C	-2.872485000	-0.278825000	1.007852000
C	-2.562281000	1.004345000	1.588684000
C	-3.466074000	1.218686000	2.675926000
H	-3.502245000	2.118157000	3.284120000
C	-4.329133000	0.092092000	2.778557000
H	-5.152162000	-0.021036000	3.476261000
C	-3.973097000	-0.823936000	1.752033000
H	-4.472367000	-1.764027000	1.541146000
C	-4.884360000	2.637726000	-0.165143000
H	-4.214113000	3.485833000	-0.253550000
C	-4.888002000	1.484285000	-1.002061000
H	-4.219748000	1.301063000	-1.837811000
C	-5.885541000	0.588073000	-0.515082000
H	-6.107666000	-0.396766000	-0.915241000
C	-6.498254000	1.190065000	0.622372000
H	-7.266767000	0.743003000	1.244061000
C	-5.878539000	2.455729000	0.839700000
H	-6.096041000	3.140086000	1.652824000
C	-1.454218000	1.951619000	1.271549000
H	-0.495604000	1.511731000	1.575534000
H	-1.611911000	2.894711000	1.811453000
C	-1.140285000	-2.478402000	0.469564000
C	-0.514914000	-3.442357000	-0.330629000
H	-0.518678000	-3.329020000	-1.415937000
C	0.093262000	-4.552225000	0.241872000
H	0.565762000	-5.298778000	-0.394835000
C	0.094491000	-4.711820000	1.627378000
H	0.563014000	-5.585527000	2.077225000
C	-0.502395000	-3.747291000	2.431213000
H	-0.507411000	-3.865998000	3.514264000
C	-1.114701000	-2.634061000	1.855681000
H	-1.590856000	-1.893816000	2.497602000
C	-3.347132000	-1.989199000	-1.226608000
C	-3.943240000	-3.120579000	-0.653886000
H	-3.570633000	-3.516584000	0.290696000
C	-5.006626000	-3.757855000	-1.285397000
H	-5.459196000	-4.635002000	-0.827049000
C	-5.482290000	-3.282237000	-2.506001000
H	-6.309899000	-3.785222000	-3.001995000
C	-4.883303000	-2.172719000	-3.094512000
H	-5.237926000	-1.804456000	-4.055194000
C	-3.821058000	-1.533423000	-2.458612000
H	-3.341294000	-0.672120000	-2.923646000
C	0.038893000	3.547929000	-0.452626000
C	0.130980000	4.420588000	-1.540435000
H	-0.594394000	4.354931000	-2.351251000
C	1.146764000	5.368927000	-1.587393000
H	1.210053000	6.044509000	-2.437902000
C	2.070603000	5.460503000	-0.547632000

H	2.860915000	6.207219000	-0.581595000
C	1.978125000	4.587570000	0.531918000
H	2.702901000	4.642559000	1.342967000
C	0.968933000	3.627761000	0.584103000
H	0.935879000	2.941564000	1.427483000
H	-0.040358000	-1.036271000	-2.381880000
H	0.549961000	1.144185000	-2.522785000
H	0.639584000	0.108408000	-0.377561000
H	-1.584926000	0.540547000	-2.914059000
O	3.139603000	1.184395000	1.468845000
C	4.092651000	1.120139000	2.539018000
H	4.267398000	0.057479000	2.793098000
H	2.263021000	0.986404000	1.847910000
O	5.154851000	-0.851988000	-0.469024000
C	6.499739000	-1.092673000	-0.033525000
H	6.469079000	-0.934338000	1.055880000
H	5.143381000	-0.904612000	-1.437118000
C	0.234448000	0.272989000	3.933168000
O	0.924427000	0.056999000	2.938743000
C	-0.094400000	1.662639000	4.369732000
C	-0.316304000	-0.836621000	4.764725000
H	0.299855000	2.408388000	3.673309000
H	0.101748000	-1.801637000	4.466068000
H	0.327769000	1.835023000	5.368570000
H	-1.182083000	1.774861000	4.471090000
H	-1.409386000	-0.856520000	4.646427000
H	-0.122398000	-0.648514000	5.827218000
C	3.685217000	-3.773147000	1.214863000
H	2.769771000	-4.381080000	1.213580000
H	3.943859000	-3.537949000	0.174932000
H	4.498682000	-4.368810000	1.647755000
C	3.064788000	-2.780964000	3.441488000
H	3.834178000	-3.363925000	3.960958000
H	2.914807000	-1.845052000	3.998161000
H	2.125677000	-3.351749000	3.461938000
C	6.903234000	-2.521223000	-0.331766000
H	6.882776000	-2.700850000	-1.416458000
H	7.922187000	-2.719838000	0.021405000
H	6.226734000	-3.236794000	0.149345000
C	7.445767000	-0.097678000	-0.672652000
H	7.155781000	0.937387000	-0.454471000
H	8.470743000	-0.249784000	-0.314298000
H	7.447630000	-0.229189000	-1.764578000
C	4.669791000	2.772403000	-1.509819000
H	5.453405000	2.190678000	-2.017335000
H	4.854833000	3.835602000	-1.707048000
H	4.751694000	2.605148000	-0.429864000
C	3.157253000	2.651421000	-3.503220000
H	2.179521000	2.313302000	-3.871296000
H	3.242819000	3.727215000	-3.704922000
H	3.940102000	2.131615000	-4.073536000
C	2.219731000	-3.777565000	-2.918780000
H	2.256957000	-4.401806000	-2.017172000
H	1.164724000	-3.578715000	-3.157583000
H	2.653532000	-4.343702000	-3.752285000
C	2.899111000	-1.575695000	-3.919070000
H	1.854549000	-1.319280000	-4.148109000
H	3.446717000	-0.641266000	-3.751434000
H	3.324034000	-2.079444000	-4.796007000
C	3.587176000	1.846635000	3.768315000
H	3.306567000	2.877522000	3.509978000
H	2.717362000	1.346940000	4.207673000
H	4.369995000	1.888774000	4.535054000
C	5.380989000	1.724379000	2.035840000
H	6.183977000	1.580962000	2.768332000
H	5.684915000	1.259925000	1.091749000
H	5.256837000	2.803309000	1.867823000

$TS_{2\text{-}3}$-Li-iso

E (BS1) = -3261.44825002

E (BS2) = -4402.130791

$G_{298.15,1M}$ (BS2) = -4401.117590

Li	-3.382582	-0.184599	0.876927
H	-3.864925	-2.153127	-0.788145
O	-4.691191	-2.675412	-0.772284
C	-5.458425	-2.241583	-1.903347
H	-5.620423	-1.151560	-1.816462
H	-4.324857	1.241147	2.578335
H	-1.504767	-1.432825	1.548339
O	-2.427451	-1.660605	1.799070
O	-3.523044	1.391958	2.056387
C	-2.384467	-2.220106	3.114936
H	-1.701729	-1.608095	3.730976
C	-2.715636	2.346911	2.763803
H	-1.857923	2.525371	2.099174
Fe	4.803693	1.474556	-0.982514
Ir	0.715366	-0.176694	0.981454
P	2.643698	-1.103085	0.002314
S	1.264339	2.055836	0.152542
C	3.490939	-0.066926	-1.245952
C	2.991113	1.144376	-1.852685
C	3.948213	1.566829	-2.827306
H	3.868408	2.472129	-3.421720
C	5.028771	0.642113	-2.835629
H	5.929202	0.716677	-3.436046
C	4.755668	-0.355083	-1.861996
H	5.407711	-1.182699	-1.603157
C	4.689803	3.184409	0.133456
H	3.847620	3.868815	0.124835
C	4.834500	2.047805	0.980735
H	4.122860	1.718420	1.731174
C	6.051533	1.389301	0.633409
H	6.427464	0.470729	1.073676
C	6.658177	2.121265	-0.428536
H	7.574712	1.855529	-0.944596
C	5.816438	3.229667	-0.738036
H	5.981675	3.953557	-1.528954
C	1.718008	1.887076	-1.624954
H	0.871200	1.398294	-2.124882
H	1.817335	2.905586	-2.022928
C	2.316167	-2.641712	-0.956998
C	1.977064	-3.804557	-0.253033
H	1.967889	-3.794935	0.837955
C	1.653425	-4.973724	-0.931310
H	1.400795	-5.871291	-0.370397
C	1.649547	-4.995112	-2.325772
H	1.394234	-5.909472	-2.857161
C	1.972810	-3.842337	-3.033688
H	1.972137	-3.850911	-4.121914
C	2.308384	-2.671640	-2.353502
H	2.565599	-1.777788	-2.921577
C	4.053446	-1.654961	1.063812
C	4.993642	-2.591322	0.614750
H	4.875153	-3.061215	-0.361058
C	6.080692	-2.940518	1.409549
H	6.802011	-3.669897	1.046582
C	6.240124	-2.365287	2.668905

H	7.088386	-2.642332	3.291401
C	5.302783	-1.446844	3.131825
H	5.412607	-1.003229	4.119349
C	4.214968	-1.097946	2.334437
H	3.473558	-0.387762	2.700386
C	-0.181159	3.105036	-0.069888
C	-0.033065	4.460170	0.226099
H	0.930959	4.837500	0.564958
C	-1.118860	5.322527	0.091485
H	-1.002523	6.377637	0.329432
C	-2.343931	4.832405	-0.349243
H	-3.192846	5.505064	-0.454823
C	-2.489492	3.477856	-0.645797
H	-3.447545	3.076944	-0.975534
C	-1.412305	2.607595	-0.500923
H	-1.537891	1.541863	-0.695622
H	0.382248	-1.618222	1.650766
H	-0.654247	0.419436	1.687689
H	-0.266383	-0.594814	-0.423666
H	1.465970	0.199464	2.407104
O	-4.541901	1.167825	-1.699908
C	-5.054670	1.402104	-3.004266
H	-5.606758	0.504842	-3.347963
H	-3.807217	0.525337	-1.735542
O	-5.338244	-0.643408	0.977151
C	-6.649332	-0.099964	0.771280
H	-6.898312	-0.189940	-0.301604
H	-5.309688	-1.527782	0.554232
C	-1.611479	-1.186969	-1.462667
O	-2.669818	-0.709501	-0.958683
C	-1.062208	-0.560048	-2.710223
C	-1.298903	-2.639867	-1.262774
H	-1.192330	0.527714	-2.702573
H	-1.536235	-2.963469	-0.244199
H	-1.645734	-0.966596	-3.553227
H	-0.011396	-0.819656	-2.877751
H	-0.256886	-2.865337	-1.499732
H	-1.937819	-3.202890	-1.962718
C	-3.932125	1.686980	-3.980644
H	-3.346921	2.554739	-3.644488
H	-3.254589	0.826234	-4.061589
H	-4.323141	1.900973	-4.982791
C	-6.026763	2.554659	-2.894994
H	-6.502305	2.758052	-3.861310
H	-6.815604	2.333432	-2.165370
H	-5.503803	3.464889	-2.570037
C	-4.713981	-2.520717	-3.192106
H	-3.763751	-1.969269	-3.225558
H	-4.495997	-3.593390	-3.283520
H	-5.310384	-2.215686	-4.061007
C	-6.786293	-2.955905	-1.837765
H	-7.438189	-2.638504	-2.659369
H	-6.637566	-4.041147	-1.914004
H	-7.300304	-2.746623	-0.890800
C	-6.637471	1.363997	1.143901
H	-6.459424	1.492679	2.221537
H	-5.870577	1.913723	0.584209
H	-7.612914	1.811003	0.919115
C	-7.654374	-0.886417	1.586076
H	-8.671938	-0.510869	1.424524
H	-7.637271	-1.950559	1.317347
H	-7.420081	-0.801430	2.656328
C	-2.238363	1.760460	4.073686
H	-3.092450	1.552287	4.734362
H	-1.688045	0.825817	3.905826
H	-1.574434	2.461629	4.593703
C	-3.500382	3.627228	2.949206
H	-3.883643	3.992471	1.988517
H	-4.355665	3.457405	3.619848
H	-2.873691	4.410277	3.392640
C	-1.858724	-3.639034	3.041174
H	-1.793236	-4.089072	4.039282
H	-2.528870	-4.255277	2.425670
H	-0.857976	-3.662984	2.589016
C	-3.777976	-2.153409	3.693840
H	-4.129166	-1.116140	3.764620
H	-4.480053	-2.715531	3.061854
H	-3.797463	-2.591231	4.698444

3-Li-iso

E (BS1) = -3261.47211095

E (BS2) = -4402.154493

$G_{298.15,1M}$ (BS2) = -4401.139221

Li	-3.335891	-0.259225	0.873138
H	-3.889129	-1.883471	-1.005697
O	-4.675613	-2.499384	-0.946410
C	-5.544542	-2.177918	-2.030855
H	-5.808488	-1.105320	-1.969375
H	-4.216077	1.101335	2.651683
H	-1.443143	-1.488491	1.628133
O	-2.363018	-1.694475	1.895499
O	-3.430969	1.302509	2.120614
C	-2.311556	-2.151719	3.249520
H	-1.644014	-1.481095	3.820119
C	-2.706715	2.341995	2.794425
H	-1.856223	2.558696	2.131463
Fe	4.775929	1.511081	-0.970439
Ir	0.751582	-0.184315	1.004484
P	2.662649	-1.103284	-0.052532
S	1.206672	2.039011	0.088204
C	3.504005	-0.050479	-1.281908
C	2.987670	1.156823	-1.883754
C	3.954990	1.612088	-2.832503
H	3.866447	2.521999	-3.418351
C	5.055085	0.710889	-2.831058
H	5.965329	0.813443	-3.412084
C	4.785802	-0.305600	-1.876794
H	5.450840	-1.121831	-1.614864
C	4.618972	3.203949	0.167686
H	3.769261	3.878719	0.153582
C	4.763429	2.057417	1.001433
H	4.044547	1.709754	1.736467
C	5.994823	1.419226	0.667026
H	6.375055	0.499378	1.101080
C	6.610766	2.174364	-0.372971
H	7.539481	1.927391	-0.876343
C	5.760431	3.276113	-0.682084
H	5.930568	4.013432	-1.459316
C	1.686221	1.857737	-1.683257
H	0.863443	1.336113	-2.190684
H	1.755639	2.874975	-2.089661
C	2.309861	-2.625929	-1.022024
C	1.961409	-3.791906	-0.327550
H	1.949554	-3.792296	0.763467
C	1.637535	-4.953686	-1.017898
H	1.378876	-5.855344	-0.466629
C	1.638809	-4.961548	-2.412340

H	1.381449	-5.869902	-2.952824
C	1.968617	-3.804202	-3.109721
H	1.971424	-3.803577	-4.197824
C	2.308056	-2.641532	-2.418740
H	2.575466	-1.744957	-2.977661
C	4.048857	-1.666808	1.022627
C	4.992811	-2.595439	0.565974
H	4.887220	-3.041491	-0.422369
C	6.066013	-2.966110	1.369392
H	6.791138	-3.688568	1.000696
C	6.206119	-2.421650	2.644455
H	7.043141	-2.716252	3.273857
C	5.264779	-1.511048	3.114391
H	5.359982	-1.091600	4.113703
C	4.191528	-1.138585	2.308428
H	3.454312	-0.430466	2.683797
C	-0.253610	3.064018	-0.127865
C	-0.112286	4.426259	0.141251
H	0.857020	4.820774	0.443323
C	-1.212732	5.272079	0.029242
H	-1.102253	6.332605	0.244625
C	-2.446571	4.757221	-0.356503
H	-3.308453	5.416317	-0.440275
C	-2.583996	3.396523	-0.625250
H	-3.545868	2.974048	-0.914244
C	-1.490025	2.541656	-0.510457
H	-1.620301	1.473365	-0.694708
H	0.520089	-1.601403	1.764656
H	-0.535564	0.389555	1.867923
H	-0.700958	-0.782910	-0.513851
H	1.593094	0.246238	2.242001
O	-4.554971	1.073157	-1.722625
C	-4.926920	1.354177	-3.058653
H	-5.358538	0.445450	-3.524824
H	-3.800605	0.434794	-1.674952
O	-5.313668	-0.702125	1.000556
C	-6.606757	-0.117543	0.809095
H	-6.868703	-0.197746	-0.260949
H	-5.261578	-1.509645	0.440556
C	-1.487896	-1.153728	-1.260596
O	-2.729383	-0.688578	-0.902151
C	-1.043411	-0.632627	-2.622160
C	-1.392938	-2.675222	-1.223942
H	-1.109831	0.462996	-2.665086
H	-1.633101	-3.062316	-0.224361
H	-1.700260	-1.040695	-3.405900
H	-0.011249	-0.931906	-2.858298
H	-0.388744	-3.020150	-1.499275
H	-2.107675	-3.109146	-1.937998
C	-3.733778	1.794354	-3.884398
H	-3.272702	2.690783	-3.446218
H	-2.973612	1.003695	-3.926359
H	-4.030927	2.029002	-4.914295
C	-6.001853	2.417503	-3.002291
H	-6.371287	2.660491	-4.005424
H	-6.852189	2.080160	-2.396027
H	-5.603610	3.336825	-2.549912
C	-4.850167	-2.426544	-3.354745
H	-3.947524	-1.805574	-3.441361
H	-4.553153	-3.481222	-3.437286
H	-5.509423	-2.187630	-4.199008
C	-6.797909	-3.006388	-1.874685
H	-7.515527	-2.785968	-2.673429
H	-6.553258	-4.076290	-1.916230
H	-7.284459	-2.804655	-0.911259
C	-6.546726	1.345083	1.185384
H	-6.347479	1.463267	2.260516
H	-5.769051	1.870895	0.617102
H	-7.510472	1.824693	0.977130
C	-7.631256	-0.873886	1.629392
H	-8.637093	-0.461333	1.483924
H	-7.653912	-1.935330	1.351973
H	-7.381451	-0.806073	2.697591
C	-2.201247	1.846870	4.131729
H	-3.045143	1.591866	4.789026
H	-1.572254	0.956432	4.007656
H	-1.607708	2.620640	4.633639
C	-3.582983	3.569067	2.927507
H	-3.984776	3.871971	1.953166
H	-4.427283	3.363870	3.601986
H	-3.016167	4.410548	3.344085
C	-1.756996	-3.561174	3.282336
H	-1.674782	-3.932324	4.311153
H	-2.416799	-4.236401	2.720521
H	-0.759315	-3.598765	2.823594
C	-3.708273	-2.067242	3.817602
H	-4.075824	-1.033164	3.806770
H	-4.397263	-2.687396	3.227720
H	-3.724679	-2.427129	4.852712

$TS_{3\text{-}4}$-Li-iso

E (BS1) = -3261.4654502
E (BS2) = -4402.14802989
$G_{298.15,1M}$ (BS2) = -4401.128943

Li	3.529673	-0.092191	-0.495701
H	2.117830	-1.418220	1.190794
O	2.713835	-1.819988	0.500910
C	3.555851	-2.829276	1.046074
H	4.582276	-2.422642	1.119795
H	4.365572	1.534080	-2.516723
H	1.923257	-1.131416	-1.882041
O	2.800231	-1.166213	-2.316982
O	4.068714	1.588772	-1.597597
C	2.675041	-0.613149	-3.631122
H	2.490232	0.472629	-3.540206
C	3.688554	2.944142	-1.320395
H	3.292371	2.894690	-0.299240
Fe	-4.864553	0.969449	-0.185293
Ir	-0.228118	0.068868	-0.508879
P	-2.133751	-1.148909	0.173210
S	-1.322447	2.240260	-0.090395
C	-3.540592	-0.195973	0.841478
C	-3.501630	1.166410	1.319566
C	-4.812785	1.499287	1.781414
H	-5.104370	2.471518	2.167238
C	-5.660708	0.372430	1.599334
H	-6.723415	0.331397	1.813023
C	-4.886828	-0.665842	1.015878
H	-5.257241	-1.641619	0.719626
C	-4.585073	2.478026	-1.538138
H	-3.918303	3.320852	-1.388002
C	-4.228566	1.223418	-2.112350
H	-3.243805	0.947831	-2.476948
C	-5.375777	0.375762	-2.070644
H	-5.417883	-0.658549	-2.398957
C	-6.441005	1.109912	-1.472229
H	-7.435371	0.731928	-1.259392

C	-5.952191	2.407965	-1.142144
H	-6.510192	3.189273	-0.637177
C	-2.359110	2.118351	1.433864
H	-1.691063	1.840591	2.260127
H	-2.747115	3.126994	1.625729
C	-1.822295	-2.402203	1.488238
C	-0.891429	-3.410000	1.207812
H	-0.369986	-3.411367	0.249211
C	-0.636372	-4.414761	2.134914
H	0.078465	-5.200644	1.896395
C	-1.296704	-4.415410	3.362984
H	-1.098046	-5.200799	4.089112
C	-2.210179	-3.407779	3.656136
H	-2.726650	-3.400485	4.613932
C	-2.475500	-2.406924	2.722557
H	-3.200379	-1.629504	2.961234
C	-2.947623	-2.213031	-1.098656
C	-3.782177	-3.275291	-0.727550
H	-3.921610	-3.519329	0.325238
C	-4.433984	-4.035625	-1.692898
H	-5.080765	-4.855474	-1.387172
C	-4.249659	-3.755873	-3.045352
H	-4.754673	-4.355405	-3.799816
C	-3.405914	-2.717061	-3.426270
H	-3.245397	-2.501177	-4.480532
C	-2.759005	-1.951566	-2.458459
H	-2.098547	-1.140807	-2.762139
C	-0.246630	3.582542	0.433816
C	-0.696714	4.893223	0.261052
H	-1.683211	5.078575	-0.163371
C	0.123242	5.955790	0.627172
H	-0.224180	6.977413	0.490260
C	1.387782	5.711436	1.160568
H	2.027621	6.544582	1.443293
C	1.831752	4.402784	1.328813
H	2.820808	4.204533	1.741913
C	1.018017	3.333615	0.959501
H	1.362087	2.307005	1.072727
H	0.379115	-1.345407	-0.998065
H	1.133766	0.794386	-1.106759
H	-0.711989	-0.245337	2.851178
H	-0.810027	0.235100	-1.946769
O	3.375371	0.945303	1.297201
C	4.527095	0.669067	2.086688
H	4.828262	-0.387746	1.931867
H	2.551941	0.520500	1.697861
O	5.324328	-1.179108	-1.084219
C	6.463232	-2.004906	-0.829417
H	6.131561	-2.919480	-0.305726
H	4.726091	-1.645795	-1.691685
C	0.368680	-0.134630	3.105118
O	1.145859	-0.260524	1.957632
C	0.519889	1.233688	3.767317
C	0.677587	-1.218833	4.133815
H	0.275795	2.048096	3.074196
H	0.571244	-2.219715	3.696112
H	1.555118	1.388633	4.104033
H	-0.136528	1.329505	4.644223
H	0.005108	-1.153326	5.001527
H	1.710029	-1.117215	4.502776
C	3.573809	-4.005989	0.092265
H	4.266592	-4.783273	0.438999
H	2.569442	-4.446487	0.016259
H	3.884247	-3.694906	-0.914189
C	3.104855	-3.234444	2.431655
H	3.118601	-2.376869	3.116971
H	2.079510	-3.631591	2.401167
H	3.762917	-4.009972	2.841786
C	7.122215	-2.397157	-2.134017
H	6.420330	-2.942214	-2.779023
H	7.466855	-1.504201	-2.672773
H	7.986954	-3.046992	-1.953962
C	7.387136	-1.230173	0.078361
H	7.726528	-0.309045	-0.414613
H	6.880819	-0.958004	1.013181
H	8.269100	-1.830557	0.328442
C	4.894484	3.858275	-1.355075
H	4.613895	4.871112	-1.037064
H	5.687358	3.496625	-0.689914
H	5.302113	3.925074	-2.373967
C	2.602682	3.406186	-2.268359
H	2.976533	3.417075	-3.303089
H	1.726762	2.747224	-2.222090
H	2.281990	4.426613	-2.019526
C	1.519405	-1.256697	-4.367456
H	1.415080	-0.831089	-5.372876
H	1.685094	-2.338103	-4.464227
H	0.574532	-1.098499	-3.830978
C	3.993919	-0.840730	-4.333549
H	4.829045	-0.399689	-3.774587
H	4.183404	-1.918222	-4.438967
H	3.980365	-0.396847	-5.335476
C	5.640152	1.568923	1.595011
H	6.577673	1.360931	2.125104
H	5.811566	1.430844	0.519791
H	5.374505	2.621993	1.768658
C	4.252096	0.878222	3.560989
H	3.931166	1.914067	3.743103
H	3.463419	0.203427	3.917768
H	5.154844	0.687744	4.154549

4-Li-iso

E (BS1) = -3261.48472036

E (BS2) = -4402.167564

$G_{298.15,1M}$ (BS2) = -4401.143569

H	-0.736457	2.163229	2.202152
H	0.911227	-0.680738	-0.428675
H	-0.761915	-0.052871	-1.818538
Li	3.601343	-0.316584	-0.211167
H	2.018928	0.120594	1.745036
O	2.737304	-0.588790	1.768779
C	3.303243	-0.652352	3.075694
H	2.479963	-0.584866	3.809535
H	1.983955	-0.025441	-1.861248
O	2.859917	-0.334217	-2.180152
C	2.894812	-0.132592	-3.593417
H	2.831091	0.952425	-3.799168
O	0.947278	1.285473	1.402221
C	0.361344	2.105861	2.370997
C	0.914936	3.521304	2.294865
C	0.574927	1.559282	3.777188
H	0.787542	3.933073	1.286120
H	1.990444	3.513488	2.530958
H	0.416930	4.195475	3.006359
H	0.060260	2.176326	4.526809
H	0.206349	0.530682	3.864976
H	1.647576	1.560496	4.023746

O	3.307804	1.757156	0.141966
C	3.508030	2.785360	-0.823851
H	2.399787	1.810376	0.543881
H	2.658672	2.780519	-1.533752
Fe	-4.722991	-0.302523	-0.845481
Ir	-0.185464	0.499361	-0.441309
P	-1.634371	-0.970015	0.663133
S	-1.928537	2.254437	-0.697645
C	-3.397982	-0.505283	0.690901
C	-3.920898	0.836998	0.647993
C	-5.341110	0.753955	0.786328
H	-6.017418	1.603130	0.759618
C	-5.704966	-0.614626	0.917053
H	-6.715373	-1.002372	0.991567
C	-4.515987	-1.390429	0.854451
H	-4.462632	-2.473834	0.883622
C	-4.549298	0.679745	-2.633707
H	-4.186918	1.695140	-2.750671
C	-3.748600	-0.499054	-2.634768
H	-2.668240	-0.530577	-2.740103
C	-4.604794	-1.615849	-2.401372
H	-4.292197	-2.651204	-2.303432
C	-5.936371	-1.124895	-2.260861
H	-6.814846	-1.720146	-2.035194
C	-5.901359	0.293475	-2.402081
H	-6.749129	0.963760	-2.308815
C	-3.197059	2.141727	0.621080
H	-2.704120	2.323855	1.587075
H	-3.923025	2.944368	0.443081
C	-1.327366	-1.303832	2.448084
C	-0.114331	-1.908141	2.799786
H	0.618799	-2.137142	2.024434
C	0.170534	-2.206429	4.127529
H	1.110509	-2.694005	4.383559
C	-0.742240	-1.874109	5.128268
H	-0.519684	-2.102943	6.168298
C	-1.934904	-1.242245	4.791965
H	-2.647969	-0.973637	5.568803
C	-2.230796	-0.963413	3.457891
H	-3.177555	-0.484535	3.209321
C	-1.713474	-2.689197	-0.009574
C	-2.173367	-3.758267	0.769744
H	-2.453844	-3.594822	1.810037
C	-2.277013	-5.036984	0.230356
H	-2.638672	-5.855506	0.849318
C	-1.913894	-5.268948	-1.094774
H	-1.991958	-6.269727	-1.514446
C	-1.441871	-4.217094	-1.875070
H	-1.142919	-4.390567	-2.907297
C	-1.343397	-2.936470	-1.335341
H	-0.967207	-2.117447	-1.946813
C	-1.297537	3.917211	-0.447182
C	-1.806790	4.816913	0.488732
H	-2.608572	4.538405	1.166735
C	-1.270519	6.101857	0.570821
H	-1.669169	6.795365	1.308425
C	-0.239511	6.496671	-0.273145
H	0.173302	7.500263	-0.201896
C	0.258448	5.597025	-1.215084
H	1.062516	5.893361	-1.886004
C	-0.265908	4.314067	-1.304731
H	0.133941	3.608893	-2.032685
H	0.836733	1.417492	-1.378284
O	5.579766	-0.466810	0.029546
C	6.539181	-0.803953	-0.988122
H	6.319947	-0.104703	-1.807843
H	5.759264	-1.029222	0.797749
O	3.124969	-2.558341	-0.012589
C	2.183334	-3.408840	-0.669481
H	1.320045	-2.798725	-0.993114
H	2.699582	-2.163694	0.773398
C	3.586696	4.148111	-0.163995
H	2.646164	4.411364	0.333083
H	4.389311	4.157184	0.586832
H	3.804778	4.925645	-0.907421
C	4.782925	2.467819	-1.573770
H	5.642938	2.487315	-0.888754
H	4.727888	1.473935	-2.034390
H	4.960469	3.206222	-2.364591
C	1.714110	-0.828097	-4.242722
H	1.718807	-0.677383	-5.329152
H	1.752500	-1.908649	-4.041666
H	0.765069	-0.441310	-3.848288
C	4.214731	-0.654390	-4.110256
H	5.061959	-0.150367	-3.628364
H	4.301022	-1.733716	-3.922682
H	4.291927	-0.489318	-5.191163
C	7.939982	-0.556922	-0.475474
H	8.055493	0.478002	-0.133326
H	8.163180	-1.228039	0.365911
H	8.680187	-0.745735	-1.261580
C	6.329738	-2.225727	-1.459553
H	7.029154	-2.474807	-2.266800
H	6.499049	-2.930670	-0.632282
H	5.307731	-2.368804	-1.829652
C	2.860017	-3.986455	-1.889416
H	3.712472	-4.615933	-1.597716
H	2.155470	-4.604495	-2.459732
H	3.226181	-3.187904	-2.546486
C	1.700316	-4.491150	0.274643
H	0.964009	-5.139586	-0.218211
H	2.544112	-5.114091	0.602347
H	1.224221	-4.062891	1.167318
C	3.978688	-1.995860	3.239786
H	4.363589	-2.111525	4.259868
H	3.278320	-2.818760	3.042556
H	4.828109	-2.098206	2.549150
C	4.260927	0.500656	3.301042
H	4.661505	0.483618	4.322522
H	5.103751	0.441099	2.598739
H	3.759948	1.465243	3.146543

D3. Sodium system

1-Na-iso

E (BS1) = -3223.14786932
E (BS2) = -4363.770703
$G_{298.15,1M}$ (BS2) = -4362.836829

Na	3.146214	-0.737233	0.129519
H	1.866553	-2.372312	1.615318
O	2.654558	-2.040481	2.079345
C	2.283522	-1.500786	3.350839
H	1.717353	-2.266746	3.908554
H	2.382305	0.368324	-2.274113
H	1.094895	-1.977125	-0.960449
O	1.782875	-2.552040	-0.563419
O	3.284805	0.052507	-2.076090

C	2.006708	-3.691721	-1.390221
H	1.043896	-4.212919	-1.539247
C	4.243725	1.068578	-2.376732
H	4.990021	1.020526	-1.564147
Fe	-4.453006	1.250533	1.112201
Ir	-0.494712	0.152241	-1.356877
P	-2.131244	-1.031440	-0.174678
S	-1.171244	2.294865	-0.337827
C	-2.954488	-0.130265	1.189199
C	-2.529445	1.117135	1.776353
C	-3.414719	1.411809	2.858935
H	-3.369654	2.308276	3.470092
C	-4.377759	0.369449	2.954762
H	-5.210730	0.330745	3.648693
C	-4.101945	-0.573763	1.929188
H	-4.684003	-1.463987	1.715805
C	-4.673313	2.969960	0.024805
H	-3.926419	3.753257	-0.047380
C	-4.772841	1.828834	-0.823156
H	-4.115889	1.593302	-1.654506
C	-5.853946	1.023692	-0.355741
H	-6.161443	0.067679	-0.768799
C	-6.421807	1.669784	0.780803
H	-7.235285	1.290173	1.389997
C	-5.691473	2.871305	1.016688
H	-5.854320	3.565519	1.834221
C	-1.353514	1.978017	1.455098
H	-0.425106	1.504923	1.804016
H	-1.472370	2.949427	1.952524
C	-1.497739	-2.514561	0.720015
C	-1.183046	-3.666985	-0.008303
H	-1.378071	-3.701306	-1.081414
C	-0.622873	-4.773128	0.621474
H	-0.390444	-5.665014	0.041527
C	-0.349291	-4.737057	1.987834
H	0.094198	-5.600495	2.479505
C	-0.660171	-3.595824	2.722052
H	-0.469554	-3.565538	3.793527
C	-1.238331	-2.494001	2.092870
H	-1.491713	-1.613792	2.683666
C	-3.578323	-1.760162	-1.070926
C	-4.334635	-2.804755	-0.522975
H	-4.041400	-3.255485	0.424356
C	-5.458972	-3.289100	-1.183599
H	-6.034009	-4.101832	-0.744458
C	-5.841568	-2.742276	-2.406996
H	-6.718486	-3.124743	-2.925262
C	-5.089096	-1.715278	-2.968216
H	-5.372851	-1.290779	-3.929203
C	-3.963521	-1.231267	-2.304750
H	-3.365477	-0.435851	-2.749016
C	0.203868	3.447404	-0.346881
C	0.422020	4.116911	-1.554974
H	-0.207787	3.897359	-2.417045
C	1.440063	5.056188	-1.658005
H	1.602599	5.568031	-2.604199
C	2.242227	5.346728	-0.555799
H	3.033112	6.089662	-0.632550
C	2.019904	4.683544	0.645948
H	2.631043	4.916228	1.516860
C	1.008752	3.727246	0.758454
H	0.858656	3.227030	1.712918
H	-0.071964	-1.213622	-2.111107
H	0.699734	0.889398	-2.249757
H	0.640526	-0.077838	-0.084649
H	-1.468938	0.448393	-2.699148
O	3.739256	1.336684	1.026970
C	4.807436	1.947704	1.759931
H	5.496387	1.116671	1.981828
H	3.082294	2.021692	0.823893
O	5.163499	-1.703020	-0.567778
C	6.482486	-1.773869	-0.018611
H	7.008262	-2.634511	-0.465380
H	5.222748	-1.519916	-1.516312
C	2.559112	-3.289274	-2.742822
H	2.672595	-4.167890	-3.390093
H	1.889607	-2.580144	-3.246638
H	3.543781	-2.814750	-2.631105
C	2.948770	-4.601042	-0.635201
H	3.901195	-4.083661	-0.451818
H	2.521978	-4.893523	0.332630
H	3.155350	-5.511374	-1.210081
C	3.601127	2.436924	-2.371816
H	2.865419	2.516827	-3.185401
H	3.083027	2.621046	-1.421452
H	4.349629	3.226768	-2.514593
C	4.928203	0.750365	-3.689383
H	5.348872	-0.263620	-3.672310
H	4.210708	0.806716	-4.519093
H	5.743449	1.456834	-3.889913
C	6.315251	-2.004501	1.464055
H	5.755380	-2.926229	1.662137
H	7.291452	-2.081848	1.956144
H	5.769464	-1.161230	1.914141
C	7.255805	-0.506553	-0.314824
H	6.762073	0.357670	0.153062
H	8.276730	-0.573256	0.080023
H	7.325388	-0.323246	-1.395130
C	3.569150	-1.199266	4.086037
H	3.359058	-0.771385	5.073384
H	4.170917	-2.105613	4.222579
H	4.165947	-0.471026	3.517381
C	5.515173	2.974172	0.900491
H	4.827292	3.791931	0.638554
H	6.370141	3.408219	1.432392
H	5.881010	2.523878	-0.031246
C	4.295631	2.537646	3.057219
H	3.813650	1.775128	3.681085
H	5.115108	2.984634	3.632354
H	3.559390	3.328089	2.851505
C	1.420117	-0.266504	3.180792
H	1.981064	0.515932	2.648625
H	0.519818	-0.499013	2.596071
H	1.105678	0.130348	4.154946

2-Na-iso

E (BS1) = -3416.19879052

E (BS2) = -4556.89755349

$G_{298.15,1M}$ (BS2) = -4555,882745

Na	3.014416	-0.469070	-0.267964
H	1.697618	-1.848904	1.736729
O	2.243260	-2.307377	1.071392
C	3.206412	-3.133130	1.724159
H	4.145235	-2.555997	1.844388
H	3.009017	0.797167	-2.391972
H	1.536991	-1.085067	-2.412301
O	2.473414	-1.380890	-2.378394

O	3.678040	1.220574	-1.828071
C	2.844488	-1.923693	-3.644492
H	3.880652	-2.269971	-3.511067
C	3.387252	2.619268	-1.750472
H	2.379844	2.750062	-1.315896
Fe	-4.222594	0.856955	1.369106
Ir	-0.522300	0.443417	-1.676085
P	-1.981003	-1.006778	-0.580288
S	-1.235480	2.328050	-0.276816
C	-2.658770	-0.414729	1.016495
C	-2.222458	0.742378	1.760897
C	-2.974472	0.785629	2.976670
H	-2.898309	1.570391	3.724128
C	-3.869577	-0.320325	2.998896
H	-4.604808	-0.532429	3.767910
C	-3.685508	-1.051320	1.794078
H	-4.246746	-1.930508	1.494582
C	-4.661492	2.726590	0.664560
H	-3.966826	3.559836	0.658987
C	-4.814404	1.758735	-0.369796
H	-4.258230	1.729299	-1.301252
C	-5.787476	0.806324	0.055815
H	-6.099826	-0.075276	-0.495877
C	-6.235864	1.188209	1.353591
H	-6.945712	0.645219	1.968655
C	-5.539338	2.373920	1.730293
H	-5.629141	2.890545	2.683
C	-1.157947	1.744213	1.452524
H	-0.160154	1.309479	1.613141
H	-1.290142	2.616022	2.107781
C	-1.210765	-2.613604	-0.098658
C	-0.688723	-3.414019	-1.122742
H	-0.712935	-3.056414	-2.153320
C	-0.155801	-4.666622	-0.842795
H	0.232102	-5.282678	-1.652149
C	-0.129034	-5.137617	0.469744
H	0.274952	-6.124386	0.688865
C	-0.618624	-4.338117	1.496713
H	-0.599841	-4.695883	2.525627
C	-1.154535	-3.081820	1.214412
H	-1.541532	-2.472816	2.029725
C	-3.507616	-1.640224	-1.418863
C	-4.141795	-2.820232	-1.009158
H	-3.708955	-3.426069	-0.213408
C	-5.321487	-3.238891	-1.616350
H	-5.802486	-4.157404	-1.285846
C	-5.878802	-2.490798	-2.651633
H	-6.798573	-2.821516	-3.129697
C	-5.245087	-1.328914	-3.082058
H	-5.665263	-0.747487	-3.900356
C	-4.065220	-0.909791	-2.470655
H	-3.560469	-0.006391	-2.814315
C	-0.020151	3.644868	-0.272414
C	-0.002735	4.445017	-1.419669
H	-0.674282	4.219503	-2.248132
C	0.868749	5.522770	-1.502167
H	0.873765	6.140840	-2.397665
C	1.723084	5.817637	-0.439955
H	2.398935	6.667937	-0.500275
C	1.707651	5.015393	0.695412
H	2.374967	5.234170	1.527866
C	0.843815	3.923657	0.785521
H	0.863429	3.303549	1.679276
H	-0.150569	-0.753604	-2.700571

H	0.641319	1.355214	-2.439362
H	0.646713	-0.037719	-0.518794
H	-1.566528	0.962752	-2.902110
O	2.905156	0.950596	1.611224
C	3.890271	1.277440	2.593331
H	4.838479	1.340400	2.034962
H	2.099655	0.651150	2.077235
O	5.205587	-1.433246	-0.410729
C	6.171812	-1.063095	0.582346
H	5.652813	-0.954380	1.554182
H	5.063742	-2.388626	-0.358712
C	0.233398	-0.495206	3.887701
O	0.857941	-0.579183	2.831711
C	0.156254	0.794678	4.634294
C	-0.454062	-1.673446	4.488223
H	0.411785	1.640045	3.986589
H	-0.163123	-2.601236	3.987122
H	0.881121	0.752073	5.460215
H	-0.830297	0.936909	5.089303
H	-1.540096	-1.528336	4.387876
H	-0.245173	-1.733227	5.562963
C	3.591226	2.625598	3.216330
H	3.514965	3.403842	2.446623
H	2.639850	2.588387	3.767308
H	4.378059	2.915352	3.923584
C	3.996342	0.183489	3.636640
H	3.080334	0.141208	4.243464
H	4.141036	-0.798429	3.165753
H	4.838773	0.368933	4.313768
C	6.728874	0.277869	0.171520
H	7.406470	0.666338	0.940917
H	7.285279	0.188576	-0.771053
H	5.920474	1.004850	0.023679
C	7.238840	-2.126657	0.709761
H	7.971959	-1.850379	1.476395
H	6.800190	-3.092620	0.995376
H	7.767153	-2.254289	-0.244297
C	4.414877	3.226617	-0.824733
H	4.365580	2.756510	0.167668
H	5.427068	3.089316	-1.230487
H	4.237179	4.301667	-0.703236
C	3.412033	3.234976	-3.133658
H	4.389487	3.069070	-3.607485
H	2.636943	2.791663	-3.773693
H	3.230409	4.316112	-3.085770
C	2.809299	-0.853144	-4.718037
H	3.482888	-0.022353	-4.471662
H	3.115014	-1.260256	-5.689680
H	1.790517	-0.452844	-4.821787
C	1.963072	-3.106602	-3.991113
H	2.288476	-3.580109	-4.925422
H	1.990081	-3.858934	-3.193699
H	0.921714	-2.778118	-4.122607
C	2.725143	-3.560613	3.094078
H	1.794866	-4.138935	3.004021
H	3.474702	-4.189679	3.588686
H	2.533943	-2.691407	3.738056
C	3.478016	-4.319433	0.824596
H	4.324632	-4.908675	1.197170
H	2.593616	-4.969068	0.775865
H	3.701667	-3.996465	-0.201893

$TS_{2\text{-}3}$-Na-iso

E (BS1) = -3416.18522556

E (BS2) = -4556.879519
$G_{298.15,1M}$ (BS2) = -4555.869254

Na	3.223260	-0.379715	0.618289
H	3.757675	2.190890	-0.522404
O	4.608683	2.654810	-0.372217
C	5.394594	2.460560	-1.553197
H	5.552937	1.374688	-1.689124
H	4.089770	-1.839640	2.975915
H	1.553798	1.363534	1.528082
O	2.479287	1.517134	1.812489
O	3.450614	-2.010405	2.269729
C	2.432502	1.999561	3.153710
H	1.666722	1.426786	3.708972
C	2.343397	-2.726626	2.836506
H	1.594048	-2.754431	2.028696
Fe	-4.793775	-1.411307	-1.122931
Ir	-0.676088	0.147601	0.867797
P	-2.675589	1.101877	0.103313
S	-1.234263	-2.006241	-0.136568
C	-3.523314	0.186021	-1.236212
C	-3.012210	-0.935472	-1.991297
C	-3.985621	-1.266625	-2.984745
H	-3.901368	-2.095226	-3.681557
C	-5.087236	-0.375569	-2.859976
H	-6.001404	-0.404291	-3.443274
C	-4.811405	0.508609	-1.783717
H	-5.473745	1.285309	-1.415415
C	-4.612530	-3.238094	-0.221226
H	-3.753940	-3.893196	-0.328119
C	-4.766868	-2.215688	0.759063
H	-4.048595	-1.960561	1.531464
C	-6.006976	-1.553334	0.516890
H	-6.396502	-0.706312	1.073135
C	-6.618705	-2.169201	-0.613316
H	-7.552734	-1.868478	-1.076054
C	-5.756929	-3.209346	-1.070082
H	-5.922145	-3.837229	-1.939173
C	-1.720223	-1.676357	-1.880056
H	-0.897195	-1.122828	-2.350291
H	-1.817921	-2.650062	-2.377461
C	-2.465947	2.769725	-0.651092
C	-2.132482	3.837885	0.192379
H	-2.055931	3.676223	1.268595
C	-1.898450	5.104834	-0.328209
H	-1.648646	5.926008	0.340569
C	-1.980276	5.321691	-1.703508
H	-1.795138	6.312933	-2.111839
C	-2.299192	4.265379	-2.550080
H	-2.365161	4.426806	-3.624220
C	-2.544938	2.995720	-2.027118
H	-2.799733	2.179695	-2.702725
C	-4.058992	1.433736	1.285168
C	-5.061873	2.371489	1.007056
H	-5.014379	2.971085	0.098523
C	-6.120829	2.558449	1.889977
H	-6.890930	3.292317	1.661127
C	-6.190141	1.815703	3.067208
H	-7.016337	1.966000	3.759005
C	-5.190727	0.892998	3.360812
H	-5.229570	0.318921	4.284375
C	-4.130267	0.708399	2.476680
H	-3.338990	-0.002500	2.713579
C	0.225268	-3.017358	-0.412236
C	0.163534	-4.342815	0.015687
H	-0.757675	-4.728055	0.450322
C	1.282066	-5.165762	-0.106482
H	1.232698	-6.196755	0.237115
C	2.453636	-4.667144	-0.664744
H	3.328066	-5.307452	-0.759806
C	2.509911	-3.343884	-1.103541
H	3.424229	-2.936670	-1.532482
C	1.400379	-2.512672	-0.974831
H	1.452035	-1.471953	-1.303142
H	-0.341364	1.530686	1.650147
H	0.746501	-0.481848	1.426179
H	0.198489	0.760476	-0.553161
H	-1.313836	-0.422279	2.282821
O	4.355617	-0.823676	-1.935632
C	4.924578	-0.974230	-3.230841
H	5.491898	-0.057394	-3.485608
H	3.650917	-0.145893	-1.975869
O	5.393727	0.356825	1.015840
C	6.675806	-0.146950	0.644616
H	6.875274	0.121844	-0.409680
H	5.306332	1.280940	0.703522
C	1.414640	1.503051	-1.542526
O	2.515627	0.988710	-1.189084
C	0.802269	1.058620	-2.841812
C	1.126594	2.916273	-1.126018
H	0.955777	-0.014258	-3.006074
H	1.426458	3.088701	-0.086922
H	1.325076	1.602382	-3.645746
H	-0.262974	1.305404	-2.910106
H	0.076048	3.181263	-1.266202
H	1.737448	3.568338	-1.771486
C	3.834936	-1.174957	-4.264417
H	3.243272	-2.070787	-4.028178
H	3.156137	-0.311202	-4.287144
H	4.257454	-1.295554	-5.269305
C	5.889362	-2.136056	-3.173215
H	6.386879	-2.274750	-4.139973
H	6.662616	-1.967596	-2.413302
H	5.358612	-3.065591	-2.924940
C	4.683708	3.007524	-2.773891
H	3.732115	2.485643	-2.940865
H	4.475073	4.078538	-2.646138
H	5.301285	2.883417	-3.672229
C	6.724668	3.136204	-1.319414
H	7.393601	2.982702	-2.173831
H	6.582631	4.216393	-1.181620
H	7.217611	2.739712	-0.422513
C	6.625906	-1.652688	0.763868
H	6.431646	-1.946690	1.804405
H	5.834695	-2.071750	0.128466
H	7.581470	-2.096090	0.459615
C	7.748764	0.457124	1.527575
H	8.745936	0.105988	1.235302
H	7.741969	1.552943	1.467257
H	7.575977	0.174777	2.575229
C	1.784311	-1.974176	4.023549
H	2.531697	-1.922281	4.828951
H	1.500599	-0.952371	3.741245
H	0.894746	-2.477459	4.421836
C	2.757133	-4.139035	3.190510
H	3.199060	-4.646467	2.324851
H	3.498084	-4.130406	4.002433
H	1.892746	-4.724366	3.527713

C	2.054541	3.467530	3.157163
H	2.011930	3.866114	4.178397
H	2.797413	4.045611	2.589286
H	1.072247	3.619713	2.689710
C	3.785520	1.751401	3.779490
H	4.028639	0.680887	3.772461
H	4.567272	2.286447	3.221798
H	3.803871	2.103974	4.817277

3-Na-iso
E (BS1) = -3416.20377499
E (BS2) = -4556.897162
$G_{298.15,1M}$ (BS2) = -4555.883124

Na	3.271604	-0.105579	0.691734
H	3.830122	2.158119	-0.588153
O	4.683671	2.606544	-0.309566
C	5.548546	2.610344	-1.442904
H	5.781008	1.563641	-1.716006
H	3.842030	-1.703306	3.029516
H	1.438942	1.282619	1.951205
O	2.352024	1.520932	2.213351
O	3.269867	-1.825176	2.258253
C	2.467066	1.284979	3.618706
H	2.240015	0.220900	3.818465
C	2.550135	-3.051332	2.430572
H	1.907241	-3.116484	1.540156
Fe	-4.797328	-1.456152	-1.107974
Ir	-0.736934	0.145288	0.894866
P	-2.739339	1.102783	0.072836
S	-1.176668	-1.967530	-0.272956
C	-3.596769	0.185506	-1.250868
C	-3.066883	-0.905525	-2.035888
C	-4.060558	-1.266056	-2.997868
H	-3.967009	-2.082238	-3.707848
C	-5.190452	-0.419656	-2.825943
H	-6.122476	-0.479042	-3.377699
C	-4.913310	0.465950	-1.751358
H	-5.593724	1.210870	-1.351898
C	-4.530002	-3.280146	-0.220143
H	-3.657652	-3.910718	-0.357596
C	-4.682027	-2.265180	0.768376
H	-3.947897	-1.991576	1.519439
C	-5.948816	-1.639828	0.569545
H	-6.343918	-0.804279	1.139167
C	-6.579305	-2.271561	-0.541382
H	-7.536506	-1.997402	-0.972016
C	-5.702446	-3.283919	-1.030168
H	-5.877815	-3.914875	-1.894924
C	-1.734144	-1.574421	-1.986588
H	-0.953313	-0.954841	-2.448955
H	-1.785419	-2.529073	-2.525553
C	-2.518370	2.763623	-0.687326
C	-2.184156	3.837358	0.148283
H	-2.096693	3.682167	1.224309
C	-1.971774	5.104403	-0.381436
H	-1.723161	5.931690	0.279965
C	-2.069503	5.312313	-1.756758
H	-1.899083	6.303195	-2.172160
C	-2.384345	4.248478	-2.595616
H	-2.461853	4.403633	-3.669790
C	-2.614468	2.980217	-2.063994
H	-2.872033	2.158625	-2.731894
C	-4.087237	1.433850	1.285683
C	-5.093907	2.370812	1.020624
H	-5.064929	2.960836	0.105156
C	-6.132006	2.567984	1.925326
H	-6.906378	3.300161	1.706404
C	-6.174361	1.838026	3.111573
H	-6.983802	1.997110	3.820819
C	-5.170625	0.915936	3.391882
H	-5.189465	0.351429	4.321709
C	-4.132027	0.718243	2.485135
H	-3.344858	0.001059	2.712984
C	0.307170	-2.900718	-0.662468
C	0.246648	-4.283725	-0.489706
H	-0.683548	-4.748461	-0.166031
C	1.378191	-5.062421	-0.722035
H	1.329928	-6.139846	-0.580444
C	2.563210	-4.458655	-1.129085
H	3.450072	-5.063628	-1.306880
C	2.618728	-3.076846	-1.309015
H	3.537796	-2.586305	-1.625306
C	1.492633	-2.290000	-1.077109
H	1.546465	-1.205821	-1.214719
H	-0.548192	1.447433	1.847880
H	0.629384	-0.501336	1.571798
H	0.597528	1.255765	-0.467159
H	-1.459391	-0.524716	2.100104
O	4.356683	-0.484149	-1.955287
C	4.793974	-0.559299	-3.299755
H	5.265072	0.401745	-3.589837
H	3.617734	0.173957	-1.868020
O	5.510919	0.387426	1.128968
C	6.659357	-0.262145	0.581586
H	6.768719	0.042904	-0.474935
H	5.422555	1.285306	0.740448
C	1.337013	1.708203	-1.220172
O	2.584415	1.179834	-1.040852
C	0.779431	1.407255	-2.606449
C	1.295759	3.204007	-0.927924
H	0.834561	0.330254	-2.819796
H	1.627340	3.403309	0.099952
H	1.378376	1.930039	-3.368261
H	-0.265705	1.732693	-2.715013
H	0.288213	3.622270	-1.053880
H	1.975089	3.732943	-1.613013
C	3.626960	-0.819132	-4.232961
H	3.134880	-1.768721	-3.979153
H	2.882432	-0.015441	-4.153854
H	3.956713	-0.870722	-5.278160
C	5.846524	-1.643155	-3.379358
H	6.249126	-1.725038	-4.395861
H	6.680348	-1.427655	-2.698853
H	5.419397	-2.617031	-3.101302
C	4.876644	3.278458	-2.625760
H	3.948679	2.752433	-2.888124
H	4.626488	4.320909	-2.384227
H	5.532881	3.276715	-3.505405
C	6.827310	3.307424	-1.041209
H	7.549318	3.307349	-1.866199
H	6.621714	4.349716	-0.762455
H	7.291425	2.808964	-0.179696
C	6.411181	-1.753049	0.636951
H	6.233013	-2.071262	1.673933
H	5.536534	-2.025873	0.031374
H	7.277671	-2.306229	0.254969
C	7.902526	0.136614	1.350315

H	8.797619	-0.330056	0.921072
H	8.045232	1.224422	1.335751
H	7.817668	-0.181791	2.398305
C	1.685113	-2.987956	3.670985
H	2.309205	-2.847746	4.565689
H	0.973074	-2.155486	3.609511
H	1.118271	-3.918069	3.799273
C	3.511644	-4.220271	2.450796
H	4.138209	-4.227664	1.550381
H	4.170344	-4.158391	3.329304
H	2.970450	-5.173049	2.501410
C	1.492933	2.154541	4.386554
H	1.581217	1.979916	5.465993
H	1.698177	3.215552	4.190318
H	0.456829	1.943947	4.092036
C	3.901889	1.566972	3.999166
H	4.595864	0.942461	3.423747
H	4.144518	2.619233	3.796096
H	4.063679	1.378488	5.066910

$TS_{3\text{-}4}$-Na-iso

E (BS1) = -3416.20137514

E (BS2) = -4556.89719024

$G_{298.15,1M}$ (BS2) = -4555.878320

Na	-3.285959	-0.454966	0.774315
H	-1.981546	-1.675544	-1.293600
O	-2.471468	-2.272654	-0.659186
C	-3.631044	-2.837697	-1.253331
H	-4.491916	-2.153752	-1.091959
H	-4.239403	1.583822	2.818732
H	-1.316039	-0.997759	2.316040
O	-2.108915	-1.182819	2.860809
O	-4.110867	1.488560	1.864424
C	-2.108130	-0.270811	3.959274
H	-2.148200	0.761813	3.564452
C	-3.796465	2.776737	1.318063
H	-3.499627	2.557595	0.282042
Fe	4.832997	1.131065	0.136022
Ir	0.241838	0.020252	0.427890
P	2.210179	-1.136999	-0.163940
S	1.230575	2.225016	-0.056053
C	3.576891	-0.140505	-0.850193
C	3.479296	1.196317	-1.389177
C	4.779268	1.569439	-1.852509
H	5.029920	2.535956	-2.279294
C	5.676861	0.493154	-1.611693
H	6.742855	0.493212	-1.812627
C	4.945605	-0.553326	-0.989064
H	5.356764	-1.496899	-0.645505
C	4.468758	2.680150	1.423173
H	3.762787	3.483057	1.237728
C	4.173003	1.434266	2.048655
H	3.201682	1.124237	2.421722
C	5.362017	0.645088	2.047084
H	5.454636	-0.370883	2.419256
C	6.391892	1.406677	1.421509
H	7.405320	1.071248	1.228099
C	5.839589	2.662934	1.034719
H	6.359877	3.449726	0.498905
C	2.295221	2.087444	-1.559084
H	1.651394	1.744194	-2.379872
H	2.638618	3.103708	-1.790885
C	1.994918	-2.465911	-1.421408
C	1.071502	-3.478618	-1.132209
H	0.509692	-3.446221	-0.198039
C	0.872967	-4.530020	-2.020178
H	0.154864	-5.311708	-1.779169
C	1.590254	-4.579188	-3.214624
H	1.436445	-5.401223	-3.910507
C	2.500047	-3.570658	-3.515648
H	3.059974	-3.600500	-4.448278
C	2.702793	-2.518182	-2.624248
H	3.420517	-1.737150	-2.872058
C	3.045053	-2.089261	1.181224
C	3.928280	-3.136565	0.889889
H	4.099193	-3.436375	-0.143730
C	4.588545	-3.811716	1.911683
H	5.272549	-4.622023	1.667959
C	4.365583	-3.460213	3.241438
H	4.877229	-3.993444	4.039894
C	3.474764	-2.434908	3.543728
H	3.283026	-2.162731	4.579782
C	2.819192	-1.755774	2.519595
H	2.121198	-0.956618	2.761776
C	0.072497	3.457742	-0.668179
C	0.442746	4.802571	-0.600241
H	1.421408	5.079006	-0.208121
C	-0.446497	5.783215	-1.029020
H	-0.160801	6.831486	-0.975233
C	-1.701756	5.423083	-1.516815
H	-2.396675	6.192029	-1.847599
C	-2.065211	4.080568	-1.579268
H	-3.045669	3.790431	-1.957166
C	-1.180995	3.092385	-1.152294
H	-1.462376	2.041007	-1.192677
H	-0.321827	-1.409747	0.918672
H	-1.151408	0.737321	0.963259
H	0.773514	-0.368191	-2.813841
H	0.797292	0.248091	1.867000
O	-3.461253	0.698759	-1.400385
C	-4.566924	0.665599	-2.290874
H	-5.105931	-0.297306	-2.166458
H	-2.636631	0.304001	-1.812570
O	-5.083945	-1.718829	1.724824
C	-6.360508	-1.911473	1.106294
H	-6.232951	-2.555922	0.216935
H	-4.796848	-2.554848	2.117015
C	-0.294125	-0.333726	-3.136444
O	-1.130839	-0.391230	-2.025664
C	-0.459107	0.959483	-3.932102
C	-0.490764	-1.524170	-4.069127
H	-0.339467	1.843098	-3.291656
H	-0.375664	-2.469524	-3.521823
H	-1.458225	1.011981	-4.386001
H	0.280398	1.026431	-4.743263
H	0.236570	-1.515650	-4.893830
H	-1.498317	-1.506375	-4.511710
C	-3.921074	-4.144970	-0.548082
H	-4.843999	-4.600591	-0.927587
H	-3.094337	-4.849874	-0.711691
H	-4.029485	-3.993918	0.533004
C	-3.459015	-3.035915	-2.743469
H	-3.244843	-2.083209	-3.242786
H	-2.624648	-3.723381	-2.942834
H	-4.368903	-3.458662	-3.187010
C	-7.328699	-2.571942	2.062013
H	-6.964019	-3.558833	2.375275

H	-7.465239	-1.951190	2.957661
H	-8.306369	-2.710990	1.585209
C	-6.821944	-0.545532	0.658873
H	-7.009958	0.102571	1.526359
H	-6.055291	-0.070196	0.034219
H	-7.744962	-0.616904	0.072355
C	-5.018627	3.669972	1.317260
H	-4.816974	4.598361	0.766592
H	-5.873324	3.169216	0.846024
H	-5.303429	3.939355	2.344287
C	-2.634018	3.406364	2.055241
H	-2.903691	3.595574	3.104998
H	-1.753508	2.751611	2.034487
H	-2.360904	4.368003	1.601042
C	-0.850764	-0.436707	4.788092
H	-0.844669	0.259619	5.635818
H	-0.785531	-1.460712	5.180079
H	0.045135	-0.241811	4.183412
C	-3.360117	-0.537134	4.762755
H	-4.257915	-0.424139	4.142981
H	-3.344984	-1.561414	5.159784
H	-3.433082	0.156232	5.608677
C	-5.506774	1.794340	-1.920362
H	-6.431399	1.740240	-2.508318
H	-5.774460	1.759191	-0.857521
H	-5.031461	2.766894	-2.115464
C	-4.129526	0.784387	-3.736240
H	-3.603518	1.736994	-3.893868
H	-3.454619	-0.032038	-4.023418
H	-4.998024	0.756943	-4.405886

4-Na-iso

E (BS1) = -3416.22119111

E (BS2) = -4556.916196

$G_{298.15,1M}$ (BS2) = -4555.898979

H	-0.694398	2.446736	2.074655
H	0.925418	-0.679423	-0.282168
H	-0.711319	-0.105605	-1.739672
Na	3.432167	-0.297926	-0.345354
H	1.993197	0.214948	1.905173
O	2.679984	-0.515532	1.991380
C	3.280411	-0.473215	3.279877
H	2.481119	-0.372579	4.037883
H	1.560551	-0.479293	-2.012078
O	2.381329	-0.783584	-2.455779
C	2.250188	-0.495226	-3.847826
H	2.107671	0.594741	-3.972948
O	0.954222	1.392580	1.426421
C	0.384993	2.319072	2.307112
C	1.050301	3.682240	2.166642
C	0.485695	1.845009	3.750834
H	1.014789	4.025088	1.123526
H	2.105403	3.617425	2.473468
H	0.564018	4.440712	2.796023
H	0.007903	2.554045	4.441139
H	0.010817	0.864645	3.880334
H	1.542732	1.753140	4.043722
O	3.283571	1.993873	0.218830
C	3.310526	2.966832	-0.821139
H	2.376932	1.936596	0.629649
H	2.278769	3.135893	-1.176464
Fe	-4.673512	-0.318831	-0.899237
Ir	-0.162855	0.499656	-0.374581
P	-1.644113	-0.934379	0.741063
S	-1.898561	2.240135	-0.733096
C	-3.408011	-0.478038	0.693083
C	-3.927001	0.862073	0.593917
C	-5.351245	0.782938	0.679150
H	-6.025578	1.630720	0.603982
C	-5.720791	-0.582086	0.832601
H	-6.733786	-0.967642	0.877807
C	-4.530830	-1.359531	0.837341
H	-4.478707	-2.441910	0.897571
C	-4.467399	0.620409	-2.707464
H	-4.127135	1.641375	-2.840799
C	-3.638330	-0.537070	-2.652760
H	-2.554864	-0.544980	-2.727321
C	-4.473051	-1.668953	-2.414729
H	-4.136102	-2.692190	-2.277911
C	-5.820093	-1.208965	-2.328516
H	-6.690024	-1.820061	-2.112359
C	-5.815997	0.205597	-2.507276
H	-6.682613	0.856153	-2.457491
C	-3.191064	2.160000	0.565412
H	-2.712147	2.345261	1.537924
H	-3.903726	2.969368	0.365863
C	-1.385511	-1.189620	2.544630
C	-0.168915	-1.749561	2.952009
H	0.584863	-2.006483	2.206202
C	0.093781	-1.970446	4.299078
H	1.037725	-2.424475	4.599169
C	-0.847172	-1.606108	5.261920
H	-0.642101	-1.774263	6.317032
C	-2.047225	-1.023094	4.868116
H	-2.783652	-0.732590	5.614697
C	-2.319827	-0.820407	3.515317
H	-3.271567	-0.378499	3.221400
C	-1.696171	-2.677027	0.129640
C	-2.141233	-3.724414	0.945082
H	-2.434597	-3.527174	1.975937
C	-2.214230	-5.024752	0.453361
H	-2.563431	-5.826893	1.100271
C	-1.838370	-5.298496	-0.860130
H	-1.893507	-6.315560	-1.242573
C	-1.386068	-4.266099	-1.677984
H	-1.080204	-4.470403	-2.702716
C	-1.313957	-2.965637	-1.184756
H	-0.945891	-2.163399	-1.823347
C	-1.270753	3.909498	-0.517794
C	-1.758054	4.819845	0.419188
H	-2.542890	4.547909	1.119473
C	-1.222786	6.106859	0.471235
H	-1.605848	6.809635	1.208318
C	-0.210621	6.491251	-0.400015
H	0.202963	7.495830	-0.350424
C	0.266489	5.580509	-1.342077
H	1.053387	5.869357	-2.036268
C	-0.262455	4.297653	-1.406285
H	0.114239	3.586671	-2.141219
H	0.897402	1.373205	-1.300490
O	5.752992	-0.546320	-0.068447
C	6.434672	-0.846604	-1.297157
H	6.208946	0.011810	-1.949392
H	5.929176	-1.273677	0.546726
O	3.099259	-2.669925	0.319387
C	2.067292	-3.526944	-0.173978

H	1.179091	-2.912374	-0.405860
H	2.748877	-2.172513	1.084484
C	3.879352	4.275783	-0.309186
H	3.266417	4.684169	0.503625
H	4.898063	4.121612	0.073037
H	3.926196	5.025150	-1.109701
C	4.137492	2.407106	-1.961485
H	5.157167	2.185049	-1.612426
H	3.690401	1.482142	-2.354652
H	4.212894	3.122432	-2.789512
C	1.045108	-1.217781	-4.416546
H	0.943260	-1.028564	-5.492212
H	1.145427	-2.301984	-4.261889
H	0.121502	-0.884655	-3.923646
C	3.538515	-0.904132	-4.521893
H	4.398960	-0.390483	-4.073422
H	3.694320	-1.987886	-4.427949
H	3.509748	-0.655524	-5.588963
C	7.926695	-0.926846	-1.062979
H	8.304202	-0.004866	-0.607092
H	8.160367	-1.766289	-0.392918
H	8.461574	-1.090608	-2.005779
C	5.878018	-2.112563	-1.913218
H	6.355721	-2.320666	-2.878664
H	6.062250	-2.970613	-1.250620
H	4.796171	-2.031269	-2.080920
C	2.567836	-4.159152	-1.451541
H	3.463179	-4.766554	-1.257458
H	1.798325	-4.812977	-1.880430
H	2.819064	-3.390939	-2.193308
C	1.691391	-4.561384	0.867746
H	0.881703	-5.206886	0.501634
H	2.556836	-5.194618	1.107721
H	1.346058	-4.086620	1.796601
C	3.995916	-1.787235	3.500274
H	4.448591	-1.823263	4.498005
H	3.305611	-2.636461	3.405951
H	4.797957	-1.914341	2.758644
C	4.223596	0.707791	3.396067
H	4.658544	0.765564	4.401874
H	5.044073	0.611655	2.670796
H	3.702010	1.652613	3.196407

D4. Potassium system

1-K-iso

E (BS1) = -3660.7353789

E (BS2) = -4801.381788

$G_{298.15,1M}$ (BS2) = -4800.451276

K	2.770983	-0.835863	-0.075753
H	1.278609	-2.540805	2.128284
O	2.220112	-2.318029	2.184810
C	2.467308	-1.714810	3.455180
H	2.161555	-2.415986	4.252210
H	3.247602	0.298241	-3.172131
H	1.031998	-2.984559	-1.309469
O	1.858585	-3.350228	-0.958064
O	3.855691	0.328567	-2.418514
C	2.592234	-3.926981	-2.039583
H	2.018884	-4.774296	-2.453530
C	4.504420	1.606811	-2.410260
H	5.192066	1.546901	-1.550884
Fe	-4.936304	1.200338	0.345109
Ir	-0.519443	0.170887	-0.883978
P	-2.274471	-1.025559	0.088974
S	-1.432983	2.340818	-0.207914
C	-3.494461	-0.054875	1.045959
C	-3.290464	1.279131	1.555798
C	-4.463867	1.652412	2.279851
H	-4.620079	2.617147	2.753136
C	-5.387025	0.571207	2.235073
H	-6.387532	0.567418	2.654490
C	-4.796686	-0.476498	1.477789
H	-5.272021	-1.418664	1.226886
C	-4.883939	2.733533	-1.011375
H	-4.178412	3.556813	-0.986302
C	-4.685327	1.482591	-1.665013
H	-3.799063	1.188202	-2.219101
C	-5.826797	0.665469	-1.410036
H	-5.963478	-0.360999	-1.737477
C	-6.730222	1.414268	-0.600320
H	-7.673670	1.057433	-0.200925
C	-6.146719	2.691108	-0.352242
H	-6.571189	3.476419	0.264077
C	-2.064852	2.129700	1.500143
H	-1.266562	1.670849	2.099606
H	-2.285841	3.128048	1.898640
C	-1.699483	-2.226973	1.365604
C	-1.019283	-3.377248	0.948022
H	-0.910371	-3.586575	-0.116859
C	-0.478242	-4.259439	1.877810
H	0.048714	-5.148176	1.535515
C	-0.604086	-3.999402	3.243578
H	-0.178083	-4.686386	3.971828
C	-1.275332	-2.856340	3.667399
H	-1.377105	-2.645597	4.730370
C	-1.822156	-1.975843	2.733929
H	-2.340588	-1.082113	3.082052
C	-3.368742	-2.104643	-0.938023
C	-4.142602	-3.126379	-0.372320
H	-4.069046	-3.341424	0.693508
C	-5.005042	-3.880523	-1.161280
H	-5.601580	-4.669297	-0.707376
C	-5.097719	-3.632574	-2.529744
H	-5.769697	-4.225790	-3.146542
C	-4.317298	-2.634955	-3.105565
H	-4.373414	-2.445387	-4.175657
C	-3.456712	-1.877751	-2.313226
H	-2.837606	-1.100912	-2.761926
C	-0.066745	3.474423	0.059852
C	0.282990	4.267787	-1.035511
H	-0.271101	4.175054	-1.969435
C	1.335567	5.170569	-0.935083
H	1.603068	5.780912	-1.795183
C	2.033492	5.300957	0.263032
H	2.846477	6.018933	0.348815
C	1.679671	4.512413	1.354774
H	2.212965	4.618690	2.298324
C	0.639409	3.587853	1.258103
H	0.392454	2.971506	2.120353
H	0.148463	-1.217504	-1.378532
H	0.832509	0.896904	-1.507519
H	0.333452	0.063061	0.580367
H	-1.207103	0.320894	-2.433292
O	3.610849	1.478982	1.068225
C	4.759770	1.974345	1.760742
H	5.484386	1.144842	1.718857

H	2.936937	2.177697	1.070611
O	5.383934	-1.186367	-0.656206
C	6.768900	-1.514917	-0.598433
H	6.995022	-2.266014	-1.376737
H	5.176634	-0.804633	-1.527381
C	2.830330	-2.905739	-3.133495
H	3.318531	-3.367764	-4.000588
H	1.886801	-2.459851	-3.475477
H	3.485785	-2.101173	-2.770033
C	3.890183	-4.437697	-1.457619
H	4.473956	-3.596874	-1.054062
H	3.705408	-5.153710	-0.648509
H	4.490639	-4.934373	-2.228543
C	3.503647	2.716742	-2.177785
H	2.770560	2.748977	-2.997131
H	2.963572	2.563854	-1.234829
H	4.007324	3.691385	-2.135446
C	5.300883	1.799115	-3.682427
H	5.999804	0.968734	-3.840054
H	4.627076	1.849771	-4.549426
H	5.875530	2.732296	-3.647657
C	7.020710	-2.126166	0.760273
H	6.371137	-2.993897	0.927796
H	8.062818	-2.453124	0.851008
H	6.823108	-1.389384	1.551582
C	7.614585	-0.280120	-0.834309
H	7.402814	0.473539	-0.061955
H	8.684159	-0.520246	-0.797388
H	7.402596	0.164336	-1.816044
C	3.960916	-1.496014	3.537706
H	4.239473	-1.061574	4.504914
H	4.500994	-2.442211	3.416728
H	4.288362	-0.804351	2.746627
C	5.340300	3.180327	1.050702
H	4.606180	3.999308	1.028675
H	6.236948	3.543050	1.567592
H	5.615501	2.941454	0.016531
C	4.420408	2.277421	3.205791
H	4.057087	1.383336	3.725080
H	5.296617	2.656818	3.745106
H	3.633927	3.044863	3.255402
C	1.684413	-0.425381	3.603248
H	2.003289	0.307469	2.847160
H	0.607902	-0.606321	3.476901
H	1.839605	0.014574	4.596875

2-K-iso

E (BS1) = -3853.78961951

E (BS2) = -4994.509371

$G_{298.15,1M}$ (BS2) = -4993.503306

K	3.201953	-0.466253	0.590617
H	4.074588	2.374329	0.010232
O	4.967648	2.571842	0.351805
C	5.841763	2.732030	-0.771302
H	5.992606	1.743292	-1.242491
H	3.453025	-2.306847	3.508661
H	1.251326	1.398186	1.769727
O	2.154191	1.638577	2.066755
O	3.246507	-2.245495	2.565927
C	2.291936	1.190275	3.415391
H	2.015395	0.119042	3.469716
C	2.515395	-3.416571	2.190869
H	2.211140	-3.232338	1.147745
Fe	-4.953625	-1.389584	-1.190526
Ir	-0.912238	0.050063	0.887330
P	-2.882016	1.072598	0.168134
S	-1.333729	-1.972602	-0.418821
C	-3.755757	0.259840	-1.219912
C	-3.225537	-0.770060	-2.081213
C	-4.219679	-1.066909	-3.064610
H	-4.123975	-1.829308	-3.832022
C	-5.352088	-0.238522	-2.831053
H	-6.285578	-0.261894	-3.383240
C	-5.072667	0.572153	-1.698379
H	-5.756553	1.281578	-1.244467
C	-4.675835	-3.260481	-0.409794
H	-3.799837	-3.876524	-0.583571
C	-4.830703	-2.303570	0.634897
H	-4.093180	-2.064980	1.394858
C	-6.101384	-1.674910	0.473322
H	-6.499335	-0.874178	1.089615
C	-6.730780	-2.246004	-0.671042
H	-7.690625	-1.953450	-1.083403
C	-5.849492	-3.224509	-1.217433
H	-6.023767	-3.807657	-2.115433
C	-1.880261	-1.414927	-2.086758
H	-1.114715	-0.722218	-2.463482
H	-1.903457	-2.303373	-2.730852
C	-2.599041	2.757334	-0.526435
C	-2.312125	3.813435	0.347351
H	-2.321926	3.643953	1.424655
C	-2.018990	5.080700	-0.144950
H	-1.807254	5.892849	0.547696
C	-1.992715	5.308620	-1.520084
H	-1.762313	6.299639	-1.905482
C	-2.259973	4.261649	-2.396507
H	-2.239698	4.429932	-3.471668
C	-2.565532	2.993660	-1.902783
H	-2.774631	2.183221	-2.601819
C	-4.266185	1.401042	1.348784
C	-5.249283	2.366574	1.096169
H	-5.184428	2.994383	0.207984
C	-6.310600	2.543169	1.978244
H	-7.066369	3.297671	1.769446
C	-6.400403	1.763305	3.129988
H	-7.228521	1.905648	3.821232
C	-5.419615	0.813623	3.399280
H	-5.475058	0.210568	4.303369
C	-4.357905	0.637647	2.514636
H	-3.580627	-0.096508	2.727714
C	0.194693	-2.792538	-0.895112
C	0.290830	-4.161225	-0.646211
H	-0.554462	-4.690414	-0.208465
C	1.467282	-4.844597	-0.951566
H	1.538436	-5.911180	-0.749107
C	2.542938	-4.163070	-1.511041
H	3.466248	-4.690812	-1.743515
C	2.437860	-2.797147	-1.777546
H	3.276859	-2.250972	-2.206035
C	1.268618	-2.107066	-1.467579
H	1.192229	-1.034700	-1.660789
H	-0.652682	1.358655	1.816344
H	0.544412	-0.569957	1.384992
H	-0.108517	0.828222	-0.397114
H	-1.555070	-0.716151	2.259485
O	4.485475	-0.183688	-1.939583
C	5.137742	-0.179193	-3.208016

H	5.843247	0.674038	-3.243576
H	3.854206	0.559159	-1.926440
O	5.760972	-0.010330	1.248314
C	6.788258	-0.661167	0.503035
H	6.812404	-0.241111	-0.519559
H	5.748040	0.945458	1.049291
C	1.769974	2.399984	-1.494609
O	2.678631	1.687971	-1.058369
C	1.094764	2.075992	-2.781922
C	1.367627	3.647100	-0.791562
H	1.261836	1.031851	-3.065184
H	2.076837	3.897457	0.002722
H	1.527600	2.729401	-3.554685
H	0.022693	2.302303	-2.737564
H	0.368929	3.494871	-0.354589
H	1.276950	4.474685	-1.506403
C	4.120651	-0.014411	-4.318380
H	3.388142	-0.833890	-4.294328
H	3.578009	0.935326	-4.211556
H	4.605730	-0.013682	-5.301849
C	5.927744	-1.461827	-3.326492
H	6.490653	-1.479099	-4.266727
H	6.641778	-1.560773	-2.499637
H	5.260020	-2.334735	-3.312094
C	5.252905	3.678177	-1.797326
H	4.324257	3.280786	-2.227358
H	5.032769	4.651158	-1.337194
H	5.959328	3.838707	-2.621076
C	7.159841	3.231529	-0.228687
H	7.905964	3.305919	-1.027695
H	7.033903	4.224702	0.222407
H	7.548668	2.553990	0.542332
C	6.419380	-2.127043	0.424063
H	6.310778	-2.540230	1.436423
H	5.476198	-2.279908	-0.119528
H	7.198656	-2.698975	-0.093888
C	8.135494	-0.459548	1.167540
H	8.934518	-0.942662	0.591753
H	8.379499	0.606218	1.257484
H	8.126605	-0.893370	2.176521
C	1.273654	-3.572682	3.040272
H	1.547113	-3.737348	4.092587
H	0.643983	-2.675587	2.979773
H	0.681014	-4.434256	2.707974
C	3.408954	-4.637893	2.233725
H	4.284836	-4.507924	1.585938
H	3.760404	-4.816435	3.259817
H	2.866129	-5.532350	1.903728
C	1.386528	1.979712	4.339054
H	1.489179	1.637128	5.376323
H	1.645894	3.046466	4.299956
H	0.334543	1.869039	4.046779
C	3.750304	1.336908	3.786865
H	4.398082	0.739149	3.132218
H	4.057112	2.388633	3.703147
H	3.920341	1.011435	4.820032

TS_{2-3}-K-iso

E (BS1) = -3853.77887267

E (BS2) = -4994.498469

$G_{298.15,1M}$ (BS2) = -4993.488978

K	3.403954	-0.094739	0.729570
H	3.588648	2.403228	-0.327578
O	4.427803	2.701387	0.078983
C	5.376839	2.850334	-0.974249
H	5.707834	1.847155	-1.304731
H	3.621725	-2.005269	3.556417
H	1.255301	1.529719	1.982053
O	2.126099	1.801678	2.340302
O	3.448671	-1.998484	2.604700
C	2.256776	1.208229	3.630872
H	2.063810	0.120438	3.549877
C	2.685838	-3.165618	2.281435
H	2.441929	-3.046181	1.213903
Fe	-4.781084	-1.566491	-1.171931
Ir	-0.775024	0.214731	0.822112
P	-2.851021	1.051008	0.131101
S	-1.145316	-1.907158	-0.329185
C	-3.662029	0.140017	-1.233846
C	-3.077851	-0.888296	-2.063703
C	-4.049455	-1.254943	-3.046200
H	-3.913872	-2.034766	-3.789689
C	-5.221057	-0.474890	-2.842586
H	-6.147553	-0.555634	-3.401046
C	-4.989860	0.374409	-1.728294
H	-5.707610	1.066281	-1.300121
C	-4.413706	-3.411893	-0.369727
H	-3.506931	-3.984391	-0.536331
C	-4.619871	-2.453722	0.664712
H	-3.899155	-2.172844	1.426005
C	-5.919575	-1.890762	0.494021
H	-6.358790	-1.106402	1.102993
C	-6.516014	-2.503469	-0.646000
H	-7.487872	-2.263394	-1.064063
C	-5.585446	-3.442411	-1.180432
H	-5.727226	-4.041272	-2.073803
C	-1.719528	-1.505663	-2.031869
H	-0.965803	-0.843907	-2.479441
H	-1.736665	-2.448233	-2.594357
C	-2.782798	2.760170	-0.553633
C	-2.547464	3.822055	0.328987
H	-2.460272	3.628957	1.398923
C	-2.424651	5.122797	-0.145193
H	-2.251272	5.938249	0.554038
C	-2.517625	5.380387	-1.512515
H	-2.418072	6.397904	-1.884459
C	-2.738520	4.330973	-2.398177
H	-2.813671	4.523887	-3.466551
C	-2.876896	3.027622	-1.921385
H	-3.060281	2.216907	-2.626393
C	-4.228626	1.228464	1.352005
C	-5.306834	2.093293	1.123738
H	-5.323239	2.726768	0.237534
C	-6.361669	2.165167	2.027794
H	-7.191133	2.842891	1.836121
C	-6.351093	1.379803	3.179036
H	-7.174168	1.440271	3.888086
C	-5.277097	0.529808	3.424286
H	-5.254411	-0.076894	4.327355
C	-4.221710	0.458588	2.517425
H	-3.374647	-0.198433	2.714469
C	0.379494	-2.761253	-0.752380
C	0.418093	-4.137440	-0.527574
H	-0.461580	-4.644277	-0.132967
C	1.581330	-4.855907	-0.798467
H	1.608600	-5.927597	-0.613237
C	2.700672	-4.200498	-1.300279

H	3.613398	-4.756369	-1.506668
C	2.655550	-2.826568	-1.537353
H	3.526605	-2.297470	-1.920288
C	1.499547	-2.099569	-1.260756
H	1.477503	-1.019629	-1.427326
H	-0.587397	1.562832	1.705382
H	0.698954	-0.333859	1.348503
H	0.049515	1.016375	-0.538258
H	-1.365912	-0.509430	2.182381
O	4.368284	-0.162257	-2.015738
C	4.919531	-0.122519	-3.327356
H	5.482839	0.823123	-3.454241
H	3.592110	0.434460	-1.995780
O	6.034486	0.135804	1.220126
C	6.895729	-0.535947	0.297701
H	6.718765	-0.130811	-0.715400
H	6.174270	1.089977	1.139831
C	1.194263	1.920118	-1.446974
O	2.325259	1.406193	-1.211711
C	0.549620	1.640413	-2.777231
C	0.884887	3.250979	-0.821958
H	0.732536	0.606719	-3.092415
H	1.240735	3.287264	0.213173
H	1.028005	2.309053	-3.511848
H	-0.525224	1.851955	-2.778203
H	-0.179755	3.497116	-0.859049
H	1.435347	4.010740	-1.400621
C	3.810195	-0.170228	-4.358647
H	3.224863	-1.094301	-4.248080
H	3.128509	0.683111	-4.237170
H	4.212695	-0.135941	-5.378172
C	5.886288	-1.276478	-3.465194
H	6.369839	-1.256575	-4.448806
H	6.671419	-1.225777	-2.700488
H	5.363569	-2.237357	-3.360471
C	4.758679	3.570210	-2.157218
H	3.896477	3.013309	-2.550752
H	4.413632	4.568927	-1.855424
H	5.486752	3.689165	-2.969355
C	6.563176	3.603019	-0.415822
H	7.353850	3.696470	-1.169326
H	6.261394	4.611743	-0.103182
H	6.986355	3.091859	0.459082
C	6.504007	-1.996409	0.321984
H	6.621485	-2.400022	1.336803
H	5.460645	-2.148194	0.008562
H	7.141891	-2.578816	-0.353112
C	8.347551	-0.329360	0.674433
H	9.010297	-0.830831	-0.041454
H	8.605682	0.737006	0.682058
H	8.544224	-0.738849	1.674161
C	1.402834	-3.208306	3.082395
H	1.625382	-3.267789	4.157955
H	0.794693	-2.312217	2.900334
H	0.805797	-4.089249	2.814465
C	3.523907	-4.413998	2.458286
H	4.431735	-4.368707	1.844090
H	3.822178	-4.527784	3.510234
H	2.958791	-5.309117	2.170283
C	1.261872	1.814486	4.599598
H	1.355360	1.363700	5.595517
H	1.435206	2.895265	4.690891
H	0.233173	1.658643	4.250051
C	3.686891	1.417681	4.075537
H	4.401961	0.960251	3.378292
H	3.911950	2.491303	4.132745
H	3.854570	0.977656	5.065625

3-K-iso

E (BS1) = -3853.79651224

E (BS2) = -4994.516918

$G_{298.15,1M}$ (BS2) = -4993.500851

K	3.228877	-0.326485	0.766824
H	3.738241	2.230283	-0.397770
O	4.591960	2.596413	-0.020784
C	5.457773	2.887953	-1.113819
H	5.789961	1.936615	-1.572927
H	3.565670	-2.400333	3.519151
H	1.256994	1.384637	2.148361
O	2.145200	1.580331	2.508462
O	3.382542	-2.287055	2.576139
C	2.261023	0.875428	3.743887
H	2.031865	-0.194155	3.569540
C	2.681195	-3.445464	2.115143
H	2.432337	-3.217375	1.065960
Fe	-4.796687	-1.430119	-1.244546
Ir	-0.773983	0.147713	0.863823
P	-2.826211	1.092511	0.153046
S	-1.169468	-1.876240	-0.460818
C	-3.659123	0.261390	-1.243278
C	-3.094027	-0.734182	-2.124626
C	-4.079611	-1.044202	-3.112302
H	-3.960582	-1.789600	-3.892859
C	-5.239273	-0.259983	-2.861269
H	-6.171846	-0.304752	-3.413435
C	-4.988243	0.535721	-1.712383
H	-5.692774	1.216449	-1.245964
C	-4.452284	-3.313334	-0.523234
H	-3.555306	-3.891769	-0.719265
C	-4.639428	-2.396885	0.551756
H	-3.912224	-2.160416	1.321767
C	-5.931357	-1.808072	0.411896
H	-6.356517	-1.043749	1.055303
C	-6.542041	-2.363588	-0.749781
H	-7.511614	-2.090918	-1.152767
C	-5.628077	-3.292518	-1.328090
H	-5.782680	-3.850464	-2.245429
C	-1.737508	-1.355552	-2.135317
H	-0.980865	-0.668912	-2.539500
H	-1.755114	-2.259971	-2.756657
C	-2.691751	2.822113	-0.458863
C	-2.413829	3.838157	0.464616
H	-2.326875	3.599637	1.525416
C	-2.253299	5.152173	0.040752
H	-2.048261	5.933442	0.769611
C	-2.347510	5.465997	-1.314492
H	-2.216870	6.493645	-1.646648
C	-2.608354	4.460973	-2.239837
H	-2.683415	4.698669	-3.298992
C	-2.786620	3.145223	-1.814457
H	-3.001630	2.369885	-2.549436
C	-4.173596	1.247786	1.400594
C	-5.230076	2.150297	1.223735
H	-5.239739	2.820410	0.364855
C	-6.270575	2.210517	2.144993
H	-7.083722	2.917491	1.994301
C	-6.266281	1.375919	3.260843

H	-7.078250	1.427488	3.983108
C	-5.213752	0.486670	3.454312
H	-5.196582	-0.159528	4.329465
C	-4.172743	0.426159	2.530830
H	-3.348077	-0.266819	2.690902
C	0.336889	-2.739269	-0.920645
C	0.337675	-4.127617	-0.783505
H	-0.560120	-4.637161	-0.436225
C	1.488435	-4.854657	-1.081377
H	1.487512	-5.936416	-0.966084
C	2.632231	-4.195271	-1.519471
H	3.534761	-4.759734	-1.745407
C	2.625069	-2.808309	-1.666255
H	3.514308	-2.272277	-1.995012
C	1.480091	-2.072288	-1.366265
H	1.489811	-0.982969	-1.468361
H	-0.605817	1.384328	1.900338
H	0.611589	-0.506121	1.476440
H	0.499036	1.256568	-0.471263
H	-1.467630	-0.634921	2.024724
O	4.350851	-0.145644	-2.001365
C	4.812693	-0.051018	-3.336725
H	5.279039	0.942572	-3.497440
H	3.577469	0.465989	-1.874656
O	5.806526	0.386072	1.237237
C	6.734307	-0.276181	0.376815
H	6.591668	0.094374	-0.652717
H	5.566081	1.255679	0.855318
C	1.211758	1.870869	-1.148767
O	2.479540	1.394128	-1.061299
C	0.639066	1.750405	-2.557495
C	1.094455	3.299783	-0.630423
H	0.745514	0.722407	-2.932160
H	1.465481	3.365161	0.401226
H	1.195590	2.413233	-3.238176
H	-0.423597	2.031102	-2.602165
H	0.060135	3.668095	-0.651148
H	1.709791	3.966297	-1.253312
C	3.661341	-0.197359	-4.313492
H	3.177393	-1.177402	-4.196643
H	2.906331	0.580900	-4.137877
H	4.005801	-0.104858	-5.351108
C	5.875455	-1.109144	-3.535426
H	6.287644	-1.063873	-4.550430
H	6.701809	-0.972625	-2.826066
H	5.454047	-2.112796	-3.381323
C	4.735187	3.698316	-2.171216
H	3.854064	3.152479	-2.534415
H	4.399695	4.658285	-1.754473
H	5.392250	3.903552	-3.025892
C	6.663587	3.612348	-0.560936
H	7.396925	3.813178	-1.350751
H	6.361081	4.570793	-0.117739
H	7.154787	3.017172	0.220193
C	6.424882	-1.759210	0.405147
H	6.463506	-2.133727	1.437497
H	5.429193	-1.976411	-0.008127
H	7.156634	-2.322201	-0.187157
C	8.154243	0.006427	0.825511
H	8.881762	-0.481731	0.165036
H	8.361504	1.083591	0.823233
H	8.310311	-0.369142	1.846018
C	1.399231	-3.643093	2.894470
H	1.621068	-3.828751	3.955433
H	0.757426	-2.754812	2.824338
H	0.838835	-4.505563	2.512492
C	3.583927	-4.659931	2.150718
H	4.487652	-4.490678	1.551973
H	3.887974	-4.878821	3.184097
H	3.069653	-5.545178	1.756720
C	1.287949	1.424821	4.766916
H	1.373773	0.887570	5.719726
H	1.492538	2.488278	4.949523
H	0.252880	1.329021	4.414150
C	3.698892	1.001425	4.193234
H	4.394435	0.625278	3.431211
H	3.944818	2.055438	4.380954
H	3.867100	0.439089	5.119327

$TS_{3\text{-}4}$-K-iso

E (BS1) = -3853.79259858

E (BS2) = -4994.515366

$G_{298.15,1M}$ (BS2) = -4993.498911

K	-3.311665	-0.429018	1.100298
H	-1.870283	-1.802947	-1.146965
O	-2.227837	-2.480183	-0.498464
C	-3.488101	-2.992810	-0.877105
H	-4.276886	-2.219412	-0.735144
H	-4.427775	2.339023	2.877076
H	-0.934075	-0.704704	2.668875
O	-1.645878	-0.864550	3.321636
O	-4.236265	1.934184	2.019562
C	-1.852011	0.344450	4.051481
H	-2.035749	1.169957	3.336849
C	-3.843789	2.976808	1.117386
H	-3.550386	2.445674	0.196803
Fe	4.942739	1.099189	-0.084981
Ir	0.395100	0.093780	0.475441
P	2.306973	-1.156875	-0.101508
S	1.367246	2.230400	-0.252106
C	3.656823	-0.245220	-0.925009
C	3.546553	1.043394	-1.570811
C	4.833119	1.370472	-2.101141
H	5.072851	2.296688	-2.614761
C	5.735019	0.314236	-1.796986
H	6.794798	0.292573	-2.027518
C	5.019803	-0.674124	-1.069535
H	5.440709	-1.585838	-0.658974
C	4.647784	2.773156	1.055299
H	3.968207	3.579463	0.799642
C	4.315636	1.602309	1.796860
H	3.337430	1.362938	2.203072
C	5.476468	0.774618	1.859567
H	5.537501	-0.204710	2.325194
C	6.525305	1.437517	1.158234
H	7.524360	1.049496	0.989917
C	6.012957	2.671032	0.659784
H	6.554642	3.385503	0.049025
C	2.358907	1.921622	-1.780268
H	1.674017	1.493830	-2.524592
H	2.692722	2.903362	-2.139786
C	2.016646	-2.589696	-1.221659
C	1.098230	-3.554752	-0.791254
H	0.593213	-3.430137	0.167142
C	0.830622	-4.674374	-1.570473
H	0.113975	-5.415712	-1.221637
C	1.476852	-4.842110	-2.794296

H	1.269616	-5.718027	-3.405497
C	2.384442	-3.883382	-3.233735
H	2.889731	-4.007593	-4.189534
C	2.654378	-2.760445	-2.452515
H	3.368371	-2.017903	-2.807386
C	3.174329	-2.002892	1.294471
C	4.069960	-3.053549	1.056918
H	4.227904	-3.417481	0.042014
C	4.757725	-3.651389	2.107988
H	5.451799	-4.464370	1.905053
C	4.549612	-3.218909	3.416159
H	5.083595	-3.690714	4.238264
C	3.643362	-2.193640	3.667224
H	3.461158	-1.861101	4.687140
C	2.959458	-1.591832	2.613290
H	2.245774	-0.796054	2.819863
C	0.142873	3.345736	-0.958265
C	0.442900	4.707491	-1.023539
H	1.407563	5.070747	-0.669622
C	-0.499440	5.594369	-1.537277
H	-0.269610	6.656495	-1.587122
C	-1.734380	5.123281	-1.980582
H	-2.469222	5.819668	-2.379499
C	-2.026426	3.763115	-1.912058
H	-2.988036	3.381822	-2.256753
C	-1.091165	2.869766	-1.395911
H	-1.309238	1.802886	-1.347291
H	-0.201697	-1.263189	1.113569
H	-0.959521	0.885381	1.019078
H	0.715342	-0.633797	-2.748596
H	1.032912	0.442638	1.849986
O	-3.525458	0.602434	-1.499405
C	-4.638605	0.394280	-2.349162
H	-5.163875	-0.538095	-2.046276
H	-2.690788	0.160532	-1.837010
O	-5.552852	-1.855947	1.846195
C	-6.654520	-1.796506	0.936307
H	-6.376125	-2.317685	0.000227
H	-5.448071	-2.773594	2.130676
C	-0.362511	-0.592810	-3.037473
O	-1.162034	-0.484419	-1.902185
C	-0.511620	0.602450	-3.976110
C	-0.636817	-1.877803	-3.812448
H	-0.268959	1.547049	-3.473418
H	-0.548822	-2.752511	-3.153888
H	-1.546740	0.679617	-4.338288
H	0.145760	0.508213	-4.852549
H	0.066584	-2.005623	-4.647983
H	-1.654194	-1.865856	-4.232115
C	-3.792586	-4.151822	0.049276
H	-4.807408	-4.536948	-0.111450
H	-3.080449	-4.969145	-0.129383
H	-3.694650	-3.849035	1.099621
C	-3.522767	-3.414707	-2.331184
H	-3.321291	-2.561882	-2.990794
H	-2.761251	-4.184269	-2.522083
H	-4.506708	-3.824396	-2.594401
C	-7.875573	-2.462901	1.530255
H	-7.677343	-3.518621	1.755345
H	-8.169044	-1.958807	2.460491
H	-8.719792	-2.420750	0.831894
C	-6.883638	-0.335682	0.630912
H	-7.194795	0.204018	1.535635
H	-5.968918	0.136850	0.250312
H	-7.660931	-0.215473	-0.133170
C	-5.014786	3.889303	0.818259
H	-4.759821	4.602418	0.023383
H	-5.891075	3.312470	0.497057
H	-5.293087	4.464388	1.713061
C	-2.653446	3.736175	1.663485
H	-2.916400	4.221046	2.615326
H	-1.802361	3.064637	1.838110
H	-2.335333	4.519140	0.962557
C	-0.631949	0.677350	4.885063
H	-0.779246	1.608378	5.446416
H	-0.429079	-0.130973	5.600432
H	0.253237	0.802237	4.246654
C	-3.093675	0.151354	4.891572
H	-3.963089	-0.092042	4.266370
H	-2.947370	-0.669961	5.605898
H	-3.324485	1.061888	5.457063
C	-5.589032	1.560684	-2.168460
H	-6.530503	1.387452	-2.704891
H	-5.823597	1.721738	-1.109579
H	-5.135745	2.484165	-2.557349
C	-4.226944	0.260394	-3.801159
H	-3.740602	1.186246	-4.140445
H	-3.523389	-0.569433	-3.947229
H	-5.102049	0.079990	-4.437895

4-K-iso

E (BS1) = -3853.81272054

E (BS2) = -4994.534447

$G_{298.15,1M}$ (BS2) = -4993.519493

H	-0.983589	2.320377	2.083961
H	1.013245	-0.590666	-0.147419
H	-0.586126	-0.197101	-1.696845
K	3.629283	0.067361	-0.362145
H	1.870544	0.406375	2.027694
O	2.568163	-0.297405	2.190206
C	3.169982	-0.125520	3.464990
H	2.375529	-0.029263	4.228810
H	1.446507	-0.615247	-2.056441
O	2.239085	-0.918913	-2.545591
C	1.981680	-0.775204	-3.939161
H	1.727027	0.280117	-4.151783
O	0.823950	1.559744	1.446871
C	0.099658	2.374717	2.326076
C	0.526897	3.832624	2.205578
C	0.267129	1.914957	3.768678
H	0.461899	4.178540	1.165960
H	1.568717	3.946348	2.541172
H	-0.099672	4.490440	2.824761
H	-0.381658	2.482078	4.451090
H	0.028520	0.850293	3.868643
H	1.307739	2.064707	4.094430
O	3.097669	2.587805	0.462298
C	2.868664	3.622571	-0.485001
H	2.220607	2.286928	0.830143
H	1.781395	3.759204	-0.598235
Fe	-4.620061	-0.588102	-0.952148
Ir	-0.136273	0.507212	-0.338549
P	-1.596209	-0.955080	0.784601
S	-1.967198	2.104409	-0.880881
C	-3.380042	-0.584347	0.668090
C	-3.967835	0.717127	0.475417
C	-5.387545	0.566788	0.542716

H	-6.103871	1.370578	0.401375
C	-5.687591	-0.804122	0.775020
H	-6.679388	-1.240403	0.828123
C	-4.458647	-1.513759	0.848257
H	-4.351216	-2.585915	0.977077
C	-4.402469	0.257971	-2.802968
H	-4.096031	1.282927	-2.981129
C	-3.532495	-0.861882	-2.662442
H	-2.447805	-0.830231	-2.702071
C	-4.331287	-2.010893	-2.385413
H	-3.962177	-3.012460	-2.185995
C	-5.696007	-1.599041	-2.357987
H	-6.548236	-2.231137	-2.132024
C	-5.739644	-0.197179	-2.614128
H	-6.630915	0.421127	-2.621868
C	-3.309116	2.051479	0.366036
H	-2.890788	2.348498	1.338208
H	-4.058080	2.795344	0.067012
C	-1.395828	-1.133331	2.606560
C	-0.222607	-1.727375	3.087470
H	0.532015	-2.078384	2.382763
C	-0.009516	-1.869011	4.453872
H	0.901050	-2.347055	4.813733
C	-0.959255	-1.398529	5.360793
H	-0.792956	-1.506845	6.430472
C	-2.118162	-0.788006	4.892410
H	-2.861688	-0.416441	5.594720
C	-2.339199	-0.659132	3.521606
H	-3.257884	-0.190149	3.169085
C	-1.560066	-2.720591	0.239178
C	-1.973045	-3.756792	1.085243
H	-2.290577	-3.536684	2.103958
C	-1.983885	-5.075876	0.639929
H	-2.307920	-5.868614	1.311086
C	-1.579811	-5.379391	-0.658259
H	-1.587275	-6.410446	-1.005444
C	-1.160704	-4.357529	-1.506448
H	-0.834364	-4.586361	-2.519581
C	-1.146050	-3.039141	-1.058460
H	-0.794815	-2.246506	-1.718134
C	-1.480467	3.830566	-0.809988
C	-2.037529	4.770634	0.056579
H	-2.799120	4.492865	0.780008
C	-1.598708	6.093519	0.011931
H	-2.033719	6.819170	0.696113
C	-0.612522	6.484452	-0.886407
H	-0.271572	7.516838	-0.911279
C	-0.069069	5.544209	-1.760655
H	0.701312	5.836198	-2.472466
C	-0.502089	4.224542	-1.728571
H	-0.064287	3.490389	-2.405728
H	0.944747	1.373144	-1.243987
O	6.233560	-0.672678	-0.379861
C	6.505376	-1.130100	-1.711028
H	6.205554	-0.288862	-2.356741
H	6.486715	-1.381157	0.229322
O	3.157442	-2.529990	0.636288
C	2.127605	-3.466986	0.323697
H	1.178005	-2.916785	0.197260
H	2.818925	-1.935125	1.334695
C	3.471101	4.922257	0.012630
H	3.035580	5.214496	0.976067
H	4.556464	4.812508	0.147957
H	3.299385	5.738132	-0.701513
C	3.443720	3.225211	-1.831830
H	4.521563	3.020240	-1.744048
H	2.943259	2.331716	-2.230177
H	3.315170	4.029361	-2.568322
C	0.819877	-1.653889	-4.358853
H	0.623775	-1.561438	-5.434342
H	1.043764	-2.707207	-4.137002
H	-0.097129	-1.375549	-3.822041
C	3.256444	-1.135305	-4.665635
H	4.089299	-0.500137	-4.337642
H	3.519578	-2.183779	-4.466171
H	3.136566	-1.012181	-5.748091
C	7.985799	-1.392293	-1.885430
H	8.574336	-0.505704	-1.624333
H	8.301110	-2.221319	-1.236173
H	8.216516	-1.668724	-2.920855
C	5.665828	-2.348512	-2.038427
H	5.873208	-2.702206	-3.055801
H	5.904475	-3.164765	-1.341443
H	4.589702	-2.138791	-1.965668
C	2.477916	-4.132349	-0.988034
H	3.448781	-4.642487	-0.912075
H	1.720836	-4.882071	-1.249874
H	2.528898	-3.395639	-1.800983
C	1.963502	-4.475646	1.444780
H	1.145512	-5.174855	1.224298
H	2.887684	-5.055349	1.577495
H	1.732568	-3.980059	2.398020
C	3.981734	-1.367094	3.760712
H	4.446657	-1.302948	4.751444
H	3.354643	-2.268069	3.732348
H	4.782178	-1.483616	3.015438
C	4.029717	1.123124	3.482770
H	4.457884	1.297070	4.478134
H	4.857077	1.019922	2.764852
H	3.445254	2.007902	3.198887

5-K-iso

E (BS1) = -3854.98556587

E (BS2) = -4995.709547

$G_{298.15,1M}$ (BS2) = -4994.689449

H	-1.048044	2.318307	2.026058
H	1.027854	-0.556524	-0.134435
H	-0.583510	-0.263600	-1.714152
K	3.698215	0.072819	-0.311409
H	1.848330	0.464426	2.022977
O	2.566720	-0.210452	2.213227
C	3.160498	0.032802	3.480708
H	2.361145	0.145371	4.237046
H	1.518204	-0.602736	-2.013730
O	2.321663	-0.901156	-2.488221
C	2.062915	-0.824331	-3.887017
H	1.755708	0.207643	-4.139901
O	0.772679	1.574227	1.412722
C	0.036648	2.429441	2.242851
C	0.410010	3.889145	2.010678
C	0.246075	2.085841	3.711625
H	0.335940	4.154095	0.948046
H	1.445482	4.066924	2.338428
H	-0.243628	4.567056	2.579155
H	-0.378038	2.710890	4.366257
H	0.008918	1.034652	3.910866
H	1.296420	2.258575	3.991544

O	3.026395	2.592374	0.370385
C	2.801854	3.567821	-0.638832
H	2.150694	2.299678	0.749140
H	1.715997	3.662666	-0.800010
Fe	-4.585118	-0.758183	-1.013362
Ir	-0.160150	0.492985	-0.376216
P	-1.589114	-0.963220	0.789984
S	-2.027299	2.028741	-0.993953
C	-3.381719	-0.656304	0.630636
C	-4.005466	0.617464	0.378171
C	-5.421127	0.424351	0.421742
H	-6.158696	1.199586	0.237110
C	-5.682931	-0.946133	0.698067
H	-6.661318	-1.412195	0.746555
C	-4.434148	-1.612779	0.823078
H	-4.296184	-2.675683	0.992557
C	-4.344449	0.024742	-2.889347
H	-4.057485	1.049593	-3.099015
C	-3.452108	-1.067928	-2.687663
H	-2.367902	-1.011933	-2.705337
C	-4.230004	-2.224985	-2.385945
H	-3.841653	-3.208908	-2.140573
C	-5.604332	-1.845416	-2.403873
H	-6.446577	-2.489129	-2.173322
C	-5.674674	-0.455282	-2.712997
H	-6.579533	0.140885	-2.762927
C	-3.384674	1.966026	0.235737
H	-2.985403	2.303149	1.202522
H	-4.151143	2.678628	-0.093007
C	-1.406644	-1.039516	2.620638
C	-0.229552	-1.585591	3.145996
H	0.535910	-1.965154	2.468451
C	-0.026863	-1.644577	4.520056
H	0.886896	-2.088823	4.913752
C	-0.990232	-1.132911	5.389506
H	-0.832049	-1.174758	6.465052
C	-2.151066	-0.563190	4.875826
H	-2.903960	-0.156624	5.548331
C	-2.362572	-0.521167	3.498089
H	-3.284294	-0.086054	3.111146
C	-1.488194	-2.749427	0.330962
C	-1.895516	-3.758856	1.211203
H	-2.247893	-3.502956	2.209982
C	-1.856951	-5.095792	0.824432
H	-2.177635	-5.867915	1.520736
C	-1.409041	-5.443430	-0.448232
H	-1.378762	-6.488376	-0.749711
C	-0.995379	-4.447919	-1.329896
H	-0.636193	-4.710786	-2.323492
C	-1.030644	-3.111425	-0.940232
H	-0.686342	-2.336834	-1.624699
C	-1.586356	3.768154	-0.932460
C	-2.180309	4.702816	-0.084185
H	-2.945105	4.414774	0.632449
C	-1.773076	6.035555	-0.135690
H	-2.237203	6.757025	0.533654
C	-0.780438	6.441645	-1.020028
H	-0.463814	7.481641	-1.048723
C	-0.198249	5.506290	-1.874379
H	0.577937	5.808771	-2.575494
C	-0.601830	4.177502	-1.837715
H	-0.136155	3.448130	-2.501433
H	0.889309	1.366635	-1.308991
O	6.314953	-0.603934	-0.294995
C	6.588562	-1.033144	-1.635286
H	6.256145	-0.191767	-2.265141
H	6.579490	-1.319534	0.300930
O	3.254187	-2.478439	0.754859
C	2.247234	-3.430465	0.416168
H	1.311169	-2.889231	0.188821
H	2.880403	-1.874701	1.427133
C	3.347217	4.910727	-0.192025
H	2.858992	5.246525	0.731225
H	4.427111	4.837831	-0.000119
H	3.190630	5.679077	-0.960115
C	3.440165	3.116065	-1.939400
H	4.521209	2.958848	-1.803175
H	2.988199	2.182577	-2.303379
H	3.309677	3.872003	-2.725024
C	0.948421	-1.776529	-4.273389
H	0.758693	-1.745334	-5.353555
H	1.219939	-2.805715	-3.997706
H	0.014264	-1.516813	-3.757288
C	3.356746	-1.145195	-4.597898
H	4.158116	-0.465294	-4.280588
H	3.665453	-2.175749	-4.372643
H	3.237856	-1.053568	-5.683583
C	8.074538	-1.246430	-1.827832
H	8.638016	-0.346979	-1.555759
H	8.421405	-2.077088	-1.197075
H	8.303324	-1.496401	-2.870383
C	5.783945	-2.272021	-1.974078
H	6.001484	-2.610015	-2.994606
H	6.044235	-3.087993	-1.284520
H	4.702050	-2.094549	-1.901799
C	2.698230	-4.160142	-0.827778
H	3.654111	-4.671835	-0.647026
H	1.957608	-4.914627	-1.120552
H	2.827832	-3.461238	-1.663471
C	1.998741	-4.383328	1.569910
H	1.198262	-5.093944	1.323002
H	2.910096	-4.953776	1.797524
H	1.698840	-3.844570	2.479554
C	3.998718	-1.176103	3.832866
H	4.462353	-1.056966	4.819083
H	3.391546	-2.090902	3.845820
H	4.801632	-1.308573	3.092665
C	3.993251	1.299761	3.445993
H	4.401979	1.532841	4.437472
H	4.835187	1.179737	2.747821
H	3.394912	2.155878	3.108949
H	-3.149235	3.417932	3.622623
H	-2.691304	2.845628	3.481980

$TS_{5\text{-}6}$-K-iso

E (BS1) = -3854.97212530
E (BS2) = -4995.69838292
$G_{298.15,1M}$ (BS2) = -4994.673670

H	0.928978	3.431971	2.466807
H	0.733793	-0.720417	0.039548
H	-0.785302	-0.311852	-1.571787
K	3.507352	-0.465608	-0.479780
H	2.569149	0.501079	2.129886
O	2.889888	-0.450718	2.292008
C	3.994094	-0.433288	3.183777
H	3.681304	0.028784	4.139208
H	1.159313	-0.868585	-1.956852

O	1.859646	-1.303296	-2.484115
C	1.493365	-1.222690	-3.859480
H	1.379500	-0.158177	-4.138963
O	2.109226	1.948798	1.646048
C	1.948000	2.982975	2.556519
C	2.922304	4.129723	2.302471
C	2.070733	2.495917	3.997319
H	2.781053	4.536916	1.291350
H	3.964245	3.785335	2.388846
H	2.774748	4.951481	3.017984
H	1.838170	3.290753	4.720190
H	1.384063	1.655801	4.181959
H	3.095129	2.147411	4.199697
O	3.841330	2.195742	-0.262625
C	3.353120	3.015109	-1.311225
H	3.165951	2.192249	0.492571
H	2.307426	3.299681	-1.084878
Fe	-4.874482	0.029759	-0.820784
Ir	-0.319113	0.450167	-0.301351
P	-1.963541	-0.701720	0.918321
S	-1.860264	2.236283	-1.035993
C	-3.654113	-0.034882	0.810736
C	-4.006366	1.330270	0.495739
C	-5.430496	1.430902	0.554877
H	-5.997366	2.330434	0.334709
C	-5.963540	0.158615	0.901648
H	-7.015993	-0.092656	0.978567
C	-4.876868	-0.744432	1.055352
H	-4.955513	-1.803765	1.278517
C	-4.552212	0.662315	-2.742063
H	-4.110516	1.613889	-3.016538
C	-3.843551	-0.548090	-2.490196
H	-2.765354	-0.672767	-2.528970
C	-4.791456	-1.545558	-2.113217
H	-4.561799	-2.564715	-1.817093
C	-6.086244	-0.949414	-2.135612
H	-7.016036	-1.434557	-1.858086
C	-5.937987	0.414910	-2.521600
H	-6.735557	1.146547	-2.594232
C	-3.121800	2.514367	0.275472
H	-2.598891	2.765348	1.206223
H	-3.724212	3.377524	-0.031954
C	-1.642365	-0.719269	2.726319
C	-0.355208	-1.090697	3.131286
H	0.377210	-1.402835	2.385047
C	0.009649	-1.032016	4.470832
H	1.015988	-1.323083	4.770734
C	-0.906070	-0.582600	5.421711
H	-0.619546	-0.521616	6.469483
C	-2.187016	-0.208961	5.026165
H	-2.904492	0.141889	5.765269
C	-2.558591	-0.281914	3.684255
H	-3.563147	0.018103	3.387250
C	-2.197822	-2.487858	0.541431
C	-2.737391	-3.365132	1.488722
H	-3.014010	-2.997708	2.476873
C	-2.922881	-4.710118	1.180958
H	-3.343502	-5.381577	1.926522
C	-2.566541	-5.194983	-0.075661
H	-2.709242	-6.246749	-0.314412
C	-2.018484	-4.331625	-1.021377
H	-1.724527	-4.705818	-2.000601
C	-1.829328	-2.987567	-0.711781
H	-1.376472	-2.321254	-1.446024

C	-0.883762	3.744658	-0.961759
C	-0.617535	4.438984	0.217340
H	-1.047950	4.123660	1.165317
C	0.231173	5.543482	0.184594
H	0.435722	6.084179	1.107160
C	0.822138	5.947176	-1.009501
H	1.490703	6.805326	-1.025273
C	0.555101	5.246008	-2.183604
H	1.010607	5.554778	-3.122578
C	-0.302496	4.151263	-2.164814
H	-0.511787	3.602282	-3.082709
H	0.884850	1.156324	-1.196681
O	5.911099	-1.683944	-0.704574
C	5.950458	-2.208108	-2.037755
H	5.648950	-1.360410	-2.675679
H	6.135407	-2.405524	-0.099829
O	2.662981	-2.795342	0.768859
C	1.466732	-3.559412	0.641018
H	0.623926	-2.871020	0.447761
H	2.538237	-2.123923	1.468037
C	4.194298	4.274458	-1.418919
H	4.175738	4.842971	-0.480714
H	5.239260	4.009425	-1.634134
H	3.838860	4.929682	-2.224991
C	3.369039	2.239453	-2.617110
H	4.393358	1.909847	-2.850239
H	2.719059	1.354805	-2.567616
H	3.017302	2.860996	-3.451696
C	0.179555	-1.938054	-4.104569
H	-0.091515	-1.909441	-5.167235
H	0.257629	-2.990311	-3.794517
H	-0.635262	-1.469726	-3.535394
C	2.627070	-1.826916	-4.654366
H	3.573809	-1.313755	-4.440213
H	2.741116	-2.889800	-4.397647
H	2.431721	-1.753113	-5.730272
C	7.359085	-2.628034	-2.399440
H	8.062774	-1.799788	-2.260725
H	7.678809	-3.465337	-1.763146
H	7.414143	-2.957706	-3.443608
C	4.956713	-3.341053	-2.192862
H	4.989138	-3.752172	-3.209178
H	5.201321	-4.152504	-1.491932
H	3.927952	-3.012607	-1.993019
C	1.632789	-4.466709	-0.556785
H	2.517710	-5.106637	-0.431714
H	0.755759	-5.114933	-0.672973
H	1.753634	-3.881014	-1.478524
C	1.184390	-4.332232	1.913937
H	0.242865	-4.892067	1.828990
H	1.994635	-5.045676	2.119952
H	1.096206	-3.655838	2.775749
C	4.408474	-1.862434	3.457074
H	5.212894	-1.897727	4.201337
H	3.564148	-2.454014	3.834178
H	4.775668	-2.338519	2.536836
C	5.137484	0.379306	2.603915
H	5.957317	0.486621	3.325503
H	5.545163	-0.115557	1.708396
H	4.794721	1.381709	2.317003
H	-0.742446	2.222642	2.741934
H	-0.808053	1.787046	2.135066

6-K-iso

E (BS1) = -3854.98392566
E (BS2) = -4995.71020563
$G_{298.15,1M}$ (BS2) = -4994.681215

H	1.148450	2.629301	2.524968
H	0.633557	-0.795357	-0.329875
H	-0.849592	-0.010947	-1.786922
K	3.313652	-0.272656	-0.379984
H	2.711195	-0.250335	2.262031
O	2.860032	-1.253305	2.298934
C	3.951420	-1.530849	3.163018
H	3.667683	-1.261746	4.199269
H	1.211551	-0.384229	-2.250062
O	2.002902	-0.540786	-2.802368
C	1.718452	-0.030468	-4.102638
H	1.362774	1.013345	-4.007663
O	2.593969	1.288396	1.910858
C	2.075454	2.119308	2.892235
C	3.051128	3.229323	3.266152
C	1.666603	1.326485	4.128479
H	3.319470	3.827136	2.384111
H	3.978153	2.799172	3.675607
H	2.628634	3.909719	4.018981
H	1.227392	1.968682	4.904507
H	0.929703	0.553583	3.865799
H	2.544490	0.821021	4.561918
O	4.200283	2.304268	0.142674
C	3.497953	3.271242	-0.624258
H	3.608324	2.017849	0.905070
H	2.459930	3.349383	-0.240750
Fe	-4.908463	0.284942	-0.709536
Ir	-0.350552	0.478889	-0.333869
P	-1.968819	-0.885006	0.730183
S	-1.865000	2.420126	-0.560187
C	-3.641240	-0.183650	0.816022
C	-3.953098	1.224080	0.836384
C	-5.369136	1.348676	0.972819
H	-5.911147	2.289265	0.989251
C	-5.934567	0.044511	1.039776
H	-6.992371	-0.188943	1.097398
C	-4.876281	-0.899867	0.941940
H	-4.982210	-1.980047	0.921513
C	-4.661342	1.343059	-2.447290
H	-4.217574	2.330198	-2.514911
C	-3.960541	0.104246	-2.516784
H	-2.887099	-0.013341	-2.635364
C	-4.905710	-0.948602	-2.335601
H	-4.678056	-2.009479	-2.296310
C	-6.190873	-0.358117	-2.157579
H	-7.114397	-0.890053	-1.955181
C	-6.039447	1.057783	-2.224468
H	-6.827853	1.789986	-2.086972
C	-3.009426	2.380517	0.878613
H	-2.403989	2.327706	1.793315
H	-3.567022	3.324661	0.876944
C	-1.580666	-1.209062	2.495925
C	-0.375862	-1.858361	2.789863
H	0.285185	-2.169324	1.980471
C	-0.002468	-2.093543	4.107335
H	0.940434	-2.597123	4.317699
C	-0.822789	-1.667037	5.151159
H	-0.529642	-1.844338	6.183666
C	-2.012155	-1.003647	4.867962
H	-2.653710	-0.663689	5.678373
C	-2.395064	-0.778488	3.545670
H	-3.335025	-0.267465	3.338640
C	-2.258556	-2.570624	0.060308
C	-2.710263	-3.610055	0.881105
H	-2.889630	-3.430706	1.940922
C	-2.932585	-4.878350	0.352474
H	-3.280992	-5.679753	1.000496
C	-2.708233	-5.120155	-1.001115
H	-2.879416	-6.112521	-1.412760
C	-2.260137	-4.090460	-1.825035
H	-2.074900	-4.275216	-2.881423
C	-2.029167	-2.823759	-1.295592
H	-1.654211	-2.025581	-1.936203
C	-0.843817	3.853213	-0.191506
C	-0.585530	4.314476	1.097685
H	-1.039196	3.846140	1.967759
C	0.282049	5.390212	1.280236
H	0.484200	5.743939	2.289435
C	0.888876	6.002086	0.188201
H	1.567057	6.839409	0.338034
C	0.624501	5.537150	-1.098910
H	1.090321	6.012562	-1.959991
C	-0.242639	4.467942	-1.292501
H	-0.446116	4.102004	-2.298903
H	0.793302	1.342610	-1.146223
O	5.708532	-1.453247	-0.856615
C	5.791707	-1.628495	-2.276302
H	5.514392	-0.644795	-2.691094
H	5.891187	-2.310696	-0.445958
O	2.528364	-2.906523	0.101421
C	1.346878	-3.669614	-0.120031
H	0.468266	-3.002697	-0.044162
H	2.487378	-2.495133	0.989231
C	4.177933	4.621665	-0.490236
H	4.183953	4.960800	0.553625
H	5.220686	4.547204	-0.829581
H	3.677871	5.387752	-1.096881
C	3.443418	2.828896	-2.075727
H	4.462920	2.718359	-2.473745
H	2.921272	1.868715	-2.188543
H	2.914683	3.564198	-2.697284
C	0.638887	-0.862073	-4.766229
H	0.392616	-0.471300	-5.761308
H	0.978400	-1.901454	-4.876842
H	-0.280133	-0.862539	-4.163943
C	3.011816	-0.043061	-4.883657
H	3.776439	0.570850	-4.391226
H	3.392025	-1.070818	-4.966509
H	2.858638	0.347345	-5.896327
C	7.211294	-1.960366	-2.683137
H	7.911765	-1.202749	-2.314450
H	7.505155	-2.935008	-2.268443
H	7.304477	-2.016542	-3.774130
C	4.798737	-2.673493	-2.741599
H	4.867008	-2.821767	-3.826582
H	5.014493	-3.636689	-2.256189
H	3.767591	-2.384229	-2.500512
C	1.414543	-4.204893	-1.531167
H	2.307204	-4.832793	-1.660967
H	0.530011	-4.812462	-1.758013
H	1.462065	-3.380584	-2.255283
C	1.209806	-4.770397	0.912151
H	0.283097	-5.338037	0.751985
H	2.059365	-5.465144	0.852896

H	1.176538	-4.356443	1.929957
C	4.223576	-3.017914	3.107183
H	5.019229	-3.297506	3.807783
H	3.324017	-3.593503	3.361845
H	4.540362	-3.308804	2.095176
C	5.171900	-0.718647	2.769632
H	5.998156	-0.876952	3.474067
H	5.521775	-1.014537	1.768759
H	4.934287	0.352577	2.747526
H	0.761419	0.960272	1.068135
H	-0.025529	1.142899	1.321122

$TS_{6\text{-}7}$-K-iso

E (BS1) = -3854.98189925

E (BS2) = -4995.706254

$G_{298.15,1M}$ (BS2) = -4994.676609

H	0.478923	1.864197	3.223008
H	0.695558	-0.753226	-0.337689
H	-0.776459	-0.119278	-1.845253
K	3.361917	-0.205227	-0.330040
H	2.613375	-0.308537	2.364134
O	3.015645	-1.226067	2.336579
C	4.181589	-1.270799	3.149264
H	3.894422	-1.080849	4.200415
H	1.330773	-0.350111	-2.247989
O	2.164091	-0.458943	-2.749597
C	1.901287	-0.095455	-4.103182
H	1.283795	0.822171	-4.109213
O	2.054833	1.219202	2.065692
C	1.596406	1.920198	3.186726
C	1.970989	3.392250	3.094623
C	2.115313	1.313948	4.481888
H	1.616192	3.819669	2.146244
H	3.065384	3.505366	3.132425
H	1.538509	3.978148	3.917460
H	1.687515	1.817660	5.359180
H	1.859291	0.248210	4.544412
H	3.210010	1.409947	4.538453
O	3.733650	2.419975	0.368301
C	3.125858	3.392985	-0.475176
H	3.086186	2.124942	1.061633
H	2.039940	3.428473	-0.261355
Fe	-4.912616	0.043635	-0.709471
Ir	-0.358672	0.463199	-0.393818
P	-1.889665	-0.932452	0.714879
S	-1.971866	2.312604	-0.663689
C	-3.599951	-0.326158	0.807220
C	-3.979978	1.063557	0.799567
C	-5.398486	1.123476	0.954412
H	-5.985776	2.036702	0.957179
C	-5.898526	-0.205062	1.060325
H	-6.942582	-0.488574	1.140487
C	-4.796180	-1.098809	0.967886
H	-4.848866	-2.183122	0.975372
C	-4.730333	1.071795	-2.472458
H	-4.325779	2.073811	-2.564770
C	-3.980838	-0.139150	-2.524342
H	-2.904817	-0.215141	-2.652522
C	-4.880685	-1.224700	-2.308829
H	-4.611191	-2.274764	-2.251441
C	-6.186391	-0.682525	-2.126450
H	-7.085166	-1.246842	-1.901413
C	-6.093021	0.736568	-2.225289
H	-6.908767	1.439271	-2.093546
C	-3.088548	2.261217	0.795123
H	-2.462571	2.257982	1.698382
H	-3.688559	3.179072	0.781297
C	-1.462405	-1.174716	2.486335
C	-0.197106	-1.695803	2.784781
H	0.493912	-1.946897	1.978673
C	0.194596	-1.887442	4.103770
H	1.180559	-2.301165	4.314483
C	-0.660212	-1.528162	5.145500
H	-0.349584	-1.665962	6.179063
C	-1.908391	-0.986521	4.857946
H	-2.578307	-0.700383	5.666263
C	-2.314219	-0.818290	3.533878
H	-3.301491	-0.407666	3.323697
C	-2.113952	-2.662096	0.127615
C	-2.476312	-3.691661	1.003686
H	-2.616327	-3.482853	2.064094
C	-2.661103	-4.987777	0.530266
H	-2.939342	-5.780381	1.221691
C	-2.489271	-5.268770	-0.823446
H	-2.631704	-6.282412	-1.192017
C	-2.130507	-4.249731	-1.702484
H	-1.987808	-4.463647	-2.760065
C	-1.936780	-2.955005	-1.227857
H	-1.632799	-2.163019	-1.912636
C	-1.070378	3.839370	-0.379250
C	-0.952891	4.460734	0.862359
H	-1.414665	4.041032	1.753072
C	-0.220789	5.642950	0.970675
H	-0.133936	6.125573	1.942268
C	0.399624	6.196566	-0.143480
H	0.970396	7.117941	-0.050521
C	0.287834	5.563505	-1.380869
H	0.769168	5.988806	-2.259502
C	-0.448461	4.391765	-1.502967
H	-0.539199	3.900037	-2.471738
H	0.777617	1.363610	-1.187172
O	5.827393	-1.220789	-0.832245
C	5.903691	-1.395189	-2.253002
H	5.548119	-0.436624	-2.666601
H	6.072329	-2.062951	-0.422654
O	2.739941	-2.891871	0.147612
C	1.529588	-3.589569	-0.132243
H	0.677126	-2.897722	-0.004772
H	2.693072	-2.516370	1.049272
C	3.732331	4.756666	-0.202544
H	3.560902	5.067872	0.835614
H	4.817291	4.723194	-0.374695
H	3.307003	5.522691	-0.863673
C	3.322214	2.978858	-1.921586
H	4.396072	2.892215	-2.145172
H	2.842984	2.012089	-2.130251
H	2.892466	3.718374	-2.610252
C	1.149386	-1.209376	-4.803781
H	0.904757	-0.935756	-5.837647
H	1.765164	-2.120279	-4.824407
H	0.212024	-1.440261	-4.279961
C	3.227782	0.197372	-4.766366
H	3.749948	1.020493	-4.263001
H	3.870669	-0.693745	-4.734729
H	3.083067	0.474191	-5.817123
C	7.338066	-1.623064	-2.678527

H	7.985845	-0.814101	-2.322603
H	7.709160	-2.571519	-2.265065
H	7.419597	-1.676473	-3.770633
C	4.982912	-2.511741	-2.701289
H	5.050259	-2.664269	-3.786035
H	5.267862	-3.453951	-2.210293
H	3.937122	-2.291070	-2.452358
C	1.580841	-4.011066	-1.582185
H	2.458664	-4.646652	-1.766525
H	0.682162	-4.578440	-1.852705
H	1.644617	-3.129926	-2.236641
C	1.350247	-4.757621	0.815658
H	0.400088	-5.274348	0.622573
H	2.169128	-5.481042	0.698601
H	1.342188	-4.415810	1.860699
C	4.754924	-2.665724	3.045812
H	5.617847	-2.784685	3.711197
H	4.002704	-3.417694	3.317158
H	5.086248	-2.865893	2.016749
C	5.180658	-0.212453	2.719886
H	6.044055	-0.187718	3.396489
H	5.557556	-0.425768	1.707700
H	4.720497	0.784861	2.717322
H	0.824300	1.093623	1.274967
H	-0.061523	1.221178	1.205447

7-K-iso

E (BS1) = -3855.00600742

E (BS2) = -4995.72823124

$G_{298.15,1M}$ (BS2) = -4994.692949

H	0.393424	0.515769	3.935779
H	0.848970	-0.542006	0.083668
H	-0.322416	0.113602	-1.811091
K	3.478654	-0.151501	-0.336712
H	2.565633	-1.116206	2.299821
O	3.015297	-1.902755	1.921516
C	4.167434	-2.211837	2.705175
H	3.854407	-2.365368	3.753363
H	1.419413	0.321066	-2.202782
O	2.283618	0.370428	-2.669650
C	2.077830	1.124681	-3.859869
H	1.467866	2.014725	-3.612181
O	1.969848	0.556232	2.603502
C	1.444523	0.853321	3.906587
C	1.506024	2.346615	4.149146
C	2.248135	0.068025	4.915909
H	0.941150	2.893672	3.381957
H	2.550990	2.688370	4.119814
H	1.089817	2.604472	5.130730
H	1.857801	0.225156	5.928297
H	2.205878	-1.006325	4.694108
H	3.300206	0.386753	4.901418
O	3.731547	2.163908	1.156170
C	3.118593	3.357141	0.659015
H	3.173002	1.799872	1.869667
H	2.020057	3.255957	0.738578
Fe	-4.980124	0.121283	-0.553961
Ir	-0.301065	0.560299	-0.167905
P	-1.886893	-0.989573	0.561148
S	-1.960536	2.328359	-0.548073
C	-3.571612	-0.339474	0.845301
C	-3.940891	1.053741	0.933647
C	-5.345265	1.113934	1.189933
H	-5.920585	2.030850	1.275868
C	-5.851581	-0.213186	1.263604
H	-6.890310	-0.493804	1.402163
C	-4.767456	-1.106459	1.049136
H	-4.838189	-2.188162	1.001312
C	-4.878008	1.243300	-2.263293
H	-4.463310	2.244039	-2.320064
C	-4.149760	0.028270	-2.420920
H	-3.084447	-0.054393	-2.614527
C	-5.053370	-1.057407	-2.217671
H	-4.796067	-2.112587	-2.230318
C	-6.339539	-0.510517	-1.936308
H	-7.233756	-1.075612	-1.695503
C	-6.230680	0.910795	-1.962617
H	-7.027948	1.614788	-1.749351
C	-3.077321	2.271645	0.906534
H	-2.450563	2.297927	1.807433
H	-3.705175	3.171050	0.878415
C	-1.437453	-1.642985	2.224352
C	-0.329122	-2.492109	2.323453
H	0.188240	-2.813274	1.418133
C	0.131103	-2.918100	3.563749
H	0.997271	-3.577510	3.618137
C	-0.508064	-2.493019	4.728558
H	-0.146642	-2.820439	5.701387
C	-1.607883	-1.644877	4.640200
H	-2.112456	-1.309937	5.544432
C	-2.073319	-1.223380	3.394349
H	-2.933276	-0.555214	3.341006
C	-2.228588	-2.551007	-0.364928
C	-2.797285	-3.668924	0.258330
H	-3.009702	-3.649223	1.327109
C	-3.091765	-4.814951	-0.474215
H	-3.535182	-5.675094	0.023288
C	-2.813944	-4.862276	-1.839298
H	-3.043297	-5.758895	-2.411375
C	-2.232247	-3.762817	-2.464867
H	-2.002927	-3.796411	-3.528456
C	-1.937185	-2.616407	-1.729550
H	-1.462502	-1.761963	-2.214967
C	-1.074239	3.865309	-0.265480
C	-0.815932	4.395711	0.998050
H	-1.195868	3.916954	1.897285
C	-0.046350	5.551097	1.113492
H	0.155761	5.959964	2.102051
C	0.470332	6.173558	-0.019883
H	1.070379	7.075895	0.077535
C	0.210382	5.638178	-1.279654
H	0.604817	6.120006	-2.171880
C	-0.564685	4.490181	-1.405563
H	-0.769671	4.071572	-2.390890
H	0.925025	1.608197	-0.528658
O	5.905781	-1.156722	-1.048848
C	6.040403	-0.938436	-2.459975
H	5.809078	0.131114	-2.595024
H	6.037813	-2.101982	-0.886510
O	2.730875	-2.834078	-0.682283
C	1.482169	-3.332959	-1.157006
H	0.677872	-2.645127	-0.837819
H	2.676462	-2.741660	0.287664
C	3.569748	4.555286	1.469487
H	3.286636	4.446354	2.524592
H	4.661851	4.662634	1.415226
H	3.114056	5.479129	1.090473

C	3.486737	3.481416	-0.803388
H	4.580071	3.488359	-0.922503
H	3.073766	2.645951	-1.385416
H	3.091065	4.412376	-1.228781
C	1.345676	0.288952	-4.891875
H	1.142355	0.869026	-5.800793
H	1.956014	-0.582646	-5.169706
H	0.388193	-0.074333	-4.495716
C	3.432121	1.574829	-4.359321
H	3.947891	2.190890	-3.612296
H	4.060378	0.700559	-4.581704
H	3.331748	2.162800	-5.279202
C	7.465037	-1.202085	-2.898530
H	8.172443	-0.601417	-2.315871
H	7.713502	-2.263539	-2.757730
H	7.599687	-0.962502	-3.959915
C	5.038384	-1.776072	-3.228481
H	5.143828	-1.616066	-4.309083
H	5.210795	-2.843914	-3.028323
H	4.005521	-1.531559	-2.947837
C	1.539687	-3.326803	-2.666132
H	2.365022	-3.959302	-3.022556
H	0.604502	-3.711097	-3.091070
H	1.695323	-2.305711	-3.039192
C	1.207164	-4.710567	-0.588096
H	0.214917	-5.070192	-0.893951
H	1.959603	-5.430767	-0.938467
H	1.237364	-4.695077	0.511166
C	4.735522	-3.502577	2.163538
H	5.594625	-3.831122	2.759171
H	3.978638	-4.297037	2.176549
H	5.072177	-3.362589	1.126075
C	5.172381	-1.077496	2.650759
H	6.018594	-1.270696	3.321538
H	5.576113	-0.967978	1.632617
H	4.714539	-0.125331	2.951500
H	1.237938	0.701257	1.949956
H	-0.244272	1.103312	1.447751

D5. Tetramethyl ammonium system

1-TMA-iso

E (BS1) = -3274.99295207

E (BS2) = -4415.687588

$G_{298.15,1M}$ (BS2) = -4414.605013

H	1.264074	-1.154958	1.092611
O	1.800365	-1.936669	1.348137
C	2.797596	-1.578296	2.303958
H	3.321638	-0.670040	1.957367
H	7.135938	3.367199	-1.022035
H	2.154209	-2.911596	-0.097282
O	2.384081	-3.493468	-0.857420
O	6.359292	2.920138	-0.658113
C	1.602831	-4.685935	-0.758469
H	2.061578	-5.392080	-1.469430
C	5.753741	3.810870	0.277501
H	4.850890	3.278501	0.622079
Fe	-5.046366	1.120802	-0.439018
Ir	-0.455787	0.070592	-0.690359
P	-2.286490	-0.797600	0.479487
S	-1.511735	2.269644	-1.010256
C	-3.657229	0.364649	0.840700
C	-3.585891	1.805813	0.811735
C	-4.862809	2.312209	1.207101
H	-5.125787	3.365059	1.247245
C	-5.720273	1.213515	1.488784
H	-6.766178	1.274078	1.770513
C	-4.985155	0.018751	1.263751
H	-5.379657	-0.987790	1.349617
C	-4.850902	2.025131	-2.263336
H	-4.179579	2.852267	-2.469428
C	-4.522366	0.641057	-2.355587
H	-3.555780	0.233549	-2.637188
C	-5.662409	-0.111787	-1.944245
H	-5.716928	-1.193747	-1.866166
C	-6.695044	0.808956	-1.599554
H	-7.674660	0.552006	-1.210561
C	-6.193190	2.129186	-1.795658
H	-6.725713	3.050630	-1.585401
C	-2.426132	2.699725	0.530915
H	-1.692869	2.672482	1.350484
H	-2.781232	3.731948	0.414527
C	-1.904355	-1.385451	2.187819
C	-1.374792	-2.662955	2.400038
H	-1.238546	-3.343189	1.558171
C	-1.034753	-3.085030	3.681667
H	-0.634214	-4.086597	3.829920
C	-1.201643	-2.229821	4.768719
H	-0.935838	-2.560582	5.770686
C	-1.702766	-0.947299	4.564642
H	-1.832235	-0.269940	5.406582
C	-2.054473	-0.529358	3.283198
H	-2.455177	0.474907	3.139490
C	-3.189153	-2.255254	-0.214326
C	-4.031798	-3.054671	0.569684
H	-4.139701	-2.856189	1.635231
C	-4.727577	-4.118974	0.005672
H	-5.378308	-4.728903	0.629106
C	-4.583187	-4.409351	-1.349786
H	-5.123072	-5.245866	-1.788748
C	-3.735778	-3.633819	-2.134642
H	-3.606588	-3.861754	-3.190877
C	-3.043304	-2.565197	-1.568772
H	-2.371498	-1.961675	-2.178794
C	-0.340073	3.636667	-0.964147
C	-0.690505	4.837654	-1.582966
H	-1.658345	4.933978	-2.074683
C	0.205045	5.904242	-1.572150
H	-0.064225	6.838730	-2.059797
C	1.443197	5.773033	-0.946095
H	2.143602	6.606421	-0.944843
C	1.782712	4.574561	-0.323325
H	2.745347	4.459488	0.175340
C	0.893431	3.504561	-0.334842
H	1.156244	2.565920	0.145085
H	0.237404	-1.384190	-0.708625
H	0.896553	0.589702	-1.489846
H	0.349254	0.434768	0.802522
H	-1.069212	-0.298024	-2.208658
O	2.668906	1.766860	1.759217
C	2.488994	2.281802	3.076202
H	2.684262	1.476511	3.808809
H	1.911045	1.191916	1.544717
O	5.153891	-3.265429	-0.928637
C	5.766919	-4.425394	-1.479450
H	6.850185	-4.269100	-1.362751
H	4.184081	-3.422635	-0.919489

C	1.075677	2.788529	3.281026
H	0.858048	3.610677	2.584850
H	0.341407	1.988774	3.111309
H	0.940416	3.159292	4.304468
C	3.518814	3.371229	3.268932
H	3.467087	3.779628	4.284814
H	4.531233	2.981891	3.106333
H	3.342186	4.192068	2.557898
C	5.357473	-5.663922	-0.706892
H	5.547358	-5.536144	0.366075
H	4.284152	-5.860979	-0.847550
H	5.906500	-6.548811	-1.051133
C	5.436918	-4.548886	-2.954468
H	4.350316	-4.657111	-3.088120
H	5.762077	-3.658337	-3.506548
H	5.921496	-5.425463	-3.402127
C	5.349194	5.103877	-0.401305
H	6.241206	5.634162	-0.765120
H	4.691183	4.913658	-1.258439
H	4.826974	5.771636	0.296646
C	6.679368	4.054188	1.451873
H	6.965015	3.109680	1.929336
H	7.595918	4.556097	1.109837
H	6.209041	4.697733	2.205691
C	1.679129	-5.267770	0.639391
H	1.125268	-6.212331	0.700895
H	2.719385	-5.458713	0.932591
H	1.241448	-4.566755	1.365156
C	0.170145	-4.420007	-1.179240
H	-0.419097	-5.346081	-1.179222
H	-0.304013	-3.711437	-0.484742
H	0.130047	-3.982514	-2.184526
C	2.154886	-1.292460	3.645324
H	1.394677	-0.503443	3.559697
H	1.662694	-2.198003	4.025617
H	2.904054	-0.972500	4.380999
C	3.778132	-2.726845	2.382086
H	4.591419	-2.490316	3.078099
H	3.271653	-3.631588	2.746429
H	4.218143	-2.950270	1.400557
N	4.249106	0.158536	-2.002918
C	5.708307	0.067117	-2.298440
H	5.865741	0.312512	-3.352057
H	6.234108	0.781060	-1.658703
H	6.036618	-0.955264	-2.092443
C	4.016952	-0.155619	-0.559885
H	4.452070	-1.138136	-0.352706
H	4.502924	0.616517	0.043165
H	2.936784	-0.162355	-0.379508
C	3.510015	-0.826118	-2.847053
H	2.439907	-0.732874	-2.637541
H	3.714583	-0.605067	-3.898045
H	3.863529	-1.828108	-2.588265
C	3.763945	1.540949	-2.292933
H	4.357097	2.245733	-1.701043
H	3.898076	1.737925	-3.360198
H	2.703332	1.596127	-2.028026

2-TMA-iso

E (BS1) = -3468.03801393

E (BS2) = -4608.805013

$G_{298.15,1M}$ (BS2) = -4607.645689

H	3.589540	-1.042190	1.639167
O	4.435270	-1.529811	1.677073
C	5.371324	-0.698496	2.372203
H	5.321931	0.320687	1.948148
H	5.514256	3.815924	-3.376317
H	4.844136	-2.521262	0.241910
O	5.052605	-3.127204	-0.505192
O	5.341528	3.287723	-2.584544
C	4.790472	-4.458930	-0.058456
H	5.013127	-5.101772	-0.924241
C	5.176093	4.194508	-1.492863
H	4.959154	3.548150	-0.626902
Fe	-5.967956	0.987635	-0.019765
Ir	-1.546033	-0.184871	-1.037314
P	-3.295518	-1.237402	0.083754
S	-2.377690	2.083230	-0.587933
C	-4.547729	-0.165054	0.875746
C	-4.363405	1.222050	1.221525
C	-5.561251	1.674505	1.857590
H	-5.734973	2.689697	2.201815
C	-6.480200	0.590597	1.917404
H	-7.493484	0.632058	2.302798
C	-5.863018	-0.538225	1.313487
H	-6.327049	-1.507242	1.164228
C	-5.845416	2.335196	-1.555281
H	-5.127672	3.146863	-1.609081
C	-5.644988	1.009525	-2.038932
H	-4.742112	0.636157	-2.513869
C	-6.807038	0.244083	-1.723402
H	-6.946858	-0.814587	-1.922565
C	-7.725541	1.099621	-1.046899
H	-8.687919	0.807435	-0.640159
C	-7.130578	2.391103	-0.941611
H	-7.563379	3.253036	-0.445056
C	-3.151474	2.078320	1.090698
H	-2.363964	1.758269	1.789074
H	-3.415304	3.118977	1.317953
C	-2.753336	-2.259725	1.521792
C	-2.070459	-3.458191	1.281373
H	-1.912787	-3.795288	0.256001
C	-1.596484	-4.227855	2.337570
H	-1.074734	-5.160985	2.134083
C	-1.787317	-3.804891	3.652547
H	-1.417579	-4.407457	4.479579
C	-2.450218	-2.607203	3.901313
H	-2.602618	-2.269197	4.924589
C	-2.933480	-1.839479	2.841812
H	-3.457404	-0.906854	3.052747
C	-4.364322	-2.448363	-0.817539
C	-5.140796	-3.396367	-0.138274
H	-5.082598	-3.474461	0.947068
C	-5.987436	-4.250653	-0.837186
H	-6.586269	-4.979894	-0.295160
C	-6.061027	-4.179267	-2.227139
H	-6.720108	-4.851146	-2.773170
C	-5.278006	-3.256382	-2.913354
H	-5.319205	-3.204145	-3.999535
C	-4.434066	-2.397976	-2.211393
H	-3.816105	-1.676193	-2.745749
C	-1.084990	3.275490	-0.183313
C	-1.426915	4.629742	-0.191996
H	-2.440344	4.931634	-0.456038
C	-0.472201	5.586508	0.138156
H	-0.741115	6.640614	0.135025
C	0.824515	5.193851	0.463398

H	1.571738	5.943000	0.720840
C	1.167432	3.843813	0.456690
H	2.183881	3.531517	0.705395
C	0.210764	2.880264	0.138792
H	0.458634	1.818807	0.136212
H	-0.970384	-1.643165	-1.436326
H	-0.205462	0.402937	-1.817698
H	-0.594386	-0.250339	0.367142
H	-2.332159	-0.128488	-2.548265
O	4.051734	2.361348	1.427821
C	4.473033	2.979627	2.640748
H	5.221400	2.328605	3.133513
H	3.375016	1.692859	1.640717
O	7.591686	-2.247401	-1.162444
C	7.932583	-2.765756	-2.439734
H	8.922542	-2.345347	-2.675641
H	6.727960	-2.639305	-0.917264
C	1.280313	-0.192752	2.408940
O	2.255815	0.222690	1.776295
C	0.355300	0.734155	3.114695
C	1.062128	-1.663881	2.551655
H	0.520210	1.774165	2.815318
H	1.135921	-2.141573	1.566505
H	0.536435	0.629956	4.195154
H	-0.686651	0.438855	2.943067
H	0.100357	-1.908757	3.012555
H	1.870321	-2.071442	3.175774
C	3.305972	3.174207	3.586402
H	2.549844	3.834412	3.137840
H	2.828423	2.213547	3.824061
H	3.638735	3.624946	4.529028
C	5.133323	4.287511	2.270810
H	5.527115	4.792020	3.160618
H	5.964307	4.121765	1.574110
H	4.403086	4.955398	1.789685
C	8.037304	-4.278256	-2.390834
H	8.745066	-4.595020	-1.615875
H	7.055047	-4.717755	-2.158536
H	8.369572	-4.690019	-3.351852
C	6.939529	-2.308095	-3.492659
H	5.938675	-2.704326	-3.260817
H	6.881116	-1.212455	-3.523836
H	7.219993	-2.665635	-4.491813
C	3.999626	5.117803	-1.735142
H	4.179050	5.734978	-2.627415
H	3.071668	4.552819	-1.890625
H	3.849954	5.797196	-0.886550
C	6.458974	4.961676	-1.246399
H	7.296819	4.277836	-1.067267
H	6.702755	5.581629	-2.121105
H	6.365002	5.629224	-0.380880
C	5.714240	-4.821106	1.086613
H	5.609675	-5.877229	1.361793
H	6.761009	-4.635077	0.813353
H	5.476485	-4.214405	1.972033
C	3.328326	-4.600400	0.314775
H	3.096824	-5.625269	0.629154
H	3.088499	-3.928228	1.151480
H	2.676813	-4.344898	-0.529754
C	5.006888	-0.639682	3.841357
H	4.007078	-0.205253	3.981323
H	5.004928	-1.651696	4.269092
H	5.723675	-0.029066	4.404342
C	6.749283	-1.267737	2.134488
H	7.509400	-0.648716	2.624722
H	6.822989	-2.284295	2.544459
H	6.982272	-1.308265	1.062538
N	3.556249	0.121038	-1.918446
C	3.762339	-0.054453	-3.384299
H	2.785320	-0.050246	-3.875245
H	4.376348	0.771332	-3.753106
H	4.266639	-1.009624	-3.554428
C	4.881578	0.185381	-1.230777
H	5.438987	-0.736116	-1.429629
H	5.424057	1.053519	-1.618378
H	4.702837	0.296974	-0.157531
C	2.757653	-1.027518	-1.394081
H	2.541054	-0.845638	-0.336526
H	1.819661	-1.081646	-1.953253
H	3.346441	-1.940500	-1.514225
C	2.819421	1.397301	-1.671828
H	3.396468	2.210861	-2.121184
H	1.826015	1.318479	-2.123850
H	2.730407	1.534295	-0.589659

$TS_{2\text{-}3}$-TMA-iso
E (BS1) = -3468.01795692
E (BS2) = -4608.786575
$G_{298.15,1M}$ (BS2) = -4607.631099

H	2.936496	-1.253066	1.768173
O	3.737642	-1.799528	1.906559
C	4.518528	-1.135548	2.889440
H	4.738704	-0.102424	2.554592
H	6.205259	3.365189	-2.606839
H	5.387666	-3.451507	-0.429726
O	5.861016	-3.019528	-1.158018
O	5.699680	2.879545	-1.940222
C	6.218537	-4.021753	-2.121040
H	6.818291	-3.475895	-2.866899
C	5.501244	3.756557	-0.829719
H	4.925481	3.159701	-0.102636
Fe	-5.929323	1.217176	-0.457946
Ir	-1.460772	-0.476813	-0.717069
P	-3.501648	-1.207685	0.205333
S	-2.232767	1.844270	-0.831277
C	-4.701334	0.080723	0.705487
C	-4.418965	1.483044	0.887750
C	-5.617700	2.111703	1.347112
H	-5.725980	3.175251	1.538095
C	-6.634562	1.123514	1.458275
H	-7.667732	1.298247	1.739405
C	-6.077782	-0.121606	1.060984
H	-6.614967	-1.062003	0.999057
C	-5.571303	2.373781	-2.106852
H	-4.792714	3.126857	-2.171225
C	-5.425932	0.992750	-2.427567
H	-4.518178	0.513790	-2.781394
C	-6.664730	0.341657	-2.149506
H	-6.864520	-0.720568	-2.255286
C	-7.575096	1.322911	-1.658903
H	-8.589110	1.139283	-1.319665
C	-6.899206	2.578018	-1.631338
H	-7.309970	3.514768	-1.269788
C	-3.136799	2.225631	0.730910
H	-2.445356	2.008315	1.558014
H	-3.340842	3.304301	0.719960
C	-3.335452	-2.150364	1.780363

C	-2.886862	-3.476239	1.743840
H	-2.702289	-3.960845	0.784416
C	-2.675074	-4.184788	2.921580
H	-2.335428	-5.217574	2.876453
C	-2.890269	-3.572126	4.155061
H	-2.720864	-4.124954	5.076662
C	-3.322181	-2.250303	4.202170
H	-3.493309	-1.764649	5.160971
C	-3.549544	-1.544781	3.021522
H	-3.895684	-0.511942	3.073162
C	-4.570511	-2.353402	-0.778468
C	-5.556048	-3.143852	-0.172109
H	-5.668258	-3.142662	0.911473
C	-6.392479	-3.947935	-0.939412
H	-7.152554	-4.555271	-0.452046
C	-6.251186	-3.982750	-2.325474
H	-6.902820	-4.615558	-2.924474
C	-5.263994	-3.216498	-2.936967
H	-5.137038	-3.248116	-4.017293
C	-4.428607	-2.409989	-2.166869
H	-3.649854	-1.816412	-2.644547
C	-0.945967	3.088197	-0.633379
C	-1.215899	4.368856	-1.120837
H	-2.172044	4.575201	-1.601525
C	-0.262401	5.373348	-0.992496
H	-0.473269	6.370582	-1.372712
C	0.960329	5.096789	-0.385588
H	1.707822	5.882009	-0.288374
C	1.228761	3.817616	0.096087
H	2.182979	3.588002	0.571912
C	0.273870	2.807719	-0.020928
H	0.473379	1.801717	0.352921
H	-0.949029	-2.010734	-0.804189
H	-0.004944	-0.109347	-1.397063
H	-0.642886	-0.314163	0.849638
H	-1.998242	-0.655131	-2.267159
O	3.426380	2.024297	1.783785
C	3.689237	2.585372	3.062024
H	4.165929	1.820455	3.706334
H	2.682050	1.389237	1.855412
O	7.975606	-1.564244	0.004147
C	8.878493	-1.060758	-0.969515
H	9.568916	-0.407007	-0.414413
H	7.317653	-2.125274	-0.451724
C	0.484674	-0.430519	2.110938
O	1.599529	-0.019138	1.675562
C	-0.304469	0.466133	3.029335
C	0.321407	-1.909618	2.335467
H	-0.262012	1.508450	2.692782
H	0.761204	-2.479829	1.509916
H	0.165123	0.409672	4.025345
H	-1.346745	0.144629	3.130680
H	-0.724078	-2.200014	2.466496
H	0.869363	-2.162761	3.257551
C	2.408223	3.045101	3.727228
H	1.896909	3.791059	3.102529
H	1.723918	2.201044	3.881677
H	2.611325	3.496116	4.706227
C	4.667231	3.719952	2.859085
H	4.958310	4.162826	3.818488
H	5.574660	3.363805	2.355575
H	4.211956	4.508019	2.241297
C	9.673433	-2.186930	-1.604141
H	10.164785	-2.797582	-0.837425
H	9.007351	-2.839603	-2.189132
H	10.443048	-1.800049	-2.283516
C	8.156325	-0.236260	-2.018674
H	7.446482	-0.869696	-2.572545
H	7.597261	0.591918	-1.564705
H	8.862430	0.185222	-2.744980
C	4.685329	4.962196	-1.246797
H	5.233560	5.552733	-1.995134
H	3.727763	4.656084	-1.686793
H	4.480149	5.616930	-0.390399
C	6.832584	4.143114	-0.220443
H	7.392570	3.252804	0.091346
H	7.439828	4.692572	-0.954205
H	6.698317	4.791345	0.654269
C	7.065471	-5.093904	-1.470744
H	7.438033	-5.806796	-2.215769
H	7.925231	-4.651934	-0.951989
H	6.471266	-5.653402	-0.734693
C	4.975501	-4.579280	-2.779525
H	5.236783	-5.316211	-3.547467
H	4.343276	-5.078750	-2.032823
H	4.386834	-3.784912	-3.253577
C	3.766504	-1.057660	4.205352
H	2.824087	-0.505618	4.077329
H	3.526037	-2.067783	4.565260
H	4.358149	-0.548722	4.977217
C	5.820918	-1.892316	3.013512
H	6.466433	-1.441320	3.776258
H	5.625254	-2.934644	3.300039
H	6.367781	-1.891280	2.061339
N	3.767421	-0.126903	-1.560057
C	4.424820	-0.331736	-2.881822
H	3.649042	-0.452851	-3.642717
H	5.039336	0.544908	-3.103747
H	5.043978	-1.231973	-2.820737
C	4.814220	0.057929	-0.506788
H	5.436421	-0.838896	-0.465507
H	5.413262	0.930526	-0.783613
H	4.311851	0.227972	0.447419
C	2.912915	-1.310946	-1.243061
H	2.375096	-1.111267	-0.311091
H	2.201636	-1.449722	-2.062028
H	3.560374	-2.185846	-1.144575
C	2.912428	1.096895	-1.603387
H	3.542819	1.946937	-1.882746
H	2.116155	0.945361	-2.337831
H	2.484942	1.238940	-0.606786

```

**3-TMA-iso**

E (BS1) = -3468.04575251

E (BS2) = -4608.814900

$G_{298.15,1M}$ (BS2) = -4607.648520

| | | | |
|---|---|---|---|
| H | 3.286303 | -0.922461 | 1.472461 |
| O | 4.140465 | -1.457048 | 1.578050 |
| C | 4.968922 | -0.722427 | 2.469846 |
| H | 4.954018 | 0.343291 | 2.173268 |
| H | 5.969300 | 4.079398 | -2.799435 |
| H | 4.768056 | -2.427003 | 0.282147 |
| O | 5.121283 | -2.977672 | -0.459281 |
| O | 5.609520 | 3.472273 | -2.137918 |
| C | 4.934668 | -4.351391 | -0.124497 |
| H | 5.288790 | -4.913607 | -1.003582 |
| C | 5.411711 | 4.219602 | -0.936951 |
```

H	4.999116	3.490645	-0.220132
Fe	-5.872551	1.021439	0.203196
Ir	-1.397096	-0.255908	-1.010957
P	-3.256800	-1.289632	0.028216
S	-2.318075	1.997854	-0.563587
C	-4.422338	-0.211846	0.930016
C	-4.191053	1.150201	1.346174
C	-5.336233	1.575589	2.088365
H	-5.472365	2.571009	2.500255
C	-6.268424	0.502761	2.141211
H	-7.253336	0.534414	2.594670
C	-5.714831	-0.592467	1.426590
H	-6.201276	-1.546802	1.253718
C	-5.834347	2.508126	-1.200520
H	-5.113257	3.319013	-1.217515
C	-5.676628	1.235448	-1.821913
H	-4.816907	0.912684	-2.400352
C	-6.823313	0.445552	-1.510429
H	-6.987138	-0.586047	-1.806973
C	-7.690190	1.233704	-0.698823
H	-8.627561	0.905923	-0.262111
C	-7.078791	2.507143	-0.506180
H	-7.470570	3.316299	0.100905
C	-3.000338	2.024934	1.152689
H	-2.179009	1.747162	1.828132
H	-3.278596	3.067172	1.356273
C	-2.805548	-2.511575	1.327373
C	-2.253102	-3.738911	0.939787
H	-2.153324	-3.979531	-0.119251
C	-1.833502	-4.657033	1.895258
H	-1.415271	-5.610879	1.580526
C	-1.940432	-4.352597	3.251525
H	-1.606177	-5.068594	3.999215
C	-2.474738	-3.130076	3.645341
H	-2.561712	-2.886456	4.702241
C	-2.911843	-2.215362	2.688650
H	-3.338287	-1.265349	3.011220
C	-4.385279	-2.280001	-1.041796
C	-5.221798	-3.266830	-0.504986
H	-5.169734	-3.510293	0.555551
C	-6.118481	-3.953301	-1.317255
H	-6.761580	-4.717072	-0.884993
C	-6.186551	-3.670007	-2.679858
H	-6.885142	-4.210606	-3.314940
C	-5.349038	-2.702815	-3.226710
H	-5.387241	-2.483892	-4.291757
C	-4.453056	-2.014337	-2.412069
H	-3.796671	-1.261933	-2.846328
C	-1.076299	3.279420	-0.337033
C	-1.472628	4.599947	-0.559294
H	-2.499379	4.815285	-0.853968
C	-0.553355	5.632604	-0.406147
H	-0.862474	6.661106	-0.579024
C	0.759407	5.344902	-0.040747
H	1.480649	6.152179	0.074646
C	1.152207	4.026715	0.178361
H	2.173478	3.786420	0.476017
C	0.233836	2.987022	0.038808
H	0.541313	1.954708	0.218630
H	-0.862640	-1.705497	-1.480842
H	-0.094612	0.332666	-1.842411
H	0.207997	-0.252683	0.573575
H	-2.156678	-0.179480	-2.367468
O	3.537378	2.294883	1.564947
C	3.734491	2.823040	2.863797
H	4.371411	2.129937	3.451070
H	2.895242	1.535110	1.601083
O	7.667281	-1.978091	-0.859886
C	8.174498	-2.534066	-2.064001
H	9.139455	-2.032813	-2.237921
H	6.799079	-2.402330	-0.689744
C	0.841032	-0.448352	1.513228
O	2.083788	0.097487	1.363620
C	0.061185	0.166935	2.670757
C	0.900098	-1.966094	1.647823
H	0.009385	1.259948	2.563809
H	1.379914	-2.410611	0.763561
H	0.568351	-0.055627	3.622571
H	-0.964275	-0.228391	2.731774
H	-0.097028	-2.411017	1.754511
H	1.499441	-2.236380	2.531097
C	2.417523	2.990546	3.596681
H	1.749124	3.661345	3.037624
H	1.911868	2.023869	3.712716
H	2.572917	3.413500	4.597129
C	4.471133	4.136820	2.718248
H	4.707793	4.566035	3.699146
H	5.411363	3.996003	2.170020
H	3.853303	4.861953	2.167475
C	8.415312	-4.024125	-1.907716
H	9.077767	-4.224947	-1.057451
H	7.461727	-4.544185	-1.729407
H	8.869485	-4.454943	-2.808632
C	7.249967	-2.237339	-3.230851
H	6.275641	-2.723838	-3.073167
H	7.086078	-1.156779	-3.332548
H	7.663444	-2.612157	-4.175389
C	4.404165	5.328434	-1.160908
H	4.782869	6.039732	-1.909251
H	3.447236	4.928775	-1.520450
H	4.220537	5.887241	-0.234199
C	6.733918	4.748209	-0.420080
H	7.446539	3.931579	-0.256808
H	7.172058	5.447272	-1.147098
H	6.604115	5.289363	0.525465
C	5.774415	-4.726199	1.080008
H	5.730684	-5.804858	1.273903
H	6.824067	-4.442781	0.926842
H	5.404136	-4.205830	1.975106
C	3.463695	-4.638346	0.097822
H	3.291870	-5.704447	0.289651
H	3.101667	-4.075032	0.969399
H	2.868107	-4.345031	-0.775774
C	4.421064	-0.839386	3.879031
H	3.392969	-0.453795	3.917174
H	4.402100	-1.894684	4.186935
H	5.028450	-0.278315	4.600973
C	6.386271	-1.236412	2.356564
H	7.058643	-0.660278	3.003660
H	6.442911	-2.289722	2.664149
H	6.758084	-1.159459	1.326127
N	3.744382	0.312492	-1.999736
C	4.151343	0.237879	-3.429220
H	3.253496	0.257800	-4.052161
H	4.788968	1.096213	-3.657100
H	4.698532	-0.695293	-3.589188
C	4.966226	0.355420	-1.138639
H	5.567488	-0.540405	-1.324172

H	5.531550	1.255480	-1.398827
H	4.647318	0.385918	-0.093149
C	2.912228	-0.879057	-1.645591
H	2.553492	-0.734787	-0.617824
H	2.070442	-0.930115	-2.342192
H	3.538410	-1.772934	-1.718290
C	2.942291	1.552639	-1.769441
H	3.553506	2.413837	-2.057711
H	2.034118	1.496005	-2.377602
H	2.684776	1.588420	-0.706441

4-TMA-iso

E (BS1) = -3468.05720426

E (BS2) = -4608.827342

$G_{298.15,1M}$ (BS2) = -4607.652460

H	-1.211094	1.184599	1.787851
O	-1.716442	2.043922	1.706626
C	-2.763308	2.081602	2.667463
H	-2.853384	1.079912	3.125206
H	-6.566409	-3.562810	-2.222211
H	-2.075537	2.788444	0.092687
O	-2.392028	3.260231	-0.711740
O	-5.805685	-3.106124	-1.837004
C	-1.626569	4.446768	-0.908791
H	-2.237995	5.077364	-1.576581
C	-5.539740	-3.713276	-0.572537
H	-4.655986	-3.174155	-0.184562
Fe	4.967794	-1.268568	-0.861394
Ir	0.380635	-0.118147	-0.267039
P	2.452234	0.830322	0.329756
S	1.389010	-2.361824	-0.489473
C	3.873636	-0.298036	0.561055
C	3.812174	-1.727670	0.757123
C	5.150411	-2.195550	0.944023
H	5.428834	-3.236467	1.079288
C	6.037000	-1.085994	0.869879
H	7.119446	-1.127664	0.929051
C	5.258588	0.077120	0.626649
H	5.646107	1.080132	0.481463
C	4.406331	-2.447879	-2.434829
H	3.717423	-3.282945	-2.359150
C	4.048288	-1.090033	-2.678158
H	3.039533	-0.713251	-2.816328
C	5.239468	-0.305098	-2.640861
H	5.295973	0.773891	-2.751682
C	6.332768	-1.180473	-2.375505
H	7.368700	-0.885783	-2.245225
C	5.817923	-2.504021	-2.247004
H	6.394285	-3.390622	-2.004874
C	2.636632	-2.644633	0.839520
H	2.122293	-2.547497	1.804800
H	2.987210	-3.679134	0.738197
C	2.471963	1.791187	1.906146
C	1.611302	2.891591	1.998551
H	0.961047	3.139827	1.157955
C	1.576351	3.672502	3.148289
H	0.906712	4.529937	3.198674
C	2.391414	3.352651	4.233305
H	2.364596	3.960601	5.135238
C	3.237016	2.250211	4.158405
H	3.873458	1.992033	5.002618
C	3.280435	1.474875	3.000326
H	3.952622	0.618655	2.955601
C	3.150234	2.106415	-0.817191
C	4.095013	3.043877	-0.379612
H	4.414612	3.052549	0.662041
C	4.627508	3.981112	-1.259538
H	5.361046	4.700584	-0.901258
C	4.213040	4.006946	-2.589564
H	4.622890	4.746069	-3.274834
C	3.262776	3.091939	-3.032154
H	2.922036	3.112718	-4.065492
C	2.737187	2.149029	-2.151644
H	1.985356	1.441982	-2.498627
C	0.322399	-3.780881	-0.200118
C	0.831265	-5.057941	-0.455281
H	1.868370	-5.179186	-0.768063
C	0.009757	-6.171976	-0.325297
H	0.410859	-7.163454	-0.524481
C	-1.325783	-6.014578	0.040092
H	-1.975146	-6.883550	0.130172
C	-1.828974	-4.741570	0.286680
H	-2.868096	-4.611845	0.581946
C	-1.011291	-3.619191	0.171585
H	-1.411616	-2.625948	0.378456
H	-0.231724	1.355909	-0.359823
H	-1.119492	-0.591310	-0.749145
H	0.738364	0.043865	-1.812896
O	-3.014231	-1.157440	1.598382
C	-3.635891	-1.585458	2.796549
H	-3.081660	-1.178420	3.665063
H	-2.058692	-0.930879	1.760934
O	-5.093574	3.009269	-1.357650
C	-5.531740	4.208643	-1.980663
H	-6.605794	4.062854	-2.174541
H	-4.154164	3.132555	-1.101728
C	-3.640889	-3.101054	2.892969
H	-4.228623	-3.525425	2.064764
H	-2.624061	-3.508344	2.829918
H	-4.089807	-3.439274	3.835883
C	-5.049537	-1.038020	2.817615
H	-5.623853	-1.440391	3.661240
H	-5.056026	0.055944	2.885440
H	-5.563761	-1.320540	1.887354
C	-5.357482	5.390517	-1.046115
H	-5.871559	5.213034	-0.094125
H	-4.290025	5.553126	-0.834516
H	-5.756336	6.312199	-1.487122
C	-4.819760	4.422649	-3.303574
H	-3.736102	4.522436	-3.140415
H	-4.985231	3.575089	-3.980322
H	-5.170691	5.333518	-3.804421
C	-5.201121	-5.179149	-0.750882
H	-6.064798	-5.716413	-1.168578
H	-4.353350	-5.306739	-1.435032
H	-4.948650	-5.651177	0.207794
C	-6.711699	-3.507697	0.364965
H	-6.969600	-2.444201	0.439984
H	-7.593780	-4.046907	-0.009693
H	-6.492095	-3.883060	1.372719
C	-1.419601	5.181477	0.400620
H	-0.845361	6.103079	0.246779
H	-2.379958	5.448405	0.860418
H	-0.867317	4.546008	1.107799
C	-0.315659	4.136636	-1.605960
H	0.217220	5.059149	-1.870930
H	0.337265	3.538168	-0.955694

H	-0.492632	3.562659	-2.525584
C	-2.432641	3.092910	3.748933
H	-1.483370	2.846607	4.241668
H	-2.338844	4.097735	3.313030
H	-3.218214	3.128399	4.514544
C	-4.064930	2.416305	1.964930
H	-4.898954	2.477549	2.675671
H	-3.981587	3.384543	1.450644
H	-4.300830	1.652536	1.211682
N	-3.819948	-0.266419	-2.698013
C	-5.079785	-0.180020	-3.488669
H	-4.834469	-0.248284	-4.551779
H	-5.729060	-1.007498	-3.191329
H	-5.559296	0.778078	-3.269957
C	-4.146076	-0.168833	-1.240925
H	-4.639576	0.792267	-1.069381
H	-4.814855	-0.996199	-0.985169
H	-3.214122	-0.234359	-0.672293
C	-2.914452	0.858881	-3.074851
H	-1.989554	0.767619	-2.496750
H	-2.707274	0.795049	-4.146659
H	-3.419849	1.797339	-2.829897
C	-3.142310	-1.568621	-2.972869
H	-3.826244	-2.377802	-2.699435
H	-2.899983	-1.614646	-4.038229
H	-2.226810	-1.613513	-2.374708
O	-0.468414	-0.308283	1.850777
C	0.293505	-0.695328	2.955723
H	1.376043	-0.654211	2.704837
C	0.067090	0.252828	4.126959
H	0.679770	-0.021694	4.997430
H	0.311690	1.285377	3.844895
H	-0.989954	0.221880	4.435053
C	-0.020343	-2.123641	3.387015
H	0.657557	-2.462136	4.183512
H	-1.047746	-2.185262	3.775794
H	0.061673	-2.824782	2.545169

E. Enantioselectivity

TS$_{2\text{-}3}$-*pro-R*-iso
E (BS1) = -2474.251023
E (BS2) = -3614.685303
$G_{298.15,1M}$ (BS2) = -3614132997

O	3.862682	2.183977	-0.152336
C	3.395049	1.163654	0.444689
C	2.833351	1.378861	1.844687
H	2.257042	0.522502	2.218183
H	2.196395	2.270418	1.837949
H	3.664688	1.572595	2.539125
Fe	-3.468302	-0.066851	0.872401
Ir	0.712795	0.540222	-1.323791
P	-0.297131	-1.178820	-0.090622
S	-0.755544	2.257992	-0.348026
C	-1.551699	-0.700104	1.152177
C	-1.737214	0.617033	1.708873
C	-2.770548	0.530906	2.692862
H	-3.157786	1.372160	3.260009
C	-3.224826	-0.815328	2.758239
H	-4.033346	-1.187695	3.378431
C	-2.483965	-1.571568	1.810895
H	-2.625857	-2.624831	1.593625
C	-4.249112	1.388464	-0.334262
H	-3.877248	2.406336	-0.389233
C	-3.789274	0.288336	-1.115012
H	-3.005970	0.323981	-1.865871
C	-4.494900	-0.878133	-0.693858
H	-4.343894	-1.885699	-1.070103
C	-5.391849	-0.496041	0.346367
H	-6.041539	-1.161773	0.904709
C	-5.238933	0.903902	0.569387
H	-5.753884	1.488369	1.324413
C	-0.992810	1.880431	1.443138
H	0.007161	1.854984	1.898283
H	-1.548704	2.724209	1.871523
C	0.854367	-2.180668	0.944362
C	1.747022	-3.051696	0.306351
H	1.755182	-3.119260	-0.782464
C	2.606127	-3.851092	1.050405
H	3.283707	-4.534077	0.541369
C	2.607431	-3.771966	2.442543
H	3.281806	-4.397629	3.024139
C	1.754175	-2.880114	3.082849
H	1.756526	-2.803987	4.168527
C	0.877395	-2.091155	2.338234
H	0.199528	-1.411531	2.854650
C	-1.177853	-2.523919	-1.006054
C	-1.431902	-3.768737	-0.415006
H	-1.056046	-3.986686	0.584109
C	-2.154657	-4.743210	-1.095272
H	-2.343870	-5.703939	-0.620521
C	-2.625118	-4.493775	-2.383039
H	-3.185681	-5.258623	-2.916586
C	-2.361477	-3.268862	-2.987927
H	-2.712054	-3.070802	-3.998838
C	-1.640715	-2.292263	-2.303434
H	-1.428834	-1.335932	-2.780397
C	-0.031952	3.891512	-0.136547
C	-0.900193	4.961701	0.092371
H	-1.975717	4.790024	0.137544
C	-0.385336	6.242821	0.258072
H	-1.060415	7.077468	0.434969
C	0.990339	6.455179	0.187709
H	1.391503	7.458750	0.312997
C	1.848554	5.384745	-0.045914
H	2.924151	5.544152	-0.098993
C	1.342861	4.096676	-0.211774
H	2.023103	3.261084	-0.383858
H	1.562202	-0.570880	-2.132077
H	1.503849	1.627563	-2.295078
H	2.132895	0.734215	-0.179331
H	-0.393229	0.444234	-2.515887
C	4.170921	-0.126015	0.236937
C	4.573903	-0.935706	1.298000
C	4.607316	-0.446926	-1.053765
C	5.396080	-2.041634	1.076185
C	5.404371	-1.559910	-1.280957
C	5.809444	-2.360989	-0.210993
H	4.254250	-0.708289	2.313780
H	4.304490	0.192632	-1.882086
H	5.707025	-2.657841	1.918691
H	5.722804	-1.801341	-2.293670
H	6.444341	-3.227690	-0.385228

TS$_{2\text{-}3}$-*pro-S*-iso
E (BS1) = -2474.254772

E (BS2) = -3614.688723
$G_{298.15,1M}$ (BS2) = -3614.138261

O	-3.774402	2.354531	-0.960627
C	-2.765353	2.443800	-0.193131
C	-1.984027	3.752992	-0.234220
H	-1.024008	3.720022	0.291173
H	-1.798466	4.026668	-1.278817
H	-2.605538	4.537317	0.223518
Fe	1.874553	-2.284471	1.283915
Ir	-0.564142	0.625577	-1.746892
P	1.293024	0.787195	-0.305785
S	-1.203038	-1.551670	-0.805949
C	1.299675	-0.325337	1.145830
C	0.208118	-1.156173	1.596818
C	0.621824	-1.790113	2.809793
H	0.025807	-2.506124	3.368041
C	1.948915	-1.376768	3.112533
H	2.552089	-1.723269	3.945055
C	2.372221	-0.489167	2.087198
H	3.350526	-0.025154	2.013094
C	1.339152	-3.945024	0.219024
H	0.314358	-4.233762	0.008924
C	2.145338	-3.083629	-0.579784
H	1.843343	-2.607550	-1.506886
C	3.400096	-2.915455	0.078068
H	4.218924	-2.289118	-0.262458
C	3.368234	-3.675320	1.283076
H	4.155842	-3.724167	2.027632
C	2.094602	-4.310393	1.371183
H	1.745779	-4.925916	2.193618
C	-1.155148	-1.365527	1.030069
H	-1.820357	-0.525963	1.269187
H	-1.581707	-2.285490	1.451461
C	1.514564	2.451423	0.463679
C	1.710636	3.535466	-0.401959
H	1.744715	3.367007	-1.479031
C	1.857509	4.824822	0.094243
H	2.011596	5.653780	-0.593567
C	1.797022	5.055172	1.467904
H	1.902385	6.065332	1.857962
C	1.602680	3.987058	2.336834
H	1.556966	4.157329	3.410740
C	1.470416	2.690624	1.838969
H	1.320394	1.865596	2.534891
C	2.994458	0.539621	-0.993533
C	4.125739	1.060111	-0.351671
H	4.014591	1.670404	0.544036
C	5.400512	0.819616	-0.854615
H	6.268173	1.233825	-0.344953
C	5.564570	0.062189	-2.012691
H	6.561407	-0.119907	-2.408949
C	4.446944	-0.445808	-2.667772
H	4.564146	-1.025865	-3.580969
C	3.171429	-0.203955	-2.162467
H	2.295993	-0.587781	-2.685547
C	-2.958684	-1.934605	-0.931955
C	-3.339452	-3.270053	-0.782309
H	-2.582254	-4.033522	-0.604179
C	-4.683539	-3.618637	-0.864477
H	-4.979754	-4.659281	-0.750879
C	-5.643589	-2.636476	-1.098598
H	-6.694515	-2.909672	-1.166900
C	-5.257642	-1.307352	-1.246183
H	-6.004691	-0.535588	-1.424506
C	-3.913894	-0.947658	-1.164771
H	-3.622433	0.098479	-1.271082
H	-0.095685	1.986715	-2.478565
H	-1.787736	0.639989	-2.859616
H	-1.688903	1.577363	-0.664554
H	0.285557	-0.184716	-2.877259
C	-2.902513	1.824398	1.194208
C	-4.076315	1.136680	1.507859
C	-1.908305	1.920755	2.171939
C	-4.240867	0.530491	2.750708
C	-2.067644	1.320361	3.416683
C	-3.233917	0.614100	3.709304
H	-4.858344	1.089313	0.753201
H	-0.984905	2.451970	1.952340
H	-5.161632	-0.006273	2.973312
H	-1.275258	1.402871	4.160381
H	-3.360015	0.141105	4.681328

TS_{2-3}-*pro-R*-Li-iso
E (BS1) = -3453.040983
E (BS2) = -4593.791006
$G_{298.15,1M}$ (BS2) = -4592.728939

Li	3.533683	-0.357642	1.007764
H	4.076740	1.924275	-0.146146
O	4.904283	2.390426	0.088298
C	5.791418	2.282369	-1.030026
H	6.207175	1.255596	-1.054061
H	1.891188	-2.065346	1.251255
H	1.550700	0.653836	1.837840
O	2.415821	0.716122	2.297486
O	2.844084	-2.169885	1.426842
C	2.148045	0.542084	3.694256
H	1.364730	-0.229779	3.799401
C	3.024716	-3.310461	2.276986
H	3.965086	-3.121701	2.817487
Fe	-5.102612	-1.431618	-0.712582
Ir	-0.774223	-0.144167	0.748867
P	-2.712414	0.997222	0.083972
S	-1.453962	-2.196109	-0.420514
C	-3.838960	0.126658	-1.063407
C	-3.499539	-0.995979	-1.900405
C	-4.644090	-1.308043	-2.696946
H	-4.703882	-2.132956	-3.400609
C	-5.683815	-0.392919	-2.373485
H	-6.690449	-0.400283	-2.777878
C	-5.195620	0.485126	-1.368982
H	-5.766600	1.269492	-0.883830
C	-4.817792	-3.251048	0.179076
H	-4.019698	-3.941331	-0.073252
C	-4.744038	-2.206111	1.145482
H	-3.878196	-1.960984	1.753165
C	-5.983241	-1.499188	1.127167
H	-6.226593	-0.623263	1.721291
C	-6.822032	-2.110116	0.149672
H	-7.815805	-1.780045	-0.133978
C	-6.101502	-3.191625	-0.436944
H	-6.452995	-3.829357	-1.241049
C	-2.202484	-1.715346	-2.037452
H	-1.455115	-1.103472	-2.564104
H	-2.358706	-2.642944	-2.602575
C	-2.450296	2.570416	-0.837412
C	-2.037949	3.710271	-0.136019

H	-1.866712	3.653939	0.939888
C	-1.873520	4.921832	-0.796126
H	-1.569295	5.804165	-0.236231
C	-2.095796	5.007273	-2.169833
H	-1.971213	5.958369	-2.683976
C	-2.461655	3.870574	-2.881885
H	-2.625404	3.926350	-3.956342
C	-2.640595	2.656963	-2.218969
H	-2.948289	1.777843	-2.785390
C	-3.886676	1.555037	1.400386
C	-4.835569	2.558853	1.164253
H	-4.853360	3.081977	0.208824
C	-5.757797	2.907885	2.145385
H	-6.488645	3.688644	1.944798
C	-5.739371	2.267787	3.383161
H	-6.458025	2.545276	4.151475
C	-4.789098	1.283684	3.635859
H	-4.758305	0.787884	4.603980
C	-3.868731	0.932168	2.650687
H	-3.122053	0.164474	2.849901
C	-0.131573	-3.217324	-1.090085
C	-0.454823	-4.524689	-1.463620
H	-1.471853	-4.892337	-1.327612
C	0.522438	-5.348902	-2.011584
H	0.268601	-6.365909	-2.302600
C	1.821423	-4.873217	-2.180977
H	2.586270	-5.519923	-2.606113
C	2.143464	-3.576399	-1.792983
H	3.158147	-3.193837	-1.891622
C	1.168581	-2.745868	-1.243634
H	1.419261	-1.735238	-0.923187
H	-0.414660	1.129663	1.691592
H	0.574480	-0.903626	1.326453
H	0.278119	0.624010	-0.500557
H	-1.503811	-0.803117	2.064697
O	4.467286	-1.315064	-1.330914
C	5.392639	-1.310126	-2.412998
H	6.096853	-0.463022	-2.290587
H	3.830629	-0.581660	-1.430593
O	5.388878	0.083319	1.515407
C	6.587341	-0.616168	1.154301
H	6.642836	-0.672479	0.051938
H	5.437473	1.003319	1.176518
C	1.763069	1.196574	-1.244421
O	2.714850	0.661135	-0.592999
C	1.413617	0.619811	-2.590268
H	1.503119	-0.469875	-2.572138
H	2.141608	1.003653	-3.323163
H	0.409334	0.899351	-2.924234
C	1.539204	2.664802	-1.043840
C	1.801187	3.247253	0.203386
C	1.248094	3.497679	-2.128979
C	1.792322	4.626620	0.355663
C	1.261579	4.882283	-1.978129
C	1.541273	5.450932	-0.740676
H	2.026926	2.608701	1.055835
H	1.036910	3.076206	-3.109151
H	1.998545	5.061341	1.331816
H	1.054093	5.516939	-2.837473
H	1.554213	6.532933	-0.626072
C	4.673178	-1.152599	-3.736845
H	5.384714	-1.152526	-4.571499
H	3.961931	-1.976125	-3.890224
H	4.115146	-0.206926	-3.772610
C	6.173542	-2.602165	-2.334586
H	6.667580	-2.704609	-1.360328
H	5.504563	-3.463170	-2.470523
H	6.943226	-2.639240	-3.114008
C	5.051114	2.541172	-2.324553
H	5.725933	2.448357	-3.184528
H	4.231112	1.821775	-2.456665
H	4.625527	3.554624	-2.321931
C	6.912295	3.268810	-0.805321
H	7.407014	3.094013	0.157773
H	7.664486	3.187762	-1.598020
H	6.517107	4.293144	-0.804457
C	7.796740	0.121131	1.684862
H	7.760310	0.175973	2.780950
H	8.721786	-0.390885	1.394607
H	7.839713	1.145259	1.290928
C	6.473164	-2.012957	1.714023
H	7.365805	-2.600123	1.468661
H	6.378082	-1.976783	2.808532
H	5.598027	-2.530399	1.301396
C	1.881008	-3.406885	3.262588
H	0.931109	-3.553526	2.725020
H	2.019042	-4.258023	3.939207
H	1.796954	-2.494200	3.865774
C	3.168001	-4.563043	1.439388
H	3.400454	-5.428283	2.072122
H	2.231882	-4.772464	0.901273
H	3.970516	-4.449476	0.698816
C	3.415436	0.061132	4.359569
H	3.246809	-0.106091	5.429821
H	3.761318	-0.880680	3.913002
H	4.212545	0.809216	4.251635
C	1.644655	1.846767	4.275879
H	0.732353	2.176565	3.761738
H	1.417000	1.741536	5.343715
H	2.406768	2.629751	4.159135

TS$_{2-3}$-*pro-S*-Li-iso

E (BS1) = -3453.047616

E (BS2) = -4593.799152

G$_{298.15,1M}$ (BS2) =-4592.734967

Li	3.738459	-0.252639	-0.917098
H	3.713827	-1.910513	1.349905
O	4.496819	-2.470583	1.535734
C	5.274827	-1.762805	2.506373
H	5.527187	-0.763903	2.099716
H	3.123041	0.414773	-3.312762
H	1.662184	-1.503165	-1.278072
O	2.562975	-1.822705	-1.503495
O	3.580538	0.835500	-2.570739
C	2.448228	-2.572594	-2.717504
H	1.829945	-1.993563	-3.427203
C	3.652315	2.252740	-2.806207
H	3.834426	2.681303	-1.810109
Fe	-5.001018	1.301124	0.496360
Ir	-0.667481	-0.257783	-1.081541
P	-2.627296	-1.203210	-0.186243
S	-1.462228	2.000101	-0.556339
C	-3.607359	-0.132531	0.927740
C	-3.211753	1.150392	1.458390
C	-4.233619	1.578863	2.361508
H	-4.234928	2.527986	2.889415
C	-5.256602	0.590658	2.394756
H	-6.185523	0.651058	2.951817

C	-4.881866	-0.453597	1.507595
H	-5.468697	-1.339610	1.287489
C	-4.940622	2.938607	-0.726365
H	-4.141505	3.672780	-0.726772
C	-4.977964	1.743824	-1.501381
H	-4.216720	1.414075	-2.200684
C	-6.166535	1.032245	-1.161313
H	-6.465952	0.066461	-1.556584
C	-6.864220	1.790281	-0.176346
H	-7.785232	1.500184	0.318214
C	-6.106162	2.967603	0.093235
H	-6.350315	3.728216	0.827195
C	-1.985213	1.960864	1.211860
H	-1.127233	1.575710	1.776906
H	-2.170253	3.000872	1.512992
C	-2.338433	-2.688116	0.867886
C	-1.798488	-3.824029	0.250783
H	-1.609771	-3.816296	-0.823519
C	-1.499564	-4.960734	0.991582
H	-1.083336	-5.835221	0.495375
C	-1.729221	-4.977815	2.367115
H	-1.490650	-5.865052	2.949734
C	-2.268855	-3.857360	2.989235
H	-2.456534	-3.864162	4.061201
C	-2.577632	-2.719285	2.243306
H	-3.000884	-1.850135	2.746613
C	-3.928087	-1.866856	-1.324626
C	-4.857219	-2.825189	-0.899910
H	-4.791495	-3.244384	0.103635
C	-5.865657	-3.262064	-1.753229
H	-6.578458	-4.008218	-1.407925
C	-5.956344	-2.753514	-3.047451
H	-6.742255	-3.099338	-3.715697
C	-5.028689	-1.813211	-3.485383
H	-5.083523	-1.421112	-4.498970
C	-4.019597	-1.377182	-2.629571
H	-3.283965	-0.652399	-2.977831
C	-0.168673	3.236360	-0.371363
C	-0.520788	4.570407	-0.584500
H	-1.543366	4.822592	-0.863506
C	0.436430	5.570387	-0.441778
H	0.162975	6.608922	-0.615197
C	1.737993	5.238176	-0.074880
H	2.486100	6.019595	0.042226
C	2.083915	3.906308	0.138147
H	3.098929	3.629079	0.416514
C	1.136524	2.897872	-0.022808
H	1.411703	1.849802	0.104832
H	-0.229076	-1.737059	-1.565479
H	0.733443	0.321516	-1.743220
H	0.249194	-0.486125	0.436506
H	-1.295737	-0.064307	-2.590025
O	4.227019	1.492773	0.384165
C	5.475758	1.853019	0.976402
H	6.177875	1.003980	0.869282
H	3.711847	0.942616	1.006320
O	5.457382	-1.240497	-0.692328
C	6.870163	-1.141076	-0.865467
H	7.325208	-0.837157	0.096032
H	5.261553	-1.895819	0.012146
C	1.463177	-0.817607	1.610255
O	2.574842	-0.486388	1.094843
C	1.147741	-2.288648	1.689827
H	1.501807	-2.788336	0.781764

H	0.085372	-2.500065	1.820382
H	1.694321	-2.703794	2.551504
C	0.866213	0.084112	2.651871
C	-0.238108	-0.299391	3.422338
C	1.446707	1.333625	2.892155
C	-0.766032	0.558449	4.380900
C	0.918692	2.194082	3.848176
C	-0.194640	1.812232	4.592768
H	-0.703151	-1.270161	3.267192
H	2.314297	1.638797	2.313141
H	-1.630567	0.244672	4.963718
H	1.381900	3.165672	4.011191
H	-0.610328	2.483751	5.340983
C	5.296121	2.158610	2.448631
H	4.623426	3.017263	2.585082
H	4.868585	1.298307	2.983266
H	6.258168	2.400557	2.916027
C	6.025383	3.037121	0.213273
H	7.041427	3.272321	0.551098
H	6.062234	2.833066	-0.864762
H	5.399184	3.926042	0.370173
C	7.440459	-2.481333	-1.280754
H	7.181827	-3.263633	-0.555053
H	7.041533	-2.776820	-2.260581
H	8.533679	-2.434685	-1.354400
C	7.127938	-0.068037	-1.895480
H	6.682386	-0.351481	-2.859059
H	6.693931	0.889693	-1.581292
H	8.204417	0.073774	-2.044765
C	1.779170	-3.903660	-2.438367
H	1.671143	-4.489736	-3.359388
H	2.384319	-4.483864	-1.727982
H	0.781173	-3.761961	-2.004319
C	3.832719	-2.757256	-3.293323
H	4.317389	-1.794031	-3.493294
H	4.461263	-3.323748	-2.592456
H	3.780673	-3.317738	-4.233879
C	4.488853	-1.588601	3.788665
H	3.567455	-1.017248	3.610570
H	4.213967	-2.569107	4.200258
H	5.080670	-1.052864	4.541147
C	6.549448	-2.546326	2.709355
H	6.319957	-3.549909	3.091086
H	7.097893	-2.655653	1.764478
H	7.204068	-2.044310	3.430552
C	2.338923	2.771486	-3.348661
H	2.136187	2.341801	-4.340444
H	1.505889	2.515422	-2.682589
H	2.371654	3.863029	-3.454613
C	4.815195	2.568616	-3.722100
H	5.757520	2.181301	-3.315203
H	4.657058	2.118352	-4.711928
H	4.919167	3.652329	-3.856075

$TS_{2\text{-}3}$-*pro*-*R*-Na-iso
E (BS1) = -3607.776321
E (BS2) = -4748.537417
$G_{298.15,1M}$ (BS2) = -4747.480993

Na	3.690800	-0.533070	0.785565
H	3.926235	1.865789	-0.082597
O	4.804995	2.167123	0.227344
C	5.657506	2.259801	-0.919952
H	5.931768	1.236399	-1.235284

H	1.759455	-2.086393	1.593634
H	1.507399	0.610829	1.858668
O	2.395365	0.717439	2.261067
O	2.676117	-2.403927	1.704376
C	2.244101	0.587852	3.675533
H	1.527540	-0.228861	3.879279
C	2.622110	-3.598321	2.489736
H	3.673729	-3.880011	2.645673
Fe	-5.056642	-1.550777	-0.807458
Ir	-0.835628	-0.132303	0.773611
P	-2.801919	0.960285	0.125081
S	-1.371645	-2.128336	-0.553744
C	-3.878482	0.088851	-1.068633
C	-3.478078	-0.965332	-1.966137
C	-4.604165	-1.294942	-2.781486
H	-4.618986	-2.081539	-3.529958
C	-5.692211	-0.457040	-2.411298
H	-6.697177	-0.497497	-2.817859
C	-5.252730	0.389365	-1.358278
H	-5.865428	1.113428	-0.831982
C	-4.669089	-3.386690	0.010147
H	-3.824395	-4.014131	-0.254154
C	-4.680336	-2.383080	1.022153
H	-3.843121	-2.109572	1.657470
C	-5.962023	-1.756263	1.008395
H	-6.272699	-0.924745	1.634244
C	-6.742285	-2.376059	-0.011360
H	-7.749941	-2.097433	-0.301120
C	-5.942828	-3.382123	-0.629010
H	-6.237978	-4.005022	-1.466668
C	-2.140873	-1.598061	-2.143420
H	-1.433114	-0.911336	-2.630129
H	-2.241343	-2.496653	-2.764909
C	-2.592566	2.577296	-0.732329
C	-2.168118	3.685963	0.011091
H	-1.945600	3.574052	1.073403
C	-2.062889	4.935999	-0.586405
H	-1.752746	5.793409	0.007820
C	-2.349563	5.091124	-1.942078
H	-2.268430	6.071573	-2.407448
C	-2.723767	3.986887	-2.698835
H	-2.937143	4.097110	-3.760293
C	-2.849396	2.735126	-2.096430
H	-3.167821	1.883104	-2.696668
C	-4.014149	1.421377	1.443337
C	-4.988267	2.406289	1.233850
H	-5.005134	2.970089	0.301668
C	-5.938862	2.682016	2.211385
H	-6.689882	3.448701	2.032231
C	-5.923547	1.986066	3.418577
H	-6.664902	2.205383	4.184097
C	-4.948049	1.020376	3.645441
H	-4.920179	0.481768	4.590514
C	-3.998829	0.742388	2.664161
H	-3.231947	-0.010429	2.842571
C	0.029726	-3.021058	-1.245104
C	-0.207435	-4.304454	-1.746114
H	-1.217161	-4.714679	-1.731529
C	0.846404	-5.055442	-2.254905
H	0.658354	-6.052580	-2.646967
C	2.139793	-4.535460	-2.247074
H	2.967868	-5.126186	-2.634427
C	2.374028	-3.262884	-1.735610
H	3.378866	-2.845733	-1.713265
C	1.319413	-2.497657	-1.240290
H	1.492104	-1.494970	-0.846205
H	-0.551711	1.075036	1.816849
H	0.541112	-0.864387	1.332634
H	0.162289	0.752552	-0.425601
H	-1.576238	-0.925748	2.010856
O	4.525007	-0.824188	-1.597367
C	5.227005	-0.921959	-2.836262
H	5.895354	-0.044589	-2.936448
H	3.782076	-0.196712	-1.698828
O	5.840484	-0.115498	1.555250
C	6.861662	-0.851983	0.873649
H	6.830787	-0.590598	-0.200039
H	5.806401	0.800821	1.221050
C	1.531846	1.387821	-1.304661
O	2.545343	0.827109	-0.791017
C	1.033077	0.880086	-2.630858
H	1.103825	-0.211765	-2.663764
H	1.690466	1.282201	-3.418586
H	0.005385	1.191459	-2.846319
C	1.330599	2.838152	-0.985866
C	1.638706	3.316855	0.294243
C	1.021184	3.753342	-1.994620
C	1.641511	4.679583	0.556181
C	1.048227	5.122322	-1.735009
C	1.362024	5.589385	-0.464111
H	1.884283	2.607943	1.083347
H	0.782514	3.408737	-2.998752
H	1.876784	5.034787	1.557853
H	0.822559	5.824535	-2.535257
H	1.381557	6.658720	-0.263728
C	4.255619	-0.921885	-3.998074
H	4.791192	-0.970774	-4.953580
H	3.576064	-1.783118	-3.938091
H	3.650045	-0.004701	-3.999955
C	6.077772	-2.170532	-2.792116
H	6.756490	-2.154942	-1.930316
H	5.448934	-3.068980	-2.720844
H	6.684830	-2.250432	-3.701244
C	4.942658	2.950146	-2.061857
H	5.596349	3.029280	-2.939230
H	4.043463	2.391555	-2.356600
H	4.636527	3.963123	-1.763899
C	6.902289	2.996105	-0.486492
H	7.377873	2.493118	0.365880
H	7.629787	3.044751	-1.304558
H	6.652414	4.020888	-0.181714
C	8.223723	-0.513355	1.440992
H	8.272118	-0.789622	2.502681
H	9.014792	-1.054483	0.907819
H	8.431689	0.560606	1.356800
C	6.523715	-2.317685	1.037528
H	7.302960	-2.950378	0.596498
H	6.446293	-2.566922	2.105250
H	5.570740	-2.573429	0.552067
C	1.963150	-3.316108	3.824282
H	0.921840	-2.996195	3.668212
H	1.949187	-4.214504	4.452871
H	2.489667	-2.522258	4.368098
C	1.905001	-4.693181	1.727022
H	1.896321	-5.628809	2.298921
H	0.859966	-4.399704	1.542670
H	2.386272	-4.882486	0.759976
C	3.595751	0.219964	4.241595

H	3.545727	0.111567	5.331590
H	3.953973	-0.727608	3.816792
H	4.329911	1.003615	4.007693
C	1.705420	1.880658	4.253603
H	0.736994	2.135057	3.802978
H	1.568080	1.803074	5.339197
H	2.404861	2.703921	4.050680

$TS_{2\text{-}3}$-*pro-S*-Na-iso

E (BS1) = -3607,782954

E (BS2) = -4748.546010

$G_{298.15,1M}$ (BS2) = -4747.485999

Na	3.330600	-0.299108	-0.937642
H	3.636324	-1.915137	1.481697
O	4.444036	-2.442611	1.661881
C	5.269297	-1.626544	2.497210
H	5.470622	-0.673582	1.968586
H	3.034425	0.520624	-3.745361
H	1.424166	-2.034089	-1.098252
O	2.315398	-2.366736	-1.336892
O	3.498972	0.823871	-2.952668
C	2.162892	-3.078344	-2.567696
H	1.494740	-2.494636	-3.226911
C	3.602235	2.255040	-3.004580
H	3.819371	2.551727	-1.965265
Fe	-5.007373	1.394006	0.415359
Ir	-0.654854	-0.340503	-0.986919
P	-2.673232	-1.183725	-0.135424
S	-1.426760	1.954954	-0.613218
C	-3.646763	-0.048712	0.917824
C	-3.232970	1.245590	1.404582
C	-4.257271	1.726775	2.277985
H	-4.247536	2.695518	2.768910
C	-5.299281	0.759714	2.335126
H	-6.233774	0.859127	2.877043
C	-4.934639	-0.323786	1.491958
H	-5.534967	-1.206897	1.297976
C	-4.898476	2.982170	-0.867323
H	-4.081126	3.695702	-0.890083
C	-4.962692	1.761029	-1.598211
H	-4.207405	1.388033	-2.282072
C	-6.170417	1.092310	-1.239213
H	-6.492965	0.121048	-1.601445
C	-6.853591	1.903897	-0.287255
H	-7.784150	1.655764	0.212240
C	-6.067296	3.070799	-0.056548
H	-6.295850	3.863631	0.647749
C	-1.988806	2.023511	1.141499
H	-1.149118	1.659948	1.746986
H	-2.164674	3.081408	1.378668
C	-2.445080	-2.638681	0.973249
C	-1.927421	-3.810692	0.406449
H	-1.731440	-3.850819	-0.665643
C	-1.662782	-4.923731	1.194720
H	-1.262290	-5.826396	0.737228
C	-1.907481	-4.881650	2.567195
H	-1.696030	-5.750715	3.186505
C	-2.427099	-3.725452	3.138926
H	-2.626579	-3.685830	4.208051
C	-2.699407	-2.610192	2.345849
H	-3.106461	-1.712075	2.809809
C	-3.958347	-1.843871	-1.289435
C	-4.925919	-2.761521	-0.860935
H	-4.897588	-3.152220	0.155734
C	-5.925030	-3.193676	-1.727428
H	-6.668416	-3.908191	-1.379792
C	-5.967933	-2.720082	-3.037298
H	-6.747166	-3.061701	-3.715413
C	-5.001496	-1.821042	-3.478237
H	-5.019270	-1.457730	-4.503756
C	-4.000672	-1.390622	-2.609886
H	-3.233923	-0.699473	-2.959486
C	-0.126092	3.188159	-0.470387
C	-0.454466	4.510708	-0.773155
H	-1.465147	4.757753	-1.096336
C	0.511864	5.506253	-0.661766
H	0.257209	6.535733	-0.903950
C	1.798148	5.181482	-0.238378
H	2.553421	5.959345	-0.145324
C	2.119858	3.860514	0.063191
H	3.121258	3.588911	0.389609
C	1.163867	2.856579	-0.064577
H	1.416152	1.815738	0.144322
H	-0.272161	-1.863229	-1.380305
H	0.800527	0.158109	-1.617760
H	0.188136	-0.531737	0.585379
H	-1.226836	-0.186076	-2.519689
O	4.266439	1.459319	0.517567
C	5.501819	1.948778	1.043907
H	6.253551	1.136545	1.006040
H	3.831530	0.883761	1.174652
O	5.325302	-1.520896	-0.780302
C	6.700009	-1.163978	-0.870805
H	7.034613	-0.763607	0.105814
H	5.177751	-2.076113	0.013137
C	1.360875	-0.802314	1.788567
O	2.486834	-0.518173	1.274818
C	1.029689	-2.262899	1.960820
H	1.372863	-2.823455	1.084017
H	-0.033752	-2.458151	2.112076
H	1.580375	-2.627591	2.842529
C	0.766609	0.172900	2.764289
C	-0.367167	-0.129455	3.527383
C	1.382763	1.415362	2.944352
C	-0.886824	0.799545	4.422289
C	0.862319	2.347284	3.835505
C	-0.278819	2.044851	4.574556
H	-0.861533	-1.091835	3.416347
H	2.272570	1.653721	2.366736
H	-1.773398	0.548477	5.002207
H	1.353243	3.312000	3.952449
H	-0.688164	2.771747	5.272867
C	5.327016	2.390148	2.481985
H	4.595262	3.208045	2.546515
H	4.974037	1.561966	3.112505
H	6.276829	2.747397	2.897709
C	5.970760	3.080613	0.157236
H	6.971530	3.408943	0.461105
H	6.019128	2.772049	-0.894594
H	5.294595	3.943204	0.229920
C	7.539656	-2.374074	-1.226054
H	7.424855	-3.170918	-0.479937
H	7.226323	-2.772725	-2.200885
H	8.603647	-2.113594	-1.285023
C	6.802485	-0.075192	-1.913598
H	6.519262	-0.468241	-2.900394
H	6.124710	0.753218	-1.669833

H	7.824884	0.315407	-1.978770
C	1.542237	-4.434688	-2.300214
H	1.374584	-4.986876	-3.233136
H	2.204932	-5.031241	-1.658416
H	0.577114	-4.327242	-1.787181
C	3.523097	-3.190786	-3.216402
H	3.947132	-2.200060	-3.424352
H	4.216653	-3.731470	-2.558059
H	3.451756	-3.739691	-4.162726
C	4.571934	-1.328996	3.808944
H	3.634213	-0.781807	3.643355
H	4.337532	-2.266140	4.331818
H	5.210448	-0.722578	4.463486
C	6.571681	-2.364188	2.698040
H	6.391555	-3.314324	3.218209
H	7.053012	-2.586928	1.737072
H	7.266663	-1.768381	3.300581
C	2.289606	2.868125	-3.439848
H	2.053145	2.567124	-4.470721
H	1.468375	2.546585	-2.786869
H	2.343485	3.963473	-3.413797
C	4.752916	2.664161	-3.899585
H	5.690610	2.193517	-3.578724
H	4.555234	2.362207	-4.937567
H	4.890636	3.752384	-3.883648

$TS_{2\text{-}3}$-*pro*-*R*-K-iso
E (BS1) = -4045.370865
E (BS2) = -5186.158493
$G_{298.15,1M}$ (BS2) = -5185.100463

K	3.706213	-1.139863	0.844029
H	3.986933	1.612125	-0.076162
O	4.853389	1.947478	0.239069
C	5.645751	2.299420	-0.897738
H	6.088646	1.377284	-1.322144
H	1.305615	-2.106294	2.134017
H	1.524398	0.527025	2.003868
O	2.396713	0.543905	2.447129
O	2.063809	-2.694886	2.315426
C	2.162342	0.464581	3.852269
H	1.337859	-0.249710	4.033187
C	1.547508	-3.849020	2.980748
H	2.428632	-4.470508	3.196787
Fe	-5.015823	-1.395400	-1.165430
Ir	-0.870484	-0.081985	0.804232
P	-2.821461	1.016512	0.115822
S	-1.387874	-2.052257	-0.560077
C	-3.781736	0.225850	-1.226606
C	-3.321085	-0.797889	-2.132201
C	-4.369794	-1.053911	-3.068940
H	-4.331257	-1.804982	-3.852321
C	-5.468935	-0.204745	-2.763467
H	-6.429315	-0.196315	-3.267839
C	-5.114694	0.575859	-1.631014
H	-5.757292	1.293481	-1.132067
C	-4.752691	-3.295386	-0.455303
H	-3.911367	-3.933636	-0.704994
C	-4.804153	-2.364271	0.622365
H	-4.007554	-2.170326	1.334095
C	-6.059994	-1.689266	0.563578
H	-6.385605	-0.891285	1.224398
C	-6.783780	-2.206520	-0.550110
H	-7.757719	-1.870179	-0.889639
C	-5.975578	-3.197746	-1.180452
H	-6.229225	-3.748767	-2.079849
C	-2.002088	-1.488224	-2.203807
H	-1.226725	-0.837298	-2.631551
H	-2.093959	-2.383577	-2.832438
C	-2.578261	2.704140	-0.582763
C	-2.212339	3.741081	0.285040
H	-2.060574	3.533968	1.345200
C	-2.066717	5.038688	-0.189503
H	-1.794384	5.837379	0.497950
C	-2.264187	5.316851	-1.541447
H	-2.154290	6.334761	-1.910419
C	-2.589078	4.287351	-2.417186
H	-2.734631	4.494128	-3.475703
C	-2.747970	2.986166	-1.940353
H	-3.023058	2.192329	-2.634332
C	-4.141849	1.352287	1.367431
C	-5.104758	2.350564	1.170827
H	-5.053344	2.996565	0.295065
C	-6.130824	2.537009	2.091477
H	-6.871011	3.316645	1.923361
C	-6.204751	1.735312	3.228844
H	-7.004781	1.885210	3.950823
C	-5.243173	0.752783	3.443489
H	-5.285488	0.130844	4.335391
C	-4.218022	0.564918	2.518839
H	-3.461723	-0.200850	2.688638
C	0.034520	-2.988623	-1.140851
C	-0.182913	-4.316252	-1.514457
H	-1.188079	-4.734526	-1.463606
C	0.884547	-5.102313	-1.938218
H	0.712377	-6.136768	-2.226951
C	2.168893	-4.565514	-1.981614
H	3.006592	-5.178580	-2.308431
C	2.381773	-3.239368	-1.612505
H	3.380057	-2.806159	-1.670131
C	1.315847	-2.444212	-1.192575
H	1.469782	-1.400991	-0.909613
H	-0.589782	1.116457	1.858174
H	0.509677	-0.808444	1.348063
H	0.141100	0.799742	-0.378270
H	-1.626389	-0.883163	2.030277
O	4.538526	-0.760086	-1.775017
C	5.070611	-0.846951	-3.094624
H	5.709080	0.038527	-3.281696
H	3.786664	-0.134364	-1.769473
O	6.230601	-0.310080	1.290387
C	7.161219	-0.841745	0.345176
H	6.812537	-0.598556	-0.674336
H	5.974541	0.592456	1.016714
C	1.524296	1.412512	-1.211342
O	2.515419	0.760845	-0.763359
C	0.984144	1.036408	-2.567051
H	1.021163	-0.050659	-2.693381
H	1.637331	1.485596	-3.332498
H	-0.038061	1.392775	-2.734410
C	1.394890	2.841905	-0.778285
C	1.796511	3.213395	0.511157
C	1.040238	3.838937	-1.690020
C	1.839025	4.550956	0.878881
C	1.110490	5.182829	-1.327463
C	1.510038	5.543174	-0.045741
H	2.076468	2.441899	1.226868
H	0.724090	3.578613	-2.698215

H	2.142605	4.822426	1.888309
H	0.848982	5.949334	-2.054458
H	1.560451	6.592736	0.236984
C	3.955275	-0.866851	-4.119429
H	4.359947	-0.923810	-5.137006
H	3.299276	-1.733988	-3.959150
H	3.343804	0.043010	-4.052423
C	5.932379	-2.087320	-3.164268
H	6.728551	-2.060679	-2.410396
H	5.326408	-2.988596	-2.994749
H	6.402072	-2.172455	-4.151000
C	4.803374	2.964820	-1.965545
H	5.422235	3.240598	-2.828079
H	4.009371	2.292245	-2.317588
H	4.333152	3.876475	-1.570593
C	6.751769	3.201201	-0.400803
H	7.297404	2.731931	0.427807
H	7.465965	3.422023	-1.202341
H	6.333046	4.148912	-0.036722
C	8.537989	-0.248917	0.562795
H	8.898612	-0.484701	1.573030
H	9.256544	-0.649808	-0.162738
H	8.519335	0.842636	0.451056
C	7.160325	-2.345278	0.511562
H	7.914403	-2.809000	-0.135254
H	7.393241	-2.612115	1.551375
H	6.187738	-2.785244	0.249914
C	0.879282	-3.445967	4.279626
H	0.019101	-2.792136	4.071790
H	0.511386	-4.323928	4.824308
H	1.575550	-2.904119	4.931213
C	0.604771	-4.609723	2.070175
H	0.256263	-5.533693	2.547677
H	-0.279034	-3.994240	1.842269
H	1.095556	-4.872406	1.124402
C	3.426826	-0.065581	4.488744
H	3.317294	-0.137384	5.577338
H	3.667667	-1.064968	4.103872
H	4.269344	0.606433	4.273738
C	1.769710	1.826315	4.388270
H	0.867645	2.198175	3.884511
H	1.567982	1.786743	5.465954
H	2.581234	2.547061	4.214477

TS$_{2\text{-}3}$-*pro*-*S*-K-iso

E (BS1) = -4045.372136

E (BS2) = -5186,160368

$G_{298.15,1M}$ (BS2) = -5185.102402

K	3.473775	-0.524616	-0.871130
H	3.352756	-1.966834	1.449262
O	4.146421	-2.533983	1.372598
C	5.175215	-1.896029	2.122416
H	5.463367	-0.957542	1.610565
H	3.515077	0.218111	-4.235837
H	1.270249	-2.493306	-1.064622
O	2.131190	-2.911845	-1.268420
O	3.709961	0.526954	-3.340321
C	2.211287	-3.040929	-2.687731
H	2.044154	-2.048242	-3.151995
C	3.333751	1.905426	-3.258318
H	3.419446	2.154875	-2.186202
Fe	-4.963790	1.639325	0.125724
Ir	-0.701690	-0.531610	-0.834853
P	-2.816470	-1.142448	-0.012991
S	-1.307216	1.842635	-0.745773
C	-3.752150	0.162884	0.859598
C	-3.262192	1.468665	1.230041
C	-4.287771	2.117311	1.985496
H	-4.225047	3.130379	2.371982
C	-5.405685	1.242515	2.081169
H	-6.355655	1.469485	2.553385
C	-5.085888	0.048852	1.380422
H	-5.744301	-0.803105	1.244298
C	-4.672580	3.086415	-1.288171
H	-3.815643	3.751786	-1.314652
C	-4.754659	1.804227	-1.903549
H	-3.973285	1.326687	-2.485891
C	-6.022492	1.236949	-1.577474
H	-6.373894	0.252146	-1.870019
C	-6.723789	2.171593	-0.761788
H	-7.700827	2.021593	-0.314740
C	-5.889537	3.313776	-0.581830
H	-6.121983	4.182707	0.024691
C	-1.947376	2.117981	0.963827
H	-1.170479	1.746346	1.643245
H	-2.045002	3.203930	1.096166
C	-2.741601	-2.483120	1.251278
C	-2.259590	-3.734464	0.843840
H	-1.979897	-3.893953	-0.198640
C	-2.130716	-4.776151	1.754190
H	-1.760065	-5.742901	1.419552
C	-2.471278	-4.581055	3.092434
H	-2.365507	-5.394446	3.807123
C	-2.948707	-3.343053	3.507705
H	-3.219613	-3.182535	4.549490
C	-3.089487	-2.300342	2.591213
H	-3.467114	-1.337550	2.934784
C	-4.091584	-1.834571	-1.159221
C	-5.143950	-2.632135	-0.692416
H	-5.194848	-2.911208	0.359629
C	-6.128042	-3.086734	-1.564808
H	-6.938439	-3.707409	-1.187834
C	-6.070822	-2.756705	-2.917518
H	-6.838291	-3.116759	-3.599527
C	-5.020688	-1.977931	-3.394390
H	-4.961364	-1.726942	-4.451567
C	-4.035857	-1.523976	-2.519945
H	-3.205365	-0.926028	-2.895122
C	0.090272	2.973621	-0.643066
C	-0.112315	4.282206	-1.085207
H	-1.086832	4.578227	-1.471981
C	0.932916	5.200570	-1.036625
H	0.774659	6.218005	-1.387529
C	2.175484	4.813761	-0.541141
H	2.993529	5.530357	-0.502539
C	2.370570	3.510709	-0.088759
H	3.336223	3.192482	0.302523
C	1.331915	2.583125	-0.145003
H	1.483063	1.557510	0.198194
H	-0.379916	-2.107477	-0.980974
H	0.816895	-0.224225	-1.419970
H	-0.007807	-0.526353	0.810317
H	-1.156556	-0.570076	-2.420606
O	4.392616	1.249760	1.142378
C	5.343234	1.778916	2.066141
H	5.975265	0.951329	2.443223
H	3.705869	0.750654	1.625076

O	6.010822	-1.402787	-1.183139
C	7.079101	-0.497394	-0.897085
H	7.010043	-0.184732	0.163550
H	6.144330	-2.210826	-0.669080
C	1.131305	-0.748120	2.042089
O	2.267716	-0.487277	1.546231
C	0.797546	-2.200433	2.271575
H	1.148039	-2.797436	1.422754
H	-0.267511	-2.394282	2.418693
H	1.342091	-2.526256	3.172318
C	0.504449	0.272381	2.948636
C	-0.652553	0.005895	3.689068
C	1.105184	1.528320	3.074323
C	-1.209490	0.982635	4.507383
C	0.554144	2.504462	3.897123
C	-0.611717	2.237777	4.611221
H	-1.137202	-0.965107	3.618523
H	2.006988	1.740741	2.506863
H	-2.116428	0.760022	5.067274
H	1.038293	3.476315	3.978954
H	-1.048230	3.000000	5.253177
C	4.653651	2.437520	3.243360
H	4.025884	3.274210	2.904219
H	4.016714	1.724363	3.783881
H	5.393018	2.829487	3.952233
C	6.219394	2.747919	1.306838
H	7.001251	3.151332	1.960475
H	6.705955	2.256725	0.455416
H	5.623374	3.588810	0.925859
C	8.418735	-1.160680	-1.133553
H	8.535591	-2.049135	-0.499472
H	8.509377	-1.470883	-2.182943
H	9.240614	-0.472381	-0.903223
C	6.868930	0.707288	-1.784482
H	6.894841	0.405897	-2.840291
H	5.899270	1.185127	-1.585489
H	7.655621	1.452856	-1.619410
C	1.154617	-3.998567	-3.200145
H	1.211537	-4.098335	-4.291174
H	1.298219	-4.991552	-2.753342
H	0.147387	-3.645978	-2.942845
C	3.611185	-3.511652	-3.012696
H	4.374298	-2.816592	-2.637194
H	3.793291	-4.492525	-2.553190
H	3.745474	-3.609055	-4.096205
C	4.695244	-1.560393	3.522515
H	3.819944	-0.896680	3.491676
H	4.408795	-2.479145	4.053103
H	5.483556	-1.062189	4.101650
C	6.368691	-2.823016	2.150285
H	6.127088	-3.742007	2.701034
H	6.676605	-3.113736	1.137341
H	7.222731	-2.340093	2.640319
C	1.898129	2.095217	-3.698641
H	1.798100	1.862537	-4.768804
H	1.222485	1.434957	-3.138560
H	1.576835	3.133958	-3.549334
C	4.294242	2.776319	-4.039592
H	5.328522	2.618378	-3.711853
H	4.232132	2.546830	-5.112471
H	4.047854	3.837160	-3.904936

F. Cation solvation

Li-6iPrOH

E (BS1) = -1173.00372053

E (BS2) = -1173.423792

$G_{298.15,1M}$ (BS2) = -1172.835510

Li	0.145832	0.848088	0.121980
H	-2.371373	0.254785	-0.082831
O	-1.702860	0.818910	-0.529961
C	-1.898771	0.657117	-1.941173
H	-2.959881	0.864188	-2.165350
H	-0.075554	2.965297	1.390034
O	0.497533	2.733024	0.644113
C	1.832985	3.171961	0.961604
H	2.382129	3.076807	0.013324
O	1.704265	0.236801	-0.866262
C	2.628265	0.797458	-1.807599
H	2.263113	1.819465	-1.997415
H	2.132298	-0.514527	-0.406327
C	2.455518	2.266530	2.002621
H	1.870206	2.296025	2.932733
H	2.495621	1.228459	1.648614
H	3.477395	2.590497	2.235017
C	1.809851	4.620426	1.391927
H	1.340623	5.251421	0.629130
H	1.246405	4.731022	2.329250
H	2.826965	4.988802	1.566995
C	-1.039248	1.684605	-2.638960
H	-1.149489	1.605161	-3.726454
H	-1.317906	2.700706	-2.336457
H	0.022258	1.530636	-2.397166
C	4.013647	0.858153	-1.201289
H	4.367327	-0.157054	-0.966559
H	4.726850	1.306814	-1.902025
H	4.022513	1.447433	-0.276116
C	2.604813	0.005535	-3.098003
H	1.588030	-0.058240	-3.504972
H	3.247936	0.473306	-3.853057
H	2.973844	-1.016031	-2.926796
C	-1.567151	-0.763109	-2.351088
H	-0.512084	-0.983544	-2.133752
H	-2.190291	-1.483682	-1.804464
H	-1.737174	-0.913742	-3.424229
O	0.205450	-0.400764	1.615368
C	-0.880504	-0.979145	2.349428
H	-1.717377	-0.278383	2.212619
C	-0.521619	-1.071959	3.816817
H	-0.243158	-0.089741	4.216342
H	-1.364760	-1.456500	4.402961
H	0.327223	-1.755608	3.956719
C	-1.255772	-2.323189	1.764344
H	-2.134998	-2.738977	2.271240
H	-1.480122	-2.233706	0.692944
H	-0.429328	-3.038910	1.885619
H	0.947214	-1.037054	1.560298
H	-5.782830	-0.719071	0.900100
O	-3.761821	-0.714725	0.476911
C	-5.094922	-0.295488	0.149664
C	-5.491080	-0.790300	-1.224552
C	-5.115382	1.210817	0.249060
H	-5.414631	-1.882989	-1.289439
H	-4.844017	-0.350863	-1.996305
H	-6.527311	-0.512738	-1.450514

H	-6.129108	1.590633	0.081937
H	-4.782533	1.543382	1.238895
H	-4.454794	1.655015	-0.507751
H	-3.702361	-1.671648	0.353527
H	2.198945	-3.822870	1.129041
O	2.510439	-1.798738	0.835418
C	2.484772	-3.141807	0.311284
C	3.849105	-3.519362	-0.216398
C	1.427899	-3.165790	-0.766089
H	4.611943	-3.464884	0.569608
H	4.141211	-2.845225	-1.033223
H	3.839940	-4.543704	-0.605419
H	1.280785	-4.188018	-1.130252
H	0.467664	-2.793614	-0.388418
H	1.734183	-2.540540	-1.616311
H	3.272476	-1.703031	1.424446

Na-6iPrOH
E (BS1) = -1327.72862861
E (BS2) = -1328,.62631
$G_{298.15,1M}$ (BS2) = -1327.580148

Na	0.144321	1.174247	0.251209
H	-2.695355	0.513063	-0.110025
O	-1.979696	1.017829	-0.554458
C	-2.080006	0.782927	-1.960638
H	-3.090360	1.078114	-2.295976
H	0.841838	3.558068	1.530601
O	1.212565	3.176329	0.721134
C	2.643020	3.141011	0.877312
H	3.024251	2.918191	-0.130433
O	1.750757	0.072335	-0.955114
C	2.719402	0.429720	-1.945289
H	2.494678	1.477097	-2.202238
H	2.048590	-0.745267	-0.508469
C	3.039748	2.030302	1.827019
H	2.586605	2.195280	2.814703
H	2.714421	1.050927	1.449925
H	4.128562	1.996981	1.956691
C	3.151021	4.493459	1.323408
H	2.831893	5.281397	0.632283
H	2.767250	4.733400	2.325150
H	4.245717	4.500434	1.373524
C	-1.061859	1.667832	-2.644149
H	-1.136358	1.570811	-3.733211
H	-1.214053	2.721589	-2.382451
H	-0.037997	1.376079	-2.362712
C	4.116518	0.335746	-1.369456
H	4.328945	-0.700718	-1.067308
H	4.868059	0.628440	-2.111791
H	4.232193	0.979623	-0.489262
C	2.559617	-0.432940	-3.180428
H	1.527718	-0.403482	-3.552538
H	3.224689	-0.092715	-3.983308
H	2.815569	-1.478014	-2.952670
C	-1.854697	-0.683488	-2.264306
H	-0.840678	-0.976920	-1.953388
H	-2.578435	-1.312455	-1.728083
H	-1.956994	-0.886844	-3.337315
O	0.258559	-0.480450	1.815539
C	-0.830184	-1.089049	2.515043
H	-1.533003	-0.266508	2.716609
C	-0.342943	-1.680411	3.820625
H	0.137848	-0.918991	4.444956
H	-1.170644	-2.121379	4.388511
H	0.390343	-2.475414	3.619365
C	-1.511165	-2.124183	1.645425
H	-2.418829	-2.506648	2.128020
H	-1.784586	-1.691938	0.673197
H	-0.839760	-2.977734	1.470664
H	0.894176	-1.176683	1.550795
H	-6.098666	-0.207601	1.045388
O	-4.075740	-0.320198	0.643216
C	-5.379036	0.177560	0.304193
C	-5.786025	-0.287780	-1.077056
C	-5.309883	1.682178	0.409221
H	-5.775221	-1.383003	-1.146141
H	-5.099999	0.114325	-1.835792
H	-6.799231	0.054367	-1.318405
H	-6.291354	2.126049	0.209901
H	-4.988927	1.991974	1.410365
H	-4.596689	2.083550	-0.323463
H	-4.076443	-1.279733	0.525492
H	1.494971	-4.015684	1.035174
O	2.261571	-2.122489	0.701470
C	1.943324	-3.439156	0.208699
C	3.196430	-4.130027	-0.276841
C	0.926592	-3.252538	-0.891229
H	3.932851	-4.237168	0.528942
H	3.653852	-3.554315	-1.093017
H	2.961273	-5.131945	-0.653768
H	0.536393	-4.222428	-1.218324
H	0.084457	-2.639285	-0.547955
H	1.384471	-2.756659	-1.758030
H	3.021080	-2.180764	1.298922

K-6iPrOH
E (BS1) = -1765.32513964
E (BS2) = -1765.784998

$G_{298.15,1M}$ (BS2) = -1765.199254

K	0.653435	-0.074606	0.227824
H	3.192638	-2.245161	0.229754
O	2.244581	-2.312714	0.405031
C	1.720821	-3.384403	-0.389411
H	2.259321	-4.312636	-0.136557
H	-0.821009	2.547463	1.421090
H	1.076898	1.330012	3.164912
O	0.919291	0.426705	2.861023
O	-1.196712	2.006387	0.711339
C	0.086759	-0.228085	3.824748
H	0.613155	-0.247416	4.793948
C	-1.646931	2.875367	-0.339754
H	-2.241800	2.219334	-0.996761
O	0.360069	0.178223	-2.441563
C	-1.038282	0.038314	-2.727070
H	-1.533592	0.262872	-1.768233
H	0.852671	-0.035787	-3.246820
O	-1.985116	-0.620584	0.508492
C	-3.313273	-1.129189	0.613719
H	-3.636617	-1.066214	1.669403
H	-2.002246	0.331019	0.726205
C	-1.227279	0.508745	3.980907
H	-1.857537	0.027459	4.738389
H	-1.065516	1.549689	4.289504
H	-1.773485	0.509327	3.027058
C	-0.116619	-1.645626	3.340903

H	-0.706370	-1.650601	2.411859
H	0.844777	-2.140347	3.157540
H	-0.665464	-2.229348	4.088804
C	-0.462454	3.420548	-1.107841
H	0.175136	4.020943	-0.443461
H	0.137001	2.603768	-1.532225
H	-0.794349	4.067533	-1.929347
C	-2.533115	3.963956	0.221669
H	-3.367959	3.539246	0.790790
H	-1.955440	4.617761	0.890537
H	-2.943657	4.585600	-0.582129
C	-3.277901	-2.581244	0.200212
H	-2.590286	-3.155574	0.833214
H	-4.274679	-3.029016	0.283456
H	-2.946181	-2.671037	-0.843980
C	-4.253774	-0.306920	-0.243128
H	-3.958714	-0.370142	-1.300933
H	-5.286492	-0.664480	-0.152628
H	-4.235370	0.749486	0.057922
C	0.266546	-3.546743	-0.008091
H	-0.188434	-4.369832	-0.572005
H	0.158625	-3.761829	1.062142
H	-0.313312	-2.639572	-0.234735
C	-1.477172	1.040115	-3.772162
H	-1.009399	0.814015	-4.740878
H	-2.565561	1.006134	-3.907760
H	-1.196740	2.060430	-3.484991
C	-1.362888	-1.385797	-3.125846
H	-1.086518	-2.094401	-2.334294
H	-2.434268	-1.500791	-3.333063
H	-0.813774	-1.659654	-4.038768
C	1.901729	-3.086270	-1.863753
H	1.389681	-2.149534	-2.129287
H	2.964649	-2.982791	-2.117200
H	1.484403	-3.891305	-2.481358
O	2.478969	1.916460	0.039204
C	3.726266	1.213676	0.083733
H	3.641067	0.557902	0.965568
C	4.872709	2.179986	0.289755
H	4.712589	2.790904	1.185016
H	5.823077	1.645070	0.401476
H	4.962403	2.851335	-0.575751
C	3.906575	0.368898	-1.161773
H	4.850587	-0.188354	-1.124761
H	3.092963	-0.358445	-1.280988
H	3.927656	1.010732	-2.054108
H	2.491244	2.485963	-0.743354

TMA-6iPrOH

E (BS1) = -1379.57901213

E (BS2) = -1380.08762695

$G_{298.15,1M}$ (BS2) = -1379,345421

H	2.715798	-0.227568	0.686465
O	2.299634	0.653868	0.800327
C	3.267506	1.557883	1.330475
H	2.725032	2.507420	1.463373
H	-0.596194	3.534224	-2.231848
H	3.168081	-2.632728	0.757496
O	3.191851	-1.873992	0.155923
O	-0.012847	2.794304	-2.011263
C	4.392852	-1.952764	-0.627784
H	4.411581	-1.002672	-1.184881
C	0.712749	3.156761	-0.832341
H	1.351871	2.285113	-0.622106
O	-0.118403	0.481019	2.189581
C	-0.193456	-0.663740	3.023238
H	0.167477	-0.409177	4.038880
H	0.790331	0.561709	1.838649
O	-3.273233	-1.755508	-0.206558
C	-4.688056	-1.973830	-0.073921
H	-5.141691	-1.162501	-0.663035
H	-2.824929	-2.441523	0.311039
C	0.659234	-1.799989	2.489463
H	0.308754	-2.105424	1.491501
H	1.712523	-1.499550	2.408435
H	0.606976	-2.672882	3.151479
C	-1.652255	-1.056808	3.110458
H	-1.796954	-1.895854	3.801624
H	-2.265534	-0.214623	3.451823
H	-2.015808	-1.356024	2.116111
C	-5.066063	-3.310366	-0.672120
H	-4.729218	-3.384190	-1.712306
H	-4.604450	-4.126909	-0.099325
H	-6.152419	-3.456981	-0.648989
C	-5.107560	-1.845640	1.375101
H	-4.638075	-2.636781	1.978353
H	-4.805653	-0.874055	1.784727
H	-6.194890	-1.945151	1.478558
C	-0.229979	3.371303	0.333705
H	-0.884421	4.233573	0.134383
H	-0.861235	2.490453	0.508839
H	0.324358	3.581135	1.257761
C	1.581288	4.368673	-1.099454
H	2.244315	4.195467	-1.955323
H	0.955621	5.245036	-1.322071
H	2.200530	4.614055	-0.227445
C	5.605843	-2.042157	0.271789
H	6.530541	-2.030720	-0.316595
H	5.638776	-1.205896	0.981158
H	5.583896	-2.979146	0.845994
C	4.308770	-3.110745	-1.597647
H	5.204294	-3.156259	-2.228381
H	4.233132	-4.060249	-1.049744
H	3.433302	-3.018976	-2.251346
C	3.755165	1.061898	2.675604
H	2.915172	0.942062	3.371649
H	4.248702	0.085221	2.558993
H	4.477804	1.755198	3.122087
C	4.392757	1.760159	0.337800
H	5.086413	2.538897	0.676649
H	4.966493	0.829821	0.214738
H	3.998666	2.051732	-0.644569
N	0.179595	-0.924426	-2.144487
C	-0.837864	-0.813642	-3.227646
H	-1.740097	-1.344338	-2.910877
H	-1.056729	0.244243	-3.395832
H	-0.433366	-1.264757	-4.137295
C	1.432381	-0.222612	-2.554561
H	1.836314	-0.715198	-3.443321
H	1.185604	0.821435	-2.770699
H	2.144027	-0.276221	-1.723721
C	0.472444	-2.362338	-1.880152
H	1.267801	-2.419241	-1.129991
H	-0.441727	-2.840594	-1.515460
H	0.794247	-2.832700	-2.813497
C	-0.351158	-0.291920	-0.899003
H	-0.544731	0.763996	-1.112494

H	-1.272633	-0.808608	-0.618231
H	0.400321	-0.387316	-0.114547
H	-5.033085	1.101906	-0.007651
O	-3.133395	0.943505	0.803351
C	-4.053361	1.617495	-0.045818
C	-3.564072	1.620955	-1.481534
C	-4.223665	3.016908	0.499132
H	-3.440096	0.595206	-1.855025
H	-2.593549	2.134396	-1.553670
H	-4.276032	2.133940	-2.140325
H	-4.959089	3.577736	-0.088874
H	-4.561479	2.991274	1.541418
H	-3.268432	3.558185	0.462217
H	-3.063255	0.023896	0.486880

***
